# Osthole Induces Apoptosis and Caspase-3/GSDME-Dependent Pyroptosis via NQO1-Mediated ROS Generation in HeLa Cells

**DOI:** 10.1155/2022/8585598

**Published:** 2022-06-08

**Authors:** Juan Wang, Mengjie Huangfu, Xiaojuan Li, Mengjie Han, Guoxiang Liu, Dan Yu, Luwei Zhou, Tong Dou, Yisa Liu, Xiao Guan, Riming Wei, Xu Chen

**Affiliations:** ^1^College of Pharmacy, Guilin Medical University, 541004 Guilin, China; ^2^Guangxi Key Laboratory of Molecular Medicine in Liver Injury and Repair, The Affiliated Hospital of Guilin Medical University, Guilin 541001, China; ^3^Guangxi Health Commission Key Laboratory of Basic Research in Sphingolipid Metabolism Related Diseases, The Affiliated Hospital of Guilin Medical University, Guilin 541001, China; ^4^Xiangya School of Medicine of Central South University, 410013 Hunan, China; ^5^Institute of Biotechnology, Guilin Medical University, 541004 Guilin, China

## Abstract

Osthole is a natural coumarin which has been proved to inhibit growth of cancer cells by inducing cell death, while its mechanism was considered to be just caused by apoptosis. In our study, we found that osthole activated not just apoptosis, but also pyroptosis which is a form of regulated cell death accompanied by loss of cell membrane integrity and lactate dehydrogenase (LDH) release. Caspase-3 is a key protein of apoptosis as well as pyroptosis. The apoptosis and pyroptosis induced by osthole were all inhibited by irreversible caspase-3 inhibitor Z-DEVD-FMK. Meanwhile, knockdown of gasdermin E (GSDME) only reduced the osthole-induced pyroptosis but did not affect the occurrence of apoptosis. Our proteomic analysis revealed that the expression of NAD(P)H: quinone oxidoreductase 1 (NQO1) was decreased in osthole-treated cells. Moreover, NQO1 inhibition by osthole induced the overproduction of reactive oxygen species (ROS), as well as apoptosis and pyroptosis. ROS inhibitor N-Acetyl-L-cysteine (NAC) not only reduced osthole-induced apoptosis but also reversed its effect on the pyroptosis. Meanwhile, knockdown of NQO1 by si-NQO1 or its inhibitor dicoumarol (DIC) not only enhanced ROS generation but also strengthened the GSDME-mediated pyroptosis. Finally, we demonstrated that osthole inhibited tumor growth and the expression of NQO1 in a HeLa xenograft mode. Similar to the results *in vitro*, osthole stimulated the activation of caspase-3, PARP, and GSDME *in vivo*. Taken together, all these data suggested that osthole induced apoptosis and caspase-3/GSDME-mediated pyroptosis via NQO1-mediated ROS accumulation.

## 1. Introduction

Cervical cancer (CC) is the fourth most common cancer in women, and the mortality rate of CC is also ranked fourth in female tumors [1]. Chemotherapy is the standard treatment for advanced or recurrent patients. However, the occurrence and development of chemotherapy resistance may affect the treatment effect [2]. Therefore, finding effective drugs is urgent for cervical cancer therapy.

Osthole, a natural coumarin extracted from the *Cnidium monnieri (L.) Cusson.*, has been shown to have inhibitory effect in multiple sorts of cancers, including breast cancer [3], ovarian cancer [4], and hepatic carcinomas [5]. In addition, osthole is used as an adjunct therapy to conquer cisplatin resistance and enhance radiotherapy sensitivity in cervical cancer cells [6, 7], and it induced apoptosis in cervical cancer cells [8]. However, very few studies are available to address whether it can induce other forms of cell death. Thus, our study attempts to reveal whether osthole induces other cell death forms besides apoptosis and explore its potential mechanism.

Pyroptosis is a form of regulated cell death mediated by gasdermin family [9]. The N-terminal domain of gasdermins is pore-forming effector which causes membrane permeabilization and cell membrane rupture [10]. The cell membrane damaged by the N-terminal domain of GSDME during pyroptosis, which causes cellular contents release and then stimulates inflammation, initiates an antitumor immune response. Thus, pyroptosis may enhance the antitumor effects for some drugs [11]. The existence of pyroptosis pathway may be an important way for drugs to kill some tumor cells resistant to apoptosis. The level of reactive oxygen species (ROS) in cancer cells is higher than normal cells, but when the production of ROS rises to the level of cytotoxicity, that can suppress the cancer cells and even induced them to death [12]. Moreover, ROS overproduction occurs in many forms of regulated cell death, including necrosis [13–15], apoptosis [16], autophagy-dependent cell death, and pyroptosis [17]. However, the relationship between ROS production and cell death triggered by osthole remains poorly defined.

In this study, we show that osthole induces apoptosis and GSDME-mediated pyroptosis by increasing generation of ROS through inhibiting NQO1, thereby inhibiting the proliferation of cervical cancer cells.

## 2. Material and Methods

### 2.1. Reagents and Antibodies

Osthole and cisplatin (DDP) were purchased from Push biotechnology Co., Ltd (purity ≥ 99%, Chengdu, China). Dicoumarol (DIC), Z-VAD-FMK, Z-DEVD-FMK, and 3-methyladenine (3-MA) were purchased from MedChem Express (Princeton, NJ, USA). N-Acetyl-L-cysteine (NAC) and JC-1 were purchased from Beyotime (Shanghai, China). Dimethyl sulfoxide (DMSO) and 2′,7′-dichlorofluorescin diacetate (DCFH-DA) were purchased from Sigma-Aldrich (St. Louis, MO, USA). Anti-rabbit IgG, anti-mouse IgG, and antibodies against *β*-actin were purchased from ZSGB-BIO (Beijing, China). Anti-NQO1, anti-caspase-3, anti-cleaved caspase-3, anti-PARP-1, and anti-GSDME antibodies were purchased from Abcam (Cambridge, MA, USA). FITC Annexin V Apoptosis Detection Kit was purchased from BD (BD Pharmingen, CA, USA).

### 2.2. Cell Cultures

The human cervical cancer HeLa cell line (whose RIP3 is deficient, supplementary Figure S3) and normal liver cell line LO2 were obtained from Conservation Genetics CAS Kunming Cell Bank (Kunming, China). The cells were cultured in DMEM (Gibco, Grand Island, NY, USA) supplemented with 10% fetal bovine serum (FBS; Gibco, Auckland, New Zealand), 100 U/mL penicillin, and 100 *μ*g/mL streptomycin (Solarbio, Beijing, China) and maintained in a humid environment with 5% CO_2_ at 37°C.

### 2.3. Cell Viability Assay

Hela cells were seeded into 96-well plates at 4000 cells/well and incubated overnight. Osthole was dissolved in DMSO and then diluted with cell culture medium to the desired final concentration (40, 80, 160, 200, 240, 320, 400, 480, and 600 *μ*M). The following day, cells were treated with osthole at indicated concentrations for 12 h, 24 h, or 48 h, respectively. Then, 20 *μ*L MTT (Solarbio, Beijing, China) was added to each well, and the plate was incubated for 4 h at 37°C. The formed formazan crystals were dissolved with 150 *μ*L DMSO, and the absorbance was detected at 490 nm with a microplate reader (TECAN, Switzerland).

### 2.4. Transmission Electron Microscopy (TEM)

Cells were harvested in 1.5 mL EP tubes and fixed with 3% glutaraldehyde overnight at 4°C and postfixed with 1% osmium tetroxide for 2 h. Samples were gradually dehydrated in ethanol-acetone solution, and dehydrated samples were passed through acetone and epoxy resin (Epon812) penetrating solution successively for 1 h, with ratios of 3 : 1, 1 : 1, and 1 : 3, respectively, using a Reichert ultramicrotome (Leica, Wetzlar, Germany) to cut the sections, poststained with 0.5% uranyl acetate and 0.3% lead citrate and examined by TEM (JEOL, Tokyo, Japan).

### 2.5. Lactate Dehydrogenase (LDH) Release Assay

The LDH release was detected by LDH assay kit (Nanjing Jiancheng Bioengineering Institute, Nanjing, China). According to the instruction, cell culture supernatant was collected and corresponding reagents were added. The absorbance was measured at 450 nm with a microplate reader.

### 2.6. Flow Cytometry Analysis of Annexin V-FITC/PI Staining

Cells were harvested and washed by cold phosphate-buffered saline (PBS) thrice. The cells were suspended in 200 *μ*L 1× binding buffer containing 5 *μ*L Annexin V-FITC and 5 *μ*L propidium iodide (PI) and incubated for 30 min in the dark. After that, 100 *μ*L 1× binding buffer was supplemented to each sample, and the cells were detected by flow cytometer (BD Biosciences, CA, USA).

### 2.7. Measurement of ROS

After being treated with osthole, cells were washed with PBS and dyed by DCFH-DA (2 *μ*M) for 30 min at 37°C in the dark. The cells were harvested, and the level of ROS was measured by flow cytometry (BD Biosciences, USA).

### 2.8. Measurement of MMP

Mitochondrial membrane potential was determined by the fluorescent probe JC-1 (Beyotime Biotech, Nanjing, China). The cells were suspended in PBS containing 0.2 *μ*M JC-1. After being incubated for 20 min in the dark, the samples were analyzed by flow cytometry. JC-1 existed as a polymer when the MMP is high otherwise exists as a monomer, and the two groups of cells can be distinguished by flow cytometry (BD Biosciences, USA).

### 2.9. Label-Free Quantitative Proteomics

Protein extraction and peptide separation were performed as previous described method [18]. MS experiments were performed on a Q Exactive mass spectrometer that was coupled to Easy nLC (ThermoFisher Scientific, Bonn, Germany). MS data was acquired using a data-dependent top 10 method dynamically choosing the most abundant precursor ions from the survey scan (350–1800 m/z) for HCD fragmentation. The instrument was run with peptide recognition mode enabled. MS experiments were performed triply for each sample.

### 2.10. Bioinformatics Analysis of Differentially Expressed Proteins

The MS raw data were processed using MaxQuant software version 1.5.5.1 and quantified by label-free quantitation (LFQ) [19]. Gene Ontology (GO) enrichment analysis with biological process, molecular function, and cellular components of potential targets was carried out for biological function annotation based on a bioinformatics database (http://geneontology.org/). The KOALA (KEGG Orthology and Links Annotation) software was used to analyze KEGG pathway database. Fisher's exact test was used to the distribution of GO classification or KEGG pathway in target protein collection and total protein collection.

### 2.11. siRNA Transfection

The siRNAs were synthesized by General Bio Co., Ltd (Hefei, China), and sequences for NQO1 (sense: 5′GAUUCUUAAUGAAAAAAGATT 3′ and antisense: 5′ UCUUUUUUCAUUAAGAAUCTT 3′) and GSDME (sense: 5′CCUAUUUGAUGAUGAACUATT 3′ and antisense: 5′ UAGUUCAUCAUCAAAUAGGTT 3′) were shown as above. For siRNA knockdown, HeLa cells were cultured to 60% confluence at the time of transfection. Transfection of siRNA was performed using the Lipofectamine®3000 transfection reagent (Invitrogen, Carlsbad, CA, USA) according to the manufacturer's protocol. The transfected cells were grown in antibiotic-free medium for 24 h and then reseeded for further 24 h before drug treatments. The knockdown efficiency was examined by western blotting.

### 2.12. Western Blotting Analysis

The cells were harvested and lysed with RIPA lysate buffer for 30 minutes on ice. After being centrifuged, the supernatant was obtained and quantified using BCA kit. The protein was separated by SDS-PAGE electrophoresis and transferred to a NC membrane. These membranes were blocked with 5% skim milk for 2 h and were incubated with primary antibody at 4°C overnight. After that, membranes were incubated with horseradish peroxidase-conjugated secondary antibody (1 : 4000) for 1 h at room temperature. Protein bands were detected by Western ECL substrates (Bio-Rad, CA, USA), and gray values were analyzed by Image J.

### 2.13. Xenograft Assay

The use of all mice in this study was approved by the Institutional Animal Care and Use Committee of Guilin Medical University. Female BALB/c-nude mice (4 weeks old) were purchased from Hunan SJA Laboratory Animal Co., Ltd. HeLa cells (1 × 10^7^) were injected subcutaneously into the right flanks of the mice. When the tumor volume reached 50 mm^3^, nude mice were intraperitoneally injected with osthole (150 mg/kg, dissolved in corn oil), and control group mice were injected with corn oil (Sigma, St. Louis, MO, USA). The length and width of the tumor were measured every two days using vernier calipers (volume = length × width^2^/2). Following treatment for 21 days, the mice were sacrificed, and the weights of xenograft tumors were measured. After that, the total protein from the excised tumor tissues was extracted by RIPA lysis buffer. The expression of proteins was detected by western blotting analysis.

### 2.14. Statistical Analysis

All data represent at least three independent experiments and were analyzed by the GraphPad Prism 8 software (GraphPad, San Diego, CA). The results were expressed as mean ± standard deviation (SD). Differences among groups were compared using a two-tailed Student's *t*-test or one-way ANOVA. *P* < 0.05 was considered statistically significant.

## 3. Results

### 3.1. Osthole Induces Apoptotic and Nonapoptotic Death in HeLa Cells

**Figure 1:**
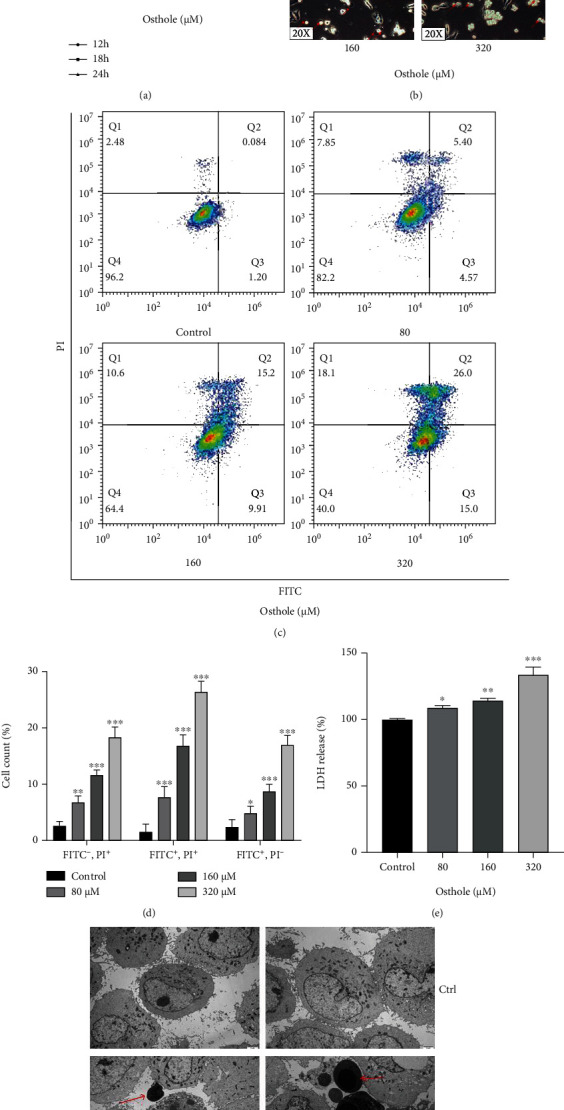
Osthole induces apoptotic and nonapoptotic death in HeLa cells. (a) MTT assay showed that the cell viability was decreased after treatment with osthole at advisable time. (b) Representative bright-field microscopy images (20x, red arrows indicated the large bubbles emerging from the plasma membrane). (c) Representative flow cytometry histograms of Annexin V-PI-stained cells after treatment 18 h in absence or presence of osthole. (d) Percentage (%) of cells stained by Annexin V-FITC and/or PI. (e) LDH release was detected after treatment 18 h in absence or presence of osthole. (f) Representative TEM image (15000x, red arrows indicate autolysosomes; black arrows indicate rupture of plasma membrane). All Data are represented as means ± SD; *n* = 3; ^∗^*P* < 0.05, ^∗∗^*P* < 0.01, and ^∗∗∗^*P* < 0.001 compared with the control group.

In order to detect the effect of osthole on cell viability, HeLa cells were treated with indicated concentrations of osthole for indicated time. Our result showed that osthole induced dose- and time-dependent reduction of cell viability in HeLa cells (Figure 1(a)), and 400 *μ*M osthole did not show obvious inhibition on normal LO2 cells (Figure S1A). The phase-contrast images demonstrated that osthole-treated cells became round, shrink, or to float (Figure 1(b)). Thus, we detected the cell death by Annexin FITC/PI staining using flow cytometry. We found that osthole-induced form of cell death was different from cisplatin-induced apoptosis (Figure S1B). The percentage of early apoptotic (Q3) and late apoptotic/secondary necrotic (Q2) and of nonapoptotic cells (Q1) increased with increasing concentrations of osthole (Figures 1(c) and 1(d)). Therefore, osthole not only induced apoptotic but also nonapoptotic death in HeLa cells.

To further determine the osthole caused necrosis in HeLa cells, we detected the release of LDH which indicates a breakdown of the cell membrane, and the result showed that osthole increased LDH release (Figure 1(e)). Meanwhile, we observed the cell morphology by TEM, and it showed that osthole-treated cells presented vesiculation of organelles and rupture of plasma membrane which were features of nonapoptotic death (Figure 1(f), black arrow). Furthermore, the treated osthole caused HeLa cell nuclear chromatin condensation, which is the typical ultrastructural marker of apoptosis (Figure 1(f), red arrow). These data suggested that osthole induced both apoptosis and a type of death associated with loss of plasma membrane integrity in HeLa cells.

### 3.2. Osthole Induces GSDME-Mediated Pyroptosis in HeLa Cells

**Figure 2:**
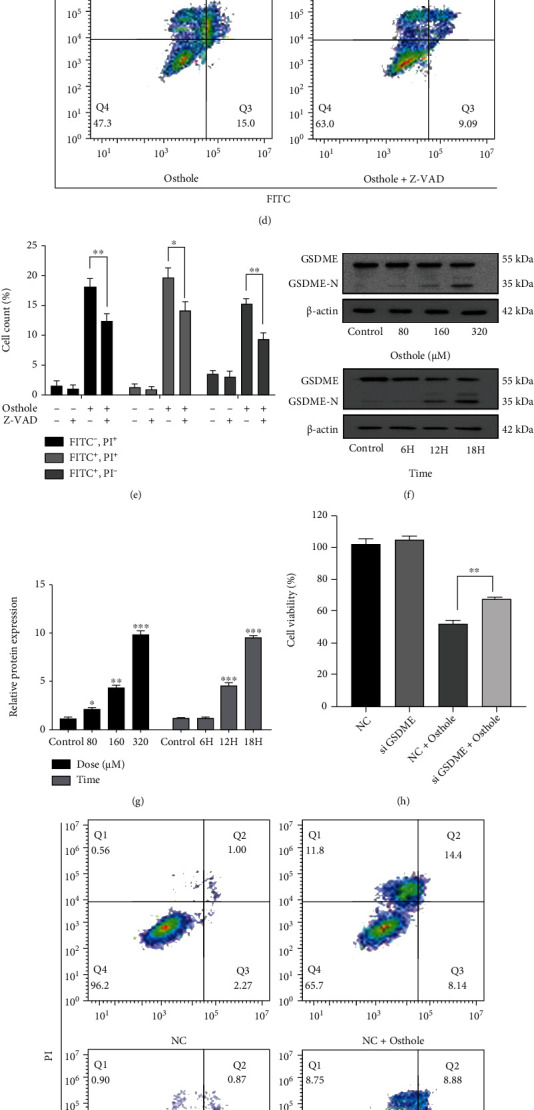
Osthole induces GSDME-mediated pyroptosis in HeLa cells. (a) After treatment with osthole, the levels of apoptosis-related protein were determined by western blotting. (b) The intensity of bands was quantified by Image J, and *β*-actin was used as a control. (c–e) Pretreatment with 20 *μ*M Z-VAD-FMK for 1 h prior to treatment with 320 *μ*M osthole for 18 h. (c) Cell viability was detected by MTT assay. (d) Cell death was determined using Annexin V-FITC/PI double staining by flow cytometry. (e) Percentage (%) of cells stained by Annexin V-FITC and/or PI. (f) The expression of GSDME and GSDME-N was analyzed by western blotting at protein levels. (g) The intensity of bands was quantified by Image J, and *β*-actin was used as a control. (h–l) GSDME was knocked down in HeLa cells. (h) Cell viability was detected by MTT assay. (i) Cell death was detected using Annexin V-FITC/PI staining by flow cytometry. (j) Percentage (%) of cells stained by Annexin V-FITC and/or PI. (k) The expression of GSDME and GSDME-N was analyzed by western blotting at protein levels. (l) The intensity of bands was quantified by Image J, and *β*-actin was used as a control.

Then, we detected the expression of apoptosis indicators caspase-3 and PARP-1. We found that osthole increased the expression of cleaved caspase-3 and cleaved PARP-1 (Figures 2(a) and 2(b)). Pretreatment with pan caspase inhibitor Z-VAD-FMK prevented osthole-induced decrease in cell viability (Figure 2(c)). To our surprise, pretreatment with Z-VAD-FMK not only decreased the osthole-induced apoptotic but also nonapoptotic death (Figures 2(d) and 2(e)). As shown in Figure 1(b), the phase-contrast images showed that the dying cells blew characteristic large bubbles from the plasma membrane which was the typical characteristics of pyroptosis [20, 21]. Gasdermin E (GSDME) is a member of gasdermin family, and the N-terminal domain of GSDME (GSDME-N) is cleaved by apoptotic caspase-3 and causes the induction of pyroptosis [22]. Thus, we detected the expression of GSDME which is the biomarker of caspase-3-mediated pyroptosis. The result showed that the expression of GSDME-N in osthole-treated cells was significantly higher than untreated cells (Figures 2(f) and 2(g)).

To further confirm the role of GSDME in osthole-induced pyroptosis, we knocked down GSDME by siRNA. MTT results showed that knockdown of GSDME partly attenuated osthole-induced cell viability decrease compared to osthole-treated NC (negative control) cells. Moreover, Annexin V-FITC and PI staining showed that knockdown of GSDME reduced the necrosis but not apoptosis (Figures 2(i) and 2(j)). Western blotting analysis also illustrated that knockdown of GSDME reduced the expression of GSDME-N compared to osthole-treated NC cells (Figures 2(k) and 2(l)).

### 3.3. Caspase-3 Contributes to Osthole-Induced Apoptosis and Pyroptosis in HeLa Cells

**Figure 3:**
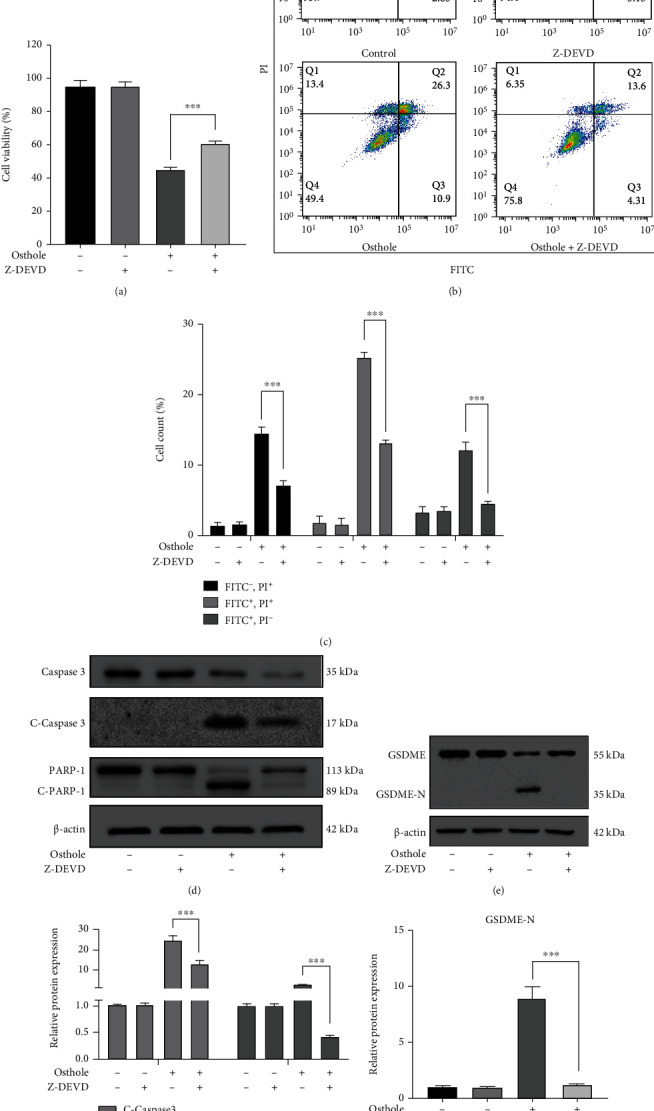
Activation of caspase-3 triggers the apoptosis and GSDME-mediated pyroptosis. (a–g) Pretreatment with 50 *μ*M Z-DEVD-FMK for 1 h prior to treatment with 320 *μ*M osthole. (a) MTT assay was used to detect the cell viability. (b) Annexin V-FITC/PI double staining was used to analyze the cell death. (c) Percentage (%) of cells stained by Annexin V-FITC and/or PI. (d) The expression of apoptosis-related proteins was detected by western blotting. (e) The intensity of bands was quantified by Image J, and *β*-actin was used as a control. (f) The expression of GSDME and GSDME-N was detected by western blotting at protein levels. (g) The intensity of bands was quantified by Image J, and *β*-actin was used as a control.

HeLa cells were pretreated with caspase-3 inhibitor Z-DEVD-FMK prior to observe the effect of osthole on pyroptosis. We found that Z-DEVD-FMK partially reversed the osthole-induced loss of cell viability (Figure 3(a)), so as the apoptotic and nonapoptotic death (Figures 3(b) and 3(c)). Western blotting analysis showed that pretreatment with Z-DEVD-FMK reversed both the expression of cleaved caspase-3 and cleaved PARP-1 (Figures 3(d) and 3(f)) and the expression of GSDME-N (Figures 3(e) and 3(g)). All these results indicated that caspase-3 mediates apoptosis and pyroptosis in osthole-treated cells.

### 3.4. Proteomic Analysis of Osthole-Treated HeLa Cells

**Figure 4:**
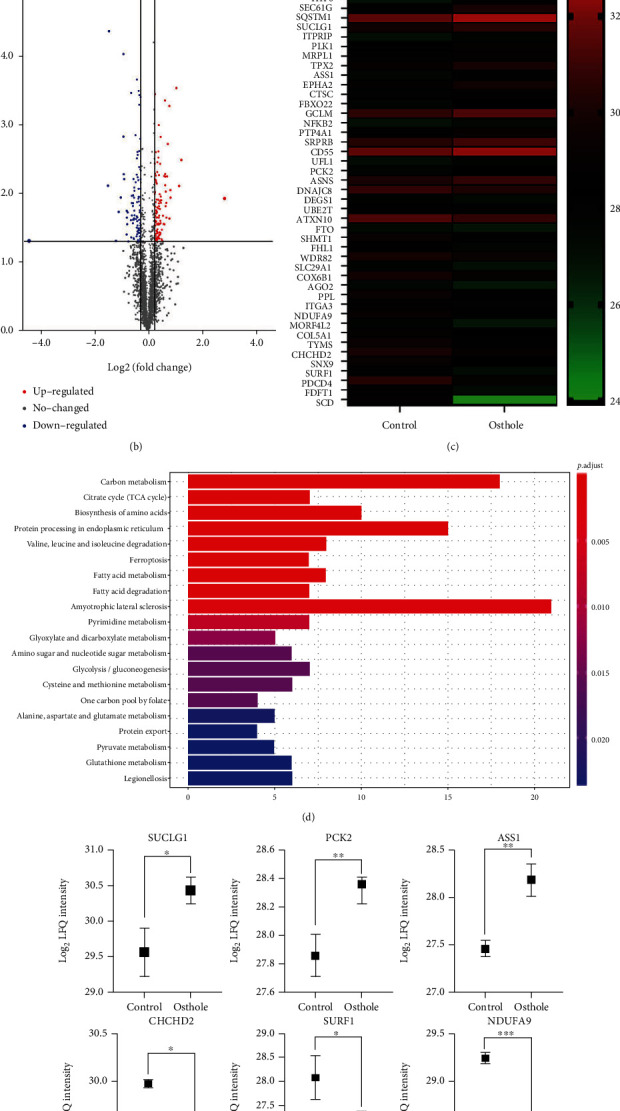
Label-free quantitative proteomic analysis. (a) Venn diagram shows the coincident proteins and special protein between treated and untreated groups. (b) Volcano plot shows the difference in expression between treated and untreated cells. Red dots represent the upregulated proteins, and blue dots represent downregulated protein while grey dots represent unchanged proteins (log2 LFQ > 1.2, *P* < 0.05). (c) Heat map of differentially expressed proteins (log2 LFQ > 1.2, *P* < 0.05). (d) KEGG pathway analysis of differentially expressed proteins. (e) Mitochondria-related differentially expressed proteins. All data are represented as means ± SD; *n* = 3; ^∗^*P* < 0.05, ^∗∗^*P* < 0.01, and ^∗∗∗^*P* < 0.001 compared with the control group.

Label-free quantitative proteomic technique was used to identify the target protein of osthole in HeLa cells. Our analysis 3,015 expressed proteins (in the form of 35340 unique peptides) (Figure 4(a)). The differential expression between the osthole-treated group and control group was presented by volcano plot (Figure 4(b)). As shown in Figure 4(c), osthole upregulated 21 proteins and downregulated 22, corresponding to a total of 43 differentially expressed proteins (DEPs). We found that the LFQ intensity of pyroptosis gene GSDME was only detected in osthole-treated sample, and the LFQ intensity of apoptosis-related gene caspase-3 in osthole-treated cells was three folds higher than the control group (supplementary table 1). Moreover, KEGG database was used to enrich differentially expressed proteins, and the top 20 pathways were screened (Figure 4(d)). The first 2 pathways were carbon metabolism and TCA cycle which mainly occurred in mitochondria. Next, we found that osthole treatment caused the mitochondria-associated proteins SUCLG1, PCK2, and ASS1 expression increased and CHCHD2, SURF1, and NDUFA9 expression decreased (Figure 4(e)). Thus, according to the above analysis, we speculated that osthole-caused cell death may be closely related to functional changes of mitochondria.

### 3.5. ROS Generation Contributes to the Osthole-Induced Apoptosis and Pyroptosis

**Figure 5:**
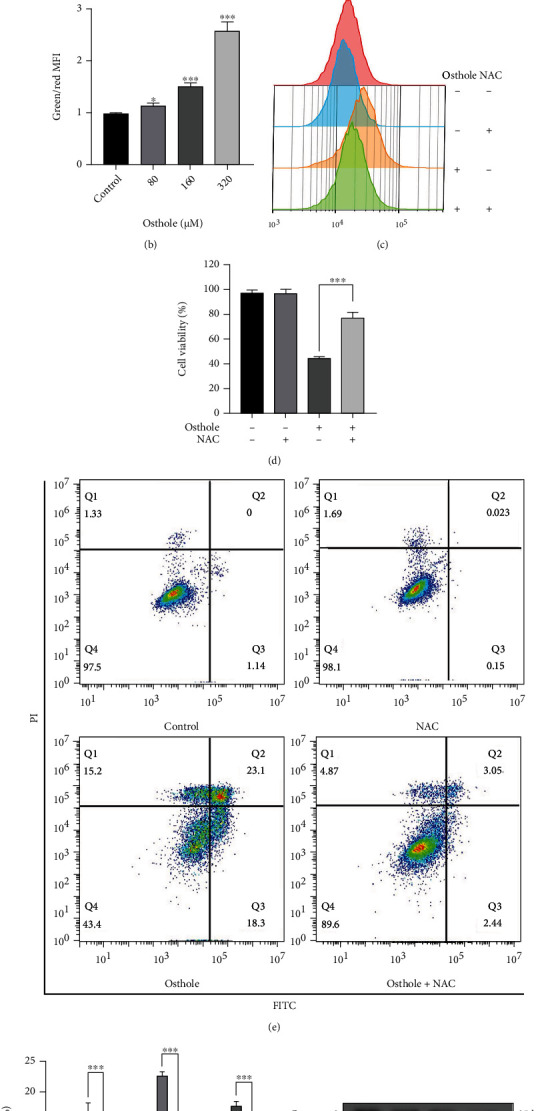
ROS initiates osthole-induced pyroptosis. (a) The JC-1 staining was analyzed by flow cytometry after treatment with osthole for 18 h. (b) The ratio between the green and red mean fluorescence intensity quantified by FlowJo. (c) After pretreatment with 0.2 *μ*M NAC for 1 h and treatment with osthole for 4 h, DCHF-DA staining was detected by flow cytometry. (d) Cell viability was detected by MTT assay. (e) Annexin V-FITC/PI staining was used to detect cell death by flow cytometry. (f) Percentage (%) of cells stained by Annexin V-FITC and/or PI. (g) The expression of apoptosis- and pyroptosis-related proteins was determined by western blotting. (h) The intensity of bands was quantified by Image J, and *β*-actin was used as a control. All data are represented as means ± SD; *n* = 3; ^∗∗∗^*P* < 0.001 compared with the control group.

Our proteomic analysis showed that the changes of mitochondria function may be the main cause of osthole-induced HeLa cell death. Then, we firstly detected the MMP by JC-1 staining using flow cytometry. The result showed that the red fluorescence of JC-1 transforms to green fluorescence as the osthole concentration increased (Figures 5(a) and 5(b)). Then, DCFH-DA staining result showed that osthole increased the production of ROS, but this effect was reversed by the ROS inhibitor NAC (Figure 5(c)). Moreover, pretreatment with NAC reduced the loss of cell viability caused by osthole (Figure 5(d)). Annexin V-FITC/PI staining showed that NAC reversed almost all apoptosis and necrosis (Figures 5(e) and 5(f)). Western blotting analysis demonstrated that NAC inhibited the increased expression of cleaved caspase-3, cleaved PARP-1, and GSDME-N (Figures 5(g) and 5(h)).

### 3.6. Inhibition of NQO1 Increases the Production of ROS and Promotes Apoptosis and Pyroptosis in HeLa Cells

**Figure 6:**
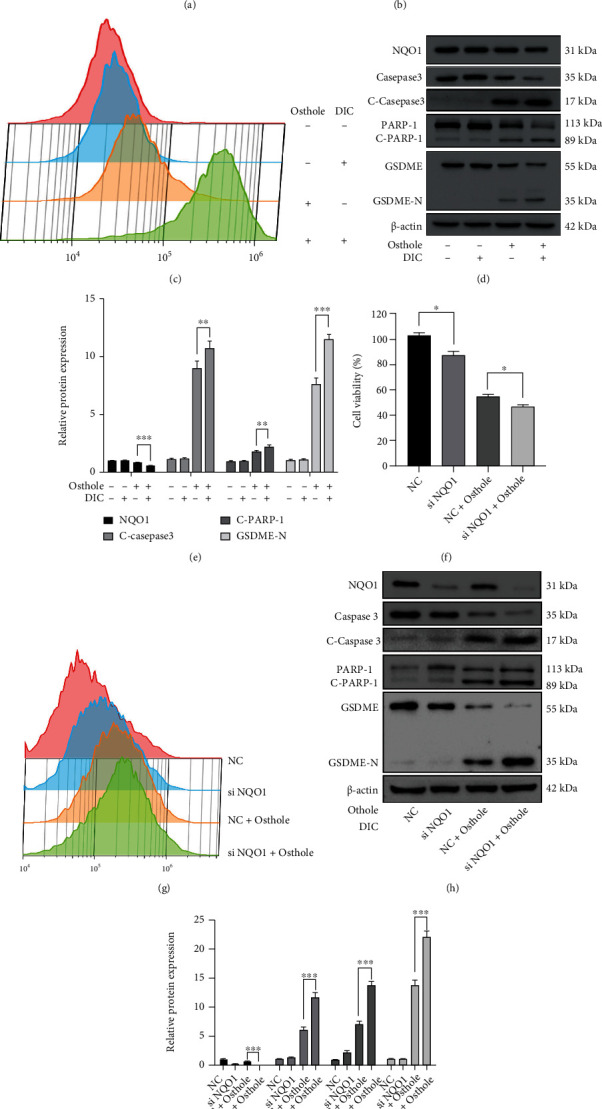
NQO1 contributes to ROS overproduction and cell death in HeLa cells. (a) The differences of NQO1 expression were detected by label-free proteomic analysis. (b–e) Incubation with 100 *μ*M DIC for 1 h ahead of treatment with 320 *μ*M osthole. (b) Cell viability was detected by MTT assay. (c) Treatment with osthole for 4 h, DCFH-DA staining was detected by flow cytometry. (d) Western blotting analysis for the expression of apoptosis- and pyroptosis-related proteins. (e) The intensity of bands was quantified by Image J, and *β*-actin was used as a control. (f–i) NQO1 was knocked down in HeLa cells. (f) Cell viability was detected by MTT assay. (g) DCHF-DA staining was detected by flow cytometry. (h) Western blotting analyzed the expression of apoptosis- and pyroptosis-related proteins. (i) The intensity of bands was quantified by Image J, and *β*-actin was used as a control. All data are represented as means ± SD; *n* = 3; ^∗^*P* < 0.5, ^∗∗^*P* < 0.01, and ^∗∗∗^*P* < 0.001 compared with the control group.

Our proteomics analysis showed that osthole-treated HeLa cells expressed lower NQO1 than the control group (Figure 6(a)). NQO1 activity and protein expression were inhibited in the osthole-treated group (Figure S2). Pretreatment with NQO1 inhibitor dicoumarol (DIC) enhanced the loss of cell viability and inhibition of NQO1 activity by osthole (Figure 6(b) and Figure S2). It has been reported that inhibiting NQO1 could increase ROS production [23]. In this study, we found that pretreated with DIC strengthened the generation of ROS induced by osthole (Figure 6(c)). In addition, we found that DIC pretreatment enhanced the expression of cleaved caspase-3 and cleaved PARP-1 and GSDME-N (Figures 6(d) and 6(e)). Moreover, knockdown of NQO1 individually not only reduced cell viability but also increased ROS production. Meanwhile, knockdown of NQO1 strengthened the loss of cell viability and ROS production induced by osthole (Figures 6(f) and 6(g)). Western blotting analysis showed that knockdown of NQO1 increased osthole-induced NQO1 expression reduction. The expression of cleaved caspase-3, cleaved PARP-1, and GSDME-N was increased in osthole-treated siNOQ1 cells (Figures 6(h) and 6(i)).

### 3.7. Osthole Suppresses HeLa Xenograft Tumor Growth *In Vivo*

**Figure 7:**
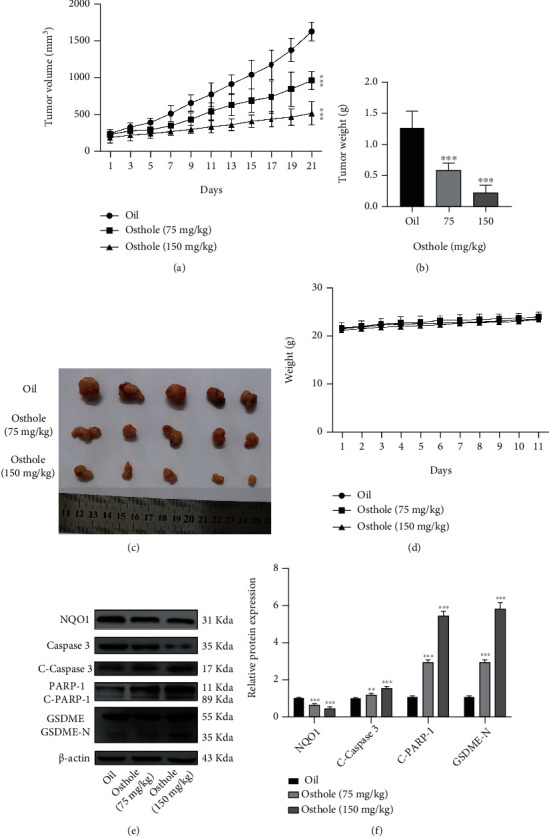
Osthole suppresses HeLa xenograft tumor growth, induces apoptosis and pyroptosis *in vivo*. (a) Tumor volume was recorded every 2 days. (b) The average weight of tumor in control and osthole-treated groups. (c) Representative images of unstripped tumor after being treated for 21 days. (d) The body weight of nude mice treated with or without osthole for 21 days. (e) The expression proteins were determined by western blotting. (f) The intensity of bands was quantified by Image J, and *β*-actin was used as a control. All data are represented as means ± SD; ^∗^*P* < 0.001, ^∗∗^*P* < 0.001, and ^∗∗∗^*P* < 0.001 compared with the control group.

To explore the antitumor effect of osthole on cervical cancer *in vivo*, HeLa cells were xenografted into BALB/c nude mice. After being treated with osthole for 21 days, the tumor size and tumor weight of nude mice were all significantly reduced than the control group (Figures 7(a) and 7(b)).  Figure 7(c) showed the pictures of tumor after peeling. Moreover, we found that nude mouse body weight in the osthole-treated group had no significant change compared with the control group (Figure 7(d)). Further, western blotting analysis revealed that osthole increased the protein expression of cleaved caspase-3 and GSDME-N and decreased the NQO1 in protein expression (Figures 7(e) and 7(f)). These results suggested that osthole suppressed the tumor proliferation, and the mechanism may be associated with the NQO1-mediated cell apoptosis and pyroptosis.

## 4. Discussion

In this study, we clarified the underlying mechanism on osthole-induced cell death, including apoptosis and pyroptosis in HeLa cells *in vitro* and *in vivo*. Our data suggested that osthole significantly increased the production of ROS by inhibiting the expression of NQO1 in HeLa cells. Moreover, the generation of ROS activated the caspase-3, and activated caspase-3 induced apoptosis and pyroptosis. Thus, our findings illustrated that osthole induced apoptosis and pyroptosis via ROS overproduction in HeLa cells.

Previous studies reported that osthole induced cell death mainly in the form of apoptosis [4, 24, 25], and a recent study demonstrated that osthole induced cell death through the GSDME-dependent pyroptosis pathway [26]. We also found that osthole-induced dying cells present cell membrane rupture, increased LDH release, and the formation of plasma membrane bubbles, which are the typical features of pyroptosis [27]. Pyroptosis is triggered primarily by GSDMD and involves inflammatory caspase-1 (canonical pathway) activation or caspase-4/5 (or mouse caspase-11) (noncanonical pathway) [28], which caused cytokines IL-18 and IL-1*β* released [29]. But, the cleaved GSDMD cannot be detected in osthole-induced HeLa cells (supplementary Figure S4). Among the alternative pathways, the most widely studied is GSDME-induced pyroptosis through caspase-3 [30]. GSDME is a member of the gasdermin family which contains a cytotoxic N-terminal domain to form pore and induce membrane permeabilization. The activation of caspase-3 mediates the occurrence of apoptosis and specifically cleaves GSDME and generates a GSDME-N fragment to induce pyroptosis [31]. In this study, caspase-3 inhibitor Z-DEVD-FMK not only reduced the apoptosis, but also the GSDME-mediated pyroptosis induced by osthole. Moreover, proteomic results also found that the expression of caspase-3 and GSDME was also elevated by osthole. Therefore, osthole-caused cell death was induced by caspase-3-mediated apoptosis and pyroptosis.

Though our and others' study found that osthole induced cancer cell death, the protein targets of osthole induced cancer cell death have not been revealed. Then, we tried to find the main protein targets of osthole-induced HeLa cell death. Unfortunately, our proteomic analysis demonstrated that the differential expressed proteins between osthole and control were hardly related with cell death. But some ROS generation proteins had been significantly changed in the osthole-treated group, included NQO1, which NQO1 was recently identified as an antitumor target of osthole through a network-based method [32]. NQO1 was highly expressed in diverse human cancers, including breast cancer, colon cancer, cervical cancer, and lung cancer [33]. It is reported that the role of NQO1 in cancer cells is to prevent excessive oxidative stress from damaging cells which is consistent with that in normal cells. So, NQO1 expression inhibition can disrupt intracellular redox balance, such as ROS overproduction in cancer cells [23, 34]. It has reported that osthole inhibited the NQO1 expression to overcome cisplatin resistance in cervical cancer SiHa and CaSki cells [7]. Considering these reports and our proteomic profiling, we evaluated the relationship between NQO1 expression and the death induction of osthole in HeLa cells as well as the production of ROS. We found that osthole suppressed the expression of NQO1, and the inhibition of NQO1 using DIC (NQO1 inhibitor) or siNQO1 enhanced the apoptosis and pyroptosis induction in HeLa cells. Meanwhile, the generation of ROS triggered by osthole was also increased by suppression of NQO1. Based on these reports and results, we conjectured that osthole-induced apoptosis and pyroptosis may be related with ROS generation.

ROS overproduction occurs in many forms of regulated cell death, including apoptosis, necroptosis, ferroptosis, and pyroptosis [35]. It has been reported that osthole-induced antitumor effects are relevant to ROS overproduction [36], and our earlier report also proved that osthole induced ROS overproduction in glioma cells [37]. Previous reports showed that apoptosis and pyroptosis share part of the upstream signal pathway, including ROS and caspase-3 [22]. In our study, we found that ROS inhibitor NAC could reduce osthole-induced apoptosis and pyroptosis via inhibition of caspase-3. Overproduction of ROS mediates DNA damage and activates PARP-1, which is downstream of activated caspase-3 [38]. Moreover, Yu et al. demonstrate that ROS plays a critical function in GSDME-mediated cell death [15]. Our study indicated that osthole-induced ROS overproduction mediated the events of apoptosis and GSDME-related pyroptosis. Meanwhile, osthole decreased NQO1 expression which caused the elevation of ROS generation in HeLa cells, and the effect of osthole on ROS production was enhanced by NQO1 inhibitor DIC and NQO1 siRNA.

In summary, ROS mediated the occurrence of apoptosis and pyroptosis in osthole-treated HeLa cells, and NQO1 expression plays an important role in the death induction and ROS generation caused by osthole. Taken together, all these data suggest that osthole induces apoptosis and caspase-3/GSDME-mediated pyroptosis through inhibition of NQO1 and ROS accumulation. Osthole may be a potential therapeutic agent for cervical cancer treatment. Future studies are thus needed to determine the contribution of these two processes to the antineoplastic effects of osthole and discover other cell death form induced by osthole.

## Data Availability

The data used to support the findings of this study are included within the article and the supplementary information files.

## Supplementary Materials

Supplementary Materials Figure S1: the effect of osthole on LO2 cells and the comparison of cell death types. (A) Cell viability of LO2 cells assessed by MTT assay after being treated with osthole for 18h. (B) Cell death was detected by Annexin V-FITC/PI staining by flow cytometry after being treated with DDP and osthole. Figure S2: the expression and activity of NQO1 were inhibited by osthole in HeLa cells. (A) The protein expression of NQO1 was decreased by osthole in HeLa cells. (B) The NQO1 activity was detected used human NQO1 ELISA kit. Figure S3: the protein expression of RIP3 was detected in U87 and HeLa cell. Figure S4: the activation of caspase-5, caspase-1, and GSDMD was detected in osthole-treated cells.
