# Supplementary material for: Osthole Induces Apoptosis and Caspase-3/GSDME-Dependent Pyroptosis via NQO1-Mediated ROS Generation in HeLa Cells
Source: Oxid Med Cell Longev. 2022 Jun 8;2022:8585598. doi: 10.1155/2022/8585598 (PMC9200556; doi:10.1155/2022/8585598)
Supplement: Supplementary Materials — Figure S1: the effect of osthole on LO2 cells and the comparison of cell death types. (A) Cell viability of LO2 cells assessed by MTT assay after being treated with osthole for 18h. (B) Cell death was detected by Annexin V-FITC/PI staining by flow cytometry after being treated with DDP and osthole. Figure S2: the expression and activity of NQO1 were inhibited by osthole in HeLa cells. (A) The protein expression of NQO1 was decreased by osthole in HeLa cells. (B) The NQO1 activity was detected used human NQO1 ELISA kit. Figure S3: the protein expression of RIP3 was detected in U87 and HeLa cell. Figure S4: the activation of caspase-5, caspase-1, and GSDMD was detected in osthole-treated cells. [file 8585598.f1.zip › supplementary table 1 (1).pdf]

Protein IDs	Gene Name	Fasta headers	Number of prot	Peptides	Razor + unique	Unique pe	Sequence cov	Mol. weight [kDa]	LFQ intensity N1	LFQ intensity N2	LFQ intensity N3	LFQ intensity O1	LFQ intensity O2	LFQ intensity O3
sp|A0A02	NUDT4B	sp|A0A02	2	4	4	3	32	20.434	1271400000	0	0	0	1464500000	1533600000
sp|Q9Y53	PPIAL4A	sp|Q9Y53	5	3	1	1	29	18.182	0	0	0	0	0	88828000
sp|P0DPI	GATD3A	sp|P0DPI	2	5	5	5	30	28.17	504530000	384590000	394500000	472040000	644230000	495730000
sp|A0A0C	IGKV1-12	sp|A0A0C	1	1	1	1	17	12.645	0	0	0	0	313010000	0
sp|A0AVT	UBA6	sp|A0AVT	1	21	21	21	26	117.97	997210000	1282600000	899440000	1137700000	1186800000	1016400000
sp|A0FGR	ESYT2	sp|A0FGR	1	9	9	9	14	102.36	211090000	255030000	264560000	311370000	234600000	233320000
sp|A0JLT	MED19	sp|A0JLT	1	2	2	2	21	26.273	0	0	0	0	0	32156000
sp|A0MZ6	SHTN1	sp|A0MZ6	1	6	6	6	13	71.639	225060000	191510000	0	226570000	223480000	0
sp|A1L0T	ILVBL	sp|A1L0T	1	7	7	7	19	67.867	271700000	248670000	326650000	345190000	265560000	187680000
sp|A1L15	TSPAN11	sp|A1L15	1	1	1	1	3.2	28.245	0	0	0	0	0	325900000
sp|A1X28	SH3PXD2	sp|A1X28	1	3	3	2	3.2	101.58	57145000	64497000	0	0	70814000	79743000
sp|A2A3N	PIPSL	sp|A2A3N	2	3	2	2	4.8	95.047	0	0	0	0	79478000	0
sp|A3KMH	VWA8	sp|A3KMH	1	3	3	3	2.9	214.82	0	0	0	0	0	41823000
sp|A3KN8	SBNO1	sp|A3KN8	1	4	4	4	4.4	154.31	0	0	79631000	0	0	57983000
sp|A4D1E	GTPBP10	sp|A4D1E	1	3	3	3	15	42.932	132030000	0	82881000	84058000	115240000	69105000
sp|A5YKK	CNOT1	sp|A5YKK	1	24	24	24	13	266.94	792400000	961250000	876170000	782180000	690360000	766000000
sp|Q9GZ	MAP1LC3	sp|Q9GZ	3	2	2	2	17	14.688	265450000	348940000	273230000	512850000	376930000	262640000
sp|A6NDG	PGP	sp|A6NDG	1	8	8	8	37	34.006	598380000	370420000	463860000	382890000	441490000	309330000
sp|A6NDR	MEIS3P1	sp|A6NDR	4	1	1	1	4	30.204	0	0	0	0	0	50886000
sp|A6NDU	C5orf51	sp|A6NDU	1	3	3	3	18	33.62	187660000	274420000	0	137500000	0	0
sp|A6NHL	TUBAL3	sp|A6NHL	1	5	1	1	11	49.908	0	0	0	0	23857000	0
sp|A6NHQ	FBLL1	sp|A6NHQ	1	2	1	1	6.6	34.803	0	0	0	0	0	740750000
sp|A6NHR	SMCHD1	sp|A6NHR	1	8	8	8	6.1	226.37	320920000	272290000	0	299240000	309340000	337490000
sp|A6NKD	CCDC85C	sp|A6NKD	1	1	1	1	4.3	45.209	0	0	0	0	0	90920000
sp|A6NKT	RGPD3	sp|A6NKT	4	18	1	1	13	197.48	0	0	0	0	0	134930000
sp|A8CG3	POM121C	sp|A8CG3	2	3	3	3	4.1	125.09	0	88746000	33293000	77209000	0	0
sp|A8K8P	SFI1	sp|A8K8P	1	1	1	1	0.6	147.66	0	170180000	0	0	0	0
sp|A8MQ0	CYSRT1	sp|A8MQ0	1	2	2	2	25	15.313	83168000	0	0	0	0	76193000
sp|P62308	SNRPG	sp|P62308	2	2	2	2	25	8.496	2115600000	1934200000	1930900000	1633900000	2234500000	1609100000
sp|A8MXV	NUDT19	sp|A8MXV	1	1	1	1	8	42.233	0	0	0	0	0	17372000
sp|B7ZAP	RABGAP1	sp|B7ZAP	1	2	1	1	10	29.038	0	0	0	0	0	372870000
sp|P0CG0	GPR89B	sp|P0CG0	2	1	1	1	2.6	52.916	0	0	0	0	74044000	0
sp|Q1376	NACA	sp|Q1376	2	5	5	4	33	23.384	7295000000	8535600000	6227800000	5949300000	6370500000	5699000000
sp|E9PRG	C11orf98	sp|E9PRG	1	1	1	1	11	13.798	0	0	0	152130000	0	0
sp|L0R6Q	SLC35A4	sp|L0R6Q	1	4	4	4	48	11.133	368050000	252330000	381750000	443910000	411600000	442300000

sp|L0R819	ASDURF	sp|L0R819	1	1	1	1	10	11.25	0	0	0	0	43716000	0
sp|O00116	AGPS	sp|O00116	1	18	18	18	41	72.911	3498100000	3475400000	3431200000	4632800000	4307800000	5328400000
sp|O00124	UBXN8	sp|O00124	1	1	1	1	6.3	30.541	0	0	0	15520000	0	0
sp|O00139	KIF2A	sp|O00139	1	10	10	9	17	79.954	349060000	425880000	319850000	425210000	351760000	331270000
sp|O00148	DDX39A	sp|O00148	1	18	7	7	49	49.129	1384400000	1321000000	1601500000	1871200000	1938300000	1959700000
sp|O00151	PDLIM1	sp|O00151	1	14	14	14	71	36.071	1612600000	1443100000	1127700000	1507400000	1508600000	1437700000
sp|O00154	ACOT7	sp|O00154	2	9	9	9	30	41.796	2409200000	2516500000	2643400000	2607200000	2622000000	2867700000
sp|O00159	MYO1C	sp|O00159	1	24	24	24	29	121.68	2085900000	2382500000	2324800000	2095500000	2224700000	2775600000
sp|O00161	SNAP23	sp|O00161	1	6	6	6	36	23.354	440450000	363770000	527800000	504700000	534240000	429870000
sp|O00165	HAX1	sp|O00165	1	3	3	3	18	31.62	102250000	0	95599000	115930000	115990000	106470000
sp|O00170	AIP	sp|O00170	1	9	9	9	42	37.636	1306200000	1163300000	1124800000	1119700000	1065100000	835570000
sp|O00178	GTPBP1	sp|O00178	1	3	3	3	7	72.453	0	0	77957000	0	0	0
sp|O00180	KCNK1	sp|O00180	1	1	1	1	5.7	38.143	0	0	86876000	98339000	0	0
sp|O00186	STXBP3	sp|O00186	1	5	5	5	9.8	67.764	76407000	60548000	87726000	72651000	85268000	81877000
sp|O00193	SMAP	sp|O00193	1	4	4	4	26	20.332	954130000	724730000	782880000	841830000	616460000	773200000
sp|O00194	RAB27B	sp|O00194	1	1	1	1	5	24.608	0	0	0	0	13376000	0
sp|O00203	AP3B1	sp|O00203	2	9	9	9	11	121.32	478770000	599930000	514690000	670640000	446270000	431830000
sp|O00214	LGALS8	sp|O00214	1	3	3	3	11	35.808	0	0	0	0	0	109140000
sp|O00217	NDUFS8	sp|O00217	1	3	3	3	18	23.705	202490000	236730000	243090000	170710000	237210000	235760000
sp|O00231	PSMD11	sp|O00231	1	15	15	15	42	47.463	2479300000	2834800000	2872700000	2799500000	3027200000	3277300000
sp|O00232	PSMD12	sp|O00232	1	18	18	18	46	52.904	2956000000	2554300000	2741300000	2711400000	2599600000	3150000000
sp|O00233	PSMD9	sp|O00233	1	8	8	8	41	24.682	679250000	566240000	502280000	630660000	687780000	586430000
sp|O00244	ATOX1	sp|O00244	1	3	3	3	50	7.4016	1136300000	1334800000	1267400000	1203400000	1762200000	1031000000
sp|O00257	CBX4	sp|O00257	1	3	3	3	10	61.367	0	0	36702000	0	0	0
sp|O00264	PGRMC1	sp|O00264	1	9	8	8	58	21.671	2168200000	2320900000	2616400000	2791600000	2637900000	2175000000
sp|O00267	SUPT5H	sp|O00267	1	11	11	11	16	121	558960000	474360000	589040000	418730000	478690000	641380000
sp|O00273	DFFA	sp|O00273	1	8	8	8	30	36.521	848230000	1057300000	745800000	817030000	809740000	930100000
sp|O00299	CLIC1	sp|O00299	1	13	13	13	70	26.922	1.2853E+10	1.451E+10	1.2087E+10	12687000000	13919000000	1.117E+10
sp|O00303	EIF3F	sp|O00303	1	13	13	13	43	37.563	2860500000	3162100000	2793100000	2618700000	2717900000	3253300000
sp|O00308	WWP2	sp|O00308	1	1	1	1	2	98.911	0	0	0	0	0	28899000
sp|O00330	PDHX	sp|O00330	1	5	5	5	16	54.122	292570000	184810000	273220000	178610000	233560000	271100000
sp|O00401	WASL	sp|O00401	1	3	3	3	6.9	54.826	0	0	0	0	164840000	0
sp|O00410	IPO5	sp|O00410	1	36	36	34	54	123.63	1.0424E+10	1.0603E+10	1.1435E+10	10919000000	9729800000	1.0188E+10
sp|O00411	POLRMT	sp|O00411	1	3	3	3	4.5	138.62	0	0	0	0	164780000	0
sp|O00422	SAP18	sp|O00422	1	6	6	6	33	17.561	601250000	697450000	389500000	529110000	753000000	958410000
sp|O00425	IGF2BP3	sp|O00425	1	18	18	16	38	63.704	2717300000	2896100000	2779800000	2646800000	2830900000	2634000000
sp|O00429	DNM1L	sp|O00429	1	24	24	24	53	81.876	1767800000	2019600000	2145100000	1741300000	1658100000	1877400000
sp|O00442	RTCA	sp|O00442	1	3	3	3	14	39.336	123670000	89422000	76924000	0	107770000	142070000
sp|O00458	IFRD1	sp|O00458	1	3	3	3	12	50.268	0	0	0	0	0	154970000

sp|O00461	GOLIM4	sp|O00461	1	8	8	8	14	81.879	342190000	409790000	476160000	480060000	557640000	498740000
sp|O00468	AGRN	sp|O00468	1	3	3	3	2.3	217.23	0	0	0	26884000	0	0
sp|O00469	PLOD2	sp|O00469	1	14	14	14	26	84.685	1564800000	2112900000	1716400000	1505500000	1721600000	1665300000
sp|O00483	NDUFA4	sp|O00483	1	4	4	4	47	9.3697	735290000	695230000	838160000	599670000	608200000	699660000
sp|O00487	PSMD14	sp|O00487	1	7	7	7	41	34.577	1491600000	1923600000	1333300000	1515900000	1607300000	1838700000
sp|O00488	ZNF593	sp|O00488	1	1	1	1	10	15.199	0	0	0	0	0	34222000
sp|O00499	BIN1	sp|O00499	1	9	9	9	25	64.699	329730000	611070000	615400000	398650000	427490000	351320000
sp|O00505	KPNA3	sp|O00505	1	9	7	7	30	57.81	581730000	522730000	644440000	473920000	396030000	577920000
sp|O00507	USP9Y	sp|O00507	1	11	1	1	5.4	291.07	0	0	0	0	48870000	0
sp|O00541	PES1	sp|O00541	1	12	12	12	21	68.002	1086000000	1160200000	1150600000	1081700000	1115800000	1310300000
sp|O00560	SDCBP	sp|O00560	1	9	9	9	51	32.444	674690000	787260000	549830000	637010000	667860000	716830000
sp|O00566	MPHOSPH	sp|O00566	1	7	7	7	19	78.863	451850000	383970000	478440000	391370000	372460000	467350000
sp|O00567	NOP56	sp|O00567	1	22	22	22	48	66.049	3771700000	4120300000	3941500000	4499600000	4616700000	4417600000
sp|O00571	DDX3X	sp|O00571	3	30	29	29	53	73.243	9735600000	1.0143E+10	1.1098E+10	9228500000	9493100000	1.0568E+10
sp|O00592	PODXL	sp|O00592	1	7	7	7	12	58.635	1548200000	1699100000	1187700000	1903100000	1700600000	1856800000
sp|O00625	PIR	sp|O00625	1	8	8	8	43	32.113	1177000000	1674600000	1433800000	1197200000	996030000	1400400000
sp|O00629	KPNA4	sp|O00629	1	11	11	9	39	57.886	1498800000	1432800000	1414000000	1236400000	1337200000	1205900000
sp|O00743	PPP6C	sp|O00743	1	9	9	9	40	35.144	810990000	861790000	742380000	763730000	762100000	651690000
sp|O00762	UBE2C	sp|O00762	1	4	4	4	39	19.652	540370000	340730000	505190000	295420000	0	281300000
sp|O00764	PDXK	sp|O00764	1	11	11	11	44	35.102	2084000000	2325900000	2035400000	2224900000	2317400000	2274800000
sp|O00767	SCD	sp|O00767	1	2	2	2	11	41.522	116060000	499020000	479250000	16272000	20105000	12995000
sp|O14493	CLDN4	sp|O14493	4	1	1	1	7.7	22.077	0	0	0	0	0	60188000
sp|O14497	ARID1A	sp|O14497	1	5	5	4	4	242.04	115670000	104560000	0	0	0	109420000
sp|O14513	NCKAP5	sp|O14513	1	3	3	3	1.8	208.53	0	259680000	0	0	0	0
sp|O14521	SDHD	sp|O14521	1	2	2	2	11	17.043	81334000	116770000	0	143700000	0	0
sp|O14530	TXNDC9	sp|O14530	1	7	7	7	37	26.534	329100000	362990000	372450000	312120000	437020000	369590000
sp|O14548	COX7A2L	sp|O14548	1	2	2	2	40	12.615	0	209280000	302690000	187880000	0	0
sp|O14561	NDUFAB1	sp|O14561	1	4	4	4	21	17.417	926040000	1249900000	1094500000	1024800000	1172400000	727810000
sp|O14562	UBFD1	sp|O14562	1	1	1	1	5.8	33.382	0	0	0	0	0	27404000
sp|O14569	CYB561D	sp|O14569	1	1	1	1	5.4	23.973	0	0	0	0	0	18161000
sp|O14579	COPE	sp|O14579	1	10	10	10	50	34.482	1687400000	1677500000	1494800000	1955500000	1415600000	1235300000
sp|O14617	AP3D1	sp|O14617	1	15	15	15	21	130.16	635370000	693950000	799070000	686800000	918310000	773160000
sp|O14618	CCS	sp|O14618	1	2	2	2	7.7	29.04	0	0	0	80991000	0	0
sp|O14639	ABLIM1	sp|O14639	1	5	5	5	9.8	87.687	130360000	185790000	179790000	0	0	196790000
sp|Q92997	DVL3	sp|Q92997	2	1	1	1	1.7	78.054	0	0	0	0	0	44223000
sp|O14646	CHD1	sp|O14646	1	5	5	5	3.7	196.69	0	85877000	0	0	0	0
sp|O14656	TOR1A	sp|O14656	1	1	1	1	3	37.808	0	0	0	0	0	58361000
sp|O14657	TOR1B	sp|O14657	1	3	3	3	8.6	37.978	82800000	75055000	100360000	85964000	93084000	0
sp|O14672	ADAM10	sp|O14672	1	1	1	1	4	84.141	0	0	0	0	0	44282000

sp|O14684	PTGES	sp|O14684	1	2	2	2	13	17.102	684160000	504420000	1273900000	1559700000	1309700000	1992100000
sp|O14732	IMPA2	sp|O14732	1	2	2	2	7.6	31.321	0	0	0	0	0	58130000
sp|O14734	ACOT8	sp|O14734	1	2	2	2	8.5	35.914	0	0	0	0	89968000	0
sp|O14735	CDIPT	sp|O14735	1	2	2	2	10	23.539	163510000	144590000	188790000	158380000	140840000	212140000
sp|O14737	PDCD5	sp|O14737	1	6	6	6	46	14.285	1956800000	1666700000	2275400000	1740100000	2175500000	2111600000
sp|O14744	PRMT5	sp|O14744	1	18	18	18	39	72.683	2388100000	2235100000	2250500000	2401900000	2228200000	2360700000
sp|O14745	SLC9A3R	sp|O14745	1	12	12	12	39	38.868	2248200000	1989900000	1721000000	2041100000	2305500000	2220000000
sp|O14773	TPP1	sp|O14773	1	2	2	2	5.7	61.247	83196000	0	0	0	0	217810000
sp|O14776	TCERG1	sp|O14776	1	24	24	24	24	123.9	1429900000	1531700000	1489900000	1374400000	1300000000	1521700000
sp|O14777	NDC80	sp|O14777	1	5	5	5	8.6	73.912	146910000	0	196220000	0	126440000	0
sp|O14787	TNPO2	sp|O14787	1	4	1	1	5.4	101.39	0	0	0	0	9895200	0
sp|O14818	PSMA7	sp|O14818	2	12	12	12	53	27.887	4805400000	4927000000	4570800000	4838100000	5554100000	5564900000
sp|O14828	SCAMP3	sp|O14828	1	7	7	7	31	38.287	755550000	1031300000	733200000	670350000	611320000	1057500000
sp|O14879	IFIT3	sp|O14879	1	8	8	8	24	55.984	199430000	212150000	167840000	260840000	209680000	201180000
sp|O14880	MGST3	sp|O14880	1	3	3	3	25	16.516	690110000	477770000	678340000	748700000	393860000	435040000
sp|O14907	TAX1BP3	sp|O14907	1	2	2	2	25	13.735	503360000	330960000	454750000	403540000	502640000	517220000
sp|O14908	GIPC1	sp|O14908	2	7	7	7	33	36.049	475260000	392070000	497810000	303340000	412730000	370660000
sp|O14925	TIMM23	sp|O14925	2	3	3	3	23	21.943	390100000	519660000	340120000	331830000	424700000	326170000
sp|O14929	HAT1	sp|O14929	1	10	10	10	35	49.512	2357700000	2127700000	2486200000	1784400000	1948500000	2033600000
sp|O14936	CASK	sp|O14936	1	5	5	5	7.5	105.12	97844000	178410000	137540000	114210000	125020000	136130000
sp|O14949	UQCRQ	sp|O14949	1	3	3	3	33	9.9062	399150000	394540000	434530000	417250000	521160000	427500000
sp|P19105	MYL12A	sp|P19105	2	8	8	3	63	19.794	7570200000	7087600000	6974800000	7474300000	8589500000	8415800000
sp|O14964	HGS	sp|O14964	1	8	8	8	11	86.191	693160000	441780000	566470000	573600000	597650000	625630000
sp|O14965	AURKA	sp|O14965	1	11	11	11	38	45.809	305790000	326930000	379340000	355290000	389540000	409550000
sp|O14972	DSCR3	sp|O14972	1	5	5	5	21	33.01	123310000	113790000	127540000	96542000	105740000	71293000
sp|O14974	PPP1R12A	sp|O14974	1	15	15	15	21	115.28	1228900000	1106600000	989780000	1039200000	1081600000	1157700000
sp|O14975	SLC27A2	sp|O14975	1	4	4	4	12	70.311	71866000	86429000	89325000	116330000	132680000	85705000
sp|O14976	GAK	sp|O14976	1	6	6	6	6.6	143.19	138750000	196040000	157570000	160330000	159380000	120420000
sp|O14979	HNRNPDL	sp|O14979	1	11	10	10	21	46.437	4476900000	3526000000	3853000000	3836900000	4132600000	3950500000
sp|O14980	XPO1	sp|O14980	1	35	35	35	45	123.38	6086400000	6653800000	6257700000	5733500000	5319900000	6071900000
sp|O14981	BTAF1	sp|O14981	1	2	2	2	1.5	206.89	0	0	0	0	0	62518000
sp|O15013	ARHGEF1	sp|O15013	1	4	4	4	4.3	151.61	33742000	52530000	54864000	31305000	32839000	31360000
sp|O15020	SPTBN2	sp|O15020	1	18	12	12	11	271.32	467990000	417560000	388780000	355690000	372840000	434350000
sp|O15027	SEC16A	sp|O15027	1	29	29	29	25	233.51	1698700000	1618500000	1629800000	1690700000	1401700000	1594300000
sp|O15031	PLXNB2	sp|O15031	1	11	11	11	8.1	205.12	530330000	533430000	690030000	419010000	605220000	548520000
sp|O15042	U2SURP	sp|O15042	1	8	8	8	13	118.29	816770000	644520000	826590000	569360000	526220000	698140000
sp|O15056	SYNJ2	sp|O15056	1	2	2	2	1.7	165.54	0	75116000	0	0	0	0
sp|O15061	SYNM	sp|O15061	1	4	4	4	4.7	172.77	0	61466000	82772000	0	81380000	78354000
sp|O15067	PFAS	sp|O15067	1	34	34	34	39	144.73	4310000000	5048800000	5188300000	4980000000	3747700000	3983600000

sp|O15078	CEP290	sp|O15078	1	2	2	2	0.7	290.38	0	0	0	0	0	661910000
sp|O15084	ANKRD28	sp|O15084	2	2	2	2	2.8	112.96	0	88324000	0	0	0	0
sp|O15091	KIAA0391	sp|O15091	1	2	2	2	7.2	67.315	0	0	0	160900000	0	0
sp|O15116	LSM1	sp|O15116	1	2	2	2	26	15.179	0	0	0	0	26857000	0
sp|O15118	NPC1	sp|O15118	1	2	2	2	1.5	142.17	0	0	0	0	0	51096000
sp|O15121	DEGS1	sp|O15121	1	3	3	3	15	37.866	293890000	237880000	233170000	155970000	179280000	170130000
sp|O15126	SCAMP1	sp|O15126	1	5	5	5	30	37.92	2501400000	1602500000	1467900000	1190500000	1314800000	2065600000
sp|O15127	SCAMP2	sp|O15127	1	1	1	1	6.1	36.648	0	66741000	139630000	0	0	110370000
sp|O15143	ARPC1B	sp|O15143	1	15	15	14	50	40.949	4928900000	3864300000	4754400000	5111300000	4716000000	4180100000
sp|O15144	ARPC2	sp|O15144	1	15	15	15	54	34.333	4193200000	5296400000	3764300000	4055600000	3794700000	3822400000
sp|O15145	ARPC3	sp|O15145	1	9	9	9	53	20.546	2303300000	2816200000	2670400000	2601600000	2442300000	2094400000
sp|O15160	POLR1C	sp|O15160	1	7	7	7	30	39.249	523380000	563940000	495970000	547470000	592260000	576900000
sp|O15162	PLSCR1	sp|O15162	1	1	1	1	6.3	35.049	0	0	0	0	0	31457000
sp|O15164	TRIM24	sp|O15164	1	1	1	1	1	116.83	0	0	27101000	0	0	0
sp|O15173	PGRMC2	sp|O15173	1	10	10	9	40	23.818	2078600000	1781000000	2175900000	2045500000	2696800000	2316000000
sp|O15212	PFDN6	sp|O15212	1	5	5	5	34	14.582	860910000	936190000	1135200000	821790000	1020400000	888840000
sp|O15213	WDR46	sp|O15213	1	3	3	3	7.7	68.07	126720000	150740000	141970000	138280000	120940000	140790000
sp|O15226	NKRF	sp|O15226	1	8	8	8	17	77.672	324100000	234770000	246960000	224740000	405350000	389970000
sp|O15228	GNPAT	sp|O15228	1	1	1	1	2.4	77.187	0	0	0	0	86124000	0
sp|O15231	ZNF185	sp|O15231	1	4	4	4	12	73.525	0	0	0	164680000	0	0
sp|O15243	LEPROT	sp|O15243	1	1	1	1	9.9	14.254	0	0	0	0	0	44346000
sp|O15247	CLIC2	sp|O15247	1	4	4	4	30	28.356	0	93168000	90404000	109960000	100570000	0
sp|O15254	ACOX3	sp|O15254	1	3	3	3	7.4	77.628	0	1407700000	1073700000	1365300000	1246600000	1907600000
sp|O15258	RER1	sp|O15258	1	3	3	3	19	22.958	420380000	463480000	354860000	525110000	489600000	503970000
sp|O15260	SURF4	sp|O15260	1	3	3	3	15	30.394	627250000	708420000	1318400000	972130000	936050000	1675300000
sp|O15269	SPTLC1	sp|O15269	1	6	6	6	17	52.743	0	0	0	0	0	498670000
sp|O15270	SPTLC2	sp|O15270	1	4	4	4	8	62.924	0	31929000	0	0	69187000	41648000
sp|O15294	OGT	sp|O15294	1	15	15	15	22	116.92	894830000	759160000	875320000	714110000	747720000	759460000
sp|O15305	PMM2	sp|O15305	2	9	9	9	42	28.082	537840000	659700000	770320000	935780000	721250000	610890000
sp|O15321	TM9SF1	sp|O15321	1	3	3	3	7.6	68.86	0	0	0	118900000	0	0
sp|O15327	INPP4B	sp|O15327	1	1	1	1	2.3	104.74	0	0	0	0	0	18189000
sp|O15344	MID1	sp|O15344	1	5	5	5	9.1	75.25	222480000	315600000	376200000	329090000	0	283570000
sp|O15347	HMGB3	sp|O15347	1	5	5	5	21	22.98	955410000	1072200000	1131500000	826150000	1075300000	1526600000
sp|O15355	PPM1G	sp|O15355	1	17	17	17	44	59.271	1976100000	1896000000	1815300000	1563500000	1802300000	1608100000
sp|O15357	INPPL1	sp|O15357	3	17	17	17	18	138.6	712340000	836240000	879290000	851660000	724280000	750620000
sp|O15371	EIF3D	sp|O15371	1	16	16	16	47	63.972	3457800000	3824200000	3617000000	3626200000	3339900000	3452500000
sp|O15372	EIF3H	sp|O15372	1	10	10	10	28	39.93	1295600000	1640300000	1303700000	1162800000	1088400000	1221600000
sp|O15379	HDAC3	sp|O15379	1	3	3	3	11	48.847	0	89515000	57395000	0	0	0
sp|O15381	NVL	sp|O15381	1	3	3	3	5.5	95.05	0	0	0	0	42836000	0

sp|O1538	BCAT2	sp|O1538	1	5	5	5	21	44.287	185390000	187690000	236950000	176160000	214080000	203930000
sp|O1539	IPO8	sp|O1539	1	7	7	7	9.8	119.94	257350000	227570000	153630000	240940000	286060000	158730000
sp|O1540	STX7	sp|O1540	1	4	4	4	22	29.815	0	125170000	0	0	103620000	202650000
sp|O1542	SLC16A3	sp|O1542	1	6	6	6	14	49.469	1731000000	2259100000	2076200000	2515800000	2046200000	2497200000
sp|O1543	ABCC4	sp|O1543	1	3	3	3	3.4	149.52	0	0	66432000	58566000	73612000	60129000
sp|O1544	CD3EAP	sp|O1544	1	7	7	7	30	54.985	664220000	656910000	542850000	512190000	646260000	539150000
sp|O1546	P4HA2	sp|O1546	1	14	14	14	34	60.901	1658300000	954970000	1331700000	987250000	989110000	1028900000
sp|O1549	YKT6	sp|O1549	1	6	6	6	33	22.417	527340000	537850000	495450000	504480000	493570000	561820000
sp|O1551	ARPC5	sp|O1551	1	5	5	4	58	16.32	1510900000	1150700000	1749800000	1545200000	1742100000	1285800000
sp|O1551	POLR2D	sp|O1551	1	3	3	3	38	16.311	0	0	158280000	116090000	154220000	0
sp|O1553	TAPBP	sp|O1553	1	2	2	2	9.6	47.625	0	119270000	139700000	120280000	0	0
sp|O1554	RNF113A	sp|O1554	2	2	2	2	6.4	38.787	0	78869000	0	0	81602000	0
sp|O1555	KCNN4	sp|O1555	1	1	1	1	3.5	47.695	0	0	0	0	892700	0
sp|O4314	DHX15	sp|O4314	1	27	27	26	38	90.932	5793700000	6063600000	6039700000	5833600000	5581100000	5731100000
sp|O4314	RNMT	sp|O4314	1	5	5	5	13	54.843	114600000	333020000	0	191070000	173110000	200520000
sp|O4315	TTI1	sp|O4315	1	1	1	1	1.7	122.07	0	0	0	0	0	43537000
sp|O4315	RRP8	sp|O4315	1	4	4	4	13	50.714	180530000	181030000	185220000	251270000	211240000	194090000
sp|O4316	SIPA1L1	sp|O4316	1	2	2	2	2.4	200.03	33934000	0	0	0	0	0
sp|O4316	CYB5B	sp|O4316	1	3	3	3	45	16.332	1210400000	1055800000	1096400000	1615100000	1357200000	1446900000
sp|O4317	PRPF4	sp|O4317	1	13	13	13	36	58.449	961690000	884670000	980780000	712250000	1094900000	1365400000
sp|O4317	PHGDH	sp|O4317	1	21	21	21	44	56.65	1.0137E+10	1.1781E+10	1.0754E+10	12772000000	13523000000	1.4685E+10
sp|O4318	NDUFS4	sp|O4318	1	1	1	1	8.6	20.108	155740000	155790000	178590000	139080000	165260000	131660000
sp|O4323	SEPT4	sp|O4323	1	1	1	1	2.3	55.098	0	0	0	0	0	916560000
sp|O4323	DYNC1LI	sp|O4323	1	9	8	8	31	54.099	447080000	459250000	370280000	361160000	404660000	416470000
sp|O4324	PSMD3	sp|O4324	1	23	23	23	46	60.977	3639100000	3410700000	3727100000	3784200000	3845500000	3548100000
sp|O4325	PAPSS1	sp|O4325	1	11	11	11	23	70.832	879740000	916720000	1192100000	1177900000	988490000	1118600000
sp|O4326	ZW10	sp|O4326	1	7	7	7	15	88.828	159190000	175420000	214260000	219650000	141630000	191860000
sp|O4328	B4GALT5	sp|O4328	1	1	1	1	2.1	45.118	0	0	0	0	0	78764000
sp|O4329	SART1	sp|O4329	1	10	10	10	20	90.254	602520000	480450000	479940000	519920000	613620000	474660000
sp|O4329	GPAA1	sp|O4329	1	2	2	2	3.1	67.622	0	842250000	0	0	216630000	0
sp|O4329	TGFB1I1	sp|O4329	1	3	3	3	11	49.814	60491000	89282000	79148000	105050000	90005000	42721000
sp|O4331	PPIP5K2	sp|O4331	1	2	2	2	1.8	140.41	49955000	0	68551000	57405000	0	0
sp|O4332	EEF1E1	sp|O4332	1	6	6	6	34	19.81	227830000	296050000	276730000	285050000	286510000	273100000
sp|O4337	WDR62	sp|O4337	1	1	1	1	0.8	165.95	0	0	0	0	0	29946000
sp|O4339	HNRNPR	sp|O4339	1	27	27	21	47	70.942	1.2544E+10	1.3613E+10	1.3498E+10	11743000000	12452000000	1.4613E+10
sp|O4339	PRPF3	sp|O4339	1	7	7	7	16	77.528	373040000	513350000	526090000	374710000	522980000	422180000
sp|O4339	TXNL1	sp|O4339	1	11	11	11	57	32.251	1575100000	1447500000	1551400000	1538000000	1611300000	1484300000
sp|O4339	TPD52L2	sp|O4339	1	12	12	12	70	22.237	4867700000	4394000000	4807600000	3714100000	4744100000	4138800000
sp|O4340	EMC8	sp|O4340	1	3	3	3	17	23.773	109390000	0	117310000	147020000	132700000	157090000

sp|O43414	ERI3	sp|O43414	1	3	3	3	8	37.238	0	0	0	0	0	52391000
sp|O43424	GRID2	sp|O43424	1	1	1	1	1.1	113.35	0	0	0	0	0	140590000
sp|O43427	FIBP	sp|O43427	1	2	2	2	6.9	41.878	0	0	0	15192000	0	0
sp|O43432	EIF4G3	sp|O43432	1	11	6	6	9.5	176.65	151380000	192730000	206130000	125750000	0	109550000
sp|O43447	PPIH	sp|O43447	1	6	6	6	44	19.208	1001100000	1002100000	799360000	874730000	1081400000	912260000
sp|O43488	AKR7A2	sp|O43488	2	4	4	4	19	39.589	330940000	284670000	315420000	224910000	304340000	252630000
sp|O43491	EPB41L2	sp|O43491	2	19	19	19	29	112.59	1228600000	1262600000	1128100000	1174300000	1285600000	1150700000
sp|O43504	LAMTOR5	sp|O43504	1	4	4	4	86	9.6138	655860000	524330000	438560000	562090000	773160000	640170000
sp|O43516	WIPF1	sp|O43516	1	1	1	1	3.2	51.258	0	0	0	0	36766000	0
sp|O43567	RNF13	sp|O43567	1	1	1	1	6.3	42.813	0	0	0	0	23885000	0
sp|O43583	DENR	sp|O43583	1	6	6	6	39	22.092	318750000	457780000	415140000	407360000	400130000	444480000
sp|O43592	XPOT	sp|O43592	1	21	21	21	31	109.96	1358600000	1387200000	1485400000	1379600000	1219100000	1355500000
sp|O43598	DNPH1	sp|O43598	1	3	3	3	25	19.108	0	177140000	0	0	0	0
sp|O43615	TIMM44	sp|O43615	1	9	9	9	25	51.355	965320000	808480000	774450000	1293800000	1139200000	1149500000
sp|O43617	TRAPPC3	sp|O43617	1	3	3	3	14	20.274	84272000	0	137950000	119280000	0	154240000
sp|O43633	CHMP2A	sp|O43633	1	1	1	1	4.5	25.104	0	0	0	0	0	98787000
sp|O43638	FOXS1	sp|O43638	3	1	1	1	3.3	35.434	0	0	0	0	0	51012000
sp|O43657	TSPAN6	sp|O43657	1	3	3	3	14	27.563	253440000	227340000	262400000	216760000	243500000	214260000
sp|O43660	PLRG1	sp|O43660	1	11	11	11	38	57.193	714070000	595400000	583690000	659540000	732070000	634580000
sp|O43663	PRC1	sp|O43663	1	7	7	7	21	71.606	81955000	0	0	74725000	66902000	0
sp|O43665	RGS10	sp|O43665	1	2	2	2	15	20.236	0	0	0	65614000	52398000	0
sp|O43670	ZNF207	sp|O43670	1	3	3	3	7.9	50.75	1078700000	1350600000	1112600000	1300500000	1470400000	1256900000
sp|O43674	NDUFB5	sp|O43674	1	3	3	3	17	21.75	85704000	0	82686000	0	0	88406000
sp|O43676	NDUFB3	sp|O43676	1	2	2	2	21	11.402	215340000	249900000	222800000	0	170880000	153670000
sp|O43678	NDUFA2	sp|O43678	1	1	1	1	21	10.921	0	0	0	0	196440000	0
sp|O43681	ASNA1	sp|O43681	1	11	11	11	52	38.792	1097400000	1311500000	1104500000	1687300000	1713200000	1302200000
sp|O43684	BUB3	sp|O43684	1	12	12	12	49	37.154	5257200000	4057500000	4355300000	5212900000	5493000000	4955600000
sp|O43707	ACTN4	sp|O43707	1	61	61	43	68	104.85	9.9294E+10	9.4717E+10	9.5216E+10	91569000000	85936000000	8.7421E+10
sp|O43709	BUD23	sp|O43709	1	4	4	4	24	31.88	268970000	257830000	277710000	243560000	327400000	293960000
sp|O43715	TRIAP1	sp|O43715	1	2	2	2	36	8.7858	0	0	0	0	51832000	0
sp|O43719	HTATSF1	sp|O43719	1	10	10	10	18	85.852	579580000	524450000	391150000	454180000	447200000	485250000
sp|O43747	AP1G1	sp|O43747	1	18	18	18	29	91.35	667530000	551080000	636620000	726100000	695000000	662640000
sp|O43752	STX6	sp|O43752	1	1	1	1	7.5	29.176	0	0	0	0	36353000	0
sp|O43760	SYNGR2	sp|O43760	1	2	2	2	8	24.81	0	0	0	198160000	0	0
sp|O43765	SGTA	sp|O43765	1	7	7	7	27	34.063	897340000	750400000	983870000	928110000	1018800000	1133100000
sp|O43768	ENSA	sp|O43768	1	5	5	4	48	13.389	1181200000	816100000	1179200000	808900000	1214300000	1143300000
sp|O43772	SLC25A20	sp|O43772	1	5	5	5	23	32.943	228780000	187620000	218160000	259350000	247660000	264250000
sp|O43776	NARS	sp|O43776	2	15	15	15	33	62.942	3462200000	4101100000	3271800000	4114200000	4387900000	3773400000
sp|O43795	MYO1B	sp|O43795	1	15	15	15	16	131.98	670360000	451960000	642640000	555760000	589540000	501540000

sp|O4380	SSNA1	sp|O4380	1	1	1	1	11	13.596	0	0	0	0	0	60043000
sp|O4380	SLC25A1	sp|O4380	1	1	1	1	3.9	34.566	0	0	0	0	10740000	0
sp|O4380	NUDT21	sp|O4380	1	11	11	11	45	26.227	1916600000	2214200000	2285000000	1900800000	1894400000	1723300000
sp|O4381	LANCL1	sp|O4381	1	9	9	9	32	45.283	1551300000	1398600000	1435600000	1158100000	1455300000	1200200000
sp|O4381	STRN	sp|O4381	1	4	4	4	9.5	86.131	0	0	0	0	130430000	142410000
sp|O4381	RRP9	sp|O4381	1	10	10	10	26	51.84	433980000	495390000	454300000	555290000	569160000	594700000
sp|O4381	SCO2	sp|O4381	1	2	2	2	11	29.81	0	0	0	0	0	162920000
sp|O4382	AKAP8	sp|O4382	1	5	5	5	14	76.107	147350000	214740000	236150000	148910000	155730000	227250000
sp|O4382	GTPBP6	sp|O4382	1	2	2	2	7.6	56.897	0	0	0	0	0	48621000
sp|O4383	IDH3B	sp|O4383	1	12	12	12	46	42.183	781750000	789550000	717260000	856000000	1237300000	1014100000
sp|O4384	NRDC	sp|O4384	1	17	17	17	18	131.57	1154600000	1083500000	1417800000	1081300000	1089100000	1203000000
sp|O4385	CALU	sp|O4385	1	19	19	19	69	37.106	9358600000	9285200000	8622900000	9441000000	10168000000	9863700000
sp|O4385	EDIL3	sp|O4385	1	7	7	7	18	53.764	473540000	518710000	400570000	468190000	409610000	408540000
sp|O4386	AHCYL1	sp|O4386	1	13	13	6	33	58.951	1614800000	1429300000	1189900000	1389100000	1323100000	1392400000
sp|O4389	KIF1C	sp|O4389	1	2	2	2	2.8	122.95	0	0	54900000	0	72442000	0
sp|O4391	ORC5	sp|O4391	1	4	4	4	9	50.282	121450000	109930000	119670000	119960000	139310000	99005000
sp|O4392	NDUFS5	sp|O4392	1	3	3	3	29	12.517	221000000	176550000	231580000	174350000	168220000	0
sp|O4392	ORC4	sp|O4392	2	4	4	4	12	50.377	102710000	145790000	102820000	162160000	0	104850000
sp|O6021	RAD21	sp|O6021	1	9	9	9	26	71.689	302490000	370790000	295330000	282580000	335860000	348150000
sp|O6022	TIMM8A	sp|O6022	1	4	4	4	68	10.998	547440000	436910000	625600000	498430000	728310000	636110000
sp|O6023	DHX16	sp|O6023	1	10	9	9	15	119.26	237720000	309850000	332830000	293480000	324530000	304820000
sp|O6023	SSSCA1	sp|O6023	1	5	5	5	46	21.474	616300000	489420000	711840000	695430000	704660000	737610000
sp|O6025	PRPSAP2	sp|O6025	1	5	4	4	20	40.925	2021900000	2592900000	2496200000	0	1749000000	1492900000
sp|O6026	SMARCA	sp|O6026	1	32	32	22	33	121.9	2403500000	2219300000	2291900000	2162900000	2360600000	2605800000
sp|O6027	SPAG9	sp|O6027	1	11	11	10	14	146.2	600630000	528060000	533480000	603350000	676480000	524210000
sp|O6028	URB1	sp|O6028	1	6	6	6	4.3	254.39	0	183190000	145130000	232920000	214200000	171650000
sp|O6029	LCMT2	sp|O6029	1	1	1	1	1.6	75.601	0	0	0	0	0	42702000
sp|O6030	AQR	sp|O6030	1	13	13	13	13	171.29	1440100000	987300000	1393200000	1528200000	1331600000	1504400000
sp|O6031	OPA1	sp|O6031	1	9	9	9	11	111.63	506000000	425790000	406050000	335220000	426820000	409370000
sp|O6031	MCM3AP	sp|O6031	1	1	1	1	0.9	218.4	0	0	0	0	26521000	0
sp|O6034	KDM1A	sp|O6034	1	8	8	8	16	92.902	438640000	419910000	316910000	286380000	385310000	416790000
sp|O6034	TBC1D4	sp|O6034	1	6	6	5	6.1	146.56	0	207980000	115580000	98624000	123520000	127900000
sp|O6036	NME2P1	sp|O6036	1	9	1	1	66	15.529	0	0	0	0	0	43119000
sp|O6042	FADS1	sp|O6042	1	4	4	4	16	51.964	158740000	271710000	204590000	133190000	0	128080000
sp|O6043	PPL	sp|O6043	1	16	16	16	15	204.74	698890000	883020000	619000000	454050000	420450000	398110000
sp|O6044	GSDME	sp|O6044	1	2	2	2	9.7	54.554	0	0	0	0	17816000	0
sp|O6048	ACSL4	sp|O6048	1	17	15	15	37	79.187	1453400000	1209200000	1256300000	1716800000	1476800000	1535700000
sp|O6049	SNX3	sp|O6049	1	9	9	8	66	18.762	1429800000	1406400000	1392000000	1192000000	1271200000	1165600000
sp|O6049	STX10	sp|O6049	1	3	3	3	19	28.114	0	0	52906000	0	0	0

sp|O6050	MGEA5	sp|O6050	1	12	12	12	23	102.91	449460000	541890000	419600000	482040000	497920000	603360000
sp|O6050	SORBS3	sp|O6050	1	2	2	2	4.5	75.34	0	0	0	0	0	56334000
sp|O6050	SYNCRIP	sp|O6050	1	26	20	20	50	69.602	1.076E+10	9809800000	1.0385E+10	10351000000	10281000000	9542100000
sp|O6050	CDC40	sp|O6050	1	6	6	6	20	65.521	148880000	163920000	0	168240000	141020000	111450000
sp|O6051	RANBP6	sp|O6051	1	11	9	9	14	124.71	174220000	302620000	316090000	247090000	207860000	220310000
sp|O6052	NEMF	sp|O6052	1	1	1	1	1.3	122.95	0	0	0	0	0	0
sp|O6054	GMDS	sp|O6054	1	3	3	3	9.4	41.949	163420000	142240000	165310000	167260000	138700000	165540000
sp|O6056	CCNT1	sp|O6056	1	3	3	3	7.6	80.684	0	0	290690000	0	0	0
sp|O6056	BUB1B	sp|O6056	1	8	8	8	11	119.54	208990000	142910000	157630000	112720000	119800000	181570000
sp|O6056	PLOD3	sp|O6056	1	15	15	15	25	84.784	1441800000	1482400000	1508000000	1615200000	1642600000	1888100000
sp|O6057	EIF4E2	sp|O6057	1	1	1	1	6.5	28.362	0	0	0	0	0	33689000
sp|O6061	DIAPH1	sp|O6061	1	25	25	25	23	141.35	2259700000	2549000000	2420800000	2585900000	2150100000	2359300000
sp|O6061	SELENOF	sp|O6061	1	2	2	2	18	18.092	0	157750000	0	0	0	0
sp|O6064	EXOC3	sp|O6064	1	2	2	2	4.4	86.844	111000000	74041000	96934000	100990000	85383000	69222000
sp|O6066	PLIN3	sp|O6066	1	17	17	17	60	47.074	3812100000	4251900000	3759100000	3508100000	3846600000	4140900000
sp|O6066	SLC16A7	sp|O6066	1	1	1	1	2.3	52.2	0	0	0	0	49001000	0
sp|O6067	RAD1	sp|O6067	1	3	3	3	18	31.827	40935000	104490000	60904000	127250000	0	80117000
sp|O6067	PRMT3	sp|O6067	1	7	7	7	16	59.902	290930000	228470000	0	0	0	383780000
sp|O6068	KPNA6	sp|O6068	2	9	9	6	19	60.029	1136700000	1245800000	1089100000	1065400000	1132400000	1216500000
sp|O6070	UGDH	sp|O6070	1	26	26	26	71	55.023	7067900000	7299300000	6838600000	8385000000	8157500000	8728900000
sp|O6071	CTNND1	sp|O6071	1	23	23	23	36	108.17	1955500000	1784000000	1677100000	1572200000	1830300000	1855400000
sp|O6073	EIF1B	sp|O6073	1	5	1	1	65	12.823	0	0	0	0	95568000	0
sp|O6074	SNX2	sp|O6074	1	10	10	8	21	58.47	814140000	672020000	929280000	723700000	722590000	778840000
sp|O6076	DPM1	sp|O6076	1	6	6	6	25	29.634	502020000	520820000	417710000	550130000	719640000	628840000
sp|O6076	USO1	sp|O6076	1	20	20	20	29	107.89	1596400000	1421800000	1789600000	1650000000	1832600000	1615900000
sp|O6078	MRPS14	sp|O6078	1	2	2	2	22	15.139	127170000	0	68084000	100830000	162580000	0
sp|O6078	TOM1	sp|O6078	1	5	5	5	21	53.818	158180000	0	194450000	151910000	180700000	0
sp|Q9988	HIST1H2E	sp|Q9988	10	6	2	2	41	13.952	2.2808E+10	2.4119E+10	2.3629E+10	24370000000	30931000000	3.2391E+10
sp|O6082	CCDC22	sp|O6082	1	8	8	8	23	70.755	365040000	427880000	313800000	299280000	316840000	296710000
sp|O6082	PQBP1	sp|O6082	1	6	6	6	41	30.472	319940000	311990000	410400000	359810000	316510000	299490000
sp|O6083	TIMM17B	sp|O6083	1	1	1	1	8.7	18.273	0	0	0	0	50857000	0
sp|O6083	PRAF2	sp|O6083	1	3	3	3	20	19.258	168190000	361980000	276320000	428270000	219460000	276000000
sp|O6083	DKC1	sp|O6083	1	19	19	19	43	57.673	2046800000	2095800000	2020800000	1889800000	2033800000	2138600000
sp|O6084	EIF5B	sp|O6084	1	25	25	25	27	138.83	3978000000	3672700000	4040800000	3693700000	3623900000	3717200000
sp|O6086	EDF1	sp|O6086	1	7	7	7	49	16.368	3103600000	3768200000	3657700000	3461500000	3794600000	3065400000
sp|O6088	DNAJA2	sp|O6088	1	11	11	11	40	45.745	1281600000	1416900000	1349600000	1442500000	1249100000	1279800000
sp|O6088	BRD4	sp|O6088	1	3	3	3	3	152.22	0	0	0	0	116810000	0
sp|O6088	CUTA	sp|O6088	1	3	3	3	33	19.116	385820000	477950000	513960000	720780000	549090000	0
sp|O6092	HUS1	sp|O6092	1	1	1	1	7.1	31.691	0	0	0	0	0	37492000

sp|O6092	PFDN1	sp|O6092	1	7	7	7	62	14.21	826050000	743170000	594880000	707500000	659280000	719000000
sp|O6093	NBN	sp|O6093	1	3	3	3	6.1	84.958	0	0	0	0	95769000	0
sp|O6093	NOL3	sp|O6093	1	4	4	4	36	22.629	318310000	371860000	281030000	354110000	316160000	250790000
sp|O6094	RNGTT	sp|O6094	1	2	2	2	4.4	68.556	0	0	0	0	56502000	0
sp|O7502	ABCB7	sp|O7502	1	6	6	6	9.8	82.64	342160000	440020000	436490000	427100000	375750000	489420000
sp|O7508	WDR1	sp|O7508	1	31	31	31	65	66.193	1.4511E+10	1.4237E+10	1.4634E+10	13875000000	14240000000	1.4432E+10
sp|O7511	ROCK2	sp|O7511	1	21	21	17	20	160.9	1245300000	909400000	1133600000	1079400000	915010000	994740000
sp|O7512	CLASP2	sp|O7512	1	11	11	9	12	141.13	847540000	721610000	767840000	714820000	810630000	754550000
sp|O7513	CPNE3	sp|O7513	5	10	10	9	27	60.13	1248500000	1451400000	1163400000	1480200000	1570300000	1310400000
sp|O7514	HIP1R	sp|O7514	1	1	1	1	1	119.39	0	0	0	0	0	26301000
sp|O7515	RNF40	sp|O7515	1	7	5	5	8.5	113.65	204070000	165340000	0	0	0	0
sp|O7515	ZC3H11A	sp|O7515	2	5	5	5	11	89.13	235720000	333510000	281920000	239090000	165490000	312620000
sp|O7515	CLUH	sp|O7515	1	21	21	21	26	146.67	1263400000	1265000000	1276600000	1224100000	1350900000	1272600000
sp|O7516	DNAJC13	sp|O7516	1	9	9	9	7	254.41	237660000	222750000	269860000	175830000	224660000	261080000
sp|O7517	CNOT3	sp|O7517	1	3	3	3	9	81.871	86005000	90089000	72442000	91121000	87962000	79347000
sp|O7517	ANKRD17	sp|O7517	1	10	6	6	6.7	274.25	0	84994000	129830000	69487000	144090000	0
sp|O7519	DNAJB6	sp|O7519	2	2	2	2	7.4	36.087	0	0	0	0	0	73895000
sp|O7520	COQ9	sp|O7520	1	3	3	3	13	35.509	0	0	0	0	0	179510000
sp|O7522	GGCT	sp|O7522	1	6	6	6	37	21.007	1064300000	564550000	790030000	895290000	1108800000	934190000
sp|O7530	NDUFS2	sp|O7530	1	10	10	10	32	52.545	568280000	644860000	530730000	475510000	505800000	486690000
sp|O7531	ZPR1	sp|O7531	1	4	4	4	15	50.925	157920000	201750000	175140000	153430000	174460000	0
sp|O7533	HMMR	sp|O7533	1	4	4	4	8.1	84.099	153400000	122700000	0	198840000	105410000	145670000
sp|O7534	PDCD6	sp|O7534	1	7	7	7	56	21.868	1604300000	1369000000	1814100000	1921600000	1787600000	1695400000
sp|O7534	TBCA	sp|O7534	1	8	8	8	58	12.855	4275800000	4813400000	4029600000	3332900000	3625500000	4203400000
sp|O7534	ATP6V1G	sp|O7534	2	3	3	3	34	13.757	404100000	385390000	387500000	470370000	505920000	303460000
sp|O7535	VPS4B	sp|O7535	2	8	8	7	28	49.301	354530000	418740000	460750000	364070000	432410000	360040000
sp|O7535	MPDU1	sp|O7535	1	1	1	1	4	26.638	0	0	0	0	0	244170000
sp|O7536	H2AFY	sp|O7536	2	15	15	15	47	39.617	6625500000	7914600000	5783400000	7643800000	7294000000	8029700000
sp|O7536	SH3BGRL	sp|O7536	1	8	8	8	79	12.774	1473200000	1784200000	1430200000	1215500000	1412000000	1222900000
sp|O7536	FLNB	sp|O7536	1	##	144	##	76	278.16	9.9145E+10	9.6166E+10	9.5536E+10	94756000000	92449000000	1.0528E+11
sp|O7537	NCOR1	sp|O7537	1	4	4	4	2.7	270.21	25315000	56189000	0	26622000	77675000	0
sp|O7538	PEX14	sp|O7538	1	2	2	2	5.6	41.236	153030000	128870000	141730000	124590000	0	0
sp|O7539	CS	sp|O7539	1	12	12	12	29	51.712	5950600000	4801800000	4563000000	5943800000	6447500000	5281000000
sp|O7539	SPAG7	sp|O7539	1	6	6	6	41	26.034	401040000	508880000	461530000	302600000	326410000	307130000
sp|O7539	SEC22B	sp|O7539	1	7	7	7	38	24.593	4389000000	3483600000	3757100000	4551400000	4328900000	4518500000
sp|O7540	PRPF40A	sp|O7540	1	6	6	6	7.5	108.8	431370000	465340000	388950000	462670000	354970000	404830000
sp|O7541	TACC1	sp|O7541	2	3	3	3	6.2	87.793	0	0	0	0	0	27395000
sp|O7541	NME6	sp|O7541	1	1	1	1	7.5	21.142	0	0	0	0	0	79443000
sp|O7543	MTX2	sp|O7543	1	5	5	5	31	29.763	356940000	339210000	392130000	418930000	480960000	466130000

sp|O7543(	VPS26A	sp|O7543(	1	7	7	7	31	38.169	1289800000	1127500000	1529500000	1338700000	1405000000	1245700000
sp|O7543(	NDUFB1	sp|O7543(	1	1	1	1	19	6.9611	0	0	0	0	0	91808000
sp|O7543(	PMPCB	sp|O7543(	1	18	18	17	44	54.366	1714700000	1504100000	1449900000	1522100000	1631900000	1620500000
sp|O7544(	MED24	sp|O7544(	1	3	3	3	4.6	110.3	30055000	0	0	0	0	0
sp|O7547(	PSIP1	sp|O7547(	1	13	12	12	28	60.103	2297100000	2659500000	2337400000	2520300000	2206800000	2421100000
sp|O7547	ERLIN1	sp|O7547	1	5	3	3	19	38.925	0	265440000	234220000	424440000	304330000	265790000
sp|O7548(	NDUFS3	sp|O7548(	1	7	7	7	31	30.241	744430000	755260000	903630000	722630000	701430000	646930000
sp|O7549(	SRSF10	sp|O7549(	1	4	4	4	13	31.3	601150000	506500000	617500000	608440000	488550000	637870000
sp|O7550(	HSBP1	sp|O7550(	1	3	3	3	42	8.5435	535310000	540420000	475730000	482670000	0	421260000
sp|O7552	ECI2	sp|O7552	1	11	11	11	43	43.585	682880000	764490000	771240000	821860000	861600000	671960000
sp|O7553(	EED	sp|O7553(	1	3	3	3	8.6	50.197	0	0	115450000	0	0	0
sp|O7553	BANF1	sp|O7553	1	6	6	6	53	10.058	5219800000	5912900000	4745600000	5439300000	7658500000	6221600000
sp|O7553(	SF3B1	sp|O7553(	1	40	40	40	42	145.83	6661000000	7548200000	7104700000	6543800000	6730400000	7160400000
sp|O7553(	CSDE1	sp|O7553(	1	24	24	24	33	88.884	2434300000	2387400000	2239500000	2183900000	2118900000	2365800000
sp|O7556(	PRKRA	sp|O7556(	1	4	4	4	20	34.404	149040000	202850000	150820000	244390000	208950000	244570000
sp|O7560	NPM3	sp|O7560	1	5	5	5	54	19.343	497220000	668130000	473740000	697620000	644750000	547220000
sp|O7560(	LYPLA1	sp|O7560(	1	2	2	2	16	24.669	0	192850000	105310000	253980000	0	0
sp|O7561(	ERAL1	sp|O7561(	1	4	4	4	15	48.349	225840000	0	211490000	255650000	0	0
sp|O7564(	SNRNP20	sp|O7564(	1	68	68	68	39	244.5	8356700000	8145000000	8602800000	7749400000	8142700000	8209600000
sp|O7566(	TIPRL	sp|O7566(	1	7	7	7	38	31.444	440070000	409720000	453070000	456920000	585710000	349230000
sp|O7568(	SURF6	sp|O7568(	1	2	2	2	12	41.45	0	0	0	0	0	183260000
sp|O7568(	PPM1B	sp|O7568(	2	2	2	2	5.8	52.642	65041000	0	0	52917000	0	0
sp|O7569	UTP20	sp|O7569	1	18	18	18	7.6	318.38	543140000	790600000	622170000	652770000	559440000	536920000
sp|O7569(	NUP155	sp|O7569(	1	31	31	31	32	155.2	4571900000	5189300000	4382900000	5101400000	5172000000	5066700000
sp|O7569(	RP2	sp|O7569(	1	2	2	2	5.4	39.641	0	0	0	0	0	61006000
sp|O7571	WDHD1	sp|O7571	1	9	9	9	11	125.97	227730000	267260000	269030000	258610000	217110000	218030000
sp|O7571(	CRTAP	sp|O7571(	1	13	13	13	34	46.561	957560000	972630000	1050800000	1096200000	1282600000	1037400000
sp|O7574(	SLC25A12	sp|O7574(	1	13	10	10	22	74.761	388360000	329940000	302210000	353310000	278620000	252190000
sp|O7578	ATP6AP2	sp|O7578	1	3	3	3	10	39.008	0	55944000	0	0	60302000	0
sp|O7579(	RNASEH2	sp|O7579(	1	3	3	3	13	33.395	0	0	0	0	77953000	0
sp|O7579(	CDC123	sp|O7579(	1	9	9	9	30	39.134	552370000	351470000	551780000	342690000	567130000	474750000
sp|O7581(	BCAR3	sp|O7581(	1	2	2	2	4.6	92.565	0	0	55427000	52130000	0	0
sp|O7581(	RPP40	sp|O7581(	1	6	6	6	26	41.833	260910000	284840000	282090000	312690000	224220000	313720000
sp|O7582	EIF3G	sp|O7582	1	13	13	13	41	35.611	2946600000	3600800000	3097100000	3438300000	3271300000	2596900000
sp|O7582(	EIF3J	sp|O7582(	1	12	12	12	52	29.062	3476600000	3357700000	2795100000	3763900000	3573700000	3079000000
sp|O7582(	CBR3	sp|O7582(	1	5	3	3	24	30.85	257710000	213000000	242040000	239670000	298320000	310650000
sp|O7583(	PSMD10	sp|O7583(	1	3	3	3	15	24.428	663530000	548180000	668920000	506690000	609560000	553130000
sp|O7584(	ZMPSTE2	sp|O7584(	1	8	8	8	21	54.812	760700000	774550000	829890000	810880000	746730000	843460000
sp|O7587(	IDH1	sp|O7587(	1	18	18	18	48	46.659	3824100000	4284700000	4949300000	4318300000	3567000000	3302500000

sp|O7588	STAM2	sp|O7588	1	1	1	1	2.7	58.164	0	0	0	0	89061000	0
sp|O7590	DGAT1	sp|O7590	1	1	1	1	3.3	55.278	0	0	0	0	0	38241000
sp|O7590	CCNK	sp|O7590	1	3	3	3	5.3	64.239	0	0	0	0	73204000	0
sp|O7591	ARL6IP5	sp|O7591	1	5	5	5	25	21.614	1130000000	1523500000	1436900000	1540900000	1281100000	1608400000
sp|O7593	BCAS2	sp|O7593	1	11	11	11	69	26.131	923520000	1309100000	953180000	979210000	1132200000	958130000
sp|O7593	DCTN3	sp|O7593	1	6	6	6	23	21.119	684800000	844730000	537670000	607880000	633430000	613380000
sp|O7593	DNAJC8	sp|O7593	1	8	8	8	36	29.841	1824700000	2166100000	1591500000	1270900000	1159600000	1260500000
sp|O7594	SMNDC1	sp|O7594	1	5	5	5	38	26.711	434020000	420150000	404280000	485300000	438510000	449920000
sp|O7594	ATP5H	sp|O7594	1	11	11	11	73	18.491	2454300000	3073600000	3048300000	3359500000	3813800000	4948900000
sp|O7595	FLOT1	sp|O7595	1	10	10	10	33	47.355	803280000	895710000	1087500000	812230000	1053900000	1116500000
sp|O7596	TRIO	sp|O7596	2	22	22	22	10	346.9	480260000	472310000	491730000	449780000	517580000	472210000
sp|O7596	ATP5L	sp|O7596	1	4	4	4	38	11.428	1991900000	1217300000	1144400000	1242500000	1745200000	1031300000
sp|O7600	GLRX3	sp|O7600	1	11	11	11	46	37.432	2769100000	2169000000	3196300000	2211400000	2676200000	2732600000
sp|O7602	RSL1D1	sp|O7602	1	16	16	16	32	54.972	3799700000	4112100000	4468400000	4337500000	4072700000	4623700000
sp|O7603	CLPX	sp|O7603	1	11	11	11	23	69.223	614840000	453080000	437800000	798340000	386560000	653980000
sp|O7607	SNCG	sp|O7607	1	8	8	8	72	13.331	922140000	762880000	907650000	721590000	1005500000	887910000
sp|O7607	CIAO1	sp|O7607	1	6	6	6	27	37.84	288510000	274080000	331680000	277800000	307130000	313010000
sp|O7609	SRP72	sp|O7609	1	20	20	20	41	74.605	2260600000	2185000000	1968900000	1989300000	2475900000	1795400000
sp|O9476	DDAH1	sp|O9476	1	8	8	8	39	31.121	398940000	381450000	428270000	403000000	407880000	382690000
sp|O9477	MTA2	sp|O9477	1	17	17	15	34	75.022	1779600000	2063400000	1920400000	1923000000	2010000000	2020200000
sp|O9478	ALDH1A2	sp|O9478	1	3	2	2	7.3	56.723	0	142900000	88696000	142510000	148030000	153960000
sp|O9480	STK10	sp|O9480	1	3	3	3	3.6	112.13	0	0	0	0	21789000	0
sp|O9480	GFPT2	sp|O9480	1	9	6	6	14	76.93	1597700000	730750000	1371800000	1472600000	1463000000	1827700000
sp|O9482	LTN1	sp|O9482	1	3	3	3	2.8	200.55	89197000	75110000	48758000	75340000	0	64509000
sp|O9482	TOMM70	sp|O9482	1	19	19	19	49	67.454	2074600000	1953100000	2063900000	2484500000	2324800000	2478500000
sp|O9484	TOX4	sp|O9484	3	2	2	2	4.3	66.194	37300000	0	0	0	0	0
sp|O9485	SEC24D	sp|O9485	1	10	10	10	16	113.01	539770000	606100000	586410000	530360000	600630000	577440000
sp|O9487	UFL1	sp|O9487	1	9	9	9	15	89.594	127170000	146260000	171250000	266790000	225130000	196290000
sp|O9488	FARP2	sp|O9488	1	2	2	2	2.7	119.89	0	213890000	160110000	0	0	0
sp|O9488	UBXN7	sp|O9488	1	5	5	5	12	54.862	117100000	135380000	107120000	129220000	121420000	98874000
sp|O9490	SUN1	sp|O9490	1	7	7	7	11	90.063	0	184660000	191970000	328200000	0	137500000
sp|O9490	PLPBP	sp|O9490	1	1	1	1	6.2	30.344	0	0	0	0	0	32230000
sp|O9490	ERLIN2	sp|O9490	1	7	7	5	27	37.839	669160000	759260000	0	683650000	692500000	741030000
sp|O9490	PRPF6	sp|O9490	1	26	26	26	34	106.92	2717000000	2221700000	2174900000	2252400000	2514900000	3017100000
sp|O9491	FRYL	sp|O9491	1	3	3	3	1.6	339.59	438490000	0	0	70806000	0	0
sp|O9491	ENDOD1	sp|O9491	1	1	1	1	2.6	55.016	0	0	0	0	0	0
sp|O9492	GLS	sp|O9492	1	19	19	19	38	73.46	5859900000	4821200000	6267100000	5649500000	5691100000	6147100000
sp|O9496	USP19	sp|O9496	1	3	3	3	4.2	145.65	0	0	0	0	0	182870000
sp|O9497	AP2A2	sp|O9497	1	13	6	6	20	103.96	152230000	105520000	159390000	126670000	117310000	117930000

sp|O9497	SEC31A	sp|O9497	1	25	25	25	25	133.01	2586300000	2897900000	2446500000	2472400000	2413500000	2574000000
sp|O9499	HEXIM1	sp|O9499	1	7	7	7	37	40.623	448280000	591030000	443460000	426380000	600680000	438620000
sp|O9505	RPP14	sp|O9505	1	3	3	3	38	13.693	0	0	0	152520000	0	0
sp|O9506	CCNB2	sp|O9506	1	3	3	3	10	45.281	0	0	0	0	45630000	0
sp|O9507	YIF1A	sp|O9507	1	3	3	3	13	32.011	405110000	500720000	372820000	494500000	416970000	462280000
sp|O9507	UBR5	sp|O9507	1	8	8	8	4.7	309.35	85228000	139670000	101290000	162240000	126530000	119030000
sp|O9513	NDUFB6	sp|O9513	1	3	3	3	30	15.489	575820000	518130000	510770000	271960000	473710000	495530000
sp|O9514	MFN2	sp|O9514	1	8	8	8	14	86.401	238210000	123640000	292850000	260500000	153050000	192570000
sp|O9514	DUSP14	sp|O9514	1	1	1	1	11	22.255	0	0	0	0	0	16567000
sp|O9514	SNUPN	sp|O9514	1	2	2	2	10	41.142	0	0	0	0	79421000	0
sp|O9515	UBE4B	sp|O9515	1	15	15	15	19	146.18	681380000	532900000	723270000	560670000	499420000	682860000
sp|O9515	ZFPL1	sp|O9515	1	3	3	3	14	34.114	59029000	0	65046000	0	159840000	0
sp|O9516	ELP1	sp|O9516	1	14	14	14	15	150.25	1178200000	823350000	1284900000	915990000	1003100000	1013400000
sp|O9516	GABARAF	sp|O9516	1	2	2	1	15	13.918	0	0	0	0	0	234760000
sp|O9516	NDUFB4	sp|O9516	1	3	3	3	31	15.208	171680000	157020000	211990000	160090000	261010000	0
sp|O9516	NDUFB8	sp|O9516	1	2	2	2	11	21.766	0	0	0	0	0	69943000
sp|O9519	RTN3	sp|O9519	1	2	2	2	4.8	112.61	363400000	603200000	432990000	428280000	435960000	438490000
sp|O9520	LETM1	sp|O9520	1	19	19	19	33	83.353	1652700000	1499200000	2015000000	1994000000	1853400000	1887300000
sp|O9521	ZRANB2	sp|O9521	1	11	11	11	31	37.404	1016200000	1271800000	870810000	959520000	1083000000	1075800000
sp|O9521	SNX4	sp|O9521	1	2	2	2	5.1	51.908	0	0	0	66230000	0	0
sp|O9523	LUC7L3	sp|O9523	1	12	12	12	30	51.466	1246100000	1413500000	1354400000	967940000	1135600000	1458000000
sp|O9523	KIF20A	sp|O9523	1	12	12	12	21	100.28	315360000	246330000	209810000	322040000	220440000	253940000
sp|O9523	KIF4A	sp|O9523	3	11	11	5	11	139.88	616310000	390720000	494250000	670820000	631540000	356090000
sp|O9524	SBF1	sp|O9524	1	2	2	2	0.9	208.44	0	0	0	0	0	118090000
sp|O9525	KAT7	sp|O9525	3	4	4	4	10	70.642	176960000	0	0	223540000	191760000	0
sp|O9526	ATE1	sp|O9526	1	2	2	2	8.3	59.09	0	0	0	131510000	0	141000000
sp|O9527	CCNDBP1	sp|O9527	1	1	1	1	3.6	40.262	0	0	29156000	0	0	0
sp|O9529	VAPB	sp|O9529	1	10	9	9	43	27.228	1254100000	1379200000	1070300000	1302800000	1435500000	1237700000
sp|O9529	SNAPIN	sp|O9529	1	2	2	2	18	14.874	115890000	0	139270000	98012000	118820000	104340000
sp|O9529	MPZL1	sp|O9529	1	3	3	3	20	29.082	271950000	222650000	0	202010000	245880000	270100000
sp|O9529	NDUFA10	sp|O9529	1	3	3	3	14	40.75	0	0	294610000	0	0	248980000
sp|O9530	FKBP9	sp|O9530	2	9	9	9	18	63.083	2649800000	3219000000	2960900000	3011700000	2384500000	3002700000
sp|O9533	PGLS	sp|O9533	1	9	9	9	52	27.547	913280000	777450000	797780000	892680000	931530000	462020000
sp|O9534	PAPSS2	sp|O9534	1	9	9	9	17	69.5	766000000	675420000	541060000	709520000	717920000	721040000
sp|O9534	SMC2	sp|O9534	1	27	27	27	28	135.65	2528300000	2698900000	2723600000	2554600000	2530500000	2448500000
sp|O9535	ATG7	sp|O9535	1	5	5	5	12	77.959	83663000	0	182290000	139770000	0	86671000
sp|O9536	TRIM16	sp|O9536	2	18	18	18	42	63.954	1750700000	1705300000	1609900000	1948900000	1978500000	1796000000
sp|O9536	ZBTB7A	sp|O9536	1	2	2	2	7.5	61.438	44500000	0	0	0	0	0
sp|O9537	LYPLA2	sp|O9537	1	6	6	6	33	24.737	359170000	808900000	599900000	467450000	467260000	499170000

sp|O9537	IPO7	sp|O9537	1	24	24	24	28	119.52	5609200000	7162100000	6413600000	6949400000	6321600000	5801500000
sp|O9537	ARIH2	sp|O9537	1	5	5	5	15	57.818	0	163820000	247360000	206700000	202340000	199490000
sp|O9537	TNFAIP8	sp|O9537	1	2	2	2	14	23.003	99055000	466400000	42065000	795720000	189910000	260760000
sp|O9539	SLU7	sp|O9539	1	1	1	1	4.6	68.386	0	0	0	0	0	26198000
sp|O9539	PGM3	sp|O9539	1	3	3	3	6.6	59.851	106350000	111970000	53469000	80373000	169750000	200270000
sp|O9539	MOCS3	sp|O9539	1	2	2	2	6.7	49.669	0	0	0	0	0	80633000
sp|O9539	UTS2	sp|O9539	1	1	1	1	6.5	14.295	0	0	0	0	0	2114100000
sp|O9540	CD2BP2	sp|O9540	1	4	4	4	14	37.646	0	228190000	0	217610000	250230000	231880000
sp|O9540	CNIH1	sp|O9540	1	2	2	2	20	16.699	459010000	244220000	369180000	547200000	475430000	440750000
sp|O9541	BRI3	sp|O9541	1	1	1	1	13	13.645	0	0	0	32334000	0	0
sp|O9542	SVIL	sp|O9542	1	6	6	6	4.8	247.74	187910000	248050000	192200000	0	214390000	209180000
sp|O9542	BAG4	sp|O9542	1	1	1	1	3.1	49.593	0	0	0	0	70269000	0
sp|O9543	AHSA1	sp|O9543	1	14	14	14	53	38.274	6350400000	6184800000	4685300000	6305900000	6249900000	5782000000
sp|O9545	PARN	sp|O9545	1	2	2	2	3.8	73.45	0	0	0	0	0	51308000
sp|O9545	PSMG1	sp|O9545	1	9	9	9	42	32.854	620420000	670530000	706200000	568590000	673120000	634480000
sp|O9546	FMNL1	sp|O9546	1	3	3	3	3.9	121.85	92301000	63721000	0	59681000	70243000	74231000
sp|O9547	SGPL1	sp|O9547	1	4	4	4	7.6	63.523	0	80861000	89549000	182870000	0	0
sp|O9547	NSA2	sp|O9547	1	4	4	4	16	30.065	356950000	301490000	0	240210000	307420000	184420000
sp|O9548	SEC24A	sp|O9548	1	3	3	3	3.3	119.75	52121000	57862000	0	57253000	0	48417000
sp|O9548	SEC24B	sp|O9548	1	2	2	2	2.3	137.42	115610000	0	0	0	87135000	0
sp|O9554	NADK	sp|O9554	1	1	1	1	3.8	49.228	0	0	0	0	0	0
sp|O9556	MPC2	sp|O9556	1	2	2	2	17	14.279	85382000	0	93165000	89274000	0	100200000
sp|O9557	ETHE1	sp|O9557	1	2	2	2	17	27.873	0	0	0	0	0	137130000
sp|O9557	ACSL3	sp|O9557	1	21	21	19	43	80.419	3250600000	2864200000	3057600000	3377700000	3174200000	3722500000
sp|O9560	POLR1A	sp|O9560	1	4	4	4	4	194.81	113260000	82121000	90894000	82843000	133240000	97690000
sp|O9563	CPSF4	sp|O9563	1	3	3	3	13	30.255	97226000	0	0	0	0	0
sp|O9567	CDS2	sp|O9567	1	1	1	1	4.3	51.417	0	0	0	0	38571000	0
sp|O9567	EYA4	sp|O9567	1	2	2	2	4.7	69.504	0	0	0	0	0	45973000
sp|O9568	FGFR1OP	sp|O9568	1	2	2	2	7.8	43.064	0	64633000	87025000	69670000	0	78865000
sp|O9574	OXSR1	sp|O9574	1	14	14	12	34	58.022	1649100000	1803100000	1556800000	1275500000	1661100000	1878600000
sp|O9575	HSPA4L	sp|O9575	1	29	25	25	49	94.511	2246400000	2717300000	2947100000	2651500000	2850000000	2787200000
sp|O9575	PTBP3	sp|O9575	1	7	5	5	19	59.689	287510000	269850000	275590000	336170000	328600000	276330000
sp|O9577	LSM8	sp|O9577	1	3	3	3	38	10.403	350200000	427110000	359930000	357090000	421840000	357800000
sp|O9578	AP2A1	sp|O9578	1	25	25	18	35	107.54	1876200000	1930800000	2059400000	1720400000	1682800000	1802800000
sp|O9578	DDX58	sp|O9578	1	5	5	5	5.2	106.6	850930000	476790000	0	480240000	640960000	0
sp|O9580	TTC4	sp|O9580	1	2	2	2	13	44.678	0	1040400000	0	0	0	0
sp|O9580	TMEM50A	sp|O9580	1	1	1	1	5.1	17.4	74624000	69792000	0	0	94075000	71751000
sp|O9581	BAG2	sp|O9581	1	12	12	12	63	23.772	3793600000	3965300000	3564000000	4174200000	4343800000	3847700000
sp|O9581	BAG3	sp|O9581	1	18	18	18	56	61.594	2054700000	2485100000	1749200000	2621100000	1917300000	2601600000

sp|O9581!	MAP4K4	sp|O9581!	2	3	3	2	3.3	142.1	0	0	0	0	78210000	0
sp|O9582!	CRYZL1	sp|O9582!	1	1	1	1	8	38.696	0	0	0	0	0	11832000
sp|O9583	AIFM1	sp|O9583	1	12	12	12	25	66.9	3003700000	2954500000	2597400000	2594300000	2599000000	2732200000
sp|O9583	CLDN1	sp|O9583	1	1	1	1	7.1	22.744	0	0	0	0	0	78376000
sp|O9583	CLIC3	sp|O9583	1	8	8	8	52	26.648	657460000	897430000	537450000	663210000	706280000	827270000
sp|O9586	BPNT1	sp|O9586	1	9	9	9	47	33.392	474250000	516770000	559630000	467240000	513220000	713420000
sp|O9586!	DDAH2	sp|O9586!	1	3	3	3	16	29.644	111190000	0	0	0	75574000	0
sp|O9588	TXNDC12	sp|O9588	1	7	7	7	44	19.206	484810000	573590000	552100000	545210000	666540000	538330000
sp|O9590!	ECD	sp|O9590!	1	3	3	3	9	72.757	76611000	77666000	84294000	57355000	64864000	65359000
sp|O9592	SYF2	sp|O9592	1	1	1	1	12	28.722	0	0	0	0	0	60901000
sp|O9598	MBD3	sp|O9598	1	5	5	5	24	32.844	0	0	254660000	0	0	0
sp|O9599!	BCL10	sp|O9599!	1	2	2	2	20	26.251	0	0	0	0	72650000	0
sp|O9600	NDUFB10	sp|O9600	1	4	4	4	28	20.776	457190000	425630000	481090000	443070000	323920000	534740000
sp|O9600!	CLPTM1	sp|O9600!	1	11	11	11	28	76.096	623860000	669680000	691960000	730830000	647710000	713290000
sp|O9600	MOCS2	sp|O9600	1	2	2	2	13	20.944	0	0	0	64249000	0	0
sp|O9600	TOMM40	sp|O9600	1	8	8	8	39	37.893	2230400000	2298700000	2083300000	2555900000	2505900000	2421500000
sp|O9601!	ACTL6A	sp|O9601!	2	12	12	12	45	47.46	2366400000	2403100000	2070600000	2550600000	2254500000	2674700000
sp|P0016	CYB5A	sp|P0016	1	5	5	5	49	15.33	306760000	440520000	383120000	372740000	384340000	380570000
sp|P0033	LDHA	sp|P0033	3	24	24	23	68	36.688	8.7011E+10	9.4285E+10	8.8105E+10	79974000000	80066000000	8.4824E+10
sp|P0036	GLUD1	sp|P0036	2	19	19	19	41	61.397	4101400000	3463700000	3735000000	4229000000	3945200000	4029600000
sp|P0037	DHFR	sp|P0037	2	7	7	7	48	21.452	1296900000	1380600000	1141100000	1062800000	1380300000	1029500000
sp|P0038	CYB5R3	sp|P0038	1	13	13	13	64	34.234	4511400000	4355900000	4624400000	5353600000	5326000000	4845400000
sp|P0039	GSR	sp|P0039	1	15	15	15	46	56.256	3151200000	3130200000	2760000000	3202700000	3679000000	3018800000
sp|P0039	MT-CO1	sp|P0039	1	1	1	1	6	57.041	0	0	0	0	22734000	0
sp|P0040	MT-CO2	sp|P0040	1	6	6	6	31	25.565	1516400000	1676100000	1432100000	1387900000	1237300000	1169200000
sp|P0044	SOD1	sp|P0044	1	6	6	6	60	15.936	8449700000	9093100000	9566800000	6682500000	6157200000	7979600000
sp|P0049	PNP	sp|P0049	1	16	16	16	74	32.118	4875800000	6149500000	5497700000	4189100000	4389700000	4346300000
sp|P0049	HPRT1	sp|P0049	2	12	12	12	73	24.579	4299800000	3497400000	4410100000	4482600000	3734200000	4070000000
sp|P0050	GOT2	sp|P0050	1	19	19	19	51	47.517	6937800000	7442400000	6770100000	8330400000	8854800000	8281500000
sp|P0053	EGFR	sp|P0053	3	18	18	18	26	134.28	981590000	1066500000	784180000	970120000	1360200000	1031400000
sp|P0055	PGK1	sp|P0055	2	27	27	27	63	44.614	7.5839E+10	7.3002E+10	7.1501E+10	67051000000	70987000000	6.7909E+10
sp|P0056	AK1	sp|P0056	2	10	10	10	61	21.635	2355500000	2019400000	3181200000	2203500000	2171100000	2679100000
sp|P0084	MT-ATP6	sp|P0084	1	1	1	1	4.4	24.817	0	0	0	0	0	315420000
sp|P0091	CA2	sp|P0091	1	1	1	1	3.5	29.246	0	0	0	0	33383000	0
sp|P0096	ASS1	sp|P0096	1	5	5	5	13	46.53	191280000	172850000	190990000	279370000	348960000	290630000
sp|P0103	TIMP1	sp|P0103	1	1	1	1	5.8	23.171	0	0	0	0	0	0
sp|P0103	CST3	sp|P0103	1	2	2	2	19	15.799	31908000	0	0	0	0	0
sp|P0111	NRAS	sp|P0111	3	7	7	7	58	21.229	378280000	672630000	772660000	629350000	529230000	644670000
sp|P0113	LDLR	sp|P0113	1	2	2	2	3.6	95.375	0	74756000	0	157640000	0	0

sp|P01891	HLA-A	sp|P01891	6	17	17	3	49	40.908	6083900000	4956200000	4696500000	5577700000	6126700000	5445600000
sp|P02462	COL4A1	sp|P02462	2	2	2	2	1.7	160.61	201690000	137970000	106420000	170750000	202540000	131220000
sp|P02545	LMNA	sp|P02545	2	46	46	44	70	74.139	6.6415E+10	6.2948E+10	6.0403E+10	64075000000	67751000000	7.1118E+10
sp|P02656	APOC3	sp|P02656	1	1	1	1	16	10.852	0	0	233480000	0	0	0
sp|P02751	FN1	sp|P02751	1	14	14	14	9.3	262.62	149700000	122640000	121420000	1028200000	616750000	1273600000
sp|P02786	TFRC	sp|P02786	2	28	28	28	41	84.87	8666200000	8874300000	8654300000	8176600000	7942400000	9217400000
sp|P02792	FTL	sp|P02792	1	3	3	3	23	20.019	196160000	154390000	0	176400000	0	0
sp|P02794	FTH1	sp|P02794	1	11	11	11	72	21.225	4430700000	4531100000	4347000000	3819400000	4038600000	3352900000
sp|P03897	MT-ND3	sp|P03897	1	1	1	1	13	13.186	0	0	0	0	0	79692000
sp|P03905	MT-ND4	sp|P03905	1	1	1	1	2.2	51.58	0	0	0	0	0	21608000
sp|P03915	MT-ND5	sp|P03915	1	2	2	2	6	67.026	97303000	85123000	123000000	0	0	98452000
sp|P03928	MT-ATP8	sp|P03928	1	3	3	3	49	7.9916	472750000	462290000	413410000	470320000	993160000	494510000
sp|P04040	CAT	sp|P04040	1	13	13	13	35	59.755	1557100000	1273900000	1318400000	1341800000	1239300000	1416800000
sp|P04062	GBA	sp|P04062	1	7	7	7	17	59.716	311760000	394420000	409590000	458700000	453000000	388500000
sp|P04075	ALDOA	sp|P04075	2	28	28	23	84	39.42	8.2045E+10	8.3422E+10	6.796E+10	74423000000	74216000000	7.2752E+10
sp|P04080	CSTB	sp|P04080	1	5	5	5	78	11.139	4344900000	2866600000	3934700000	3780600000	5838700000	4680000000
sp|P04083	ANXA1	sp|P04083	1	24	24	24	71	38.714	3.5307E+10	3.712E+10	3.2867E+10	36753000000	32976000000	3.6082E+10
sp|P04150	NR3C1	sp|P04150	2	11	11	11	21	85.658	482600000	406240000	275760000	439000000	531480000	445350000
sp|P04156	PRNP	sp|P04156	1	2	2	2	9.5	27.661	0	0	0	0	0	92096000
sp|P04179	SOD2	sp|P04179	1	8	8	8	46	24.75	1777200000	1907600000	2086600000	2555200000	2102700000	2291500000
sp|P04181	OAT	sp|P04181	1	10	10	10	38	48.534	883790000	806150000	1053900000	972850000	974120000	700420000
sp|P04183	TK1	sp|P04183	1	4	4	4	20	25.468	346860000	380620000	325050000	427670000	543190000	464510000
sp|P04406	GAPDH	sp|P04406	2	17	17	17	68	36.053	1.4562E+11	1.4331E+11	1.3625E+11	1.3327E+11	1.3604E+11	1.3816E+11
sp|P04632	CAPNS1	sp|P04632	2	10	10	10	59	28.315	2034500000	2670200000	2230300000	2309900000	2151000000	2219700000
sp|P04792	HSPB1	sp|P04792	1	17	17	17	85	22.782	3.9908E+10	3.7397E+10	3.7673E+10	37348000000	37821000000	3.6436E+10
sp|P04818	TYMS	sp|P04818	1	6	6	6	27	35.716	712830000	788210000	745250000	397640000	390870000	404970000
sp|P04843	RPN1	sp|P04843	1	31	31	31	60	68.569	8583200000	9181400000	9200200000	10345000000	9544400000	1.031E+10
sp|P04844	RPN2	sp|P04844	1	20	20	20	45	69.283	6994300000	6746900000	7709200000	7110100000	7015000000	8168800000
sp|P04899	GNAI2	sp|P04899	1	14	14	9	54	40.45	3435200000	3618900000	3420100000	3121300000	4014100000	3597400000
sp|Q99878	HIST1H2A	sp|Q99878	12	3	1	1	27	13.936	1.1255E+10	1.1729E+10	1.14E+10	10297000000	13292000000	8935500000
sp|P05023	ATP1A1	sp|P05023	5	38	38	25	44	112.89	9798700000	8839600000	1.0414E+10	8827100000	9017400000	1.0418E+10
sp|P05026	ATP1B1	sp|P05026	1	7	7	7	32	35.061	768660000	901530000	935280000	841810000	865270000	917390000
sp|P05067	APP	sp|P05067	1	4	4	4	6.5	86.942	0	202210000	0	156070000	181760000	0
sp|P05091	ALDH2	sp|P05091	1	14	13	13	41	56.381	830370000	917430000	838560000	940700000	992930000	1045300000
sp|P05114	HMGN1	sp|P05114	1	4	4	4	34	10.659	641420000	459960000	606420000	521620000	518230000	502280000
sp|P05141	SLC25A5	sp|P05141	2	15	6	6	43	32.852	7620300000	6472000000	6020900000	8422400000	8274100000	7595500000
sp|P05161	ISG15	sp|P05161	1	10	10	10	64	17.887	6811100000	6967200000	5148400000	7061800000	9150900000	8526500000
sp|P05166	PCCB	sp|P05166	1	1	1	1	3	58.215	0	0	0	0	0	29453000
sp|P05198	EIF2S1	sp|P05198	1	21	21	21	67	36.112	6708600000	5762800000	6699700000	6020900000	6135600000	6381600000

sp|P05204	HMGN2	sp|P05204	2	1	1	1	10	9.3926	0	0	0	0	0	4422800
sp|P05362	ICAM1	sp|P05362	1	4	4	4	9.2	57.825	0	0	0	167550000	0	0
sp|P05386	RPLP1	sp|P05386	1	4	3	3	90	11.514	3022900000	2767300000	2875000000	2406000000	2083000000	2292500000
sp|P05387	RPLP2	sp|P05387	1	9	9	8	92	11.665	1.823E+10	2.3252E+10	2.056E+10	24302000000	30008000000	2.0953E+10
sp|P05388	RPLP0	sp|P05388	2	13	13	13	58	34.273	2.3603E+10	2.0347E+10	1.8852E+10	22612000000	24363000000	1.8606E+10
sp|P05423	POLR3D	sp|P05423	1	1	1	1	2.3	44.395	0	0	0	0	16530000	0
sp|P05455	SSB	sp|P05455	1	24	24	24	57	46.836	4474800000	5095300000	3870700000	4700500000	4056100000	4738300000
sp|P05556	ITGB1	sp|P05556	1	23	23	23	33	88.414	9028300000	1.0868E+10	9825100000	10953000000	11857000000	1.0751E+10
sp|P05783	KRT18	sp|P05783	5	29	28	19	69	48.057	4.4078E+10	4.8988E+10	4.5817E+10	49210000000	52913000000	4.4978E+10
sp|P05976	MYL1	sp|P05976	2	2	2	2	8.2	21.145	0	222750000	0	0	599420000	841810000
sp|P06132	UROD	sp|P06132	1	9	9	9	38	40.786	1321100000	986730000	1083100000	1025600000	1215200000	964730000
sp|P06280	GLA	sp|P06280	1	3	3	3	11	48.766	0	0	0	0	121730000	0
sp|P06396	GSN	sp|P06396	1	14	14	5	27	85.696	1410500000	1694800000	1765500000	1789700000	1761700000	1772200000
sp|P06454	PTMA	sp|P06454	1	7	7	7	36	12.203	9956400000	9599700000	1.0074E+10	6798100000	8953600000	7731500000
sp|P06493	CDK1	sp|P06493	1	10	10	9	39	34.095	3808500000	3307500000	3502700000	3240700000	3773600000	3972600000
sp|P06576	ATP5F1B	sp|P06576	1	25	25	25	71	56.559	3.2886E+10	2.9643E+10	3.1949E+10	41715000000	35224000000	3.8526E+10
sp|P06703	S100A6	sp|P06703	1	3	3	3	24	10.18	2.8156E+10	6023500000	3.4875E+10	23889000000	28768000000	4.0191E+10
sp|P06730	EIF4E	sp|P06730	1	5	5	5	36	25.097	1465600000	1389200000	916530000	1017800000	1067500000	1239200000
sp|P06733	ENO1	sp|P06733	1	24	24	21	71	47.168	1.3938E+11	1.5757E+11	1.5238E+11	1.2747E+11	1.312E+11	1.2922E+11
sp|P06737	PYGL	sp|P06737	1	27	27	22	38	97.147	2561600000	2313500000	2316800000	2290700000	1986900000	2055600000
sp|P06744	GPI	sp|P06744	2	22	22	17	51	63.146	2.1387E+10	2.1577E+10	2.3517E+10	19515000000	22063000000	2.1673E+10
sp|P06748	NPM1	sp|P06748	1	11	11	11	44	32.575	4.0671E+10	5.1833E+10	5.2599E+10	47591000000	49605000000	5.5484E+10
sp|P06753	TPM3	sp|P06753	1	15	5	5	40	32.95	5047300000	7393100000	5664300000	5762400000	7396600000	6406900000
sp|P06756	ITGAV	sp|P06756	1	20	20	20	24	116.04	1578900000	1700300000	1530500000	1183600000	1238000000	1261100000
sp|P06858	LPL	sp|P06858	1	3	3	3	10	53.162	145620000	157170000	164630000	148260000	159600000	104340000
sp|P06865	HEXA	sp|P06865	1	4	4	4	8.9	60.702	0	221540000	174380000	210490000	0	211980000
sp|Q16778	HIST2H2E	sp|Q16778	6	6	6	0	41	13.92	8.6605E+10	1.0164E+11	1.0324E+11	1.0924E+11	1.0507E+11	1.0175E+11
sp|P07093	SERPINE	sp|P07093	1	1	1	1	4.5	44.002	0	13182000	0	52246000	23929000	17919000
sp|P07099	EPHX1	sp|P07099	1	17	17	17	54	52.948	4347700000	5006900000	4348900000	4783900000	4609200000	5386600000
sp|P07108	DBI	sp|P07108	1	6	6	6	61	10.044	3840500000	4120800000	2999900000	3781600000	3778200000	3015100000
sp|P07195	LDHB	sp|P07195	1	20	19	19	56	36.638	5.6804E+10	6.4408E+10	6.1062E+10	60772000000	57477000000	5.008E+10
sp|P07203	GPX1	sp|P07203	1	10	10	10	59	22.088	727480000	749340000	932960000	1127200000	1069900000	1009000000
sp|P07237	P4HB	sp|P07237	1	32	32	32	67	57.116	2.822E+10	2.637E+10	2.4862E+10	30628000000	29025000000	2.9233E+10
sp|P07305	H1F0	sp|P07305	1	3	3	3	16	20.863	118080000	95233000	147280000	286610000	291250000	0
sp|P07311	ACYP1	sp|P07311	1	2	2	2	23	11.261	170940000	177000000	132930000	107840000	117290000	0
sp|P07339	CTSD	sp|P07339	1	14	14	14	45	44.552	1796200000	2378100000	2265100000	2419200000	2673200000	2264200000
sp|P07355	ANXA2	sp|P07355	2	28	28	28	68	38.604	1.138E+11	1.2734E+11	1.1257E+11	1.2608E+11	1.2891E+11	1.2547E+11
sp|P07384	CAPN1	sp|P07384	1	20	20	20	34	81.889	1047900000	1463200000	1315100000	1202900000	1140100000	987920000
sp|P07437	TUBB	sp|P07437	2	22	5	4	70	49.67	2.8015E+10	3.3499E+10	2.1109E+10	25943000000	26098000000	2.4492E+10

sp|P07602	PSAP	sp|P07602	1	13	13	13	28	58.112	3209700000	2769700000	3091000000	3855000000	3574200000	3246300000
sp|P07686	HEXB	sp|P07686	1	7	7	7	19	63.111	599420000	714790000	655740000	687270000	637360000	698770000
sp|P07711	CTSL	sp|P07711	1	2	2	2	7.8	37.564	0	0	0	0	0	215900000
sp|P07737	PFN1	sp|P07737	2	11	11	11	90	15.054	5.5468E+10	6.112E+10	4.37E+10	50837000000	56515000000	5.7448E+10
sp|P07741	APRT	sp|P07741	1	9	9	9	55	19.608	1537800000	1760200000	1450000000	1792200000	1834100000	1505500000
sp|P07814	EPRS	sp|P07814	1	56	56	55	50	170.59	1.7579E+10	1.8016E+10	1.6648E+10	18660000000	16909000000	1.9043E+10
sp|P07858	CTSB	sp|P07858	1	2	2	2	6.2	37.821	0	0	0	159410000	0	0
sp|P07900	HSP90AA	sp|P07900	4	44	28	28	59	84.659	4.9572E+10	5.0568E+10	5.1136E+10	51274000000	44352000000	4.7651E+10
sp|P07910	HNRNPC	sp|P07910	5	13	13	13	41	33.67	1.5786E+10	1.4492E+10	1.7599E+10	16187000000	18020000000	1.9596E+10
sp|P07919	UQCRH	sp|P07919	2	5	5	5	67	10.739	1964400000	2108900000	1939100000	1975900000	2040500000	2767900000
sp|P07942	LAMB1	sp|P07942	1	9	9	9	8.3	198.04	176280000	226280000	227470000	260680000	260950000	160990000
sp|P07954	FH	sp|P07954	1	17	17	17	50	54.636	3680500000	4347700000	4046000000	4214400000	4072500000	4127200000
sp|P08047	SP1	sp|P08047	1	2	2	2	5.5	80.692	0	0	0	0	0	75212000
sp|P08133	ANXA6	sp|P08133	1	31	31	31	56	75.872	4770000000	4834100000	5057600000	4588300000	4842000000	4846400000
sp|P08134	RHOC	sp|P08134	1	7	2	2	45	22.006	346240000	327340000	310300000	196960000	392050000	280090000
sp|P08174	CD55	sp|P08174	1	15	15	15	42	41.4	3495100000	3668500000	3289400000	5195600000	5717700000	5508100000
sp|P08195	SLC3A2	sp|P08195	1	25	25	25	42	67.993	2.829E+10	2.5481E+10	2.8328E+10	32979000000	39107000000	3.6048E+10
sp|P08237	PFKM	sp|P08237	1	20	18	17	35	85.182	2663900000	2453300000	2601700000	2195800000	2301500000	2383100000
sp|P08238	HSP90AB	sp|P08238	2	51	51	27	64	83.263	1.511E+11	1.6378E+11	1.5149E+11	1.4991E+11	1.551E+11	1.7473E+11
sp|P08240	SRPRA	sp|P08240	1	10	10	10	20	69.81	406520000	556930000	287320000	395120000	522250000	0
sp|P08243	ASNS	sp|P08243	1	16	16	16	36	64.369	1169100000	1164500000	1429400000	2218800000	1827200000	1645900000
sp|P08397	HMBS	sp|P08397	1	5	5	5	18	39.33	0	121760000	0	0	0	0
sp|P08559	PDHA1	sp|P08559	2	17	17	17	57	43.295	3230300000	3428100000	3247500000	2902300000	3034600000	3879300000
sp|P08572	COL4A2	sp|P08572	1	5	5	5	3.9	167.55	0	0	0	201510000	220110000	176720000
sp|P08574	CYC1	sp|P08574	1	7	7	7	35	35.422	1994400000	1899900000	2232000000	1849400000	1288300000	1931200000
sp|P08579	SNRPB2	sp|P08579	1	7	5	5	35	25.486	1083700000	1092400000	1012400000	1003800000	965030000	1083400000
sp|P08581	MET	sp|P08581	1	7	7	7	8.7	155.54	155870000	167860000	196730000	187280000	175830000	188970000
sp|P08621	SNRNP70	sp|P08621	1	15	15	15	29	51.556	3704200000	3913500000	3559000000	2947500000	3630000000	4646600000
sp|P08648	ITGA5	sp|P08648	1	16	16	16	22	114.54	1481000000	1626800000	1540400000	1707400000	1564100000	1573700000
sp|P08651	NFIC	sp|P08651	1	2	2	2	8.9	55.674	168930000	154410000	164450000	135930000	154200000	0
sp|P08670	VIM	sp|P08670	8	42	42	39	83	53.651	2.1934E+11	2.2951E+11	1.9611E+11	2.1612E+11	2.5771E+11	2.2382E+11
sp|P08708	RPS17	sp|P08708	1	8	8	8	57	15.55	8691500000	6592100000	6769200000	6948400000	8443500000	6948000000
sp|P08754	GNAI3	sp|P08754	1	12	7	7	48	40.532	1268300000	794420000	1084600000	948750000	803370000	1265100000
sp|P08758	ANXA5	sp|P08758	1	20	20	20	70	35.936	2.0088E+10	1.9815E+10	2.0061E+10	20784000000	21438000000	1.6621E+10
sp|P08865	RPSA	sp|P08865	1	15	15	15	59	32.854	1.9165E+10	2.3768E+10	2.1059E+10	21010000000	21912000000	1.907E+10
sp|P08962	CD63	sp|P08962	1	2	2	2	7.6	25.636	1896900000	1504100000	1360000000	1455100000	1688400000	1505200000
sp|P09001	MRPL3	sp|P09001	1	6	6	6	28	38.632	173680000	159600000	206770000	173160000	232730000	0
sp|P09012	SNRPA	sp|P09012	1	8	8	6	30	31.279	1812300000	990880000	1603200000	1216000000	1713600000	1378000000
sp|P09038	FGF2	sp|P09038	1	5	5	5	16	30.77	334800000	244930000	268300000	380310000	317770000	417230000

sp|P09104	ENO2	sp|P09104	1	8	5	5	33	47.268	666350000	564250000	658280000	564400000	517930000	479700000
sp|P09110	ACAA1	sp|P09110	1	10	10	10	44	44.292	803980000	662910000	741250000	916770000	841810000	1037000000
sp|P09132	SRP19	sp|P09132	1	4	4	4	46	16.156	367690000	333840000	305540000	458020000	147740000	388500000
sp|P09211	GSTP1	sp|P09211	1	12	12	12	70	23.356	2.1247E+10	1.9626E+10	1.8902E+10	21784000000	20444000000	1.8711E+10
sp|P09234	SNRPC	sp|P09234	1	3	3	3	25	17.394	0	1058700000	1063800000	0	0	1248800000
sp|P09382	LGALS1	sp|P09382	1	10	10	10	91	14.716	2.3237E+10	2.857E+10	2.2801E+10	25052000000	25270000000	2.3983E+10
sp|P09417	QDPR	sp|P09417	1	8	8	8	46	25.789	430780000	374220000	454920000	407950000	434110000	330380000
sp|P09429	HMGB1	sp|P09429	2	12	12	9	47	24.893	1.7674E+10	1.4571E+10	1.684E+10	15295000000	16123000000	1.6439E+10
sp|P09488	GSTM1	sp|P09488	1	9	9	1	53	25.712	923150000	1044700000	1210500000	899110000	761270000	982860000
sp|P09493	TPM1	sp|P09493	1	16	12	5	46	32.708	6920600000	6461800000	6428400000	5248500000	6018000000	4990500000
sp|P09496	CLTA	sp|P09496	1	6	6	6	19	27.076	3100400000	2489900000	2010500000	2755400000	2994100000	2961800000
sp|P09497	CLTB	sp|P09497	1	6	6	6	22	25.19	356480000	292420000	342440000	0	442320000	374000000
sp|P09525	ANXA4	sp|P09525	1	12	12	12	46	35.882	1410600000	1046800000	1263000000	979900000	1154500000	1164500000
sp|P09543	CNP	sp|P09543	1	11	11	11	31	47.578	1079700000	1187400000	1137800000	1018800000	1300900000	1118900000
sp|P09601	HMOX1	sp|P09601	1	8	8	8	42	32.818	655640000	663010000	677190000	570450000	601200000	569340000
sp|P09622	DLD	sp|P09622	1	10	10	10	25	54.177	2553900000	2123400000	3392300000	2219400000	1867400000	2430200000
sp|P09651	HNRNPA1	sp|P09651	2	16	16	16	48	38.746	2.5174E+10	2.23E+10	2.4563E+10	22385000000	21266000000	2.287E+10
sp|P09661	SNRPA1	sp|P09661	1	8	8	8	43	28.415	1227500000	1637400000	1155900000	1061200000	991700000	986620000
sp|P09669	COX6C	sp|P09669	1	2	2	2	25	8.7813	457930000	358980000	433830000	295120000	401810000	320990000
sp|P09758	TACSTD2	sp|P09758	1	1	1	1	5	35.709	0	0	0	0	0	32365000
sp|P09874	PARP1	sp|P09874	1	42	42	42	49	113.08	8403400000	1.0045E+10	8965600000	9072200000	9068700000	9606000000
sp|P09884	POLA1	sp|P09884	1	12	12	12	12	165.91	424590000	491940000	484280000	408230000	357440000	403510000
sp|P09913	IFIT2	sp|P09913	1	1	1	1	2.3	54.632	0	0	0	0	0	48348000
sp|P09914	IFIT1	sp|P09914	2	5	5	5	16	55.36	123170000	138030000	134450000	112990000	178480000	162600000
sp|P09960	LTA4H	sp|P09960	1	17	17	17	39	69.284	3677100000	3406600000	3466000000	2757400000	3120200000	2832300000
sp|P09972	ALDOC	sp|P09972	1	7	2	2	24	39.455	5545600000	5893100000	4663100000	6653600000	4625900000	4854800000
sp|Q71UI9	H2AFV	sp|Q71UI9	2	4	4	2	31	13.509	4.408E+10	3.8475E+10	4.5845E+10	44031000000	42718000000	4.3037E+10
sp|P0CG3	POTEJ	sp|P0CG3	1	7	1	1	9.6	117.39	0	0	0	0	0	315650000
sp|P0DMN	SULT1A4	sp|P0DMN	3	2	2	2	11	34.196	74729000	54853000	86782000	48017000	78297000	65782000
sp|P0DMV	HSPA1B	sp|P0DMV	2	31	29	16	60	70.051	4.9914E+10	6.1458E+10	5.2616E+10	60482000000	62009000000	6.2277E+10
sp|Q01081	U2AF1	sp|Q01081	3	7	7	7	29	27.872	2327500000	2244800000	2193400000	2828300000	2492900000	2341000000
sp|P35520	CBS	sp|P35520	2	11	11	11	33	60.586	693790000	949860000	871000000	1053800000	819750000	933080000
sp|P0DP2	CALM3	sp|P0DP2	5	11	11	11	60	16.837	1.4585E+10	1.6098E+10	1.3786E+10	14301000000	16601000000	1.4165E+10
sp|P0DP9	CSB-PGB	sp|P0DP9	2	1	1	1	1.2	119.49	0	0	0	0	47589000	0
sp|P0DPB	POLR1D	sp|P0DPB	1	1	1	1	14	15.237	0	0	0	0	185390000	0
sp|P10109	FDX1	sp|P10109	1	1	1	1	11	19.393	14682000	23052000	25523000	15230000	22619000	31846000
sp|P10155	TROVE2	sp|P10155	1	13	13	13	28	60.67	1016000000	877830000	1097400000	1079900000	1085600000	815550000
sp|P10301	RRAS	sp|P10301	1	3	2	2	19	23.48	0	0	0	0	0	49844000
sp|P10316	HLA-A	sp|P10316	2	16	2	0	46	40.976	0	0	0	0	270900000	239080000

sp|P10321	HLA-C	sp|P10321	1	9	1	1	31	40.648	358100000	0	434320000	562890000	0	576650000
sp|P10398	ARAF	sp|P10398	2	4	4	3	7.4	67.585	196680000	167570000	197180000	206380000	224610000	220430000
sp|P10412	HIST1H1E	sp|P10412	1	7	1	1	23	21.865	0	0	0	0	0	271300000
sp|P10515	DLAT	sp|P10515	1	10	10	10	21	68.996	738000000	953120000	751510000	844360000	1036100000	846820000
sp|P10599	TXN	sp|P10599	1	7	7	7	74	11.737	1.9567E+10	1.8328E+10	2.1981E+10	15061000000	16732000000	2.1493E+10
sp|P10606	COX5B	sp|P10606	1	5	5	5	40	13.696	1081800000	878100000	973980000	1078900000	1444400000	1152300000
sp|P10619	CTSA	sp|P10619	1	3	3	3	7.7	54.465	422080000	482930000	0	433360000	0	338690000
sp|P10620	MGST1	sp|P10620	1	4	4	4	27	17.598	2936100000	2641200000	2594800000	3364200000	2681500000	3302800000
sp|P10644	PRKAR1A	sp|P10644	1	8	8	7	21	42.981	623280000	531400000	811940000	722110000	690810000	554730000
sp|P10646	TFPI	sp|P10646	1	1	1	1	6.9	35.015	0	0	0	0	0	51984000
sp|P10768	ESD	sp|P10768	1	11	11	11	53	31.462	2066500000	2152000000	1885400000	1670400000	1783500000	1551300000
sp|P10809	HSPD1	sp|P10809	1	45	45	45	80	61.054	8.7005E+10	9.5645E+10	9.0117E+10	1.0383E+11	97518000000	1.0364E+11
sp|P10909	CLU	sp|P10909	1	9	9	9	26	52.494	1544000000	1257700000	1465200000	1415000000	1930700000	1002600000
sp|P11021	HSPA5	sp|P11021	1	32	31	31	49	72.332	5.4704E+10	6.0383E+10	5.1164E+10	79702000000	76743000000	7.6505E+10
sp|P11047	LAMC1	sp|P11047	1	16	16	16	16	177.6	482940000	534900000	508220000	495670000	481500000	528730000
sp|P11055	MYH3	sp|P11055	1	2	1	1	1.2	223.9	0	0	0	0	0	70752000
sp|P11117	ACP2	sp|P11117	1	6	6	6	17	48.344	155100000	114650000	190420000	177260000	143340000	167500000
sp|P11137	MAP2	sp|P11137	1	1	1	1	0.5	199.52	0	0	0	0	34879000	0
sp|P11142	HSPA8	sp|P11142	2	33	32	28	58	70.897	1.1675E+11	1.1398E+11	1.1736E+11	1.1297E+11	1.2562E+11	1.3109E+11
sp|P11166	SLC2A1	sp|P11166	1	6	6	6	12	54.083	1335800000	1503000000	1252700000	1146300000	1220000000	1162100000
sp|P11171	EPB41	sp|P11171	1	3	2	2	5.3	97.016	87707000	0	0	0	0	0
sp|P11172	UMPS	sp|P11172	1	13	13	13	34	52.221	1307300000	991910000	1739300000	1512000000	1389000000	1486000000
sp|P11177	PDHB	sp|P11177	1	11	11	11	44	39.233	3512600000	4064000000	2864800000	3788100000	4287400000	4304500000
sp|P11182	DBT	sp|P11182	1	2	2	2	4.8	53.486	0	0	0	0	0	70616000
sp|P11216	PYGB	sp|P11216	1	17	13	11	25	96.695	509050000	434890000	410910000	565530000	658220000	318830000
sp|P11233	RALA	sp|P11233	1	5	5	4	26	23.567	1089100000	1198700000	1153800000	1061100000	1106800000	1037500000
sp|P11234	RALB	sp|P11234	1	2	1	1	11	23.408	0	0	0	22096000	0	0
sp|P11274	BCR	sp|P11274	1	3	3	3	3.9	142.82	0	50101000	0	0	71102000	0
sp|P11279	LAMP1	sp|P11279	1	4	4	4	9.8	44.882	1753100000	840660000	1099100000	1260200000	1831600000	1875700000
sp|P11310	ACADM	sp|P11310	1	9	9	9	31	46.588	788780000	806770000	881380000	896970000	1099700000	979600000
sp|P11387	TOP1	sp|P11387	2	23	23	23	31	90.725	3216300000	2615000000	2607800000	3011900000	3159000000	3533700000
sp|P11388	TOP2A	sp|P11388	1	41	41	31	32	174.38	4516400000	4149800000	4323800000	4119400000	3720800000	4238900000
sp|P11413	G6PD	sp|P11413	1	32	32	32	62	59.256	1.7767E+10	1.7457E+10	1.7086E+10	17024000000	15734000000	1.6423E+10
sp|P11441	UBL4A	sp|P11441	1	5	5	5	36	17.776	154010000	168730000	0	165850000	176560000	158190000
sp|P11498	PC	sp|P11498	1	23	23	23	28	129.63	1846200000	1526600000	1920800000	1730500000	1553500000	1715700000
sp|P11586	MTHFD1	sp|P11586	1	37	37	36	50	101.56	1.1119E+10	9883100000	1.1038E+10	10970000000	10518000000	1.1773E+10
sp|P11717	IGF2R	sp|P11717	1	38	38	38	23	274.37	2503700000	2460000000	2432500000	2727600000	2702600000	2533600000
sp|P11766	ADH5	sp|P11766	1	14	14	14	50	39.724	3918300000	4635700000	4060500000	3944000000	3795200000	3849200000
sp|P11802	CDK4	sp|P11802	1	7	7	7	31	33.729	308990000	241120000	329470000	282810000	253950000	236360000

sp|P11908	PRPS2	sp|P11908	1	13	5	4	49	34.769	432720000	254750000	245920000	394870000	347940000	255170000
sp|P11940	PABPC1	sp|P11940	4	28	28	20	56	70.67	1.4221E+10	1.4897E+10	1.3601E+10	13103000000	13508000000	1.6339E+10
sp|P12004	PCNA	sp|P12004	1	11	11	11	54	28.768	5561700000	5694200000	5237500000	5653200000	6608500000	6032200000
sp|P12081	HARS	sp|P12081	1	21	21	16	49	57.41	3616100000	3329500000	3715000000	3304200000	3506900000	3099800000
sp|P12235	SLC25A4	sp|P12235	1	11	1	1	39	33.064	0	0	0	0	0	42218000
sp|P12236	SLC25A6	sp|P12236	1	16	16	3	52	32.866	1.3823E+10	1.8036E+10	1.7134E+10	19739000000	17131000000	2.117E+10
sp|P12268	IMPDH2	sp|P12268	1	24	24	23	62	55.804	8516100000	9147100000	8606200000	9318000000	10381000000	1.0354E+10
sp|P12270	TPR	sp|P12270	1	63	63	63	33	267.29	5775100000	5337200000	5853700000	5416400000	5530700000	6309000000
sp|P12277	CKB	sp|P12277	2	18	18	18	71	42.644	7149900000	9396500000	7153600000	7043500000	6842600000	6456700000
sp|P12429	ANXA3	sp|P12429	1	18	18	18	61	36.375	5997600000	7583300000	6150200000	5196400000	5679000000	5681300000
sp|P12694	BCKDHA	sp|P12694	1	3	3	3	10	50.47	0	0	0	0	52452000	44012000
sp|P12814	ACTN1	sp|P12814	1	52	36	31	66	103.06	1.4724E+10	1.5537E+10	1.444E+10	14833000000	13660000000	1.2475E+10
sp|P12955	PEPD	sp|P12955	1	5	5	5	16	54.548	183350000	246150000	274640000	201300000	230170000	178400000
sp|P12956	XRCC6	sp|P12956	1	34	34	34	61	69.842	2.0305E+10	2.1016E+10	2.0712E+10	22450000000	19278000000	2.0232E+10
sp|P13010	XRCC5	sp|P13010	1	33	33	33	57	82.704	1.5982E+10	1.5771E+10	1.6972E+10	15825000000	13582000000	1.7124E+10
sp|P13073	COX4I1	sp|P13073	1	6	6	6	39	19.576	3057800000	3119600000	2917800000	2579800000	3125300000	2289100000
sp|P13473	LAMP2	sp|P13473	1	3	3	3	7.1	44.96	146060000	1056700000	0	1260200000	908400000	1165500000
sp|P13489	RNH1	sp|P13489	1	18	18	18	59	49.973	2807800000	3209100000	3480500000	3188300000	2855700000	2926900000
sp|P13639	EEF2	sp|P13639	1	49	49	48	61	95.337	8.7703E+10	8.7795E+10	7.9036E+10	81272000000	75057000000	7.0376E+10
sp|P13667	PDIA4	sp|P13667	1	31	31	31	51	72.932	7846200000	6918600000	6240100000	7944600000	9194500000	9210400000
sp|P13674	P4HA1	sp|P13674	1	14	14	14	34	61.049	863020000	1003400000	1047000000	1179700000	1066900000	1136500000
sp|P13693	TPT1	sp|P13693	2	5	5	5	31	19.595	2944100000	3586500000	3168100000	3150800000	3308900000	2719500000
sp|P13797	PLS3	sp|P13797	2	36	36	33	59	70.81	1.987E+10	2.2041E+10	2.0289E+10	20180000000	18728000000	1.675E+10
sp|P13798	APEH	sp|P13798	1	13	13	13	29	81.224	1198100000	1251600000	1238100000	1064700000	1130800000	1133100000
sp|P13804	ETFA	sp|P13804	1	14	14	14	52	35.079	3475200000	2720300000	3184400000	4011400000	4476400000	4646200000
sp|P13807	GYS1	sp|P13807	1	10	10	10	21	83.785	332370000	378240000	326970000	351550000	405060000	282970000
sp|P13861	PRKAR2A	sp|P13861	2	17	17	17	58	45.518	1556100000	1528900000	1727000000	1466900000	1431400000	1489600000
sp|P13929	ENO3	sp|P13929	1	9	6	6	34	46.986	346000000	600180000	511370000	688750000	867870000	438910000
sp|P13984	GTF2F2	sp|P13984	1	4	4	4	25	28.38	0	0	244000000	380200000	0	252660000
sp|P13987	CD59	sp|P13987	1	4	4	4	25	14.177	5983700000	6996600000	5811100000	6349900000	7951400000	6625900000
sp|P13995	MTHFD2	sp|P13995	1	6	6	6	27	37.895	541780000	285930000	587030000	855930000	682450000	590700000
sp|P14174	MIF	sp|P14174	1	4	4	4	37	12.476	8238700000	8578600000	8631900000	8646200000	7745200000	7109200000
sp|P14209	CD99	sp|P14209	1	3	3	3	30	18.848	475400000	362600000	476210000	376670000	432120000	493900000
sp|P14314	PRKCSH	sp|P14314	1	21	21	21	51	59.425	8574500000	8456100000	1.0539E+10	9565000000	9794200000	1.0223E+10
sp|P14324	FDPS	sp|P14324	1	8	8	8	22	48.275	4862400000	5236300000	4803700000	4388000000	4015600000	3091800000
sp|P14384	CPM	sp|P14384	1	2	2	2	10	50.513	0	0	24902000	28460000	0	0
sp|P14406	COX7A2	sp|P14406	1	2	2	2	28	9.3959	0	241670000	0	716430000	879310000	0
sp|P14550	AKR1A1	sp|P14550	1	16	16	16	63	36.573	2282700000	2728700000	2265700000	2090600000	2093200000	2040200000
sp|P14618	PKM	sp|P14618	2	37	37	37	72	57.936	1.5501E+11	1.7151E+11	1.763E+11	1.5721E+11	1.5621E+11	1.7659E+11

sp|P14625	HSP90B1	sp|P14625	2	38	36	36	51	92.468	2.5594E+10	2.6736E+10	2.9948E+10	30947000000	30942000000	2.8562E+10
sp|P14635	CCNB1	sp|P14635	1	6	6	6	23	48.337	416300000	508690000	496980000	342960000	291800000	336800000
sp|P63162	SNRPN	sp|P63162	2	6	6	6	22	24.614	2408700000	2276600000	2649200000	2198700000	2209900000	2230100000
sp|P14735	IDE	sp|P14735	1	14	14	14	17	117.97	937750000	1128600000	1044100000	978220000	1146900000	1172100000
sp|P14854	COX6B1	sp|P14854	1	3	3	3	48	10.192	998160000	1092600000	716620000	589920000	540270000	513670000
sp|P14859	POU2F1	sp|P14859	3	2	2	2	4.2	76.471	99584000	63651000	0	86276000	83468000	0
sp|P14866	HNRNPL	sp|P14866	1	22	22	21	60	64.132	1.2227E+10	1.6773E+10	1.605E+10	16550000000	15967000000	1.7244E+10
sp|P14868	DARS	sp|P14868	1	21	21	21	50	57.136	4954300000	5289600000	5414300000	4957400000	4601900000	6118000000
sp|P14923	JUP	sp|P14923	1	26	26	21	48	81.744	3166100000	2549200000	3012100000	2592000000	2578100000	2636500000
sp|P14927	UQCRB	sp|P14927	1	3	3	3	34	13.53	611490000	624490000	829710000	819120000	0	704700000
sp|P15104	GLUL	sp|P15104	1	2	2	2	6.2	42.064	95545000	84034000	87250000	0	55887000	0
sp|P15121	AKR1B1	sp|P15121	2	12	12	12	44	35.853	1.2198E+10	1.1114E+10	1.3224E+10	11592000000	13382000000	9453000000
sp|P15144	ANPEP	sp|P15144	1	5	5	5	6.7	109.54	103880000	106780000	0	0	79805000	0
sp|P15151	PVR	sp|P15151	1	2	2	2	8.4	45.302	277480000	0	0	159760000	0	335370000
sp|P15170	GSPT1	sp|P15170	1	17	17	10	39	55.755	3569400000	3735800000	3743400000	3883500000	3882400000	4072600000
sp|P15291	B4GALT1	sp|P15291	1	7	7	7	31	43.92	756220000	910860000	922210000	1109800000	1081200000	917130000
sp|P15311	EZR	sp|P15311	1	37	24	23	54	69.412	1.8397E+10	1.9292E+10	1.5393E+10	15816000000	17791000000	1.657E+10
sp|P15328	FOLR1	sp|P15328	1	6	6	6	27	29.819	2133000000	2145100000	2359400000	2546800000	2381800000	2361100000
sp|P15374	UCHL3	sp|P15374	1	8	8	8	50	26.182	1044800000	1357500000	1242600000	1066500000	1141200000	975720000
sp|P15408	FOSL2	sp|P15408	1	1	1	1	3.7	35.193	0	0	0	0	0	62781000
sp|P15529	CD46	sp|P15529	1	3	3	3	8.7	43.747	0	329250000	0	265030000	244670000	0
sp|P15531	NME1	sp|P15531	1	11	6	6	70	17.149	8905600000	7589600000	9029800000	7208800000	8353600000	9341300000
sp|P15559	NQO1	sp|P15559	1	11	11	11	39	30.867	7611600000	8639400000	7966600000	6357500000	5979200000	6785100000
sp|P15586	GNS	sp|P15586	1	4	4	4	8.3	62.081	114710000	0	0	248570000	531670000	0
sp|P15880	RPS2	sp|P15880	1	15	15	15	56	31.324	1.4748E+10	1.7748E+10	1.5651E+10	14741000000	16021000000	1.4176E+10
sp|P15924	DSP	sp|P15924	1	22	22	22	10	331.77	487230000	589870000	588090000	559310000	466370000	578840000
sp|P15927	RPA2	sp|P15927	1	5	5	5	38	29.247	1392600000	450510000	991800000	873580000	893730000	868590000
sp|P15941	MUC1	sp|P15941	1	2	2	2	1.8	122.1	0	0	0	42194000	90609000	0
sp|P15954	COX7C	sp|P15954	1	1	1	1	14	7.2454	0	0	0	0	0	202170000
sp|P16035	TIMP2	sp|P16035	1	1	1	1	6.4	24.399	0	0	0	0	0	0
sp|P16070	CD44	sp|P16070	1	9	9	9	12	81.537	5323800000	4907700000	4465800000	4622300000	5565100000	5754700000
sp|P16083	NQO2	sp|P16083	1	3	3	3	20	25.918	355220000	386160000	319100000	418370000	466220000	366530000
sp|P16144	ITGB4	sp|P16144	1	7	7	7	6.5	202.16	155180000	156220000	183350000	134850000	124960000	142170000
sp|P16152	CBR1	sp|P16152	1	15	15	13	65	30.375	7114700000	7288100000	7872900000	7913700000	6662300000	5944000000
sp|P16220	CREB1	sp|P16220	1	3	3	2	15	36.688	0	0	0	0	210120000	0
sp|P16278	GLB1	sp|P16278	1	2	2	2	4.4	76.074	80289000	105180000	0	113890000	94695000	0
sp|P16401	HIST1H1E	sp|P16401	1	5	5	5	22	22.58	5879100000	8790900000	8572200000	6867300000	10580000000	1.1919E+10
sp|P16402	HIST1H1D	sp|P16402	1	7	1	1	23	22.35	0	0	0	0	0	208340000
sp|P16403	HIST1H1C	sp|P16403	2	7	7	1	24	21.364	1.9977E+10	2.4118E+10	2.2931E+10	25697000000	34939000000	2.7908E+10

sp|P1643	POR	sp|P1643	1	16	16	16	29	76.689	1234800000	1088100000	1186400000	1437300000	1535000000	1166000000
sp|P1645	MGMT	sp|P1645	1	3	3	3	22	21.646	0	99754000	94263000	156780000	0	87667000
sp|P1652	GNB3	sp|P1652	1	3	1	1	7.1	37.221	0	506490000	0	0	0	0
sp|P1661	ATP2A2	sp|P1661	3	40	40	40	45	114.76	6236400000	6919700000	6904600000	7957500000	7723300000	7998000000
sp|P1693	FAH	sp|P1693	1	3	3	3	12	46.374	84751000	84856000	154700000	93026000	81005000	123170000
sp|P1694	STMN1	sp|P1694	1	6	6	5	38	17.302	2919500000	3356300000	3099500000	3525900000	2687400000	2793000000
sp|P1698	YBX3	sp|P1698	1	12	8	8	58	40.089	1854800000	1802000000	2279800000	1609600000	1887000000	2131100000
sp|P1706	HSPA6	sp|P1706	2	11	2	2	17	71.027	7146000000	1.1878E+10	9203700000	11591000000	11924000000	1.8096E+10
sp|P1709	HMGA1	sp|P1709	1	4	4	4	41	11.676	1800200000	1092400000	1809000000	804390000	1230600000	1221200000
sp|P1715	TMEM11	sp|P1715	1	2	2	2	13	21.541	0	0	0	0	156980000	0
sp|P1717	GOT1	sp|P1717	1	16	16	16	51	46.247	2724000000	2552900000	2560000000	3014300000	2785700000	2251300000
sp|P1725	PRKCA	sp|P1725	3	11	11	11	26	76.749	800260000	670610000	705280000	677810000	794530000	786280000
sp|P1727	JUNB	sp|P1727	1	5	5	5	27	35.879	0	0	0	201920000	240230000	253540000
sp|P1730	ITGA2	sp|P1730	1	15	15	15	23	129.29	492550000	328070000	460040000	457220000	503890000	612870000
sp|P1734	NPR3	sp|P1734	1	2	2	2	6.1	59.807	0	0	0	0	85157000	0
sp|P1748	UBTF	sp|P1748	1	12	12	12	18	89.405	689740000	816350000	539900000	760080000	716910000	583240000
sp|P1754	ATF7	sp|P1754	1	2	2	2	6.7	52.967	48140000	0	0	0	0	0
sp|P1761	PRKACA	sp|P1761	1	11	3	3	37	40.589	307220000	258410000	234160000	247170000	305890000	365930000
sp|P1765	CAPN2	sp|P1765	1	23	23	23	44	79.994	5941900000	5773500000	5400600000	5812900000	6152200000	6162200000
sp|P1767	CEBPB	sp|P1767	1	4	4	4	17	36.105	381150000	980070000	0	0	513210000	0
sp|P1781	CTPS1	sp|P1781	1	21	21	20	47	66.69	4831300000	4885200000	4225400000	5492900000	5702100000	6181300000
sp|P1781	ENG	sp|P1781	1	8	8	8	17	70.577	871830000	815640000	931490000	729850000	842950000	717570000
sp|P1784	DDX5	sp|P1784	1	34	34	25	54	69.147	1.5712E+10	1.6944E+10	1.4925E+10	17048000000	15705000000	1.6361E+10
sp|P1785	PFKL	sp|P1785	1	14	10	10	23	85.018	673050000	465360000	664330000	744030000	747710000	774460000
sp|P1790	GM2A	sp|P1790	1	2	2	2	16	20.838	61554000	54864000	65581000	0	70185000	0
sp|P1793	LGALS3	sp|P1793	1	6	6	6	29	26.152	2187900000	1904300000	1568200000	1686900000	2257000000	2073500000
sp|P1798	PSMC3	sp|P1798	1	23	23	23	60	49.203	4957400000	5218700000	4907200000	4994500000	4621400000	4681000000
sp|P1798	TCP1	sp|P1798	1	28	28	28	67	60.343	1.7282E+10	1.5785E+10	1.6135E+10	18915000000	20543000000	1.6302E+10
sp|P1803	PTPN1	sp|P1803	1	11	11	11	33	49.966	1134400000	1232300000	1192800000	1039300000	1042000000	1589500000
sp|P1807	RPL35A	sp|P1807	1	3	3	3	21	12.538	2952100000	3386200000	2557900000	3351300000	4047500000	4062800000
sp|P1808	ITGB5	sp|P1808	1	3	3	3	5.5	88.053	64656000	0	0	0	0	58353000
sp|P1808	ARF4	sp|P1808	1	10	10	6	64	20.511	5372000000	5286500000	4939700000	5191600000	4873900000	5482800000
sp|P1812	RPL7	sp|P1812	1	18	18	18	58	29.225	1.5049E+10	1.5308E+10	1.3948E+10	17460000000	19500000000	1.6683E+10
sp|P1820	VCL	sp|P1820	1	49	49	49	51	123.8	1.9579E+10	2.0098E+10	2.0625E+10	18953000000	19218000000	2.1061E+10
sp|P1856	ITGB6	sp|P1856	1	1	1	1	1.1	85.935	0	0	0	0	0	375810000
sp|P1858	SON	sp|P1858	1	14	14	14	9.4	263.83	904270000	837980000	876560000	580540000	928680000	841930000
sp|P1861	NELFE	sp|P1861	1	5	5	5	21	43.239	225340000	256700000	249460000	210440000	265490000	204660000
sp|P1862	RPL17	sp|P1862	1	8	8	8	47	21.397	9821500000	1.0133E+10	9367400000	9246200000	8454200000	1.0179E+10
sp|P1866	PGAM1	sp|P1866	3	17	17	17	71	28.804	2.6206E+10	2.7411E+10	2.7024E+10	21406000000	25163000000	3.0191E+10

sp|P18754	RCC1	sp|P18754	1	13	13	13	54	44.969	2398800000	2532300000	2547400000	2631800000	2818100000	2679500000
sp|P18846	ATF1	sp|P18846	1	2	1	1	13	29.232	0	0	0	0	0	32234000
sp|P18858	LIG1	sp|P18858	1	5	5	5	8.5	101.73	207810000	223860000	240860000	181950000	169680000	220060000
sp|P18859	ATP5J	sp|P18859	1	6	6	6	52	12.587	1425700000	1650000000	1281900000	1679500000	1663700000	1635200000
sp|P18887	XRCC1	sp|P18887	1	4	4	4	8.8	69.476	0	309620000	0	0	220150000	0
sp|P19022	CDH2	sp|P19022	2	10	10	10	20	99.808	359300000	380510000	381980000	491890000	444730000	452410000
sp|P19174	PLCG1	sp|P19174	1	4	4	4	3.9	148.53	1247900000	0	0	0	0	0
sp|P19256	CD58	sp|P19256	1	2	2	2	11	28.147	94678000	0	0	0	0	0
sp|P19338	NCL	sp|P19338	1	27	27	27	34	76.613	2.8567E+10	2.7698E+10	2.9328E+10	27759000000	26391000000	2.7148E+10
sp|P19367	HK1	sp|P19367	2	26	26	22	36	102.48	1852300000	1929200000	2211800000	2208700000	2042000000	2310500000
sp|P19387	POLR2C	sp|P19387	1	5	5	5	27	31.441	281130000	231110000	196160000	190180000	309350000	230820000
sp|P19388	POLR2E	sp|P19388	1	5	5	5	27	24.551	732890000	713930000	603000000	557080000	586840000	623930000
sp|P19404	NDUFV2	sp|P19404	1	4	4	4	21	27.391	246830000	328710000	284380000	301470000	219530000	226030000
sp|P19525	EIF2AK2	sp|P19525	1	11	11	11	27	62.094	788810000	959300000	750420000	809240000	869430000	828040000
sp|P19623	SRM	sp|P19623	1	14	14	14	71	33.824	2565700000	2717800000	2587800000	2499100000	2925800000	3077500000
sp|P19784	CSNK2A2	sp|P19784	1	6	6	6	26	41.213	264690000	485340000	330160000	471850000	315820000	306920000
sp|P19838	NFKB1	sp|P19838	1	4	4	4	7.1	105.35	0	0	0	107990000	0	0
sp|P19971	TYMP	sp|P19971	1	5	5	5	18	49.955	42667000	43171000	43034000	44799000	68524000	54664000
sp|P20020	ATP2B1	sp|P20020	3	14	7	7	17	138.75	310140000	157450000	300380000	217970000	251150000	215250000
sp|P20042	EIF2S2	sp|P20042	1	15	15	15	54	38.388	5223400000	5944700000	4494000000	5498000000	5683900000	6562600000
sp|P20073	ANXA7	sp|P20073	1	10	10	10	24	52.739	1402800000	1460100000	1422800000	1300400000	1417200000	1625400000
sp|P20290	BTF3	sp|P20290	1	10	10	10	64	22.168	4221500000	3795600000	3741400000	2591900000	3040600000	2467600000
sp|P20336	RAB3A	sp|P20336	1	3	2	1	14	24.984	0	0	0	0	82775000	68160000
sp|P20338	RAB4A	sp|P20338	1	3	3	3	17	24.389	0	0	0	152790000	0	0
sp|P20339	RAB5A	sp|P20339	1	8	5	5	50	23.658	335840000	253200000	210990000	180790000	275340000	281810000
sp|P20340	RAB6A	sp|P20340	3	9	9	8	47	23.593	3405700000	3660100000	3479800000	3339000000	4257500000	3239600000
sp|P20585	MSH3	sp|P20585	1	3	3	3	2.9	127.41	0	0	0	0	48588000	0
sp|P20618	PSMB1	sp|P20618	1	9	9	9	43	26.489	4721800000	5929800000	5940200000	4698900000	5240200000	5403300000
sp|P20645	M6PR	sp|P20645	1	5	5	5	21	30.993	752500000	835940000	720150000	687630000	810990000	899610000
sp|P20674	COX5A	sp|P20674	1	8	8	8	53	16.762	2066800000	2876200000	1747800000	1692000000	1865900000	2101800000
sp|P20700	LMNB1	sp|P20700	1	34	32	32	61	66.408	1.3928E+10	1.2019E+10	1.364E+10	13197000000	13583000000	1.2633E+10
sp|P20810	CAST	sp|P20810	1	23	23	23	49	76.572	4534900000	4910300000	4744400000	4298700000	5015400000	4280800000
sp|P20839	IMPDH1	sp|P20839	1	13	12	12	36	55.405	930750000	1531100000	1233100000	1191000000	1093100000	1134100000
sp|P20908	COL5A1	sp|P20908	1	10	10	10	9.6	183.56	566940000	522500000	534490000	227900000	317340000	317870000
sp|P20936	RASA1	sp|P20936	1	1	1	1	1.3	116.4	0	0	0	0	0	0
sp|P20962	PTMS	sp|P20962	1	5	5	5	27	11.53	3776100000	3023500000	3882600000	2777800000	2644600000	3854500000
sp|Q9UQ8	CDK11A	sp|Q9UQ8	3	6	6	6	10	91.361	331960000	327370000	408650000	298990000	367210000	317910000
sp|P21266	GSTM3	sp|P21266	1	14	14	13	64	26.559	4107900000	3795900000	4141300000	4533200000	4250200000	4019600000
sp|P21281	ATP6V1B	sp|P21281	2	10	10	10	25	56.5	776050000	683850000	771120000	952490000	650630000	947240000

sp|P21283	ATP6V1C	sp|P21283	1	9	9	9	27	43.941	466510000	484580000	476890000	531220000	444290000	481840000
sp|P21291	CSRP1	sp|P21291	1	11	11	11	65	20.567	2.2442E+10	1.9896E+10	2.2922E+10	17407000000	19416000000	2.3993E+10
sp|P21333	FLNA	sp|P21333	1	##	124	##	70	280.74	2.5025E+11	2.7088E+11	2.3951E+11	2.4019E+11	2.4469E+11	2.5235E+11
sp|P21399	ACO1	sp|P21399	2	23	23	23	36	98.398	2071800000	2165600000	1901200000	2453200000	1949400000	1920600000
sp|P21796	VDAC1	sp|P21796	1	15	15	15	75	30.772	1.6012E+10	1.6262E+10	2.1321E+10	18756000000	17905000000	1.5202E+10
sp|P21912	SDHB	sp|P21912	1	4	4	4	18	31.629	0	553650000	539250000	281510000	0	681310000
sp|P21926	CD9	sp|P21926	1	3	3	3	18	25.416	577800000	642700000	815480000	713640000	905120000	972800000
sp|P21964	COMT	sp|P21964	1	9	9	9	45	30.037	1197100000	1305900000	1163300000	1377200000	1195200000	1376200000
sp|P21980	TGM2	sp|P21980	1	12	12	12	23	77.328	634900000	619070000	615440000	727730000	727150000	727680000
sp|P22033	MUT	sp|P22033	1	3	3	3	6.4	83.134	0	114920000	0	90328000	124580000	178770000
sp|P22059	OSBP	sp|P22059	2	10	10	10	18	89.42	563090000	512220000	360330000	623460000	514910000	575020000
sp|P22061	PCMT1	sp|P22061	1	7	7	7	52	24.636	1851800000	2210300000	2383400000	2185200000	1963400000	2429600000
sp|P22087	FBL	sp|P22087	1	10	10	9	40	33.784	3378100000	2974100000	3364500000	3477000000	4242800000	3114100000
sp|P22102	GART	sp|P22102	1	29	29	29	46	107.77	6093000000	5801600000	5789400000	5630900000	5202800000	4907400000
sp|P22234	PAICS	sp|P22234	1	22	22	22	52	47.079	1.209E+10	1.2638E+10	1.4035E+10	12577000000	10858000000	1.2494E+10
sp|P22307	SCP2	sp|P22307	1	8	8	8	14	58.993	1115200000	1467700000	1239100000	1603700000	1929800000	1257600000
sp|P22314	UBA1	sp|P22314	1	38	38	38	50	117.85	2.1066E+10	2.1785E+10	2.0205E+10	19907000000	21334000000	1.9963E+10
sp|P22392	NME2	sp|P22392	1	11	11	1	76	17.298	3.4079E+10	3.4449E+10	3.9687E+10	33183000000	31044000000	3.0786E+10
sp|P22570	FDXR	sp|P22570	1	2	2	2	5.7	53.836	0	0	0	0	0	36424000
sp|P22612	PRKACG	sp|P22612	1	3	1	1	15	40.434	0	0	0	0	0	55794000
sp|P22626	HNRNPA2	sp|P22626	1	20	20	20	65	37.429	5.1008E+10	4.8248E+10	4.3576E+10	44845000000	42984000000	5.2309E+10
sp|P22694	PRKACB	sp|P22694	1	12	12	4	46	40.622	911760000	1145800000	1113300000	1033200000	1190300000	1022000000
sp|P22695	UQCRC2	sp|P22695	1	17	17	17	55	48.442	4659700000	3813600000	4657500000	4933500000	5297000000	5785200000
sp|P22830	FECH	sp|P22830	1	3	3	3	10	47.862	0	336700000	0	0	321230000	389640000
sp|P23142	FBLN1	sp|P23142	1	1	1	1	2.4	77.213	0	0	0	0	0	0
sp|P23193	TCEA1	sp|P23193	2	13	13	13	50	33.969	1660600000	1653900000	1553800000	1701400000	1725100000	1794800000
sp|P23229	ITGA6	sp|P23229	1	16	16	16	18	126.6	886190000	721650000	754890000	706080000	809360000	848940000
sp|P23246	SFPQ	sp|P23246	1	31	31	30	51	76.149	1.9755E+10	2.2124E+10	2.1805E+10	23641000000	23356000000	2.4604E+10
sp|P23258	TUBG1	sp|P23258	2	11	11	11	43	51.169	912340000	787280000	637220000	721340000	622970000	801010000
sp|P23284	PPIB	sp|P23284	1	14	14	14	55	23.742	1.4327E+10	1.5546E+10	1.2542E+10	16475000000	17307000000	1.6499E+10
sp|P23368	ME2	sp|P23368	1	12	12	12	34	65.443	893100000	1119700000	899580000	1193200000	943650000	994660000
sp|P23381	WARS	sp|P23381	1	16	16	16	48	53.165	1760100000	1996100000	1831300000	2387400000	2383200000	1948700000
sp|P23396	RPS3	sp|P23396	1	19	19	19	78	26.688	2.4891E+10	2.4967E+10	2.5093E+10	23858000000	25371000000	2.4262E+10
sp|P23434	GCSH	sp|P23434	1	4	4	4	56	18.884	0	682840000	501170000	474690000	629100000	639250000
sp|P23497	SP100	sp|P23497	1	4	2	1	4	100.42	84924000	90444000	100200000	91429000	82549000	0
sp|P23526	AHCY	sp|P23526	1	20	20	20	45	47.716	1.0735E+10	1.1512E+10	1.0352E+10	9686400000	10166000000	1.0092E+10
sp|P23528	CFL1	sp|P23528	1	15	15	12	74	18.502	6.1341E+10	6.1346E+10	6.8193E+10	55989000000	52619000000	5.4968E+10
sp|P23588	EIF4B	sp|P23588	1	16	16	16	35	69.15	4401200000	4773400000	4267700000	3387200000	3456500000	3516000000
sp|P23634	ATP2B4	sp|P23634	1	16	16	9	18	137.92	830420000	929610000	859660000	804370000	750690000	1016900000

sp|P23743	DGKA	sp|P23743	1	1	1	1	3	82.629	0	0	0	0	0	27986000
sp|P23786	CPT2	sp|P23786	1	9	9	9	20	73.776	240180000	197260000	208650000	200960000	232220000	204300000
sp|P23919	DTYMK	sp|P23919	1	10	10	10	45	23.819	1443900000	1323700000	1411100000	1202000000	1406300000	1534000000
sp|P23921	RRM1	sp|P23921	1	33	33	33	55	90.069	6612200000	7085900000	6531100000	5633700000	6067200000	5983500000
sp|P24386	CHM	sp|P24386	1	1	1	1	3.5	73.475	0	0	0	0	0	52228000
sp|P24390	KDELR1	sp|P24390	2	2	2	2	14	24.542	109720000	0	62656000	0	0	0
sp|P24534	EEF1B2	sp|P24534	1	10	8	8	64	24.763	1.3285E+10	9859200000	5545200000	10731000000	11102000000	9473600000
sp|P24539	ATP5F1	sp|P24539	1	9	9	9	35	28.908	5847400000	4157900000	4813200000	6756200000	6116200000	7200200000
sp|P24666	ACP1	sp|P24666	1	7	7	7	65	18.042	1613300000	1411800000	1217400000	1688700000	1802600000	1569900000
sp|P24752	ACAT1	sp|P24752	1	12	12	12	43	45.199	1309800000	1480000000	1367100000	1574300000	1586500000	1825600000
sp|P24844	MYL9	sp|P24844	1	8	3	3	62	19.827	332570000	367600000	544440000	451340000	321680000	344870000
sp|P24928	POLR2A	sp|P24928	1	8	8	8	8	217.17	351690000	267770000	363070000	230810000	321470000	272370000
sp|P24941	CDK2	sp|P24941	2	8	7	7	36	33.929	862340000	858690000	849290000	744540000	939360000	815110000
sp|P25205	MCM3	sp|P25205	1	35	35	35	55	90.98	5884800000	6241200000	5832600000	5774500000	5510400000	5157900000
sp|P25325	MPST	sp|P25325	1	6	6	6	35	33.178	522410000	368230000	460270000	542140000	357250000	377280000
sp|P25398	RPS12	sp|P25398	1	8	8	8	71	14.515	1.5958E+10	1.3419E+10	1.8667E+10	15195000000	19356000000	1.578E+10
sp|P25440	BRD2	sp|P25440	1	6	6	6	14	88.06	241810000	0	311880000	252590000	0	0
sp|P25685	DNAJB1	sp|P25685	1	14	14	13	49	38.044	2064100000	2059900000	1839700000	2178800000	2362600000	2375200000
sp|P25686	DNAJB2	sp|P25686	1	1	1	1	3.4	35.58	0	0	0	0	18226000	0
sp|P25705	ATP5F1A	sp|P25705	1	26	26	26	51	59.75	2.0758E+10	2.1994E+10	2.1588E+10	23453000000	25241000000	2.6528E+10
sp|P25786	PSMA1	sp|P25786	1	16	16	16	50	29.555	4227700000	4662200000	4968700000	4108400000	4152900000	3790000000
sp|P25787	PSMA2	sp|P25787	1	9	9	9	47	25.898	2407200000	3532100000	2633600000	2431500000	2131000000	2301000000
sp|P25788	PSMA3	sp|P25788	1	12	12	12	43	28.433	2790100000	2675400000	3134000000	3142900000	2787200000	2870500000
sp|P25789	PSMA4	sp|P25789	1	11	11	11	53	29.483	7028000000	6182100000	6007300000	5239200000	6447000000	5026800000
sp|P25815	S100P	sp|P25815	1	2	2	2	24	10.4	0	134070000	0	0	286350000	263480000
sp|P26006	ITGA3	sp|P26006	1	5	5	5	5.9	116.61	0	492290000	424130000	243800000	284710000	0
sp|P26038	MSN	sp|P26038	1	43	43	28	63	67.819	5.9503E+10	6.5256E+10	5.3495E+10	54865000000	54141000000	5.1556E+10
sp|P26196	DDX6	sp|P26196	1	13	13	13	37	54.416	1754900000	1436000000	1482000000	1393000000	1405600000	1429300000
sp|P26232	CTNNA2	sp|P26232	1	9	1	1	12	105.31	174130000	0	206130000	154330000	0	146360000
sp|P26358	DNMT1	sp|P26358	1	23	23	23	21	183.16	926250000	951850000	1088800000	819250000	1048000000	855900000
sp|P26368	U2AF2	sp|P26368	1	13	13	13	44	53.5	4123600000	4366400000	4161400000	4291700000	5287800000	3797200000
sp|P26373	RPL13	sp|P26373	1	9	9	9	42	24.261	1.1384E+10	1.0861E+10	9576800000	11312000000	12747000000	9322700000
sp|P26374	CHML	sp|P26374	1	1	1	1	2.9	74.071	0	0	0	0	0	35687000
sp|P26440	IVD	sp|P26440	1	2	2	2	5.9	46.319	0	0	0	0	0	153290000
sp|P26447	S100A4	sp|P26447	1	5	5	5	37	11.728	1.2187E+10	1.2491E+10	1.3265E+10	10807000000	11604000000	1.1301E+10
sp|P26583	HMGB2	sp|P26583	1	8	7	7	29	24.033	3370500000	2665400000	3760100000	3326500000	3837900000	3561400000
sp|P26599	PTBP1	sp|P26599	1	16	16	14	50	57.221	1.1161E+10	1.2342E+10	1.1164E+10	11557000000	11710000000	1.2619E+10
sp|P26639	TARS	sp|P26639	2	36	36	36	55	83.434	2.9403E+10	2.953E+10	2.6604E+10	32932000000	30635000000	2.8372E+10
sp|P26640	VARS	sp|P26640	1	37	37	37	40	140.47	5057200000	6082900000	4684700000	5063300000	5436400000	5444100000

sp|P26641	EEF1G	sp|P26641	1	22	22	22	52	50.118	2.4991E+10	2.6193E+10	2.4352E+10	26140000000	25579000000	2.424E+10
sp|P26885	FKBP2	sp|P26885	1	5	5	5	47	15.649	832580000	501470000	1271900000	1085200000	1436400000	1134500000
sp|P27105	STOM	sp|P27105	1	10	10	10	49	31.73	2810600000	3047000000	3727700000	2770500000	2972000000	2913400000
sp|P27144	AK4	sp|P27144	1	6	6	6	40	25.268	243100000	554770000	542620000	424580000	615390000	809080000
sp|P27348	YWHAQ	sp|P27348	1	14	10	10	54	27.764	2.0774E+10	1.6819E+10	1.9166E+10	17752000000	16400000000	1.6796E+10
sp|P27361	MAPK3	sp|P27361	1	5	2	2	19	43.135	0	0	0	0	0	73252000
sp|P27635	RPL10	sp|P27635	2	9	9	9	43	24.604	8724100000	1.0239E+10	9054200000	8456200000	9718000000	9303400000
sp|P27694	RPA1	sp|P27694	1	19	19	19	39	68.137	2826800000	2693400000	2596800000	2381600000	2580500000	2606800000
sp|P27695	APEX1	sp|P27695	1	15	15	15	55	35.554	5798000000	5113700000	6707800000	5229200000	5327900000	4589700000
sp|P27707	DCK	sp|P27707	1	5	5	5	24	30.518	231930000	98193000	273230000	168220000	265660000	185410000
sp|P27708	CAD	sp|P27708	1	49	46	46	32	242.98	5128900000	4413700000	4741600000	4595900000	4707800000	4797400000
sp|P27797	CALR	sp|P27797	1	20	20	20	62	48.141	2.5326E+10	3.2581E+10	2.6038E+10	32974000000	32151000000	3.5043E+10
sp|P27816	MAP4	sp|P27816	1	45	45	45	51	121	1.2051E+10	1.3078E+10	1.1969E+10	11195000000	11388000000	1.0969E+10
sp|P27824	CANX	sp|P27824	1	25	25	25	38	67.567	1.466E+10	1.6177E+10	1.5733E+10	19536000000	17429000000	1.7529E+10
sp|P28062	PSMB8	sp|P28062	1	3	3	3	15	30.354	0	253320000	0	0	0	123340000
sp|P28066	PSMA5	sp|P28066	1	12	12	12	61	26.411	5458700000	5598000000	5372200000	5515500000	6577000000	5498700000
sp|P28070	PSMB4	sp|P28070	1	7	7	7	46	29.204	1930600000	3022900000	2234300000	2302600000	2509600000	2420900000
sp|P28072	PSMB6	sp|P28072	1	6	6	6	41	25.357	1826200000	2770400000	2811300000	1898100000	1912800000	2410400000
sp|P28074	PSMB5	sp|P28074	1	11	11	11	49	28.48	4098100000	4557100000	3763100000	4333300000	3886400000	2959300000
sp|P28288	ABCD3	sp|P28288	1	7	7	7	15	75.475	533080000	624700000	546810000	752290000	591150000	639250000
sp|P28290	SSFA2	sp|P28290	1	6	6	6	9.4	138.38	0	116180000	135270000	0	114350000	108570000
sp|P28300	LOX	sp|P28300	1	1	1	1	3.4	46.944	0	0	0	19930000	0	0
sp|P28331	NDUFS1	sp|P28331	1	12	12	12	27	79.467	792790000	866770000	819540000	822720000	924840000	562700000
sp|P28340	POLD1	sp|P28340	1	15	15	15	19	123.63	656620000	783010000	652630000	755130000	737210000	603630000
sp|P28347	TEAD1	sp|P28347	4	2	2	2	8.5	47.945	59934000	0	0	0	0	0
sp|P28482	MAPK1	sp|P28482	1	13	13	10	51	41.389	1281400000	1483600000	1504700000	1427300000	1566700000	1686300000
sp|P28838	LAP3	sp|P28838	1	16	16	16	42	56.166	1488000000	1781600000	1696300000	1664000000	1487100000	1774200000
sp|P29083	GTF2E1	sp|P29083	1	2	2	2	6.2	49.452	0	0	0	0	0	44483000
sp|P29084	GTF2E2	sp|P29084	1	2	2	2	12	33.043	0	0	0	0	0	40592000
sp|P29144	TPP2	sp|P29144	1	27	27	27	29	138.35	1689000000	1817100000	1825800000	1486300000	1674600000	1632500000
sp|P29218	IMPA1	sp|P29218	1	4	4	4	22	30.188	241510000	184950000	0	0	162610000	0
sp|P29317	EPHA2	sp|P29317	6	14	14	13	18	108.27	482990000	502130000	587030000	694480000	878120000	987060000
sp|P29353	SHC1	sp|P29353	1	6	6	6	18	62.821	359820000	397000000	394730000	428260000	342880000	387640000
sp|P29372	MPG	sp|P29372	1	3	3	3	15	32.868	111970000	107990000	0	141180000	129440000	123350000
sp|P29401	TKT	sp|P29401	1	32	32	32	68	67.877	9.6761E+10	9.7446E+10	8.503E+10	89271000000	83991000000	8.1966E+10
sp|P29558	RBMS1	sp|P29558	2	2	2	2	7.4	44.505	394330000	343280000	0	0	371960000	312570000
sp|P29590	PML	sp|P29590	1	3	3	3	4.4	97.55	0	0	0	0	91296000	121410000
sp|P29692	EEF1D	sp|P29692	1	16	16	14	64	31.121	1.9585E+10	1.712E+10	1.9745E+10	17649000000	19624000000	1.9711E+10
sp|P29966	MARCKS	sp|P29966	1	8	8	8	48	31.554	5235600000	6870300000	4384700000	4825800000	5646800000	5241800000

sp|P29992	GNA11	sp|P29992	2	5	5	3	19	42.123	53805000	175100000	139720000	230000000	98492000	127780000
sp|P30040	ERP29	sp|P30040	1	10	10	10	46	28.993	3363500000	3673600000	3626600000	3401600000	3879900000	3698100000
sp|P30041	PRDX6	sp|P30041	1	17	17	17	70	25.035	2.1177E+10	2.392E+10	2.5211E+10	22676000000	21406000000	2.1297E+10
sp|P30043	BLVRB	sp|P30043	1	8	8	8	56	22.119	1384700000	1252100000	1244400000	1270500000	1258200000	1334800000
sp|P30044	PRDX5	sp|P30044	1	9	9	9	56	22.086	9054900000	8653900000	7764600000	9742500000	10429000000	7473500000
sp|P30046	DDT	sp|P30046	2	6	6	6	53	12.712	2209900000	1860500000	2304100000	1817000000	1606900000	1722500000
sp|P30048	PRDX3	sp|P30048	1	8	8	8	39	27.692	4539800000	8453400000	6665400000	6628700000	7453900000	6553500000
sp|P30049	ATP5F1D	sp|P30049	1	2	2	2	14	17.49	0	1495300000	0	1158000000	1193400000	1429300000
sp|P30050	RPL12	sp|P30050	1	7	7	7	59	17.818	1.456E+10	1.3344E+10	1.3637E+10	14695000000	13435000000	1.4961E+10
sp|P30084	ECHS1	sp|P30084	1	11	11	11	52	31.387	2868200000	2505800000	2521400000	3881700000	3892400000	3086100000
sp|P30085	CMPK1	sp|P30085	1	9	9	9	54	22.222	2329900000	2883800000	2984400000	2598600000	2675200000	2637000000
sp|P30086	PEBP1	sp|P30086	1	9	9	9	73	21.057	1.1245E+10	1.2426E+10	1.0237E+10	8247400000	10116000000	1.0012E+10
sp|P30101	PDIA3	sp|P30101	1	30	30	30	63	56.782	2.6141E+10	2.9172E+10	2.7947E+10	28750000000	26812000000	2.9498E+10
sp|P30153	PPP2R1A	sp|P30153	1	24	24	19	50	65.308	8456000000	9134700000	8961700000	8624800000	9303800000	7190300000
sp|P30154	PPP2R1B	sp|P30154	1	8	3	3	19	66.213	0	0	0	0	123580000	0
sp|P30260	CDC27	sp|P30260	1	5	5	5	6.6	91.866	136680000	140200000	0	150800000	157570000	0
sp|P30405	PPIF	sp|P30405	1	4	3	3	34	22.04	0	0	0	246930000	0	0
sp|P30408	TM4SF1	sp|P30408	1	1	1	1	4.5	21.632	1176400000	1489600000	1264200000	1482200000	1376100000	1577900000
sp|P30419	NMT1	sp|P30419	2	11	11	11	29	56.806	2277200000	1942200000	1932200000	2012700000	2143600000	1740200000
sp|P30453	HLA-A	sp|P30453	5	11	1	0	34	41.054	0	0	0	0	0	683020000
sp|P30685	HLA-B	sp|P30685	6	11	3	0	40	40.455	321700000	312990000	332290000	374040000	376840000	423920000
sp|P30519	HMOX2	sp|P30519	1	6	6	6	29	36.032	486800000	553280000	520010000	590710000	450830000	704030000
sp|P30520	ADSS	sp|P30520	1	12	12	12	34	50.097	2049500000	2254900000	2140800000	2290700000	2651800000	2264300000
sp|P30530	AXL	sp|P30530	2	6	6	6	9.4	98.336	151880000	183750000	126170000	119060000	121610000	102110000
sp|P30533	LRPAP1	sp|P30533	1	7	7	7	21	41.465	590710000	462830000	378510000	585160000	502100000	427300000
sp|P30536	TSPO	sp|P30536	1	2	2	2	9.5	18.828	600170000	270870000	528440000	0	0	781520000
sp|P30566	ADSL	sp|P30566	1	14	14	14	41	54.889	2063400000	2620600000	1770300000	1656200000	1768300000	1539100000
sp|P30622	CLIP1	sp|P30622	2	7	7	7	6.5	162.24	277960000	353570000	286880000	298440000	309880000	219850000
sp|P30626	SRI	sp|P30626	1	10	10	10	56	21.676	2294000000	2750300000	2323000000	2072500000	2317500000	2246000000
sp|P30740	SERPINB	sp|P30740	1	4	4	4	13	42.741	398450000	351700000	307830000	362070000	364810000	429850000
sp|P30825	SLC7A1	sp|P30825	1	1	1	1	1.7	67.638	0	0	0	29223000	0	0
sp|P30837	ALDH1B1	sp|P30837	3	18	18	17	36	57.206	3404800000	4748300000	5273900000	3718500000	3864500000	4226800000
sp|P30838	ALDH3A1	sp|P30838	3	9	8	8	28	50.394	718190000	1045200000	878280000	842840000	693160000	723540000
sp|P30876	POLR2B	sp|P30876	1	16	16	16	17	133.9	1046000000	1263300000	1179900000	880550000	997430000	1110500000
sp|P31040	SDHA	sp|P31040	1	26	26	26	62	72.691	9300300000	9750000000	8187300000	7201100000	7469600000	7709200000
sp|P31150	GDI1	sp|P31150	1	16	9	9	53	50.582	1628700000	1146800000	2148600000	1516600000	1627900000	1443300000
sp|P31153	MAT2A	sp|P31153	2	13	13	13	42	43.66	4172400000	4267700000	4829800000	4357700000	4701500000	4257700000
sp|P31327	CPS1	sp|P31327	1	75	75	72	62	164.94	2.323E+10	2.3329E+10	2.4291E+10	27560000000	28677000000	2.7602E+10
sp|P31350	RRM2	sp|P31350	2	11	11	11	38	44.877	1233600000	1133200000	1269000000	1027100000	977870000	883650000

sp|P31689	DNAJA1	sp|P31689	1	16	16	16	53	44.868	7693900000	7108400000	5948000000	6392900000	6525600000	5663800000
sp|P31751	AKT2	sp|P31751	1	3	3	2	12	55.768	105060000	148490000	110080000	128440000	153140000	140340000
sp|P31930	UQCRC1	sp|P31930	1	16	15	15	47	52.645	5590900000	5391900000	4869700000	5689400000	5369700000	6132900000
sp|P31937	HIBADH	sp|P31937	1	9	9	9	44	35.329	622780000	702600000	602870000	737360000	855220000	814440000
sp|P31939	ATIC	sp|P31939	1	35	35	35	75	64.615	1.2312E+10	1.3453E+10	1.2004E+10	11875000000	12679000000	1.1598E+10
sp|P31942	HNRNPH3	sp|P31942	1	14	13	13	62	36.926	4355500000	3550000000	3046300000	4211900000	4308400000	4655200000
sp|P31943	HNRNPH1	sp|P31943	1	17	17	11	52	49.229	1.7908E+10	1.9162E+10	2.21E+10	19602000000	18255000000	1.8328E+10
sp|P31946	YWHAB	sp|P31946	1	13	9	8	63	28.082	1.2066E+10	1.316E+10	1.3382E+10	16013000000	14372000000	1.4852E+10
sp|P31947	SFN	sp|P31947	1	14	12	12	62	27.774	7737500000	7021900000	7544200000	8696800000	9164800000	7176200000
sp|P31948	STIP1	sp|P31948	1	36	36	36	57	62.639	1.9674E+10	2.1592E+10	1.7952E+10	18378000000	20832000000	2.1608E+10
sp|P31949	S100A11	sp|P31949	1	6	6	6	53	11.74	6568300000	9095800000	7862800000	8400600000	9049200000	7293700000
sp|P32004	L1CAM	sp|P32004	1	23	23	23	25	140	2798000000	2662300000	2704400000	2280500000	2238300000	2267000000
sp|P32119	PRDX2	sp|P32119	1	10	9	9	54	21.892	1.0952E+10	1.3792E+10	1.1702E+10	12239000000	12061000000	9817100000
sp|P32320	CDA	sp|P32320	1	4	4	4	55	16.185	2025100000	1815700000	1581600000	1729300000	2142000000	1657700000
sp|P32321	DCTD	sp|P32321	1	1	1	1	10	20.016	0	0	0	0	0	32887000
sp|P32322	PYCR1	sp|P32322	1	10	10	9	47	33.36	1088800000	1077200000	864590000	1043100000	979060000	1076400000
sp|P32455	GBP1	sp|P32455	2	6	6	4	15	67.93	424680000	799960000	674050000	692160000	603040000	690190000
sp|P32456	GBP2	sp|P32456	1	5	3	3	9.3	67.208	0	0	0	0	0	118300000
sp|P32780	GTF2H1	sp|P32780	1	3	3	3	6.6	62.031	0	45830000	0	0	0	0
sp|P32969	RPL9	sp|P32969	1	9	9	9	54	21.863	6440800000	6269300000	6688500000	7805500000	7610500000	6355100000
sp|P32970	CD70	sp|P32970	1	5	5	5	33	21.118	172710000	245930000	178180000	150030000	136300000	141080000
sp|P33121	ACSL1	sp|P33121	1	4	4	4	7.2	77.942	0	121140000	114510000	103850000	0	96063000
sp|P33176	KIF5B	sp|P33176	3	35	35	35	49	109.68	4457300000	4629200000	4187300000	3934900000	4202900000	4440300000
sp|P33240	CSTF2	sp|P33240	1	9	9	4	26	60.959	742850000	825870000	819340000	814740000	805140000	721180000
sp|P33316	DUT	sp|P33316	1	10	10	10	50	26.563	7316300000	6833800000	6959900000	7129600000	7208400000	7377400000
sp|P33527	ABCC1	sp|P33527	1	15	15	15	15	171.59	704060000	801200000	648780000	705400000	843420000	788840000
sp|P33552	CKS2	sp|P33552	1	2	2	2	24	9.8602	270170000	253550000	199470000	213550000	192920000	256500000
sp|P33897	ABCD1	sp|P33897	2	7	7	7	12	82.936	427440000	367800000	457130000	363260000	494510000	648890000
sp|P33991	MCM4	sp|P33991	2	29	29	29	41	96.557	4334600000	4523400000	3610500000	3764500000	3554700000	3990400000
sp|P33992	MCM5	sp|P33992	1	30	30	30	47	82.285	4560000000	4698400000	4682300000	4510400000	5032100000	4790600000
sp|P33993	MCM7	sp|P33993	1	26	26	26	44	81.307	4385800000	3938600000	4167500000	4456200000	3874100000	4291200000
sp|P34059	GALNS	sp|P34059	1	1	1	1	3.1	58.025	0	0	0	0	37342000	0
sp|P34741	SDC2	sp|P34741	1	1	1	1	7.5	22.16	0	0	0	0	0	0
sp|P34896	SHMT1	sp|P34896	1	10	9	9	32	53.082	643420000	656850000	673030000	458770000	394880000	427730000
sp|P34897	SHMT2	sp|P34897	1	18	18	17	52	55.992	5251400000	5798600000	5355900000	6165300000	6020500000	5919800000
sp|P34932	HSPA4	sp|P34932	1	45	45	43	70	94.33	1.8235E+10	1.8071E+10	1.7959E+10	19023000000	18844000000	1.7239E+10
sp|P43250	GRK6	sp|P43250	2	1	1	1	2.4	65.99	0	0	0	0	8441900	0
sp|P34949	MPI	sp|P34949	1	3	3	3	13	46.655	0	0	0	0	123540000	86550000
sp|P35052	GPC1	sp|P35052	1	4	4	4	12	61.68	0	0	0	158330000	214170000	233670000

sp|P3508(	PFN2	sp|P3508(	1	4	4	4	49	15.046	873750000	0	802060000	805070000	984340000	0
sp|P35221	CTNNA1	sp|P35221	2	40	40	32	62	100.07	6008200000	5903600000	6838900000	4986800000	5034500000	5192900000
sp|P3522	CTNNB1	sp|P3522	1	20	15	15	36	85.496	889560000	1151600000	940790000	933400000	866630000	934490000
sp|P3523	PHB	sp|P3523	1	15	15	15	56	29.804	6435300000	6722000000	6122100000	7303400000	8558000000	7705700000
sp|P3523	SERPINB	sp|P3523	1	12	12	11	43	42.621	3177100000	2467100000	3734800000	3483300000	3579900000	3216000000
sp|P35241	RDX	sp|P35241	1	31	15	15	46	68.563	3266200000	3500200000	3952200000	3251800000	3108200000	2667900000
sp|P3524	RPA3	sp|P3524	1	5	5	5	52	13.569	1007400000	1031400000	1160300000	1015300000	1242000000	849620000
sp|P3524	RFC4	sp|P3524	1	8	8	8	32	39.681	865280000	1026600000	1166400000	988400000	806830000	756930000
sp|P3525(	RFC2	sp|P3525(	1	8	8	8	29	39.157	945220000	1242200000	829950000	1107500000	941290000	941060000
sp|P35251	RFC1	sp|P35251	1	10	10	10	11	128.25	308740000	361310000	332380000	360150000	254410000	274880000
sp|P3526	RPL22	sp|P3526	1	5	5	5	52	14.787	5506600000	4925700000	1.0927E+10	5665900000	4719700000	6728700000
sp|P3526	GTF2F1	sp|P3526	1	7	7	7	22	58.24	571070000	622350000	530030000	525360000	398790000	492140000
sp|P3527(	SPR	sp|P3527(	1	5	5	5	28	28.048	369550000	413730000	388430000	693430000	474950000	470020000
sp|P3555	FBN1	sp|P3555	1	2	2	2	0.8	312.24	0	0	0	0	0	19334000
sp|P3557	AGL	sp|P3557	1	12	12	12	11	174.76	522930000	604360000	499730000	516800000	571320000	553680000
sp|P3557	MYH9	sp|P3557	9	##	122	##	59	226.53	1.003E+11	1.039E+11	1.0154E+11	1.0216E+11	1.0782E+11	1.0594E+11
sp|P3558(	MYH10	sp|P3558(	1	40	24	22	26	229	803630000	881050000	982830000	908130000	988580000	1050500000
sp|P3560	COPB2	sp|P3560	1	26	26	26	41	102.49	5245600000	4250600000	4614300000	4076700000	4527600000	4719900000
sp|P3561(	SOAT1	sp|P3561(	1	4	4	4	11	64.734	96484000	167800000	112490000	129990000	56347000	0
sp|P35611	ADD1	sp|P35611	1	5	5	5	11	80.954	230590000	241480000	234910000	157280000	163970000	195960000
sp|P3561	BSG	sp|P3561	1	10	10	10	34	42.2	6654800000	6762400000	7923400000	7646900000	9330000000	7418500000
sp|P3563	FUS	sp|P3563	1	9	9	7	22	53.425	7755500000	7504800000	8664600000	8805700000	8301800000	9665900000
sp|P3565	NUP214	sp|P3565	1	26	26	26	19	213.62	1580300000	1454300000	1910200000	1638800000	1487700000	1643500000
sp|P3565	DEK	sp|P3565	1	10	10	10	33	42.674	1972000000	2295600000	1685300000	1842800000	2090400000	1988000000
sp|P3575	GLRX	sp|P3575	1	4	4	4	39	11.776	548640000	485930000	482320000	517710000	529130000	411210000
sp|P35914	HMGCL	sp|P35914	1	2	2	2	8	34.36	0	0	0	123330000	0	0
sp|P3599	PSMC2	sp|P3599	1	24	24	24	61	48.633	4323400000	5083800000	4124200000	4484700000	4680500000	4268600000
sp|P3640	ARL2	sp|P3640	1	5	5	5	33	20.878	0	180290000	178470000	181890000	230790000	0
sp|P3640	ARL3	sp|P3640	1	8	8	8	54	20.455	750420000	877900000	681910000	847960000	697560000	607890000
sp|P3650	MAP2K2	sp|P3650	1	9	9	7	35	44.424	706870000	993950000	926750000	980190000	1128100000	932000000
sp|P3654	ATP5F1C	sp|P3654	1	10	10	10	35	32.996	3686400000	3845200000	3964200000	4343300000	4108700000	5060200000
sp|P3654	ATP6V1E	sp|P3654	1	3	3	3	16	26.145	218490000	219430000	200350000	264580000	190660000	235240000
sp|P36551	CPOX	sp|P36551	1	4	4	4	12	50.151	0	91882000	0	0	0	0
sp|P3657	RPL4	sp|P3657	1	19	19	19	48	47.697	2.0592E+10	2.4728E+10	1.7535E+10	21475000000	25192000000	2.0536E+10
sp|P3663	NUDT1	sp|P3663	1	3	3	3	20	22.519	394280000	504760000	441860000	414180000	598700000	244750000
sp|P3677	LONP1	sp|P3677	1	22	22	22	35	106.49	2367700000	2454800000	2603200000	3649200000	2845500000	3066900000
sp|P36871	PGM1	sp|P36871	1	24	24	24	56	61.448	3015100000	3961400000	3457000000	3493000000	3712500000	3096700000
sp|P3687	PPP1CC	sp|P3687	1	12	1	1	43	36.983	0	0	0	0	0	71956000
sp|P3691	GNL1	sp|P3691	1	1	1	1	3	68.66	0	0	0	0	0	41757000

sp|P36952	SERPINB5	sp|P36952	1	21	21	21	73	42.1	1.1497E+10	1.1558E+10	1.268E+10	10881000000	10960000000	1.0513E+10
sp|P36954	POLR2I	sp|P36954	1	3	3	3	42	14.523	160080000	134320000	137530000	135500000	167390000	130740000
sp|P36957	DLST	sp|P36957	1	12	12	12	37	48.755	1949900000	2127900000	2337100000	2538100000	2583500000	2563900000
sp|P36969	GPX4	sp|P36969	1	2	2	2	13	22.174	0	156920000	0	0	0	0
sp|P37108	SRP14	sp|P37108	1	7	7	7	60	14.57	5835400000	6010200000	6936800000	5093300000	5823100000	4510100000
sp|P37198	NUP62	sp|P37198	1	8	8	8	22	53.254	982760000	1107100000	902090000	944780000	1071000000	1112000000
sp|P37235	HPCAL1	sp|P37235	3	11	11	11	60	22.313	1333500000	1613200000	1084200000	1084600000	1449800000	1050700000
sp|P37268	FDFT1	sp|P37268	1	7	7	7	22	48.115	382450000	502350000	443970000	0	154600000	158940000
sp|P37802	TAGLN2	sp|P37802	1	16	16	15	88	22.391	3.0385E+10	3.7217E+10	3.1584E+10	34085000000	34597000000	2.9097E+10
sp|P37837	TALDO1	sp|P37837	1	17	17	17	43	37.54	5578600000	6413200000	6192700000	6075600000	5512500000	5490600000
sp|P37840	SNCA	sp|P37840	1	3	3	3	35	14.46	348980000	286310000	266130000	378920000	429270000	349040000
sp|P38117	ETFB	sp|P38117	1	14	14	14	50	27.843	2971300000	3011800000	2852300000	3465800000	3389000000	3541800000
sp|P38159	RBMX	sp|P38159	5	17	17	17	42	42.331	9141500000	1.0505E+10	8864600000	9039400000	8529400000	9349700000
sp|P38432	COIL	sp|P38432	1	3	3	3	8.9	62.608	52126000	0	0	68197000	59157000	0
sp|P38606	ATP6V1A	sp|P38606	1	17	17	17	33	68.303	2382600000	2593500000	2800100000	2051500000	2323200000	2598800000
sp|P38646	HSPA9	sp|P38646	1	37	37	36	61	73.68	3.2234E+10	3.6835E+10	3.3076E+10	39351000000	40436000000	4.0177E+10
sp|P38919	EIF4A3	sp|P38919	1	23	19	19	62	46.871	4947900000	5467500000	5669400000	5817300000	6598600000	6135700000
sp|P39019	RPS19	sp|P39019	1	11	11	11	57	16.06	1.311E+10	1.0123E+10	1.171E+10	10892000000	12082000000	1.1752E+10
sp|P39023	RPL3	sp|P39023	2	22	22	22	46	46.108	1.9327E+10	1.8337E+10	1.9044E+10	19011000000	20144000000	1.9125E+10
sp|P39656	DDOST	sp|P39656	1	12	12	12	44	50.8	1537800000	1430400000	1863100000	1900100000	1511300000	1473000000
sp|P39687	ANP32A	sp|P39687	3	11	11	9	40	28.585	6955400000	7894200000	5909800000	6105300000	6784300000	5940200000
sp|P39748	FEN1	sp|P39748	3	12	12	12	43	42.592	4777200000	5371800000	4856000000	4580500000	4173900000	4501400000
sp|Q13948	CUX1	sp|Q13948	2	4	4	4	11	77.454	0	69214000	82427000	0	82998000	67191000
sp|P40121	CAPG	sp|P40121	1	11	11	11	46	38.498	3632300000	3458200000	3973000000	3383400000	3697500000	3484500000
sp|P40123	CAP2	sp|P40123	1	6	5	5	15	52.823	208730000	188910000	268310000	0	239270000	206250000
sp|P40222	TXLNA	sp|P40222	1	14	14	14	32	61.89	897440000	773300000	1075100000	855170000	964790000	1106200000
sp|P40227	CCT6A	sp|P40227	2	25	25	25	66	58.024	1.5584E+10	1.4941E+10	1.4168E+10	15014000000	16331000000	1.5402E+10
sp|P40261	NNMT	sp|P40261	1	8	8	8	42	29.574	1584900000	1736000000	1441000000	1702000000	1661200000	1572300000
sp|P40424	PBX1	sp|P40424	2	2	2	2	6.7	46.625	0	98982000	0	0	0	0
sp|P40429	RPL13A	sp|P40429	2	8	8	8	38	23.577	3782600000	4068100000	3970000000	4728100000	5327000000	3611000000
sp|P40616	ARL1	sp|P40616	1	4	4	4	39	20.417	333940000	421230000	319130000	417080000	445190000	509910000
sp|P40692	MLH1	sp|P40692	1	5	5	5	9.7	84.6	166880000	128260000	107210000	104500000	58704000	0
sp|P40763	STAT3	sp|P40763	1	21	21	21	39	88.067	1652300000	2100300000	1861600000	1610600000	1704900000	1627200000
sp|P40818	USP8	sp|P40818	1	3	3	3	4.7	127.52	239210000	178520000	221460000	196810000	166180000	82085000
sp|P40855	PEX19	sp|P40855	1	4	4	4	20	32.806	120100000	0	0	0	100320000	0
sp|P40925	MDH1	sp|P40925	1	13	13	13	43	36.426	8217400000	7482300000	8161400000	7388600000	7351500000	7751300000
sp|P40926	MDH2	sp|P40926	1	15	15	15	57	35.503	1.7889E+10	1.8918E+10	1.7919E+10	20218000000	23864000000	2.1752E+10
sp|P40937	RFC5	sp|P40937	1	6	6	6	19	38.496	588740000	653690000	530750000	529100000	590730000	492970000
sp|P40938	RFC3	sp|P40938	1	9	9	9	38	40.556	1020600000	1211200000	1028500000	1095300000	1100400000	1052000000

sp|P40939	HADHA	sp|P40939	1	27	27	27	50	82.999	5334200000	6214500000	5942900000	6862200000	6519300000	8052700000
sp|P41091	EIF2S3	sp|P41091	2	24	24	24	60	51.109	1.0713E+10	9638500000	1.0668E+10	9140800000	8940300000	1.0319E+10
sp|P41208	CETN2	sp|P41208	1	3	3	3	22	19.738	0	0	0	0	76806000	46304000
sp|P41214	EIF2D	sp|P41214	1	5	5	5	14	64.706	136290000	201360000	78608000	147510000	193080000	134640000
sp|P41221	WNT5A	sp|P41221	2	3	3	3	13	42.339	0	0	41032000	0	0	0
sp|P41223	BUD31	sp|P41223	1	7	7	7	46	17	414710000	393880000	414600000	454460000	481550000	338790000
sp|P41227	NAA10	sp|P41227	2	12	12	12	58	26.458	1581400000	1754500000	2011500000	1810300000	2040000000	1779700000
sp|P41236	PPP1R2	sp|P41236	2	5	5	5	37	23.015	0	0	146560000	167560000	158150000	206940000
sp|P41240	CSK	sp|P41240	1	4	4	4	13	50.704	77930000	0	0	0	114240000	114950000
sp|P41250	GARS	sp|P41250	1	25	25	25	40	83.165	3986700000	4256700000	4215500000	4440200000	4467900000	5219200000
sp|P41252	IARS	sp|P41252	1	41	41	41	41	144.5	6448700000	6295600000	6580700000	6930400000	6952700000	6985500000
sp|P41567	EIF1	sp|P41567	1	6	6	2	65	12.732	1232900000	1425300000	1124500000	1397600000	1691800000	1349500000
sp|P41743	PRKCI	sp|P41743	1	6	6	6	18	68.262	165530000	192830000	212550000	220530000	253100000	197300000
sp|P42025	ACTR1B	sp|P42025	1	7	2	2	20	42.293	0	0	0	0	0	91650000
sp|P42126	ECI1	sp|P42126	1	5	5	5	18	32.816	818360000	924940000	675770000	778050000	1174400000	1093000000
sp|P42166	TMPO	sp|P42166	1	20	20	13	43	75.491	3865200000	4229000000	3701700000	4918200000	5240800000	5018100000
sp|P42167	TMPO	sp|P42167	1	16	9	9	53	50.67	1651300000	1511300000	1279200000	1632100000	1547700000	1541200000
sp|P42224	STAT1	sp|P42224	1	20	20	20	33	87.334	2158300000	2540000000	2565800000	2270400000	2528900000	2387300000
sp|P42285	MTREX	sp|P42285	1	20	20	20	25	117.8	2077000000	2580800000	2152300000	1820900000	1984800000	1710100000
sp|P42330	AKR1C3	sp|P42330	1	16	9	9	69	36.853	1.6266E+10	1.654E+10	1.284E+10	12180000000	13640000000	1.17E+10
sp|P42345	MTOR	sp|P42345	1	16	16	16	8.6	288.89	1442000000	1658700000	1456600000	1473600000	1489400000	1527100000
sp|P42566	EPS15	sp|P42566	1	6	6	6	9.7	98.655	182260000	198930000	148320000	175680000	185830000	178260000
sp|P42574	CASP3	sp|P42574	1	2	2	2	6.9	31.608	24200000	0	0	0	72886000	0
sp|P42677	RPS27	sp|P42677	1	5	5	2	41	9.461	4662300000	6887200000	4968500000	4570900000	5900100000	6361700000
sp|P42695	NCAPD3	sp|P42695	1	4	4	4	3.7	168.89	5069000000	5795000000	0	0	0	3800400000
sp|P42696	RBM34	sp|P42696	1	3	3	3	7.2	48.564	0	158390000	0	84398000	65822000	0
sp|P42704	LRPPRC	sp|P42704	2	60	60	60	52	157.9	1.1768E+10	1.2274E+10	1.0502E+10	11288000000	11163000000	1.1589E+10
sp|P42765	ACAA2	sp|P42765	1	17	17	17	62	41.924	2330200000	2217400000	2446400000	2959000000	3611200000	3015700000
sp|P42766	RPL35	sp|P42766	1	3	3	3	26	14.551	4566000000	3338400000	3251700000	3462200000	3854300000	4110000000
sp|P42771	CDKN2A	sp|P42771	2	5	5	5	55	16.532	4125000000	4041600000	3618300000	3338700000	3563700000	2690900000
sp|P42773	CDKN2C	sp|P42773	1	3	3	3	19	18.127	175890000	228610000	237960000	0	178060000	0
sp|P42785	PRCP	sp|P42785	1	2	2	2	5.4	55.799	0	0	0	0	96901000	0
sp|P42858	HTT	sp|P42858	1	4	4	4	2.1	347.6	93579000	0	0	0	0	0
sp|P42892	ECE1	sp|P42892	1	15	15	15	30	87.163	910360000	1106800000	1043700000	904260000	859040000	1209000000
sp|P43007	SLC1A4	sp|P43007	1	3	3	3	9.8	55.722	121720000	146170000	110950000	94821000	129850000	178960000
sp|P43034	PAFAH1B	sp|P43034	1	14	14	14	44	46.637	2297300000	2255700000	2424200000	2070100000	2689700000	2809600000
sp|P43121	MCAM	sp|P43121	1	14	14	14	31	71.607	811750000	770420000	971700000	855130000	864660000	966400000
sp|P43243	MATR3	sp|P43243	1	31	31	31	45	94.622	1.4701E+10	1.3466E+10	1.4839E+10	14804000000	14912000000	1.4087E+10
sp|P43246	MSH2	sp|P43246	1	14	14	14	23	104.74	880290000	825990000	796410000	986610000	991450000	1001100000

sp|P43304	GPD2	sp|P43304	1	21	21	21	37	80.852	1901400000	1670800000	1980900000	1953300000	2057700000	2207900000
sp|P43307	SSR1	sp|P43307	1	4	4	4	24	32.235	728820000	725100000	815270000	842820000	875150000	762550000
sp|P43487	RANBP1	sp|P43487	2	7	7	7	58	23.31	4973600000	4805700000	5547700000	5645600000	4768800000	4641200000
sp|P43490	NAMPT	sp|P43490	1	17	17	17	51	55.52	1488700000	1426700000	1406700000	1563800000	1745400000	1303900000
sp|P43686	PSMC4	sp|P43686	1	19	19	19	65	47.366	2915500000	3707300000	2858400000	2766600000	3175300000	3050800000
sp|P43897	TSFM	sp|P43897	1	10	10	10	48	35.39	1201600000	1041300000	1146000000	1301100000	1221900000	1075600000
sp|P45877	PPIC	sp|P45877	1	3	3	3	15	22.763	200700000	274540000	262050000	346990000	271430000	257360000
sp|P45880	VDAC2	sp|P45880	1	14	14	14	64	31.566	8391400000	6646400000	7835600000	9540600000	10031000000	1.1213E+10
sp|P45973	CBX5	sp|P45973	1	8	8	8	47	22.225	1826700000	1999200000	1890500000	1268500000	1624800000	1706000000
sp|P45974	USP5	sp|P45974	1	22	22	22	38	95.785	4207600000	4557600000	3732500000	3475500000	3707400000	4110600000
sp|P46013	MKI67	sp|P46013	1	57	57	57	29	358.69	2918400000	3260600000	3153300000	2928500000	3145200000	3512300000
sp|P46060	RANGAP1	sp|P46060	1	21	21	21	44	63.541	3497900000	3546400000	3415300000	3526600000	3832700000	3429600000
sp|P46063	RECQL	sp|P46063	1	20	20	20	39	73.457	3553700000	3256900000	3474000000	3540200000	3105100000	3375800000
sp|P46087	NOP2	sp|P46087	1	17	17	17	28	89.301	2571300000	2346900000	2544200000	2997700000	2673600000	3362000000
sp|P46100	ATRX	sp|P46100	1	3	3	3	1.8	282.58	108320000	92926000	93565000	0	0	0
sp|P46108	CRK	sp|P46108	1	8	8	8	37	33.83	436680000	374110000	423360000	538390000	502810000	520690000
sp|P46109	CRKL	sp|P46109	1	7	7	7	38	33.777	951520000	849060000	742190000	849840000	888130000	860330000
sp|P46199	MTIF2	sp|P46199	1	1	1	1	1.7	81.316	0	0	0	0	60681000	0
sp|P46379	BAG6	sp|P46379	1	17	17	17	27	119.41	920640000	1079200000	1011700000	942290000	996000000	1022000000
sp|P46459	NSF	sp|P46459	1	20	20	20	30	82.593	1206700000	1111200000	1135000000	1424000000	1590500000	1543300000
sp|P46734	MAP2K3	sp|P46734	2	7	7	7	28	39.318	403120000	327340000	271930000	534700000	394250000	469880000
sp|P46736	BRCC3	sp|P46736	1	5	5	5	21	36.072	214930000	187210000	183900000	147740000	171040000	0
sp|P46776	RPL27A	sp|P46776	1	6	6	6	35	16.561	6774800000	5311300000	7822300000	7121300000	5490700000	7130600000
sp|P46777	RPL5	sp|P46777	1	15	15	15	56	34.362	1.4877E+10	1.624E+10	1.6219E+10	13956000000	14243000000	1.5687E+10
sp|P46778	RPL21	sp|P46778	1	7	7	7	37	18.565	5738600000	6139800000	6651700000	6140800000	6388400000	7606300000
sp|P46779	RPL28	sp|P46779	1	7	7	7	40	15.747	6903300000	7520600000	5697500000	6079100000	6556400000	6706100000
sp|P46781	RPS9	sp|P46781	1	15	15	15	58	22.591	1.1472E+10	1.4149E+10	1.3301E+10	13401000000	12870000000	1.3461E+10
sp|P46782	RPS5	sp|P46782	1	12	12	12	55	22.876	1.04E+10	1.1518E+10	1.1404E+10	12428000000	12978000000	1.2766E+10
sp|P46783	RPS10	sp|P46783	2	8	7	7	44	18.898	4918400000	3454400000	4040500000	4205600000	3641200000	3922500000
sp|P46821	MAP1B	sp|P46821	2	60	60	60	38	270.63	5754400000	6180200000	5803700000	5500500000	5713500000	5910500000
sp|P46926	GNPDA1	sp|P46926	1	9	9	6	46	32.668	1172100000	1404600000	992440000	833970000	897520000	760080000
sp|P46934	NEDD4	sp|P46934	1	2	2	2	2	149.11	0	0	0	0	0	49726000
sp|P46937	YAP1	sp|P46937	1	9	9	9	35	54.461	301330000	307960000	371170000	348620000	344610000	277230000
sp|P46939	UTRN	sp|P46939	1	5	5	5	2.5	394.46	0	0	0	0	45403000	0
sp|P46940	IQGAP1	sp|P46940	1	69	69	66	54	189.25	4.4635E+10	4.2773E+10	4.6171E+10	43278000000	39644000000	4.3299E+10
sp|P46976	GYG1	sp|P46976	1	3	3	3	11	39.383	0	245190000	0	0	0	0
sp|P46977	STT3A	sp|P46977	1	12	12	12	17	80.529	1594200000	1280100000	1583700000	1462600000	1578700000	1698700000
sp|P47224	RABIF	sp|P47224	1	1	1	1	8.9	13.839	0	0	0	0	0	66954000
sp|P47712	PLA2G4A	sp|P47712	1	17	17	17	30	85.238	1237500000	1537100000	1817200000	1411500000	1486800000	1593700000

sp|P4775	CAPZA2	sp|P4775	1	12	10	10	64	32.949	2651000000	3465000000	3960100000	2777900000	3042500000	2675700000
sp|P4775	CAPZB	sp|P4775	1	17	17	17	61	31.35	6709200000	8742100000	6837700000	7688300000	7522200000	7818800000
sp|P4781	EIF1AX	sp|P4781	2	4	4	4	42	16.46	1528400000	1870000000	2256700000	1730900000	1630400000	1551300000
sp|P4789	QARS	sp|P4789	1	34	33	33	55	87.798	5251700000	5035800000	4767200000	5206900000	5152400000	5505000000
sp|P4791	RPL29	sp|P4791	1	2	2	2	15	17.752	4590900000	0	2694700000	2376300000	4949700000	6297300000
sp|P4798	UQCRFS1	sp|P4798	2	8	8	8	39	29.668	1221700000	1094700000	1098200000	1102700000	1410200000	964120000
sp|P4804	ATP5O	sp|P4804	1	9	9	9	58	23.277	3798300000	2922800000	2878300000	3698400000	4001800000	3838500000
sp|P4805	LIMS1	sp|P4805	2	9	9	9	35	37.251	1041200000	997060000	769020000	671220000	743380000	964170000
sp|P4806	GLIPR1	sp|P4806	1	2	2	2	8.6	30.366	0	0	2112200000	0	0	0
sp|P4814	PREP	sp|P4814	1	15	15	15	31	80.699	975270000	944810000	857890000	790280000	819990000	815220000
sp|P4816	ME1	sp|P4816	1	14	14	14	34	64.149	1060500000	1132000000	907630000	1111800000	1019400000	979760000
sp|P4842	PIP4K2A	sp|P4842	1	3	3	1	9.6	46.224	66896000	0	0	0	59077000	0
sp|P4844	ARCN1	sp|P4844	1	19	19	19	41	57.21	1825200000	2207500000	2130100000	1902400000	1944500000	2128500000
sp|P4844	LSS	sp|P4844	1	8	8	8	14	83.308	520530000	447180000	410310000	447470000	401990000	682210000
sp|P4850	GCLC	sp|P4850	1	10	10	10	22	72.765	380040000	440050000	455240000	501220000	465470000	471160000
sp|P4850	GCLM	sp|P4850	1	8	8	8	38	30.727	1695000000	2000800000	1568200000	2459500000	3220300000	2721700000
sp|P4850	CD151	sp|P4850	1	3	3	3	11	28.295	0	366600000	0	0	345370000	368720000
sp|P4855	PSMD8	sp|P4855	1	7	7	7	25	39.611	1446900000	1480800000	1180600000	1369200000	1372600000	1367600000
sp|P4863	PRRC2A	sp|P4863	1	8	7	7	6.6	228.86	177910000	152520000	132350000	160050000	139330000	150240000
sp|P4863	GSS	sp|P4863	1	13	13	13	34	52.384	1014500000	1023200000	1076900000	855100000	927910000	973160000
sp|P4864	CCT5	sp|P4864	1	36	36	36	72	59.67	1.6834E+10	1.7972E+10	1.6914E+10	15537000000	15466000000	1.7047E+10
sp|P4865	PTDSS1	sp|P4865	1	6	6	6	18	55.527	230160000	220130000	199360000	245430000	211770000	175670000
sp|P4868	NES	sp|P4868	1	3	3	3	2.5	177.44	0	13856000	0	0	0	101000000
sp|P4872	HSPA13	sp|P4872	1	6	6	6	19	51.927	142730000	215370000	145420000	187650000	173990000	175060000
sp|P4872	CSNK1A1	sp|P4872	2	6	6	6	23	38.914	340940000	385050000	264350000	316300000	351280000	387790000
sp|P4873	CSNK1D	sp|P4873	2	5	5	5	18	47.33	198530000	255210000	0	172320000	189750000	0
sp|P4873	IDH2	sp|P4873	1	4	4	4	11	50.909	151310000	152730000	176290000	181500000	267090000	152470000
sp|P4873	PITPNB	sp|P4873	1	12	12	11	53	31.54	1146100000	1476200000	1140200000	1321400000	1301800000	1497500000
sp|P4896	CD97	sp|P4896	2	10	10	10	18	91.868	1120300000	1243900000	1117100000	1448200000	1716700000	1279400000
sp|P4900	POLD2	sp|P4900	1	9	9	9	34	51.289	470540000	365780000	374670000	355060000	356510000	349420000
sp|P4900	MARCKSI	sp|P4900	1	2	2	2	14	19.529	495250000	0	0	164930000	0	342170000
sp|P4902	PXN	sp|P4902	1	13	13	13	36	64.505	795630000	782020000	539050000	576240000	665180000	718370000
sp|P4918	ALDH9A1	sp|P4918	1	15	15	15	35	53.801	1137100000	1525400000	1078700000	1163900000	1499400000	1306300000
sp|P4920	RPL34	sp|P4920	1	5	5	5	36	13.293	5056600000	5312400000	4901200000	5170400000	5812500000	5248500000
sp|P4924	RPIA	sp|P4924	1	1	1	1	5.1	33.269	0	0	0	0	0	22923000
sp|P4925	LMAN1	sp|P4925	1	13	13	13	35	57.548	4330000000	3812600000	4729800000	4480900000	4416200000	3959000000
sp|P4932	NASP	sp|P4932	1	23	23	23	49	85.237	5153100000	5403300000	5874600000	5181900000	5059200000	4088900000
sp|P4932	FASN	sp|P4932	1	87	87	87	47	273.42	3.3325E+10	3.3636E+10	3.3584E+10	27855000000	25900000000	2.7242E+10
sp|P4935	FNTA	sp|P4935	1	5	5	5	15	44.408	132470000	132430000	120100000	121950000	102270000	119570000

sp|P4935(	FNTB	sp|P4935(	1	1	1	1	3.7	48.773	0	0	0	0	0	50552000
sp|P4936(	DHPS	sp|P4936(	1	2	2	2	8.7	40.97	0	173000000	0	0	0	0
sp|P4936{	CCT3	sp|P4936{	1	35	35	35	65	60.533	2.4999E+10	2.7424E+10	2.5754E+10	23939000000	24959000000	2.3023E+10
sp|P4940(	MRPL19	sp|P4940(	1	5	5	5	17	33.535	181110000	209120000	184840000	220450000	205420000	171210000
sp|P4941	TUFM	sp|P4941	1	28	28	28	70	49.541	1.4662E+10	1.4006E+10	1.5977E+10	17288000000	16580000000	1.5897E+10
sp|P4941{	ALDH7A1	sp|P4941{	1	15	15	15	42	58.486	2617900000	2202900000	2226500000	2386700000	2509800000	2172000000
sp|P4944	INPP1	sp|P4944	1	1	1	1	3	43.998	0	0	0	0	0	24610000
sp|P4945	CENPF	sp|P4945	1	7	7	7	3	367.76	0	100310000	0	0	0	0
sp|P4945{	SRP9	sp|P4945{	1	4	4	4	43	10.112	3375000000	4069400000	3235000000	2744400000	3287900000	3564200000
sp|P4958	PCYT1A	sp|P4958	2	4	4	4	15	41.731	231050000	278210000	257320000	228620000	244750000	220330000
sp|P4958{	AARS	sp|P4958{	1	34	34	34	50	106.81	4528200000	5053900000	4704600000	6598200000	6214400000	6049500000
sp|P4958{	CARS	sp|P4958{	1	14	14	14	23	85.472	719160000	784860000	735430000	997830000	1030600000	957270000
sp|P4959(	HARS2	sp|P4959(	1	8	3	3	16	56.888	0	0	109390000	0	0	0
sp|P4959	SARS	sp|P4959	1	16	16	16	38	58.777	3970900000	3636600000	3095100000	3596900000	4255000000	3408200000
sp|P4964	PRIM1	sp|P4964	1	6	6	6	19	49.901	208300000	279280000	274470000	228020000	243950000	356520000
sp|P4964	PRIM2	sp|P4964	1	9	9	9	22	58.805	381080000	358330000	498170000	339820000	253820000	326850000
sp|P4971	CTCF	sp|P4971	1	3	3	3	7.6	82.785	0	0	0	0	0	216900000
sp|P4972(	PSMB3	sp|P4972(	1	7	7	7	42	22.949	1920300000	1936800000	2387500000	2144400000	1872000000	1613900000
sp|P4972	PSMB2	sp|P4972	1	6	6	6	32	22.836	1387700000	1603400000	1411200000	1410100000	1216500000	1174800000
sp|P4973(	MCM2	sp|P4973(	1	27	27	27	37	101.89	4468200000	4348800000	4161500000	3882100000	4212100000	3957200000
sp|P4974{	ACADVL	sp|P4974{	1	24	24	24	49	70.389	4828800000	4930300000	4662100000	5900800000	5881600000	5745200000
sp|P4975(	YLPM1	sp|P4975(	1	5	5	5	3.6	241.64	238280000	167820000	247560000	123850000	203070000	215880000
sp|P4975	TMED10	sp|P4975	1	7	7	7	35	24.976	2532300000	2409100000	2464000000	2352500000	2117500000	2672700000
sp|P4975(	RBM25	sp|P4975(	1	14	14	14	20	100.18	935440000	1414700000	1008500000	1222600000	1380000000	1318200000
sp|P4975	NUMB	sp|P4975	1	2	2	2	6.9	70.803	0	0	0	103880000	0	0
sp|P4977(	EIF2B2	sp|P4977(	1	8	8	8	29	38.989	615700000	468740000	501280000	504620000	610920000	658900000
sp|P4977	HINT1	sp|P4977	1	8	8	8	75	13.802	9450200000	8662000000	6640700000	6808300000	4982200000	5357500000
sp|P4979(	NUP153	sp|P4979(	1	15	15	15	17	153.94	687320000	720230000	644790000	617520000	588500000	673640000
sp|P4979	RANBP2	sp|P4979	3	69	69	52	30	358.2	4175800000	4286700000	4225500000	4292500000	4463500000	4101500000
sp|P4982	NDUFV1	sp|P4982	1	5	5	5	19	50.817	380430000	436710000	306480000	300110000	286510000	243270000
sp|P4984(	GSK3A	sp|P4984(	1	7	7	5	22	50.98	172830000	0	174250000	0	136400000	126700000
sp|P4984	GSK3B	sp|P4984	1	6	4	4	26	46.744	113980000	182900000	155680000	153060000	124450000	137460000
sp|P4990	NT5C2	sp|P4990	1	5	5	5	12	64.969	100770000	124990000	0	171560000	203010000	0
sp|P4990	SEPHS1	sp|P4990	1	6	6	6	26	42.91	397810000	591880000	258960000	402310000	407920000	355600000
sp|P4991	MTHFS	sp|P4991	1	2	2	2	16	23.255	62183000	100080000	0	0	80486000	64025000
sp|P4991	GMPS	sp|P4991	1	21	21	21	43	76.715	2096600000	3108800000	2629800000	2920400000	2477700000	2364100000
sp|P4991(	LIG3	sp|P4991(	1	5	5	5	5.1	112.91	125670000	127420000	150550000	144140000	159280000	163620000
sp|P4991	LIG4	sp|P4991	1	1	1	1	1.5	103.97	0	0	0	0	0	40707000
sp|P4995{	MRE11	sp|P4995{	1	9	9	9	16	80.592	477220000	477560000	518590000	456160000	600970000	613600000

sp|P5013	HNMT	sp|P5013	1	2	2	2	14	33.295	206790000	158090000	137340000	125410000	234650000	159340000
sp|P50151	GNG10	sp|P50151	1	1	1	1	22	7.2053	0	0	0	0	0	19369000
sp|P5021	IDH3A	sp|P5021	1	11	11	11	34	39.591	2152500000	2679400000	2341100000	2346400000	2312100000	2305400000
sp|P5023	CRIP1	sp|P5023	1	3	3	3	56	8.5328	6189000000	3647800000	2573800000	5271200000	4696500000	4917200000
sp|P5033	PPOX	sp|P5033	1	2	2	2	10	50.765	60955000	0	62978000	129460000	91226000	0
sp|P5039	GDI2	sp|P5039	1	27	27	20	69	50.663	1.3886E+10	1.4053E+10	1.3911E+10	12692000000	13489000000	1.3338E+10
sp|P5040	EMD	sp|P5040	1	13	13	13	65	28.994	3532700000	3264000000	2397200000	3493600000	4580800000	4101600000
sp|P5041	CPT1A	sp|P5041	1	20	20	20	29	88.367	2921000000	2466500000	2212900000	2407100000	2776400000	3293000000
sp|P5045	SERPINB	sp|P5045	1	3	2	2	8.6	42.766	0	30990000	0	0	0	0
sp|P5045	SERPINB	sp|P5045	1	1	1	1	2.9	42.403	0	0	0	0	62574000	0
sp|P5045	SERPINH	sp|P5045	1	19	19	19	48	46.44	2.1465E+10	2.0332E+10	2.0557E+10	22862000000	21304000000	2.3027E+10
sp|P5050	ST13	sp|P5050	3	10	10	10	28	41.331	7812600000	6678400000	6793200000	6408200000	6152900000	6402700000
sp|P5055	VASP	sp|P5055	1	10	10	10	36	39.829	1143700000	1006500000	1341200000	1009500000	1240300000	1212500000
sp|P5057	DNM2	sp|P5057	2	19	19	13	29	98.063	1311100000	1293100000	1358200000	1374200000	1263100000	1105700000
sp|P5057	METAP2	sp|P5057	1	14	14	14	35	52.891	2816600000	2831100000	2434300000	2098200000	2474900000	2905000000
sp|P5058	NUDT2	sp|P5058	1	4	4	4	46	16.829	364060000	469820000	309080000	299780000	400760000	448600000
sp|P5061	CDK7	sp|P5061	1	2	2	2	12	39.038	74968000	116950000	94153000	111670000	118790000	111430000
sp|P5074	KNTC1	sp|P5074	1	5	5	5	2.9	250.75	195620000	272200000	270190000	246880000	242990000	118670000
sp|P5075	CDK9	sp|P5075	1	5	5	5	17	42.777	0	0	0	163310000	0	0
sp|P50851	LRBA	sp|P50851	1	7	7	7	3.3	319.1	179060000	205070000	203620000	191710000	200820000	190580000
sp|P50897	PPT1	sp|P50897	1	7	7	7	31	34.193	1593900000	1428200000	1096700000	1090600000	1196500000	1157500000
sp|P50914	RPL14	sp|P50914	1	5	5	5	24	23.432	7139800000	7947900000	7143000000	9473100000	9904700000	7481500000
sp|P5099	CCT8	sp|P5099	1	36	36	36	72	59.62	2.5905E+10	2.2624E+10	2.6055E+10	26595000000	27463000000	2.3657E+10
sp|P50991	CCT4	sp|P50991	1	28	28	28	61	57.924	1.6649E+10	1.7044E+10	1.7438E+10	15918000000	17504000000	1.822E+10
sp|P5099	ATP1A2	sp|P5099	1	14	1	1	18	112.26	0	0	0	0	52004000	0
sp|P5099	ANXA11	sp|P5099	1	13	13	13	29	54.389	982810000	1190200000	992470000	946320000	999770000	1183400000
sp|P5100	PAPOLA	sp|P5100	3	7	7	7	12	82.842	203170000	206880000	198790000	168280000	166140000	188640000
sp|P51114	FXR1	sp|P51114	1	11	11	10	24	69.72	949230000	1211900000	1129300000	900230000	1086600000	998040000
sp|P5111	FXR2	sp|P5111	1	9	8	7	18	74.222	492920000	523460000	611590000	610400000	499970000	559590000
sp|P51148	RAB5C	sp|P51148	1	10	10	7	72	23.482	3185100000	2596100000	3144300000	3196900000	3099900000	3136600000
sp|P5114	RAB7A	sp|P5114	1	15	15	15	74	23.489	4150700000	4312600000	4253200000	4105300000	4826600000	4399100000
sp|P51151	RAB9A	sp|P51151	2	4	4	4	27	22.837	0	0	136440000	274560000	144140000	179050000
sp|P5115	RAB13	sp|P5115	1	4	2	2	20	22.774	167410000	205610000	223840000	170310000	0	0
sp|P5115	RAB27A	sp|P5115	1	1	1	1	4.1	24.868	0	0	0	0	0	40358000
sp|P51172	SCNN1D	sp|P51172	1	1	1	1	2.4	70.214	0	0	0	0	0	59125000
sp|P51398	DAP3	sp|P51398	1	9	9	9	31	45.566	657520000	759410000	841240000	568250000	633150000	639880000
sp|P51452	DUSP3	sp|P51452	1	4	4	4	29	20.478	289070000	152230000	226820000	208780000	190770000	173070000
sp|P51531	SMARCA2	sp|P51531	1	11	4	4	8.7	181.28	167630000	73023000	167880000	282050000	127650000	123630000
sp|P51532	SMARCA4	sp|P51532	1	16	16	9	12	184.64	2252900000	2081900000	2332400000	1967300000	2115000000	2301600000

sp|P51553	IDH3G	sp|P51553	1	7	7	7	32	42.794	669610000	783100000	609720000	888740000	942800000	708560000
sp|P51570	GALK1	sp|P51570	1	7	7	7	29	42.272	297920000	316110000	258590000	343040000	294890000	263400000
sp|P51571	SSR4	sp|P51571	1	5	5	5	36	18.998	2435200000	2391500000	2730500000	2766400000	2618700000	2485000000
sp|P51572	BCAP31	sp|P51572	1	9	9	9	32	27.991	2071500000	3117900000	2086600000	2127700000	2154500000	2080400000
sp|P51608	MECP2	sp|P51608	1	1	1	1	2.9	52.44	0	0	0	0	0	19579000
sp|P51610	HCFC1	sp|P51610	2	23	23	23	18	208.73	1598800000	1849300000	1528800000	1558400000	1581100000	1605600000
sp|P51636	CAV2	sp|P51636	1	2	2	2	21	18.291	146260000	0	52721000	0	0	0
sp|P51648	ALDH3A2	sp|P51648	1	13	13	12	29	54.847	1923800000	2408600000	1950000000	2135300000	2400300000	2108900000
sp|P51659	HSD17B4	sp|P51659	1	27	27	27	51	79.685	3859400000	4097000000	4089100000	4142900000	4985300000	4719000000
sp|P51665	PSMD7	sp|P51665	1	7	7	7	30	37.025	1464300000	1710600000	1262500000	1143600000	1124400000	1783300000
sp|P51692	STAT5B	sp|P51692	2	7	7	7	9.5	89.865	113740000	122160000	0	177720000	89823000	166950000
sp|P51790	CLCN3	sp|P51790	1	1	1	1	2.2	90.965	0	0	0	0	0	22720000
sp|P51808	DYNLT3	sp|P51808	1	3	3	3	65	13.062	0	150230000	152110000	166110000	205410000	0
sp|P51809	VAMP7	sp|P51809	1	5	5	5	29	24.935	128480000	274060000	239620000	249290000	324160000	0
sp|P51812	RPS6KA3	sp|P51812	1	13	13	8	23	83.735	901500000	766470000	951940000	960350000	893180000	1040400000
sp|P51857	AKR1D1	sp|P51857	1	1	1	1	2.8	37.376	0	0	0	0	0	509160000
sp|P51858	HDGF	sp|P51858	1	21	21	20	79	26.788	1.064E+10	1.0242E+10	9986600000	10287000000	9957200000	9304000000
sp|P51948	MNAT1	sp|P51948	1	3	3	3	14	35.823	411840000	331220000	279760000	316610000	0	0
sp|P51965	UBE2E1	sp|P51965	1	1	1	1	9.3	21.404	0	0	0	0	0	85245000
sp|P51970	NDUFA8	sp|P51970	1	6	6	6	52	20.105	594350000	530450000	404120000	483860000	473330000	390240000
sp|P51991	HNRNPA3	sp|P51991	1	14	14	14	44	39.594	1.3958E+10	1.1604E+10	1.2257E+10	12892000000	12967000000	1.1704E+10
sp|P52209	PGD	sp|P52209	1	22	22	22	59	53.139	1.094E+10	1.0262E+10	1.2862E+10	9554800000	7527900000	8354400000
sp|P52272	HNRNPM	sp|P52272	1	37	37	37	60	77.515	1.6257E+10	1.7805E+10	1.6181E+10	18698000000	17717000000	2.0665E+10
sp|P52292	KPNA2	sp|P52292	1	17	17	17	53	57.861	5706400000	4845100000	5069400000	5157200000	4969700000	5010700000
sp|P52294	KPNA1	sp|P52294	1	8	5	5	18	60.221	338580000	340640000	277510000	314020000	360260000	274140000
sp|P52298	NCBP2	sp|P52298	1	3	3	3	21	18.001	118310000	109000000	0	78151000	105800000	0
sp|P52306	RAP1GDS	sp|P52306	1	15	15	15	36	66.316	907040000	1045700000	970180000	997890000	894910000	908250000
sp|P52434	POLR2H	sp|P52434	1	5	5	5	41	17.143	944380000	1003800000	885360000	703430000	1119700000	1165900000
sp|Q9H1A	POLR2J3	sp|Q9H1A	3	2	2	2	24	13.092	39516000	46400000	60055000	64752000	46119000	37095000
sp|P52565	ARHGDIA	sp|P52565	1	9	9	9	65	23.207	1.0332E+10	1.034E+10	1.2988E+10	10820000000	9948500000	1.157E+10
sp|P52594	AGFG1	sp|P52594	1	5	5	5	17	58.259	539860000	711380000	855680000	521850000	547260000	480130000
sp|P52597	HNRNPF	sp|P52597	1	17	15	15	58	45.671	4163900000	4273200000	4386300000	4461000000	4937800000	4873400000
sp|P52655	GTF2A1	sp|P52655	1	1	1	1	2.9	41.513	0	0	0	0	0	56785000
sp|P52701	MSH6	sp|P52701	1	21	21	21	23	152.78	1292000000	1189000000	1549400000	1386200000	1255900000	1371600000
sp|P52732	KIF11	sp|P52732	1	7	7	7	8.7	119.16	385830000	366470000	331720000	432520000	500270000	448360000
sp|P52735	VAV2	sp|P52735	1	8	8	8	13	101.29	169160000	129210000	206060000	185370000	185220000	253200000
sp|P52756	RBM5	sp|P52756	1	3	3	3	4.8	92.153	0	0	0	0	69214000	0
sp|P52758	RIDA	sp|P52758	1	1	1	1	12	14.494	0	0	0	0	0	111320000
sp|P52788	SMS	sp|P52788	1	14	14	14	56	41.268	1212600000	1525000000	1294200000	1331500000	1244800000	1063000000

sp|P52789	HK2	sp|P52789	2	16	12	12	22	102.38	535980000	563380000	475230000	673570000	894200000	706040000
sp|P52815	MRPL12	sp|P52815	1	6	6	6	40	21.348	1489700000	2787000000	1133000000	2183700000	2661800000	2655500000
sp|P52888	THOP1	sp|P52888	1	11	11	11	20	78.839	1181200000	900520000	1007300000	938130000	1024800000	831500000
sp|P52895	AKR1C2	sp|P52895	1	18	18	5	65	36.735	2.9256E+10	3.0178E+10	2.6721E+10	27855000000	25553000000	2.4395E+10
sp|P52907	CAPZA1	sp|P52907	1	12	12	10	66	32.922	6949200000	6159700000	8003200000	8647100000	8206600000	7951800000
sp|P52943	CRIP2	sp|P52943	1	9	9	9	48	22.492	2862900000	2810300000	2168400000	2248200000	2941300000	1910500000
sp|P52948	NUP98	sp|P52948	1	22	22	22	16	197.58	1539000000	1353800000	1474400000	1587200000	1307600000	1175300000
sp|P53004	BLVRA	sp|P53004	1	5	5	5	21	33.428	572920000	636740000	612700000	533590000	600900000	608200000
sp|P53007	SLC25A1	sp|P53007	1	4	4	4	15	34.012	339650000	465600000	448870000	275080000	340510000	332120000
sp|P53041	PPP5C	sp|P53041	1	8	8	8	23	56.878	825740000	694090000	769160000	732880000	804640000	969160000
sp|P53350	PLK1	sp|P53350	1	9	9	9	23	68.254	266100000	343560000	369230000	484850000	544220000	693200000
sp|P53365	ARFIP2	sp|P53365	1	3	3	3	8.8	37.855	0	0	0	0	182180000	0
sp|P53367	ARFIP1	sp|P53367	1	6	6	6	25	41.738	0	261360000	332330000	0	0	265720000
sp|P53384	NUBP1	sp|P53384	1	4	4	4	20	34.534	237590000	254010000	274110000	242200000	339880000	328390000
sp|P53396	ACLY	sp|P53396	1	49	49	49	50	120.84	2.415E+10	2.4083E+10	2.526E+10	23003000000	21585000000	2.0998E+10
sp|P53582	METAP1	sp|P53582	1	10	10	10	40	43.215	1003400000	829860000	1137600000	814410000	896520000	978060000
sp|P53597	SUCLG1	sp|P53597	1	5	5	5	20	36.249	904780000	603850000	911580000	1254700000	1616700000	1504900000
sp|P53602	MVD	sp|P53602	1	2	2	2	11	43.404	0	0	0	0	49373000	0
sp|P53611	RABGGTE	sp|P53611	1	3	3	3	11	36.924	0	145100000	261670000	221210000	0	167910000
sp|P53618	COPB1	sp|P53618	1	24	24	24	38	107.14	4738400000	4471500000	4844600000	4654000000	4520900000	4989700000
sp|P53621	COPA	sp|P53621	1	53	53	53	54	138.34	7418600000	8834100000	7605400000	7845200000	8070400000	7367900000
sp|P53634	CTSC	sp|P53634	1	7	7	7	21	51.853	265690000	293880000	274650000	384050000	446930000	524050000
sp|P53680	AP2S1	sp|P53680	1	3	3	3	17	17.018	271870000	187950000	180940000	0	0	144070000
sp|P53701	HCCS	sp|P53701	1	13	13	13	59	30.601	1600500000	1180400000	1607400000	1471700000	1390200000	1057300000
sp|P53801	PTTG1IP	sp|P53801	1	2	2	2	14	20.324	541460000	0	209440000	882500000	388040000	217320000
sp|P53985	SLC16A1	sp|P53985	1	5	5	5	8	53.944	1407000000	759790000	701030000	1236300000	1018500000	888600000
sp|P53990	IST1	sp|P53990	1	6	6	6	19	39.75	229380000	225160000	216870000	243060000	190110000	152600000
sp|P53992	SEC24C	sp|P53992	1	15	15	15	20	118.32	1164500000	1001200000	1166500000	1284200000	1188500000	1101600000
sp|P53999	SUB1	sp|P53999	1	7	7	7	60	14.395	1.1036E+10	1.0301E+10	8775200000	9093100000	11414000000	1.031E+10
sp|P54105	CLNS1A	sp|P54105	1	5	5	5	41	26.215	594310000	839500000	875330000	666940000	567720000	798110000
sp|P54136	RARS	sp|P54136	1	29	29	29	51	75.378	5308900000	5163300000	5556800000	5250300000	5142200000	5046400000
sp|P54289	CACNA2D	sp|P54289	2	5	5	5	6.6	124.57	108610000	107580000	85393000	74775000	149030000	124380000
sp|P54577	YARS	sp|P54577	1	30	30	30	57	59.143	6744800000	7647900000	6445800000	7800800000	7551000000	8708400000
sp|P54578	USP14	sp|P54578	1	20	20	20	52	56.068	3363300000	3500800000	2975800000	3077200000	3192800000	3006000000
sp|P54619	PRKAG1	sp|P54619	1	2	2	1	8.5	37.579	131440000	91045000	106410000	72912000	85178000	0
sp|P54687	BCAT1	sp|P54687	1	2	2	2	8.5	42.966	0	0	43566000	52838000	53458000	0
sp|P54709	ATP1B3	sp|P54709	1	10	10	10	42	31.512	1069900000	1017500000	1193000000	1184200000	1226000000	1174400000
sp|P54725	RAD23A	sp|P54725	1	12	10	10	57	39.609	1380500000	1385400000	1243700000	982450000	993380000	1528700000
sp|P54727	RAD23B	sp|P54727	1	13	13	11	53	43.171	3500900000	3688700000	3302900000	2996900000	2770200000	3247200000

sp|P54819	AK2	sp|P54819	1	10	10	10	56	26.477	4345600000	3526800000	3598100000	3371900000	3326600000	3666900000
sp|P54852	EMP3	sp|P54852	1	1	1	1	8.6	18.429	0	0	0	0	0	136210000
sp|P54886	ALDH18A	sp|P54886	1	27	27	27	42	87.301	3199100000	2632600000	2832200000	3080700000	2822600000	2914100000
sp|P54920	NAPA	sp|P54920	2	12	12	12	54	33.232	2183000000	1955700000	1523600000	2115500000	2620500000	2383900000
sp|P55010	EIF5	sp|P55010	1	8	8	8	19	49.222	1729200000	1852600000	1838700000	1494400000	1637000000	1788400000
sp|P55011	SLC12A2	sp|P55011	1	4	4	4	6.8	131.45	118390000	99761000	110720000	0	158050000	105660000
sp|P55036	PSMD4	sp|P55036	1	10	10	9	40	40.736	1706700000	1560800000	1566200000	1536600000	1927800000	1641400000
sp|P55039	DRG2	sp|P55039	1	10	10	10	35	40.746	308850000	516380000	471520000	632350000	434190000	392680000
sp|P55060	CSE1L	sp|P55060	1	33	33	33	39	110.42	1.5776E+10	1.4539E+10	1.4296E+10	14722000000	12812000000	1.4364E+10
sp|P55072	VCP	sp|P55072	1	47	47	47	71	89.321	4.317E+10	4.3019E+10	4.2441E+10	39421000000	40680000000	3.6273E+10
sp|P55081	MFAP1	sp|P55081	1	3	3	3	11	51.958	206990000	0	288490000	176920000	198820000	239740000
sp|P55084	HADHB	sp|P55084	1	17	17	17	41	51.294	2077100000	2302100000	2028300000	2526300000	2731100000	2351000000
sp|P55145	MANF	sp|P55145	1	8	8	8	35	20.7	1594900000	1533200000	1533100000	1867600000	2903500000	1586300000
sp|P55196	AFDN	sp|P55196	1	3	3	3	2.4	206.8	0	0	0	0	0	1029400000
sp|P55209	NAP1L1	sp|P55209	1	11	10	10	39	45.374	5344700000	5305500000	5952000000	6192600000	6805800000	6313100000
sp|P55211	CASP9	sp|P55211	1	1	1	1	7.7	46.28	0	0	0	0	29482000	0
sp|P55212	CASP6	sp|P55212	1	1	1	1	3.8	33.31	0	0	0	0	0	8147600
sp|P55263	ADK	sp|P55263	1	9	9	9	27	40.545	1210400000	1019500000	1227000000	1190000000	1085000000	1118200000
sp|P55265	ADAR	sp|P55265	1	24	24	24	30	136.06	2063600000	1669000000	2352700000	2106900000	2007900000	2268400000
sp|P55290	CDH13	sp|P55290	1	1	1	1	2	78.286	0	0	0	0	0	67919000
sp|P55327	TPD52	sp|P55327	1	9	9	9	58	24.327	1879800000	2168500000	2390800000	1739600000	2003000000	2081000000
sp|P55735	SEC13	sp|P55735	1	9	9	9	45	35.54	2998700000	2840900000	2752300000	2486700000	3261200000	2625500000
sp|P55769	SNU13	sp|P55769	1	7	7	7	53	14.173	1736100000	1914800000	1974800000	1785200000	1909500000	2243500000
sp|P55786	NPEPPS	sp|P55786	2	34	34	34	48	103.28	4964600000	5468600000	4929800000	4995900000	4682500000	4456100000
sp|P55795	HNRNPH2	sp|P55795	1	11	6	6	37	49.263	1387800000	1426700000	1410400000	1790000000	1967300000	1634500000
sp|P55809	OXCT1	sp|P55809	2	17	17	17	41	56.157	7003300000	6614100000	7772500000	7526400000	8428000000	8513200000
sp|P55854	SUMO3	sp|P55854	2	2	2	1	20	11.637	826140000	0	1442400000	0	0	1963600000
sp|P55884	EIF3B	sp|P55884	1	29	29	29	47	92.48	7799300000	8013400000	7951200000	8369900000	7605000000	8763500000
sp|P55957	BID	sp|P55957	1	2	2	2	20	21.994	290080000	352060000	242920000	207930000	231810000	313120000
sp|P56134	ATP5J2	sp|P56134	1	3	3	3	39	10.918	539930000	618190000	626760000	552130000	573200000	522660000
sp|P56182	RRP1	sp|P56182	1	12	12	12	30	52.839	799020000	1069700000	913460000	981070000	967120000	898270000
sp|P56192	MARS	sp|P56192	1	27	27	27	43	101.11	3979900000	4009300000	3796900000	4352600000	4095500000	3853200000
sp|P56211	ARPP19	sp|P56211	1	5	4	4	57	12.323	179220000	159950000	0	253300000	0	105320000
sp|P56270	MAZ	sp|P56270	1	2	2	2	6.7	48.607	164350000	139760000	161320000	231440000	165930000	189440000
sp|Q5VTU	ATP5EP2	sp|Q5VTU	2	2	2	2	29	5.8068	836280000	565410000	609630000	920620000	161370000	1054800000
sp|P56385	ATP5ME	sp|P56385	1	4	4	4	42	7.9331	876740000	1064900000	790580000	1057500000	1200900000	1019100000
sp|P56537	EIF6	sp|P56537	1	8	8	8	57	26.599	4176500000	4812800000	4696400000	4745600000	5503900000	5377700000
sp|P56545	CTBP2	sp|P56545	1	10	10	6	25	48.944	1270400000	1755700000	1653400000	1234000000	1722800000	1435300000
sp|P56556	NDUFA6	sp|P56556	1	2	2	2	15	15.136	179440000	210030000	160910000	177640000	167180000	172150000

sp|P56589	PEX3	sp|P56589	1	2	2	2	9.7	42.139	32779000	24866000	0	0	22189000	24014000
sp|P56945	BCAR1	sp|P56945	1	6	6	6	11	93.371	141560000	0	113170000	0	0	97232000
sp|P57076	CFAP298	sp|P57076	1	2	2	2	14	33.224	0	0	0	0	61503000	0
sp|P57081	WDR4	sp|P57081	1	2	2	2	8	45.489	0	0	166650000	198440000	108630000	135840000
sp|P57088	TMEM33	sp|P57088	1	5	5	5	19	27.978	818710000	1099200000	944420000	1094800000	1048500000	984400000
sp|P57678	GEMIN4	sp|P57678	1	9	9	9	14	120.04	283060000	470350000	321990000	319040000	303320000	323050000
sp|P57740	NUP107	sp|P57740	1	11	11	11	19	106.37	512760000	407960000	542360000	487440000	725410000	521220000
sp|P57772	EEFSEC	sp|P57772	1	1	1	1	2.2	65.304	0	0	0	0	83028000	0
sp|P58107	EPPK1	sp|P58107	1	93	85	85	52	555.65	1.2122E+10	1.2203E+10	1.1758E+10	13131000000	13018000000	1.3608E+10
sp|P58546	MTPN	sp|P58546	1	5	5	5	58	12.895	1908800000	2420100000	2200200000	1794900000	2195600000	2197500000
sp|P59998	ARPC4	sp|P59998	1	5	5	5	36	19.667	2747500000	2442100000	2393400000	2216900000	2070600000	2710900000
sp|P60002	ELOF1	sp|P60002	1	1	1	1	22	9.4618	0	113780000	0	0	180750000	0
sp|P60033	CD81	sp|P60033	1	3	3	3	25	25.809	311350000	341470000	0	177260000	0	284100000
sp|P60059	SEC61G	sp|P60059	1	2	2	2	37	7.7412	710120000	380960000	335730000	1021300000	1128100000	1086400000
sp|P60174	TPI1	sp|P60174	1	18	18	18	74	30.791	6.0919E+10	6.1797E+10	6.5101E+10	61314000000	64455000000	6.8415E+10
sp|P60228	EIF3E	sp|P60228	1	18	18	18	44	52.22	3877800000	3505200000	3865100000	3274500000	3935500000	3861600000
sp|P60468	SEC61B	sp|P60468	1	3	3	3	38	9.9743	2155300000	1604100000	1449300000	1916000000	1736300000	2364500000
sp|P60510	PPP4C	sp|P60510	1	4	3	3	21	35.08	251890000	266260000	224770000	258960000	254720000	214980000
sp|P60602	ROMO1	sp|P60602	1	1	1	1	22	8.1828	0	0	0	0	0	482150000
sp|P60604	UBE2G2	sp|P60604	1	2	2	2	22	18.566	0	168130000	177290000	161000000	213330000	0
sp|P60660	MYL6	sp|P60660	1	8	8	6	56	16.93	1.6941E+10	1.7356E+10	1.4107E+10	14291000000	18151000000	1.4657E+10
sp|P60709	ACTB	sp|P60709	2	24	1	1	77	41.736	0	0	0	0	8390700000	0
sp|P60842	EIF4A1	sp|P60842	1	26	26	14	72	46.153	4.1585E+10	3.7143E+10	4.5889E+10	42068000000	41957000000	4.5046E+10
sp|P60866	RPS20	sp|P60866	1	3	3	3	25	13.373	6568700000	7868200000	6843300000	5980000000	7279900000	8539200000
sp|P60891	PRPS1	sp|P60891	1	13	13	4	44	34.834	3338600000	3619400000	3320000000	3607200000	3141600000	3291700000
sp|P60900	PSMA6	sp|P60900	1	13	13	13	59	27.399	6760000000	5409500000	6393300000	5665400000	6382100000	6699000000
sp|P60903	S100A10	sp|P60903	1	4	4	4	36	11.203	9380300000	5107000000	4583100000	6254500000	7660400000	6784600000
sp|P60953	CDC42	sp|P60953	3	8	7	7	48	21.258	4029500000	5337300000	4256900000	4363300000	4271200000	4059400000
sp|P60981	DSTN	sp|P60981	1	14	13	13	69	18.506	7519800000	6181600000	6539900000	5503300000	5393300000	5815900000
sp|P60983	GMFB	sp|P60983	2	5	5	5	49	16.713	519720000	627040000	554340000	569570000	627210000	634780000
sp|P61006	RAB8A	sp|P61006	4	9	7	5	37	23.668	971810000	828610000	986070000	736110000	845580000	830860000
sp|P61009	SPCS3	sp|P61009	1	3	3	3	18	20.313	319910000	384670000	292220000	350530000	440130000	541640000
sp|P61011	SRP54	sp|P61011	1	15	15	15	38	55.704	1018300000	980060000	1044000000	1136500000	1114800000	886430000
sp|P61019	RAB2A	sp|P61019	1	12	12	7	59	23.545	2473100000	2451400000	2252400000	2146500000	1968700000	2232000000
sp|P61020	RAB5B	sp|P61020	1	6	3	3	33	23.707	249860000	221560000	0	247550000	262250000	208510000
sp|P61024	CKS1B	sp|P61024	1	2	2	2	33	9.6601	372180000	386560000	269160000	458920000	476380000	355620000
sp|P61026	RAB10	sp|P61026	1	11	11	10	45	22.541	3735000000	5148500000	4123100000	3599800000	4266600000	4975200000
sp|P61073	CXCR4	sp|P61073	1	3	3	3	11	39.745	170010000	0	168340000	198500000	144860000	0
sp|P61077	UBE2D3	sp|P61077	2	3	3	3	29	16.687	742990000	607590000	2131400000	1294900000	1041400000	838620000

sp|P61081	UBE2M	sp|P61081	1	7	7	7	42	20.9	1612400000	1844300000	1260200000	1786700000	2194300000	2244300000
sp|P61086	UBE2K	sp|P61086	1	9	9	9	50	22.406	1515800000	1478700000	1532900000	1277900000	1444400000	1402900000
sp|P61088	UBE2N	sp|P61088	2	7	7	7	52	17.138	3670500000	3410900000	4160700000	3369600000	3005200000	3607200000
sp|P61106	RAB14	sp|P61106	1	14	14	14	73	23.897	4988700000	5029900000	5437900000	5108100000	5089000000	6117300000
sp|P61158	ACTR3	sp|P61158	3	21	21	21	60	47.371	8464400000	8021200000	9136400000	8474800000	8308400000	9012200000
sp|P61160	ACTR2	sp|P61160	1	10	10	10	29	44.76	4741900000	5640000000	5185200000	4254600000	4002400000	5707900000
sp|P61163	ACTR1A	sp|P61163	1	10	10	5	33	42.613	1407000000	1275800000	1261900000	1476300000	1484400000	1240500000
sp|P61165	TMEM258	sp|P61165	1	1	1	1	10	9.0788	184270000	194190000	162460000	194250000	0	209750000
sp|P61201	COPS2	sp|P61201	1	15	15	15	42	51.596	1509200000	1296600000	1379300000	1287900000	1434900000	1333400000
sp|P84077	ARF1	sp|P84077	2	10	6	5	59	20.697	2593500000	4005200000	3152700000	3568600000	3077000000	3052000000
sp|P61221	ABCE1	sp|P61221	1	19	19	19	41	67.314	4052300000	3578200000	3700600000	3483800000	3149700000	3885700000
sp|P61224	RAP1B	sp|P61224	2	9	9	5	52	20.825	1956300000	2608500000	3366100000	3207700000	2818600000	2582200000
sp|P61225	RAP2B	sp|P61225	1	5	3	3	40	20.504	62561000	109610000	82498000	67249000	94629000	106480000
sp|P61247	RPS3A	sp|P61247	1	16	16	16	57	29.945	1.9523E+10	1.8443E+10	1.9257E+10	22757000000	25360000000	2.1682E+10
sp|P61254	RPL26	sp|P61254	1	9	9	2	45	17.258	1.1682E+10	1.1293E+10	1.1751E+10	11707000000	13566000000	1.2563E+10
sp|P61289	PSME3	sp|P61289	1	12	12	12	56	29.506	2950200000	2832200000	2635600000	2649800000	2959200000	2932100000
sp|P61313	RPL15	sp|P61313	1	10	10	10	40	24.146	1.0722E+10	1.1382E+10	1.0537E+10	11610000000	12260000000	1.3771E+10
sp|P61326	MAGOH	sp|P61326	1	4	1	1	31	17.163	106550000	114800000	109690000	126580000	184540000	155630000
sp|P61353	RPL27	sp|P61353	1	5	5	5	43	15.798	1.0451E+10	1.0384E+10	1.305E+10	8705000000	11897000000	1.1354E+10
sp|P61421	ATP6V0D	sp|P61421	1	7	7	7	29	40.329	636840000	917800000	634200000	923130000	617420000	596460000
sp|P61457	PCBD1	sp|P61457	1	3	3	3	36	11.999	0	0	282950000	236570000	0	152780000
sp|P61513	RPL37A	sp|P61513	2	4	4	4	50	10.275	2483300000	1524300000	2392700000	3013400000	2184900000	2438000000
sp|P61586	RHOA	sp|P61586	1	9	9	4	51	21.768	4685800000	4202900000	4470900000	4003400000	4308400000	4624800000
sp|P61587	RND3	sp|P61587	1	1	1	1	5.7	27.368	0	0	0	0	0	60259000
sp|P61599	NAA20	sp|P61599	1	1	1	1	6.2	20.368	0	0	0	0	17996000	0
sp|P61604	HSPE1	sp|P61604	1	10	10	10	70	10.932	2.1732E+10	2.4509E+10	2.0763E+10	20996000000	21482000000	2.4162E+10
sp|P61619	SEC61A1	sp|P61619	2	8	8	8	16	52.264	2093000000	2074400000	1664300000	1982600000	1938200000	2098400000
sp|P61758	VBP1	sp|P61758	1	9	9	9	49	22.658	999560000	956980000	1195100000	1114000000	1332000000	1040800000
sp|P61764	STXBP1	sp|P61764	1	9	9	9	20	67.568	279260000	384280000	313990000	287450000	331280000	284280000
sp|P61769	B2M	sp|P61769	1	3	3	3	35	13.714	2975600000	2778500000	3650800000	2766200000	2849400000	1437000000
sp|P61803	DAD1	sp|P61803	1	4	4	4	35	12.497	1363500000	1471100000	1199000000	1889900000	1795600000	1504900000
sp|P61916	NPC2	sp|P61916	1	2	2	2	21	16.57	130410000	202110000	135670000	171940000	0	204370000
sp|P61923	COPZ1	sp|P61923	1	4	4	4	33	20.198	939280000	1200800000	976650000	886510000	1363100000	765380000
sp|P61956	SUMO2	sp|P61956	1	2	1	1	23	10.871	0	0	0	0	0	1578600000
sp|P61960	UFM1	sp|P61960	1	3	3	3	59	9.1175	686450000	720050000	702010000	747580000	841210000	646090000
sp|P61962	DCAF7	sp|P61962	1	2	2	2	6.7	38.926	0	0	0	0	0	82367000
sp|P61964	WDR5	sp|P61964	1	6	6	6	25	36.588	576900000	498870000	563880000	489930000	600130000	559550000
sp|P61966	AP1S1	sp|P61966	1	3	3	2	21	18.733	161230000	129790000	159290000	147270000	159520000	141940000
sp|P61970	NUTF2	sp|P61970	1	6	6	6	75	14.478	1817200000	1586100000	1911500000	1329300000	1982100000	1508700000

sp|P61978	HNRNPK	sp|P61978	1	22	22	22	59	50.976	4.3873E+10	3.8136E+10	4.1403E+10	43797000000	40864000000	4.0947E+10
sp|P61981	YWHAG	sp|P61981	1	13	10	10	60	28.302	1.0082E+10	1.0815E+10	1.0879E+10	10319000000	10482000000	1.1413E+10
sp|P62070	RRAS2	sp|P62070	1	3	3	2	20	23.399	261080000	188280000	226950000	276270000	326950000	379280000
sp|P62072	TIMM10	sp|P62072	1	1	1	1	14	10.333	0	0	0	0	0	88787000
sp|P62081	RPS7	sp|P62081	1	10	10	10	63	22.127	1.0865E+10	1.2382E+10	1.0487E+10	11558000000	10675000000	1.1845E+10
sp|P62136	PPP1CA	sp|P62136	1	17	17	6	62	37.512	6913900000	7349700000	6792700000	6631300000	7340100000	5562700000
sp|P62140	PPP1CB	sp|P62140	1	16	6	6	60	37.186	1486500000	1440300000	968580000	1035600000	1036500000	1498700000
sp|P62166	NCS1	sp|P62166	1	2	2	2	14	21.878	24567000	45622000	0	0	56620000	0
sp|P62191	PSMC1	sp|P62191	1	20	20	20	56	49.184	2985600000	3746100000	3049000000	2695000000	3147200000	3516200000
sp|P62195	PSMC5	sp|P62195	1	24	24	23	62	45.626	3373600000	3697100000	3465600000	3373300000	3462200000	3952100000
sp|P62241	RPS8	sp|P62241	1	10	10	10	50	24.205	1.58E+10	1.5116E+10	1.5498E+10	16745000000	14572000000	1.6385E+10
sp|P62244	RPS15A	sp|P62244	1	6	6	6	42	14.839	6311300000	7099600000	6666200000	6004600000	5696500000	4610300000
sp|P62249	RPS16	sp|P62249	1	10	10	10	54	16.445	1.0735E+10	9555800000	9950900000	10253000000	12303000000	1.0767E+10
sp|P62253	UBE2G1	sp|P62253	1	3	3	3	24	19.509	159130000	153290000	90803000	88652000	157300000	91356000
sp|P62256	UBE2H	sp|P62256	1	5	5	5	31	20.655	502660000	773160000	540810000	758830000	584460000	642390000
sp|P62258	YWHAE	sp|P62258	1	20	20	18	73	29.174	4.3429E+10	4.5376E+10	4.6188E+10	38810000000	46200000000	3.8586E+10
sp|P62263	RPS14	sp|P62263	1	6	6	6	38	16.273	1.4284E+10	1.3751E+10	1.6034E+10	16320000000	15757000000	1.5319E+10
sp|P62266	RPS23	sp|P62266	1	6	6	6	48	15.807	6006600000	6313400000	6711600000	6208100000	6920800000	5011400000
sp|P62269	RPS18	sp|P62269	1	10	10	10	50	17.718	1.4756E+10	1.578E+10	1.4473E+10	15965000000	15452000000	1.2791E+10
sp|P62273	RPS29	sp|P62273	1	4	4	4	48	6.6767	2076400000	2088100000	2550600000	2231600000	2455400000	1105800000
sp|P62277	RPS13	sp|P62277	1	10	10	10	57	17.222	7059800000	6722100000	8138900000	7693100000	7158400000	7084400000
sp|P62280	RPS11	sp|P62280	1	13	13	13	61	18.431	1.3892E+10	1.2582E+10	1.4698E+10	12316000000	13708000000	1.4497E+10
sp|P62304	SNRPE	sp|P62304	1	3	3	3	52	10.803	943720000	1078400000	911230000	1428700000	1042800000	459070000
sp|P62306	SNRPF	sp|P62306	1	2	2	2	24	9.7251	986430000	700980000	924470000	903590000	977750000	971630000
sp|P62310	LSM3	sp|P62310	1	3	3	3	40	11.845	474930000	495870000	540830000	373250000	476520000	365740000
sp|P62312	LSM6	sp|P62312	1	2	2	2	20	9.1275	103530000	165860000	121780000	0	0	140340000
sp|P62314	SNRPD1	sp|P62314	1	5	5	5	55	13.281	2709400000	2547600000	2030600000	2853400000	3340500000	1236600000
sp|P62316	SNRPD2	sp|P62316	1	9	9	9	68	13.527	4841700000	4954800000	4060200000	4234600000	4080100000	4450000000
sp|P62318	SNRPD3	sp|P62318	1	5	5	5	53	13.916	5542300000	5650400000	3688200000	5461100000	6253400000	5093200000
sp|P62328	TMSB4X	sp|P62328	1	3	3	3	61	5.0526	7391200000	1.1112E+10	7958500000	6865200000	11891000000	1.2451E+10
sp|P62330	ARF6	sp|P62330	1	4	4	4	42	20.082	605270000	583550000	616660000	572480000	837910000	555470000
sp|P62333	PSMC6	sp|P62333	1	19	19	19	59	44.172	4912800000	4274700000	4054800000	4288500000	3622400000	3683900000
sp|P62341	SELENOT	sp|P62341	1	1	1	1	6.7	22.324	0	0	0	0	0	81364000
sp|P62380	TBPL1	sp|P62380	1	1	1	1	10	20.886	0	0	0	0	0	70557000
sp|P62424	RPL7A	sp|P62424	1	17	17	17	45	29.995	2.0922E+10	2.1169E+10	1.873E+10	18758000000	21877000000	2.1432E+10
sp|P62495	ETF1	sp|P62495	1	17	17	17	46	49.03	4072900000	4792300000	4502900000	4080400000	4123500000	4192200000
sp|P62633	CNBP	sp|P62633	1	7	7	7	48	19.463	4248600000	5170600000	4261000000	4045600000	5299200000	4013500000
sp|P62701	RPS4X	sp|P62701	3	17	17	17	59	29.597	1.5438E+10	1.7146E+10	1.4746E+10	17161000000	17509000000	1.7955E+10
sp|P62745	RHOB	sp|P62745	1	3	2	2	20	22.123	0	0	0	72837000	0	0

sp|P62750	RPL23A	sp|P62750	2	9	9	9	39	17.695	1.053E+10	1.2696E+10	1.0414E+10	11108000000	13349000000	1.1626E+10
sp|P62753	RPS6	sp|P62753	1	7	7	7	29	28.68	6551400000	1.0523E+10	7535800000	7476300000	8610800000	9898300000
sp|P62805	HIST1H4A	sp|P62805	1	11	11	11	60	11.367	1.6528E+11	1.118E+11	1.96E+11	1.8663E+11	2.1603E+11	1.924E+11
sp|P62820	RAB1A	sp|P62820	1	10	5	5	54	22.677	1533900000	1605300000	1770000000	1832700000	1953600000	1885100000
sp|P62826	RAN	sp|P62826	1	12	12	12	57	24.423	2.2022E+10	1.9252E+10	2.5163E+10	20972000000	18342000000	2.1106E+10
sp|P62829	RPL23	sp|P62829	1	7	7	7	62	14.865	1.2925E+10	1.1562E+10	1.1278E+10	12597000000	13662000000	1.184E+10
sp|P62834	RAP1A	sp|P62834	1	7	3	3	45	20.987	171250000	0	183160000	201210000	289950000	242610000
sp|P62841	RPS15	sp|P62841	1	3	3	3	22	17.04	1608600000	2351900000	2251300000	1604800000	1551000000	1510100000
sp|P62847	RPS24	sp|P62847	1	4	4	4	26	15.423	2653500000	3370600000	2483000000	2766100000	4136200000	3340800000
sp|P62851	RPS25	sp|P62851	1	5	5	5	30	13.742	6159300000	7139900000	5301200000	5654300000	7876000000	7442600000
sp|P62854	RPS26	sp|P62854	2	3	3	3	31	13.015	4246200000	3759200000	3383900000	3993000000	5111500000	2593000000
sp|P62857	RPS28	sp|P62857	1	2	2	2	30	7.8409	3221200000	4389000000	3660800000	3937600000	4721200000	4424300000
sp|P62861	FAU	sp|P62861	1	1	1	1	17	6.6478	0	0	0	0	0	1676000000
sp|P62873	GNB1	sp|P62873	1	13	13	8	58	37.377	3089800000	2892500000	2637300000	2609900000	2761600000	2717600000
sp|P62877	RBX1	sp|P62877	1	2	2	2	24	12.274	0	516070000	0	0	480030000	0
sp|P62879	GNB2	sp|P62879	1	12	8	5	45	37.331	1623500000	2139700000	1993600000	1207300000	1391300000	1399600000
sp|P62888	RPL30	sp|P62888	1	7	7	7	67	12.784	1.1512E+10	1.1591E+10	1.4024E+10	11210000000	12568000000	1.368E+10
sp|Q59GN	RPL39P5	sp|Q59GN	2	1	1	1	20	6.3225	0	0	0	0	0	147470000
sp|P62899	RPL31	sp|P62899	1	5	5	5	37	14.463	5391100000	5851700000	6023000000	5472700000	4954200000	4905900000
sp|P62906	RPL10A	sp|P62906	1	9	9	9	39	24.831	1.2891E+10	1.1658E+10	1.4096E+10	11657000000	13390000000	1.2335E+10
sp|P62910	RPL32	sp|P62910	1	7	7	7	53	15.86	5528700000	4347800000	6200500000	5245800000	5076200000	5204500000
sp|P62913	RPL11	sp|P62913	1	8	8	8	46	20.252	9182700000	1.0614E+10	9142300000	10443000000	10863000000	9525600000
sp|P62917	RPL8	sp|P62917	1	12	12	12	55	28.024	1.2941E+10	1.2483E+10	1.1295E+10	12277000000	12723000000	1.5852E+10
sp|P62937	PPIA	sp|P62937	1	12	12	10	70	18.012	6.4379E+10	7.9933E+10	7.6037E+10	78488000000	76169000000	6.7106E+10
sp|P62942	FKBP1A	sp|P62942	1	6	6	6	42	11.951	2211600000	1946700000	2592500000	2033600000	2223700000	2082700000
sp|P62979	RPS27A	sp|P62979	4	8	8	8	51	17.965	4.1625E+10	4.0177E+10	3.5724E+10	44417000000	46107000000	5.5279E+10
sp|P62993	GRB2	sp|P62993	1	7	7	7	45	25.206	373770000	340950000	531010000	472260000	614790000	428620000
sp|P62995	TRA2B	sp|P62995	1	7	7	6	30	33.665	1221600000	1034700000	1166300000	1326200000	1476000000	1366800000
sp|P63000	RAC1	sp|P63000	2	8	7	4	44	21.45	2916200000	2801900000	2467500000	2325400000	2274100000	2227900000
sp|P63010	AP2B1	sp|P63010	1	27	27	17	42	104.55	3937100000	4220000000	3722800000	4143200000	4350300000	3860100000
sp|P63092	GNAS	sp|P63092	3	9	8	8	34	45.664	666070000	565650000	465340000	612090000	596460000	565700000
sp|P63096	GNAI1	sp|P63096	5	10	5	5	40	40.361	331510000	343920000	278590000	320870000	374060000	364440000
sp|P63098	PPP3R1	sp|P63098	1	5	5	5	49	19.3	118510000	187470000	156400000	99870000	118790000	115790000
sp|P63104	YWHAZ	sp|P63104	1	17	15	13	70	27.745	4.5409E+10	4.1377E+10	4.1346E+10	45431000000	52732000000	4.3723E+10
sp|P63151	PPP2R2A	sp|P63151	4	12	12	12	36	51.691	1184300000	1238100000	1549000000	1427000000	1140600000	1193600000
sp|P63165	SUMO1	sp|P63165	1	4	4	4	43	11.557	798910000	637300000	962700000	782590000	967190000	565770000
sp|P63167	DYNLL1	sp|P63167	1	4	4	2	57	10.366	2127800000	3084500000	2011200000	2024000000	2385800000	3084900000
sp|P63172	DYNLT1	sp|P63172	1	3	3	3	50	12.452	77058000	0	250510000	285150000	385130000	294610000
sp|P63173	RPL38	sp|P63173	1	6	6	6	50	8.2178	2265100000	2583700000	1857500000	2232300000	1888100000	1954700000

sp|P63208	SKP1	sp|P63208	1	10	10	10	62	18.658	3271100000	2750800000	3097200000	2917100000	3406300000	3061300000
sp|P63218	GNG5	sp|P63218	1	2	2	2	53	7.3184	651020000	448540000	0	499180000	0	0
sp|P63220	RPS21	sp|P63220	1	5	5	5	59	9.1113	3960500000	3138300000	3261400000	3641200000	5608500000	4065400000
sp|P63241	EIF5A	sp|P63241	3	14	14	14	88	16.832	2.0551E+10	2.7881E+10	2.6782E+10	23835000000	29832000000	2.6463E+10
sp|P63244	RACK1	sp|P63244	1	19	19	19	75	35.076	2.9095E+10	2.3788E+10	2.2997E+10	23137000000	21900000000	2.2763E+10
sp|P63261	ACTG1	sp|P63261	1	25	25	2	77	41.792	4.7639E+11	4.798E+11	4.5833E+11	4.9364E+11	4.5277E+11	4.5344E+11
sp|P63272	SUPT4H1	sp|P63272	1	3	3	3	26	13.193	192240000	0	0	176720000	373510000	0
sp|P63279	UBE2I	sp|P63279	1	8	8	8	59	18.007	2449200000	3200900000	2509800000	2114600000	2676200000	2315800000
sp|P63313	TMSB10	sp|P63313	1	1	1	1	32	5.0256	0	0	0	0	0	3396000000
sp|P67775	PPP2CA	sp|P67775	2	11	11	10	52	35.594	3971300000	3846000000	3828800000	3951000000	4030800000	4174900000
sp|P67809	YBX1	sp|P67809	2	14	14	10	66	35.924	1.445E+10	1.2048E+10	1.1093E+10	12220000000	12837000000	1.4205E+10
sp|P67812	SEC11A	sp|P67812	1	4	4	4	20	20.625	277620000	460360000	310040000	408390000	313390000	370820000
sp|P67870	CSNK2B	sp|P67870	1	7	7	7	41	24.942	1618300000	1923400000	1732000000	1518000000	1866800000	1729100000
sp|P67936	TPM4	sp|P67936	2	17	17	9	51	28.521	2.9632E+10	2.5656E+10	2.8136E+10	22862000000	26765000000	2.6199E+10
sp|P68032	ACTC1	sp|P68032	4	20	7	7	68	42.019	5.663E+10	8.4529E+10	7.2001E+10	62548000000	72128000000	5.3062E+10
sp|P68036	UBE2L3	sp|P68036	2	9	9	9	70	17.861	2151700000	2321400000	3038300000	2704800000	2479600000	3282300000
sp|P68104	EEF1A1	sp|P68104	2	29	29	18	70	50.14	1.27E+11	1.4078E+11	1.4961E+11	1.3089E+11	1.0613E+11	1.2766E+11
sp|P68363	TUBA1B	sp|P68363	1	20	4	0	61	50.151	1.9071E+10	1.62E+10	1.3236E+10	16111000000	10682000000	1.2465E+10
sp|P68366	TUBA4A	sp|P68366	1	19	4	4	57	49.924	5411900000	6344400000	6436400000	8933300000	5445600000	6588900000
sp|P68371	TUBB4B	sp|P68371	1	23	23	1	74	49.83	1.0327E+11	1.0727E+11	1.1066E+11	97510000000	90013000000	9.3756E+10
sp|P68400	CSNK2A1	sp|P68400	2	14	14	14	54	45.143	1097700000	1081500000	1017800000	850300000	843900000	1033500000
sp|P68402	PAFAH1B	sp|P68402	1	3	3	3	22	25.569	402090000	401630000	500340000	385060000	238620000	268300000
sp|P68431	HIST1H3A	sp|P68431	3	5	5	1	46	15.404	3.2676E+10	5.6493E+10	4.7194E+10	52270000000	56705000000	5.8558E+10
sp|P78310	CXADR	sp|P78310	1	2	2	2	9.3	40.029	102630000	121550000	206050000	239470000	81384000	72007000
sp|P78316	NOP14	sp|P78316	1	9	9	9	14	97.667	377800000	470820000	516850000	0	0	368930000
sp|P78318	IGBP1	sp|P78318	1	8	8	8	31	39.221	299750000	162340000	391660000	242360000	280040000	222010000
sp|P78330	PSPH	sp|P78330	1	7	7	7	33	25.007	1359100000	1339900000	1214200000	1289300000	1372100000	1213400000
sp|P78344	EIF4G2	sp|P78344	1	27	27	27	35	102.36	3906800000	3649700000	3719000000	4524300000	3715200000	4389700000
sp|P78346	RPP30	sp|P78346	1	8	8	8	43	29.321	690840000	572870000	546580000	557840000	592160000	576780000
sp|P78347	GTF2I	sp|P78347	3	24	24	24	32	112.42	3208900000	3111800000	2906700000	2726600000	2861000000	2837900000
sp|P78357	CNTNAP1	sp|P78357	1	2	2	2	2.8	156.26	0	0	0	0	0	104930000
sp|P78362	SRPK2	sp|P78362	1	4	3	3	9.4	77.526	0	0	0	0	58913000	0
sp|P78371	CCT2	sp|P78371	1	34	34	34	71	57.488	2.1415E+10	2.6209E+10	2.0747E+10	21275000000	20974000000	2.1106E+10
sp|P78381	SLC35A2	sp|P78381	1	3	3	3	19	41.307	0	0	0	112130000	0	0
sp|P78406	RAE1	sp|P78406	1	12	12	12	56	40.968	960750000	1004100000	863240000	1205300000	1173500000	945040000
sp|P78417	GSTO1	sp|P78417	1	14	14	14	60	27.566	2569300000	3523000000	2982200000	2435700000	2733400000	2978600000
sp|P78527	PRKDC	sp|P78527	1	##	135	##	37	469.08	2.6474E+10	2.6105E+10	2.6624E+10	25656000000	25089000000	2.6518E+10
sp|P78537	BLOC1S1	sp|P78537	1	3	3	3	21	17.262	0	31122000	0	0	0	0
sp|P80217	IFI35	sp|P80217	1	2	2	2	8.7	31.546	65602000	59215000	68341000	80467000	81043000	85936000

sp|P80297	MT1X	sp|P80297	1	3	1	1	54	6.0683	0	0	0	0	0	0
sp|P80303	NUCB2	sp|P80303	1	6	6	6	18	50.222	325040000	327600000	345320000	425990000	408110000	378240000
sp|P80723	BASP1	sp|P80723	1	15	15	15	85	22.693	6.122E+10	5.717E+10	5.1724E+10	53602000000	55916000000	5.3041E+10
sp|P82094	TMF1	sp|P82094	1	2	2	2	2.3	122.84	0	0	0	0	0	48908000
sp|P82650	MRPS22	sp|P82650	1	7	7	7	26	41.28	303760000	272610000	212030000	277740000	255620000	241670000
sp|P82663	MRPS25	sp|P82663	1	1	1	1	12	20.116	0	0	0	0	0	43485000
sp|P82664	MRPS10	sp|P82664	1	1	1	1	9	22.999	165600000	92445000	0	165220000	156680000	0
sp|P82673	MRPS35	sp|P82673	1	6	6	6	30	36.844	289900000	240130000	261180000	256570000	245390000	175050000
sp|P82675	MRPS5	sp|P82675	1	5	5	5	12	48.006	166040000	201410000	240980000	198690000	263030000	0
sp|P82909	MRPS36	sp|P82909	1	5	5	5	69	11.466	195070000	347930000	0	102890000	196980000	152720000
sp|P82912	MRPS11	sp|P82912	1	1	1	1	15	20.616	0	0	0	0	0	75908000
sp|P82921	MRPS21	sp|P82921	1	1	1	1	16	10.688	0	0	0	0	0	42753000
sp|P82930	MRPS34	sp|P82930	1	6	6	6	43	25.65	336730000	411030000	283160000	256060000	338510000	339960000
sp|P82932	MRPS6	sp|P82932	1	3	3	3	23	14.226	209400000	198620000	198080000	187160000	0	199820000
sp|P82933	MRPS9	sp|P82933	1	9	9	9	31	45.834	562080000	555940000	465010000	429290000	439920000	495020000
sp|P82970	HMGN5	sp|P82970	1	3	3	3	14	31.524	429730000	309820000	0	352930000	404600000	441150000
sp|P82979	SARNP	sp|P82979	1	8	8	8	42	23.671	3993600000	2798500000	3842000000	3527600000	3273600000	3840500000
sp|P83436	COG7	sp|P83436	1	4	4	4	6.8	86.343	0	0	0	0	177680000	0
sp|P83731	RPL24	sp|P83731	1	5	5	5	31	17.779	5939000000	5717000000	6921300000	6184400000	5775600000	7187900000
sp|P83876	TXNL4A	sp|P83876	1	5	5	5	38	16.786	491930000	0	0	0	412810000	0
sp|P83916	CBX1	sp|P83916	1	4	3	3	40	21.418	219160000	435160000	312670000	221710000	185060000	170500000
sp|P84085	ARF5	sp|P84085	1	8	3	3	53	20.529	592540000	619140000	738790000	626200000	657130000	874360000
sp|P84090	ERH	sp|P84090	1	4	4	4	44	12.259	4092600000	3530600000	4036000000	4004800000	4963800000	4468000000
sp|P84095	RHOG	sp|P84095	1	9	9	8	61	21.308	1257400000	1088600000	1167800000	1236700000	1188000000	1238600000
sp|P84098	RPL19	sp|P84098	1	7	7	7	27	23.466	6375500000	7437600000	6807100000	5681900000	7030200000	6524200000
sp|P84101	SERF2	sp|P84101	1	3	3	3	31	6.8998	694640000	225460000	298150000	227450000	229150000	200360000
sp|P84103	SRSF3	sp|P84103	1	7	7	6	35	19.329	3117800000	2888200000	3285800000	3048800000	3737800000	4041000000
sp|P84243	H3F3A	sp|P84243	1	5	1	1	46	15.328	0	0	0	0	0	26539000
sp|P85037	FOXK1	sp|P85037	2	2	2	2	1.9	75.456	145110000	112150000	146180000	135210000	105820000	131610000
sp|P86791	CCZ1	sp|P86791	2	1	1	1	3.5	55.866	0	0	0	0	0	45881000
sp|P98082	DAB2	sp|P98082	1	7	7	7	12	82.447	542880000	463970000	547870000	333450000	364090000	563360000
sp|P98160	HSPG2	sp|P98160	1	1	1	1	0.3	468.83	0	0	0	0	0	30187000
sp|P98175	RBM10	sp|P98175	1	11	11	11	14	103.53	283760000	290520000	283700000	260600000	362450000	283710000
sp|P98179	RBM3	sp|P98179	1	4	4	4	27	17.17	938500000	1104500000	1169600000	868440000	538980000	945850000
sp|P98194	ATP2C1	sp|P98194	1	5	5	5	9.2	100.58	0	208180000	0	0	194130000	209260000
sp|P99999	CYCS	sp|P99999	2	7	7	7	56	11.749	2266300000	2473100000	1914300000	1739200000	1690400000	1880600000
sp|Q00059	TFAM	sp|Q00059	1	7	7	7	27	29.096	331230000	323390000	339940000	398080000	653190000	403810000
sp|Q00169	PITPNA	sp|Q00169	1	6	5	5	34	31.806	275880000	209870000	290750000	248930000	248020000	175130000
sp|Q00325	SLC25A3	sp|Q00325	1	12	12	12	32	40.094	8191500000	9635300000	6734200000	8668500000	6883100000	6730400000

sp|Q0034	HDLBP	sp|Q0034	1	29	29	29	31	141.45	2847800000	3169500000	3139900000	3045000000	2903200000	2724400000
sp|Q0040	GTF2B	sp|Q0040	1	3	3	3	17	34.833	212630000	197610000	179060000	196910000	205780000	204080000
sp|Q0053	CDK6	sp|Q0053	1	10	10	10	38	36.938	829540000	623590000	648070000	584130000	494610000	527520000
sp|Q0053	CDK5	sp|Q0053	1	2	2	2	8.9	33.304	46523000	62835000	62323000	64265000	61741000	63787000
sp|Q0057	PURA	sp|Q0057	1	4	4	4	27	34.91	347050000	472520000	294170000	284350000	363270000	329590000
sp|Q0058	CDC42EP	sp|Q0058	1	2	2	2	11	40.294	0	0	0	0	0	31017000
sp|Q0061	CLTC	sp|Q0061	2	72	72	72	50	191.61	4.9506E+10	4.6891E+10	4.6821E+10	49507000000	43032000000	4.6563E+10
sp|Q0061	HSF1	sp|Q0061	1	1	1	1	4	57.26	0	0	0	47391000	49427000	0
sp|Q0065	NFKB2	sp|Q0065	1	6	6	6	9.4	96.748	127020000	101770000	146450000	195410000	192640000	204170000
sp|Q0068	FKBP3	sp|Q0068	1	8	8	8	44	25.177	2331700000	1591800000	2008300000	1712500000	2217200000	2041700000
sp|Q0076	REEP5	sp|Q0076	1	5	5	5	20	21.493	2174600000	2179800000	1899600000	2128000000	2006100000	2279900000
sp|Q0079	SORD	sp|Q0079	1	14	14	14	58	38.324	2480600000	2485900000	2801300000	2536300000	2266100000	2157500000
sp|Q0083	HNRNPU	sp|Q0083	1	32	32	32	42	90.583	3.1116E+10	3.1115E+10	3.252E+10	29919000000	30156000000	3.2734E+10
sp|Q0108	SPTBN1	sp|Q0108	2	##	107	##	58	274.61	2.8994E+10	2.9165E+10	2.8172E+10	23424000000	22718000000	2.5606E+10
sp|Q0108	TIAL1	sp|Q0108	1	8	8	5	33	41.59	672440000	685330000	667570000	693220000	717570000	699220000
sp|Q0110	SET	sp|Q0110	2	8	8	8	33	33.488	1.8853E+10	1.7606E+10	1.5785E+10	16586000000	16862000000	1.601E+10
sp|Q0113	SRSF2	sp|Q0113	2	6	6	6	29	25.476	3998100000	3814300000	3357200000	3627700000	4458300000	3910200000
sp|Q0143	AMPD2	sp|Q0143	1	8	8	8	12	100.69	245260000	243190000	258250000	233680000	201890000	219010000
sp|Q0146	FABP5	sp|Q0146	2	10	10	10	72	15.164	8941700000	8540000000	6862300000	8060100000	9357500000	8388700000
sp|Q0151	CAP1	sp|Q0151	1	18	18	17	46	51.901	1.5861E+10	1.8562E+10	1.6225E+10	14792000000	15217000000	1.9951E+10
sp|Q0158	HMGCS1	sp|Q0158	2	11	11	11	26	57.293	782280000	788900000	586790000	426270000	561680000	502940000
sp|Q0162	IFITM3	sp|Q0162	4	3	3	3	49	14.632	1114100000	951350000	1347700000	1340000000	0	0
sp|Q0165	SLC7A5	sp|Q0165	2	7	7	6	14	55.01	4171800000	6971500000	3686500000	4572600000	5690800000	5252500000
sp|Q0165	DR1	sp|Q0165	1	3	3	3	25	19.443	409470000	426980000	373210000	286150000	447640000	458220000
sp|Q0166	TFAP4	sp|Q0166	1	2	2	2	7.4	38.725	0	68567000	0	0	0	0
sp|Q0178	EXOSC10	sp|Q0178	1	20	20	20	29	100.83	1038000000	1041000000	973990000	1067300000	1033600000	1063300000
sp|Q0180	OTUD4	sp|Q0180	1	3	3	3	4.4	124.04	0	0	0	0	0	50018000
sp|Q0181	PFKP	sp|Q0181	1	28	28	25	43	85.595	1.1414E+10	1.0028E+10	1.1647E+10	11679000000	9840200000	1.0636E+10
sp|Q0183	XPC	sp|Q0183	1	3	3	3	4.5	105.95	0	0	0	53071000	0	0
sp|Q0184	EWSR1	sp|Q0184	1	6	6	6	13	68.477	1883700000	1443400000	1636300000	1682100000	1644700000	1166200000
sp|Q0196	OCRL	sp|Q0196	1	1	1	1	2.3	104.2	0	0	0	0	0	30669000
sp|Q0197	PLCB3	sp|Q0197	1	12	12	12	15	138.8	334440000	393710000	476950000	446360000	394660000	496340000
sp|Q0199	TAGLN	sp|Q0199	1	13	13	13	69	22.611	4772900000	5685100000	4215500000	3373000000	4231100000	3768400000
sp|Q0641	MEF2C	sp|Q0641	3	1	1	1	2.3	51.221	0	45540000	0	0	0	0
sp|Q0212	DHODH	sp|Q0212	1	3	3	3	11	42.867	0	0	121780000	115150000	0	0
sp|Q0221	OGDH	sp|Q0221	2	31	31	31	40	115.93	2912200000	2881400000	2672400000	2832600000	2566100000	2571900000
sp|Q0224	KIF23	sp|Q0224	1	10	10	10	17	110.06	380830000	413940000	437670000	491830000	408340000	415170000
sp|Q0233	BDH1	sp|Q0233	1	1	1	1	7.6	38.157	0	0	0	0	0	23179000
sp|Q0238	COL7A1	sp|Q0238	1	4	4	4	2.7	295.22	128500000	217660000	232420000	158920000	140760000	202170000

<table>
<tr><th></th><th></th><th></th><th></th><th></th><th></th><th></th><th></th><th></th><th></th><th></th><th></th><th></th><th></th><th></th></tr>
<tr><td>sp|Q0244</td><td>SP3</td><td>sp|Q0244</td><td>1</td><td>2</td><td>2</td><td>2</td><td>5.6</td><td>81.924</td><td>0</td><td>0</td><td>0</td><td>0</td><td>0</td><td>23296000</td></tr>
<tr><td>sp|Q0253</td><td>HIST1H1A</td><td>sp|Q0253</td><td>1</td><td>4</td><td>2</td><td>2</td><td>24</td><td>21.842</td><td>0</td><td>0</td><td>0</td><td>0</td><td>186380000</td><td>0</td></tr>
<tr><td>sp|Q0254</td><td>RPL18A</td><td>sp|Q0254</td><td>1</td><td>7</td><td>7</td><td>7</td><td>35</td><td>20.762</td><td>7197400000</td><td>7319200000</td><td>6705100000</td><td>7828400000</td><td>7348600000</td><td>7899600000</td></tr>
<tr><td>sp|Q0275</td><td>MAP2K1</td><td>sp|Q0275</td><td>1</td><td>6</td><td>4</td><td>4</td><td>20</td><td>43.439</td><td>524180000</td><td>552260000</td><td>524190000</td><td>515190000</td><td>573680000</td><td>589300000</td></tr>
<tr><td>sp|Q0279</td><td>FKBP4</td><td>sp|Q0279</td><td>1</td><td>32</td><td>32</td><td>32</td><td>74</td><td>51.804</td><td>1.6134E+10</td><td>1.8775E+10</td><td>1.8083E+10</td><td>18458000000</td><td>18547000000</td><td>2.0097E+10</td></tr>
<tr><td>sp|Q0280</td><td>PLOD1</td><td>sp|Q0280</td><td>1</td><td>18</td><td>18</td><td>18</td><td>34</td><td>83.549</td><td>2907100000</td><td>3243800000</td><td>3260500000</td><td>3267700000</td><td>3064600000</td><td>2895800000</td></tr>
<tr><td>sp|Q0281</td><td>NUCB1</td><td>sp|Q0281</td><td>1</td><td>12</td><td>12</td><td>12</td><td>33</td><td>53.879</td><td>856390000</td><td>1026500000</td><td>900720000</td><td>674730000</td><td>721770000</td><td>803870000</td></tr>
<tr><td>sp|Q0287</td><td>RPL6</td><td>sp|Q0287</td><td>1</td><td>14</td><td>14</td><td>14</td><td>40</td><td>32.728</td><td>1.6436E+10</td><td>1.5707E+10</td><td>1.5905E+10</td><td>16765000000</td><td>16937000000</td><td>1.6449E+10</td></tr>
<tr><td>sp|Q0288</td><td>TOP2B</td><td>sp|Q0288</td><td>1</td><td>22</td><td>12</td><td>12</td><td>15</td><td>183.26</td><td>398260000</td><td>396290000</td><td>416280000</td><td>356780000</td><td>309100000</td><td>399980000</td></tr>
<tr><td>sp|Q0295</td><td>AKAP12</td><td>sp|Q0295</td><td>1</td><td>12</td><td>12</td><td>12</td><td>13</td><td>191.48</td><td>224440000</td><td>275440000</td><td>178780000</td><td>207140000</td><td>277230000</td><td>207410000</td></tr>
<tr><td>sp|Q0297</td><td>SLC25A1</td><td>sp|Q0297</td><td>1</td><td>9</td><td>9</td><td>9</td><td>32</td><td>34.061</td><td>1239300000</td><td>1324700000</td><td>1314000000</td><td>1532000000</td><td>1886000000</td><td>1774300000</td></tr>
<tr><td>sp|Q0300</td><td>DST</td><td>sp|Q0300</td><td>1</td><td>40</td><td>40</td><td>40</td><td>7</td><td>860.65</td><td>1806100000</td><td>1769600000</td><td>1778600000</td><td>1879500000</td><td>1576600000</td><td>1807100000</td></tr>
<tr><td>sp|Q0301</td><td>GSTM4</td><td>sp|Q0301</td><td>1</td><td>7</td><td>2</td><td>2</td><td>43</td><td>25.561</td><td>0</td><td>0</td><td>0</td><td>0</td><td>8165900</td><td>0</td></tr>
<tr><td>sp|Q0311</td><td>MLLT1</td><td>sp|Q0311</td><td>1</td><td>2</td><td>2</td><td>2</td><td>3.9</td><td>62.055</td><td>151750000</td><td>0</td><td>166600000</td><td>110600000</td><td>0</td><td>0</td></tr>
<tr><td>sp|Q0313</td><td>CAV1</td><td>sp|Q0313</td><td>2</td><td>7</td><td>7</td><td>7</td><td>41</td><td>20.471</td><td>3051000000</td><td>3681900000</td><td>3417900000</td><td>2797700000</td><td>3161300000</td><td>3361000000</td></tr>
<tr><td>sp|Q0316</td><td>TNFAIP2</td><td>sp|Q0316</td><td>1</td><td>2</td><td>2</td><td>2</td><td>5</td><td>72.66</td><td>0</td><td>82267000</td><td>0</td><td>89241000</td><td>53699000</td><td>38445000</td></tr>
<tr><td>sp|Q0325</td><td>LMNB2</td><td>sp|Q0325</td><td>1</td><td>17</td><td>15</td><td>15</td><td>29</td><td>69.948</td><td>1343700000</td><td>1361200000</td><td>1679600000</td><td>1519700000</td><td>1580400000</td><td>1455100000</td></tr>
<tr><td>sp|Q0340</td><td>PLAUR</td><td>sp|Q0340</td><td>1</td><td>2</td><td>2</td><td>2</td><td>8.4</td><td>36.978</td><td>0</td><td>0</td><td>0</td><td>0</td><td>0</td><td>99061000</td></tr>
<tr><td>sp|Q0370</td><td>CEBPZ</td><td>sp|Q0370</td><td>1</td><td>11</td><td>11</td><td>11</td><td>15</td><td>120.97</td><td>545410000</td><td>672970000</td><td>590270000</td><td>559560000</td><td>611950000</td><td>623860000</td></tr>
<tr><td>sp|Q0420</td><td>RELA</td><td>sp|Q0420</td><td>1</td><td>5</td><td>5</td><td>5</td><td>13</td><td>60.218</td><td>279160000</td><td>244750000</td><td>264380000</td><td>282610000</td><td>331310000</td><td>273780000</td></tr>
<tr><td>sp|Q0432</td><td>UBXN1</td><td>sp|Q0432</td><td>1</td><td>9</td><td>9</td><td>9</td><td>42</td><td>33.325</td><td>986050000</td><td>1388200000</td><td>1098100000</td><td>1268700000</td><td>1232600000</td><td>922290000</td></tr>
<tr><td>sp|Q0444</td><td>GBE1</td><td>sp|Q0444</td><td>1</td><td>18</td><td>18</td><td>18</td><td>33</td><td>80.473</td><td>2722800000</td><td>2207100000</td><td>2520200000</td><td>2372600000</td><td>2378400000</td><td>2641100000</td></tr>
<tr><td>sp|Q0463</td><td>EIF4G1</td><td>sp|Q0463</td><td>2</td><td>47</td><td>47</td><td>42</td><td>35</td><td>175.49</td><td>1.3364E+10</td><td>1.466E+10</td><td>1.2043E+10</td><td>12569000000</td><td>11741000000</td><td>1.2484E+10</td></tr>
<tr><td>sp|Q0472</td><td>TLE3</td><td>sp|Q0472</td><td>4</td><td>6</td><td>6</td><td>6</td><td>8.7</td><td>83.416</td><td>852640000</td><td>459010000</td><td>740200000</td><td>347370000</td><td>944110000</td><td>600990000</td></tr>
<tr><td>sp|Q0476</td><td>GLO1</td><td>sp|Q0476</td><td>1</td><td>8</td><td>8</td><td>8</td><td>35</td><td>20.777</td><td>2174000000</td><td>1733200000</td><td>1700700000</td><td>1765400000</td><td>2254400000</td><td>1712600000</td></tr>
<tr><td>sp|Q0482</td><td>AKR1C1</td><td>sp|Q0482</td><td>1</td><td>16</td><td>3</td><td>0</td><td>59</td><td>36.788</td><td>5649800000</td><td>5770100000</td><td>4290500000</td><td>3596600000</td><td>4209100000</td><td>4415800000</td></tr>
<tr><td>sp|Q0483</td><td>SSBP1</td><td>sp|Q0483</td><td>1</td><td>6</td><td>6</td><td>6</td><td>49</td><td>17.259</td><td>1966100000</td><td>2344100000</td><td>1991000000</td><td>2348800000</td><td>2573800000</td><td>2274600000</td></tr>
<tr><td>sp|Q0491</td><td>YWHAH</td><td>sp|Q0491</td><td>1</td><td>12</td><td>10</td><td>10</td><td>54</td><td>28.218</td><td>4403100000</td><td>4638500000</td><td>3538200000</td><td>3687000000</td><td>4196000000</td><td>3841400000</td></tr>
<tr><td>sp|Q0494</td><td>PLP2</td><td>sp|Q0494</td><td>1</td><td>2</td><td>2</td><td>2</td><td>18</td><td>16.691</td><td>1514600000</td><td>1281900000</td><td>0</td><td>1344800000</td><td>1357600000</td><td>0</td></tr>
<tr><td>sp|Q0504</td><td>CSTF1</td><td>sp|Q0504</td><td>1</td><td>10</td><td>10</td><td>10</td><td>39</td><td>48.357</td><td>857900000</td><td>646730000</td><td>625150000</td><td>672260000</td><td>775530000</td><td>633400000</td></tr>
<tr><td>sp|Q0520</td><td>PTPN12</td><td>sp|Q0520</td><td>1</td><td>6</td><td>6</td><td>6</td><td>14</td><td>88.105</td><td>0</td><td>0</td><td>0</td><td>0</td><td>0</td><td>97544000</td></tr>
<tr><td>sp|Q0539</td><td>PTK2</td><td>sp|Q0539</td><td>1</td><td>4</td><td>4</td><td>4</td><td>4.9</td><td>119.23</td><td>80452000</td><td>102150000</td><td>84935000</td><td>0</td><td>0</td><td>0</td></tr>
<tr><td>sp|Q0551</td><td>SRSF11</td><td>sp|Q0551</td><td>1</td><td>4</td><td>4</td><td>4</td><td>14</td><td>53.542</td><td>630770000</td><td>525390000</td><td>511310000</td><td>681840000</td><td>602700000</td><td>527860000</td></tr>
<tr><td>sp|Q0563</td><td>EEF1A2</td><td>sp|Q0563</td><td>1</td><td>20</td><td>9</td><td>9</td><td>68</td><td>50.47</td><td>706700000</td><td>694160000</td><td>645970000</td><td>562330000</td><td>482820000</td><td>393240000</td></tr>
<tr><td>sp|Q0568</td><td>CALD1</td><td>sp|Q0568</td><td>3</td><td>23</td><td>23</td><td>23</td><td>29</td><td>93.23</td><td>6048500000</td><td>4942100000</td><td>5004400000</td><td>4935000000</td><td>5069400000</td><td>4493300000</td></tr>
<tr><td>sp|Q0593</td><td>FPGS</td><td>sp|Q0593</td><td>1</td><td>4</td><td>4</td><td>4</td><td>11</td><td>64.608</td><td>75688000</td><td>19246000</td><td>53743000</td><td>107100000</td><td>45168000</td><td>0</td></tr>
<tr><td>sp|Q05D3</td><td>CTDSPL2</td><td>sp|Q05D3</td><td>1</td><td>3</td><td>3</td><td>3</td><td>6.7</td><td>52.998</td><td>86760000</td><td>0</td><td>0</td><td>76911000</td><td>81787000</td><td>80032000</td></tr>
<tr><td>sp|Q0612</td><td>PTPN11</td><td>sp|Q0612</td><td>1</td><td>7</td><td>7</td><td>7</td><td>14</td><td>68.436</td><td>471630000</td><td>429950000</td><td>443840000</td><td>342720000</td><td>383410000</td><td>451770000</td></tr>
<tr><td>sp|Q0613</td><td>KDSR</td><td>sp|Q0613</td><td>1</td><td>3</td><td>3</td><td>3</td><td>14</td><td>36.187</td><td>0</td><td>166920000</td><td>131600000</td><td>0</td><td>122130000</td><td>0</td></tr>
</table>

sp|Q0620	PPAT	sp|Q0620	1	7	7	7	21	57.398	352400000	344420000	376200000	383920000	474580000	392710000
sp|Q0621	GFPT1	sp|Q0621	1	20	20	17	38	78.806	1723800000	1711500000	1565800000	2025500000	2112000000	2034200000
sp|Q0626	EXOSC9	sp|Q0626	1	6	6	6	18	48.948	384800000	373070000	368260000	303080000	435920000	413920000
sp|Q0632	PSME1	sp|Q0632	1	12	12	12	43	28.723	955930000	982500000	1006500000	993410000	1002600000	662040000
sp|Q0654	GABPA	sp|Q0654	1	5	5	5	20	51.295	213840000	202590000	173090000	175140000	128600000	113880000
sp|Q0658	RING1	sp|Q0658	2	3	3	3	15	42.429	186650000	202650000	148720000	188090000	182350000	206080000
sp|Q0678	FMR1	sp|Q0678	1	8	6	6	16	71.174	151490000	135280000	178460000	163540000	167060000	223850000
sp|Q0683	PRDX1	sp|Q0683	1	16	16	12	73	22.11	1.0597E+11	9.412E+10	8.5051E+10	94321000000	1.0085E+11	9.3037E+10
sp|Q0702	RPL18	sp|Q0702	1	7	7	7	42	21.634	9177800000	1.1543E+10	1.0052E+10	11870000000	12778000000	1.3321E+10
sp|Q0702	C1QBP	sp|Q0702	1	6	6	6	32	31.362	7755300000	6181200000	7358000000	8822900000	8368900000	7548500000
sp|Q0706	CKAP4	sp|Q0706	1	30	30	29	56	66.022	7491800000	6885800000	6887900000	8256800000	8650900000	8880100000
sp|Q0715	TJP1	sp|Q0715	1	14	14	14	13	195.46	679410000	791550000	626420000	600390000	632280000	502850000
sp|Q0766	KHDRBS1	sp|Q0766	2	7	7	6	24	48.227	3384000000	4014800000	5164500000	4309100000	4819500000	5451100000
sp|Q0781	BAX	sp|Q0781	1	5	5	5	37	21.184	392600000	428810000	192740000	337630000	372530000	408350000
sp|Q0782	MCL1	sp|Q0782	1	1	1	1	6	37.337	0	0	0	0	36191000	0
sp|Q0786	POLE	sp|Q0786	1	3	3	3	1.7	261.51	60705000	0	0	52538000	0	0
sp|Q0786	KLC1	sp|Q0786	2	16	16	11	29	65.309	760530000	802610000	1027000000	716420000	765460000	745580000
sp|Q0795	SRSF1	sp|Q0795	1	12	12	12	44	27.744	6490500000	7284200000	6395500000	7051000000	7595600000	6999100000
sp|Q0796	ARHGAP1	sp|Q0796	1	12	12	12	39	50.435	2157100000	1802300000	1934400000	2080400000	1873200000	2195900000
sp|Q0817	SRSF4	sp|Q0817	1	5	2	2	11	56.678	137400000	0	148660000	108100000	0	170850000
sp|Q0820	PPP3CA	sp|Q0820	3	9	9	9	20	58.687	1031800000	1350600000	1043400000	957880000	971190000	854680000
sp|Q0821	DHX9	sp|Q0821	1	46	46	46	48	140.96	1.9682E+10	2.016E+10	2.0112E+10	18576000000	18174000000	1.9062E+10
sp|Q0825	CRYZ	sp|Q0825	1	12	12	12	47	35.206	1649400000	1561600000	1296100000	1451900000	1685400000	1544400000
sp|Q0837	GOLGA3	sp|Q0837	1	7	7	7	7	167.35	0	85663000	112300000	110520000	99095000	121120000
sp|Q0837	GOLGA2	sp|Q0837	1	12	12	12	19	113.08	683680000	681110000	423430000	585980000	446950000	652600000
sp|Q0838	LGALS3B	sp|Q0838	1	10	10	10	27	65.33	826620000	907850000	665950000	978430000	1034800000	1135700000
sp|Q0849	PDE4D	sp|Q0849	1	3	3	3	4.4	91.114	0	0	0	0	0	121580000
sp|Q0872	CD47	sp|Q0872	1	2	2	2	5.9	35.213	201990000	279170000	213740000	262540000	308650000	212270000
sp|Q0875	PPID	sp|Q0875	1	9	9	9	26	40.763	653950000	834950000	828860000	858920000	807040000	831460000
sp|Q0894	SSRP1	sp|Q0894	1	20	20	20	36	81.074	3900900000	3929800000	3909200000	3729600000	4155000000	4382300000
sp|Q08AD	CAMSAP2	sp|Q08AD	1	4	4	4	5.2	168.09	719930000	676800000	0	0	0	0
sp|Q08AF	SLFN5	sp|Q08AF	1	3	3	3	3.9	101.05	0	0	0	0	139970000	0
sp|Q08AG	MZT1	sp|Q08AG	1	2	2	2	45	8.4787	152780000	0	0	0	162150000	121010000
sp|Q08AH	ACSM1	sp|Q08AH	1	1	1	1	1.6	65.272	0	0	0	0	0	139270000
sp|Q08AM	VAC14	sp|Q08AM	1	3	3	3	6.6	87.972	35777000	0	0	0	35472000	49478000
sp|Q08J2	NSUN2	sp|Q08J2	1	40	40	40	68	86.47	1.1315E+10	1.1067E+10	1.0122E+10	10735000000	11523000000	1.2967E+10
sp|Q0902	RBBP4	sp|Q0902	1	12	8	8	55	47.655	4438200000	5471700000	4159800000	3294000000	4142900000	3295900000
sp|Q0916	NCBP1	sp|Q0916	1	16	16	16	26	91.838	1549700000	1565200000	1442900000	1212100000	1269400000	1319200000
sp|Q0932	MGAT5	sp|Q0932	1	1	1	1	1.2	84.542	0	0	0	0	0	327510000

sp|Q0966	AHNAK	sp|Q0966	2	##	241	##	69	629.09	1.0797E+11	1.0172E+11	1.0332E+11	92659000000	97008000000	9.3449E+10
sp|Q0P6D	CCDC15	sp|Q0P6D	1	1	1	1	0.8	110.48	125780000	0	0	0	0	0
sp|Q0VDF	HSPA14	sp|Q0VDF	1	7	7	7	27	54.794	199610000	215230000	307350000	206110000	197920000	156260000
sp|Q0VGL	LAMTOR4	sp|Q0VGL	1	1	1	1	10	10.741	0	0	0	0	61658000	0
sp|Q1046	MGAT2	sp|Q1046	1	2	2	2	7.2	51.55	0	0	0	0	115400000	0
sp|Q1047	GALNT2	sp|Q1047	2	23	23	23	50	64.732	5452900000	5204000000	5244400000	6440000000	5039500000	6420100000
sp|Q1047	GALNT1	sp|Q1047	2	8	8	8	16	64.218	445150000	412150000	482810000	365150000	447660000	497400000
sp|Q1056	AP1B1	sp|Q1056	1	19	9	9	25	104.64	839320000	777650000	972720000	847180000	644760000	649130000
sp|Q1057	CPSF1	sp|Q1057	1	11	11	11	9.5	160.88	455820000	762270000	603560000	367580000	376490000	496890000
sp|Q1058	BST2	sp|Q1058	1	3	3	3	19	19.769	1044300000	1450000000	1100200000	1130500000	962810000	856920000
sp|Q1071	PMPCA	sp|Q1071	1	14	14	14	31	58.252	1178100000	1500000000	1170600000	1294300000	1287200000	1412400000
sp|Q1276	SCRN1	sp|Q1276	1	4	4	4	12	46.382	276780000	0	234900000	0	0	280790000
sp|Q1276	WASHC5	sp|Q1276	1	6	6	6	7.4	134.28	189260000	251950000	155670000	118980000	0	0
sp|Q1276	NUP160	sp|Q1276	1	16	16	16	16	162.12	970730000	932370000	893690000	880050000	898150000	993400000
sp|Q1278	TBL3	sp|Q1278	1	15	15	15	29	89.034	1358600000	1159500000	1317700000	1386400000	1774700000	1469200000
sp|Q1278	GTF3C1	sp|Q1278	1	10	10	10	7.9	238.87	249900000	276630000	249270000	243060000	245780000	216240000
sp|Q1279	TWF1	sp|Q1279	1	12	9	9	35	40.282	1119100000	1270700000	1190400000	863870000	998730000	1303000000
sp|Q1279	ASPH	sp|Q1279	1	22	22	22	40	85.862	3075700000	3117700000	2984800000	3174500000	3402500000	3795600000
sp|Q1280	TFCP2	sp|Q1280	2	5	5	4	15	57.255	205570000	200710000	368780000	254080000	253190000	284770000
sp|Q1280	AKAP13	sp|Q1280	1	5	5	5	3.4	307.55	198400000	0	159040000	0	0	0
sp|Q1282	SMARCB1	sp|Q1282	1	8	8	8	38	44.141	342830000	290210000	350190000	492180000	403620000	241400000
sp|Q1284	FSTL1	sp|Q1284	1	1	1	1	3.9	34.985	0	0	0	0	30660000	0
sp|Q1284	STX4	sp|Q1284	1	5	5	5	28	34.18	281450000	335460000	246730000	303380000	291000000	362730000
sp|Q1284	GRSF1	sp|Q1284	1	13	13	13	36	53.126	862500000	789470000	839810000	787500000	897450000	793500000
sp|Q1287	CHD3	sp|Q1287	1	8	2	2	6.1	226.59	0	0	0	0	54697000	0
sp|Q1287	SF3A3	sp|Q1287	1	12	12	12	28	58.848	2055000000	2526700000	2387800000	2426300000	2355900000	2258700000
sp|Q1288	TP53BP1	sp|Q1288	1	30	30	30	26	213.57	1587700000	1508000000	1944900000	1324000000	1541900000	1509400000
sp|Q1289	TMEM115	sp|Q1289	1	1	1	1	6.6	38.197	0	0	0	0	0	37250000
sp|Q1290	AIMP1	sp|Q1290	1	13	13	13	61	34.352	3665900000	3402000000	3883100000	4004900000	3491800000	4347100000
sp|Q1290	ILF2	sp|Q1290	1	15	15	15	59	43.062	5595500000	4496000000	4843400000	5464700000	6124100000	5437100000
sp|Q1290	ILF3	sp|Q1290	1	27	27	24	43	95.337	1.0629E+10	1.1821E+10	1.1568E+10	10246000000	11390000000	1.0955E+10
sp|Q1290	LMAN2	sp|Q1290	1	12	12	12	47	40.228	2472700000	2165700000	2278100000	2193500000	3125000000	2556800000
sp|Q1291	PTPRJ	sp|Q1291	1	2	2	2	2.1	145.94	48565000	85305000	0	40798000	40570000	0
sp|Q1293	TRAP1	sp|Q1293	1	26	26	26	44	80.109	6787000000	7836500000	8052200000	8258700000	7253500000	8286300000
sp|Q1293	TRAF2	sp|Q1293	1	2	2	2	4	55.859	0	155230000	0	0	0	0
sp|Q1296	TAF10	sp|Q1296	1	1	1	1	12	21.711	0	0	0	0	0	36763000
sp|Q1296	MYO1E	sp|Q1296	2	22	22	22	25	127.06	1811100000	1828100000	1520400000	1712400000	1740300000	1696100000
sp|Q1297	PPP1R8	sp|Q1297	1	7	7	7	39	38.478	511010000	377540000	368800000	337960000	381870000	384440000
sp|Q1297	PTP4A2	sp|Q1297	1	4	1	1	29	19.127	0	0	0	0	0	69483000

sp|Q1297	ABR	sp|Q1297	1	2	2	2	2.6	97.597	0	0	0	0	0	61106000
sp|Q1299	CSTF3	sp|Q1299	1	8	8	8	15	82.921	0	0	174260000	176690000	177740000	195930000
sp|Q1301	ECH1	sp|Q1301	1	8	8	8	33	35.816	1234700000	1308800000	1319300000	1302600000	1539300000	1279200000
sp|Q1301	MLLT11	sp|Q1301	1	3	3	3	54	10.061	128270000	148900000	212250000	157990000	235100000	58223000
sp|Q1304	CDC16	sp|Q1304	1	6	6	6	13	71.655	190640000	220130000	221720000	140180000	208050000	236460000
sp|Q1304	STK4	sp|Q1304	1	1	1	1	3.3	55.63	0	0	0	0	0	19361000
sp|Q1304	FLII	sp|Q1304	1	24	24	24	26	144.75	2103600000	2212000000	1912200000	2076400000	2270100000	1951400000
sp|Q1304	TRIM32	sp|Q1304	1	3	3	3	5.7	71.988	0	0	41311000	0	44611000	0
sp|Q1305	COASY	sp|Q1305	1	13	13	13	38	62.328	936440000	879820000	1199100000	981640000	832250000	834900000
sp|Q1308	MRPL28	sp|Q1308	1	3	3	3	21	30.156	116630000	104410000	104990000	107650000	83149000	113210000
sp|Q1308	ACACA	sp|Q1308	1	36	36	32	23	265.55	1835100000	1959000000	2186300000	1829000000	1997300000	1847300000
sp|Q1309	GPS1	sp|Q1309	1	13	13	13	33	55.536	1531900000	1336600000	1497300000	1263200000	1185300000	1306400000
sp|Q1311	CHAF1A	sp|Q1311	1	1	1	1	2.1	106.91	0	0	0	0	41947000	0
sp|Q1311	CHAF1B	sp|Q1311	1	2	2	2	5.9	61.492	0	0	70174000	0	0	0
sp|Q1312	IK	sp|Q1312	1	11	11	11	27	65.601	733960000	777150000	751470000	580400000	701250000	751450000
sp|Q1312	MTAP	sp|Q1312	1	16	16	16	73	31.236	3168700000	3212300000	3563700000	2798500000	3139800000	2886700000
sp|Q1313	PRKAA1	sp|Q1313	2	4	4	4	11	64.009	110120000	111310000	104700000	84804000	104350000	102420000
sp|Q1313	PPFIA1	sp|Q1313	1	6	6	6	7.8	135.78	142630000	0	150610000	91573000	212770000	164310000
sp|Q1314	EIF2B5	sp|Q1314	1	7	7	7	16	80.379	497440000	616670000	540020000	549280000	547790000	680050000
sp|Q1314	TARDBP	sp|Q1314	1	11	11	11	39	44.739	2095800000	2234000000	1669000000	2255100000	2224000000	1891200000
sp|Q1315	HNRNPA	sp|Q1315	1	8	8	8	37	30.84	5289800000	6280100000	4873500000	4751400000	4626000000	4488900000
sp|Q1315	AIMP2	sp|Q1315	1	9	9	9	46	35.348	2193500000	2382700000	2102100000	2321200000	2576000000	2770300000
sp|Q1315	FADD	sp|Q1315	1	2	2	2	21	23.279	73328000	81268000	79562000	0	80806000	0
sp|Q1316	PRDX4	sp|Q1316	1	13	10	10	60	30.54	3762800000	3639800000	4562600000	4370100000	5788600000	5319900000
sp|Q1317	PAK2	sp|Q1317	3	12	12	12	36	58.042	1262700000	1032700000	1444400000	998790000	1001600000	1085000000
sp|Q1318	CBX3	sp|Q1318	1	6	6	5	52	20.811	4653500000	5645900000	4724800000	3972400000	4733500000	5115400000
sp|Q1318	STK3	sp|Q1318	1	2	2	2	4.5	56.3	0	0	0	0	0	26950000
sp|Q1319	STX5	sp|Q1319	1	5	5	5	23	39.672	268250000	202060000	0	214910000	231090000	214260000
sp|Q1320	PSMD2	sp|Q1320	1	33	33	33	49	100.2	6667000000	6713400000	7027400000	7289700000	6054500000	5624800000
sp|Q1320	DDX10	sp|Q1320	1	1	1	1	1.7	100.89	0	0	0	0	9287500	0
sp|Q1321	DNAJC3	sp|Q1321	1	4	4	4	9.1	57.579	153630000	121750000	0	0	160070000	327730000
sp|Q1322	SELENBP	sp|Q1322	1	9	9	9	24	52.39	399770000	554860000	396330000	357930000	283090000	358950000
sp|Q1323	NME3	sp|Q1323	1	3	3	3	25	19.015	220350000	0	140620000	0	0	232870000
sp|Q1324	SRSF9	sp|Q1324	1	6	6	6	31	25.542	1151300000	1074400000	1427400000	967160000	1057700000	829990000
sp|Q1324	SRSF5	sp|Q1324	1	7	6	6	25	31.263	373240000	475120000	403950000	391720000	356240000	327120000
sp|Q1324	SRSF6	sp|Q1324	1	8	8	5	21	39.586	1733700000	2207900000	1910700000	2318600000	2281400000	2463200000
sp|Q1325	MAD2L1	sp|Q1325	1	7	7	7	32	23.51	431310000	432300000	485030000	389620000	343940000	361110000
sp|Q1326	TRIM28	sp|Q1326	1	27	27	27	61	88.549	8619300000	8315500000	8489300000	8778200000	8060500000	8391500000
sp|Q1326	DHRS2	sp|Q1326	1	1	1	1	2.5	29.926	0	0	0	0	0	0

sp|Q1328	G3BP1	sp|Q1328	1	18	18	17	55	52.164	8516900000	7944400000	8566300000	7108100000	6896300000	8367000000
sp|Q1330	KCNAB2	sp|Q1330	1	1	1	1	7.9	41	0	0	0	0	0	42564000
sp|Q1331	PABPC4	sp|Q1331	2	23	16	15	37	70.782	2098600000	1984500000	2298500000	2116500000	2586400000	2941000000
sp|Q1332	IFIT5	sp|Q1332	1	1	1	1	2.1	55.846	0	0	0	0	0	0
sp|Q1333	MTA1	sp|Q1333	1	5	4	3	9.2	80.785	185880000	139680000	0	122190000	137570000	143880000
sp|Q1334	EIF3I	sp|Q1334	1	12	12	12	53	36.501	4842400000	4884300000	4951900000	4570400000	4252800000	4862600000
sp|Q1336	CTBP1	sp|Q1336	1	9	5	5	29	47.535	635350000	401280000	182000000	319710000	442840000	375020000
sp|Q1340	UBE2V1	sp|Q1340	1	6	6	2	44	16.495	1665700000	1241600000	1375500000	1397900000	1457900000	1446300000
sp|Q1340	MRPL49	sp|Q1340	1	3	3	3	24	19.198	583790000	287990000	342470000	330660000	463290000	273560000
sp|Q1340	DYNC1I2	sp|Q1340	1	12	12	12	31	71.456	2068600000	2355300000	2298200000	1839400000	1639500000	2229500000
sp|Q1341	ORC2	sp|Q1341	1	2	2	2	5.2	65.971	0	0	0	23467000	0	0
sp|Q1341	ILK	sp|Q1341	1	15	15	15	38	51.419	1126100000	1307600000	1244100000	1170300000	1454400000	1399500000
sp|Q1342	NNT	sp|Q1342	1	33	33	33	38	113.89	5737000000	5682000000	5086900000	5844000000	5779100000	5917100000
sp|Q1342	SNTB2	sp|Q1342	1	5	5	5	14	57.949	139880000	136840000	133920000	0	147190000	135570000
sp|Q1342	PPIG	sp|Q1342	1	2	2	2	3.2	88.616	0	0	0	97456000	0	0
sp|Q1342	TCOF1	sp|Q1342	1	19	19	19	17	152.1	2490200000	2210000000	2138700000	2338400000	2374300000	2553900000
sp|Q1343	SF3B2	sp|Q1343	1	27	27	27	40	100.23	5556900000	4787400000	5664000000	4376400000	4745900000	4267700000
sp|Q1343	GOLGA4	sp|Q1343	1	6	6	6	3.7	261.14	0	0	0	2360000000	1869300000	0
sp|Q1344	PDAP1	sp|Q1344	1	7	7	7	38	20.63	2285300000	2479900000	1874100000	1995200000	2391200000	1768900000
sp|Q1344	ADAM9	sp|Q1344	1	5	5	5	9.8	90.555	303220000	405130000	231750000	366330000	269670000	292160000
sp|Q1344	TMED1	sp|Q1344	1	3	3	3	17	25.206	366010000	460630000	271070000	365240000	450350000	588030000
sp|Q1345	FKBP5	sp|Q1345	1	4	4	4	14	51.212	140850000	0	131950000	161750000	110320000	0
sp|Q1345	TUSC3	sp|Q1345	1	1	1	1	2.9	39.676	0	0	0	7535500	0	0
sp|Q1346	ROCK1	sp|Q1346	1	7	3	3	6.5	158.17	0	0	0	104090000	0	0
sp|Q1348	SMAD4	sp|Q1348	1	5	5	5	19	60.438	0	150570000	135900000	0	140210000	100420000
sp|Q1349	PICALM	sp|Q1349	2	10	10	10	24	70.754	912170000	743960000	745030000	1022600000	803870000	801530000
sp|Q1350	SQSTM1	sp|Q1350	1	15	15	15	58	47.687	3207000000	3476200000	2910500000	6421600000	6613600000	7259100000
sp|Q1350	MED21	sp|Q1350	1	1	1	1	17	15.564	13440000	11649000	0	13300000	19835000	0
sp|Q1350	MTX1	sp|Q1350	1	5	5	5	16	51.462	287380000	0	329510000	292500000	311850000	0
sp|Q1350	TUBB3	sp|Q1350	1	17	4	4	53	50.432	756750000	451070000	431070000	877920000	384960000	450950000
sp|Q1351	ASAH1	sp|Q1351	1	1	1	1	4.3	44.659	0	0	0	0	0	29619000
sp|Q1352	PRPF4B	sp|Q1352	1	6	6	6	6.2	116.99	154880000	186710000	159660000	131770000	163920000	111650000
sp|Q1352	PIN1	sp|Q1352	2	7	7	7	64	18.243	737260000	807300000	687120000	713220000	896770000	771400000
sp|Q1354	EIF4EBP1	sp|Q1354	1	1	1	1	10	12.58	0	0	0	0	0	71069000
sp|Q1354	EIF4EBP2	sp|Q1354	1	2	2	2	38	12.939	0	0	0	0	0	74730000
sp|Q1354	RIPK1	sp|Q1354	1	1	1	1	1.5	75.93	0	0	0	0	0	28194000
sp|Q1354	HDAC1	sp|Q1354	1	8	5	5	24	55.102	898550000	567690000	800970000	625740000	755540000	811570000
sp|Q1355	CAMK2G	sp|Q1355	1	4	2	2	9.3	62.606	0	0	0	0	0	52888000
sp|Q1355	CAMK2D	sp|Q1355	1	5	5	3	16	56.369	294150000	348900000	208090000	501210000	273120000	354590000

sp|Q13561	DCTN2	sp|Q13561	1	16	16	16	56	44.23	2014900000	2196700000	2025900000	1708500000	1755400000	1672100000
sp|Q13564	NAE1	sp|Q13564	1	9	9	9	28	60.246	687630000	728670000	663740000	645220000	797760000	560210000
sp|Q13573	SNW1	sp|Q13573	1	12	12	12	33	61.494	825240000	969170000	929900000	788380000	882650000	938210000
sp|Q13576	IQGAP2	sp|Q13576	1	26	24	24	25	180.58	928190000	940240000	882510000	889050000	990310000	994400000
sp|Q13586	STIM1	sp|Q13586	1	3	3	3	6.3	77.422	48820000	50855000	65658000	62281000	61688000	77954000
sp|Q13595	TRA2A	sp|Q13595	1	6	5	5	25	32.688	576770000	738530000	551800000	846120000	587500000	587460000
sp|Q13596	SNX1	sp|Q13596	1	9	7	7	19	59.069	653730000	737320000	533650000	628040000	816600000	721640000
sp|Q13601	KRR1	sp|Q13601	1	3	3	3	6.6	43.664	0	100990000	126820000	106790000	0	0
sp|Q13610	PWP1	sp|Q13610	1	7	7	7	17	55.827	247080000	259130000	293580000	235130000	204560000	223480000
sp|Q13614	MTMR2	sp|Q13614	2	4	4	4	7.6	73.38	211360000	208610000	200630000	206540000	139660000	183480000
sp|Q13616	CUL1	sp|Q13616	1	10	10	10	18	89.677	346930000	389550000	349180000	327310000	315880000	387080000
sp|Q13617	CUL2	sp|Q13617	1	11	11	11	20	86.982	437860000	402680000	499250000	341000000	353510000	329820000
sp|Q13618	CUL3	sp|Q13618	1	6	6	6	10	88.929	180020000	225780000	208610000	270580000	148510000	168760000
sp|Q13619	CUL4A	sp|Q13619	1	20	20	14	31	87.679	1337900000	1339100000	1355700000	1119500000	1294800000	1099400000
sp|Q13620	CUL4B	sp|Q13620	1	15	9	9	16	103.98	539260000	680500000	546060000	535910000	506120000	520780000
sp|Q13630	TSTA3	sp|Q13630	1	5	5	5	19	35.892	672520000	763130000	665420000	622990000	705460000	549080000
sp|Q13636	RAB31	sp|Q13636	1	7	7	6	47	21.569	661400000	457920000	504080000	561950000	510390000	586250000
sp|Q13637	RAB32	sp|Q13637	3	10	10	10	53	24.997	1640700000	1417400000	1410600000	1482000000	1434200000	1618600000
sp|Q13641	TPBG	sp|Q13641	1	2	2	2	6.9	46.031	0	0	0	0	0	36088000
sp|Q13642	FHL1	sp|Q13642	1	4	4	4	14	36.263	339200000	283890000	0	207280000	218760000	176430000
sp|Q13643	FHL3	sp|Q13643	1	8	8	8	44	31.192	436490000	584450000	733600000	522750000	587710000	466230000
sp|Q13685	AAMP	sp|Q13685	1	4	4	4	12	46.75	259830000	166820000	206950000	177810000	232220000	0
sp|Q13724	MOGS	sp|Q13724	1	6	6	6	10	91.916	283020000	366110000	322890000	320280000	388890000	336160000
sp|Q13740	ALCAM	sp|Q13740	1	9	9	9	24	65.102	1025600000	1002000000	1240500000	759070000	1018100000	1151200000
sp|Q13751	LAMB3	sp|Q13751	1	4	4	4	7.7	129.57	107940000	155420000	116830000	173920000	94156000	96680000
sp|Q13769	THOC5	sp|Q13769	1	9	9	9	24	78.507	268890000	0	260130000	308970000	207160000	283180000
sp|Q13813	SPTAN1	sp|Q13813	2	##	126	##	65	284.54	3.5328E+10	3.6439E+10	3.5611E+10	30218000000	30396000000	3.2155E+10
sp|Q13823	GNL2	sp|Q13823	1	9	9	9	15	83.654	182060000	157890000	198800000	153000000	184800000	209980000
sp|Q13838	DDX39B	sp|Q13838	1	21	21	10	60	48.991	9949800000	1.1378E+10	1.1567E+10	10792000000	10238000000	1.173E+10
sp|Q13867	BLMH	sp|Q13867	1	12	12	12	36	52.562	1037300000	992440000	1161500000	1117500000	862550000	919700000
sp|Q13868	EXOSC2	sp|Q13868	1	7	7	7	32	32.789	733210000	661120000	804050000	762740000	810360000	764860000
sp|Q13885	TUBB2A	sp|Q13885	1	22	6	1	67	49.906	951150000	2517100000	658930000	1151900000	1281000000	584460000
sp|Q6P1K	GTF2H2C	sp|Q6P1K	2	3	3	3	8.4	44.452	85115000	70936000	85288000	78610000	90311000	69876000
sp|Q13895	BYSL	sp|Q13895	1	11	11	11	31	49.601	425590000	446950000	442580000	425080000	353710000	329100000
sp|Q13907	IDI1	sp|Q13907	2	5	5	5	33	26.319	503270000	465660000	414230000	279070000	445230000	367190000
sp|Q13951	CBFB	sp|Q13951	1	3	3	3	18	21.508	255540000	226190000	236290000	284500000	360730000	312990000
sp|Q13952	NFYC	sp|Q13952	1	1	1	1	2.6	50.302	0	0	0	0	0	38834000
sp|Q14008	CKAP5	sp|Q14008	1	28	28	28	20	225.49	1751200000	1599500000	1405100000	1628500000	1665200000	1519200000
sp|Q14011	CIRBP	sp|Q14011	1	1	1	1	6.4	18.648	0	0	0	0	0	44629000

sp|Q1401	COTL1	sp|Q1401	1	9	9	9	63	15.945	3691800000	4183600000	3333100000	3476000000	3707400000	3284400000
sp|Q1406	COX17	sp|Q1406	1	3	3	3	57	6.9151	356200000	272170000	296690000	262890000	0	0
sp|Q1410	HNRNPD	sp|Q1410	1	12	12	11	30	38.434	8652400000	7743400000	7170300000	8998100000	7693400000	8154500000
sp|Q1410	SCARB2	sp|Q1410	1	6	6	6	18	54.29	439590000	701970000	510420000	599310000	495030000	500660000
sp|Q1411	LRP8	sp|Q1411	1	1	1	1	1.8	105.63	0	0	0	0	0	32750000
sp|Q1411	IL18	sp|Q1411	1	5	5	5	35	22.326	1098400000	1038700000	767270000	771280000	828920000	858290000
sp|Q1411	DAG1	sp|Q1411	1	3	3	3	6	97.44	0	0	0	0	234560000	0
sp|Q1412	DSG2	sp|Q1412	1	17	17	17	26	122.29	1394900000	1476600000	1326100000	1471300000	1481100000	1500000000
sp|Q1413	BOP1	sp|Q1413	1	19	19	19	38	83.629	1149600000	1495600000	972320000	1421300000	1670300000	1434900000
sp|Q1413	UBE4A	sp|Q1413	1	2	2	2	2.8	122.56	0	0	0	0	0	69452000
sp|Q1414	SEPT6	sp|Q1414	1	8	3	3	28	49.716	241410000	161850000	201860000	223930000	206470000	230410000
sp|Q1414	URB2	sp|Q1414	1	5	5	5	5	170.54	0	128320000	0	0	76794000	0
sp|Q1415	SAFB2	sp|Q1415	1	15	7	7	17	107.47	153710000	115500000	0	0	0	0
sp|Q1415	EIF3A	sp|Q1415	1	53	53	53	40	166.57	8405500000	9229000000	8422400000	7926800000	8313700000	9192300000
sp|Q1415	UBAP2L	sp|Q1415	1	20	20	20	34	114.53	2526100000	2520400000	2446900000	1924300000	2026800000	2060400000
sp|Q1416	SCRIB	sp|Q1416	2	10	10	10	12	174.88	411520000	315380000	404220000	402970000	472520000	251340000
sp|Q1416	GIT2	sp|Q1416	1	4	4	3	12	84.542	0	138520000	137890000	91468000	0	0
sp|Q1416	MLEC	sp|Q1416	1	7	7	7	30	32.233	391420000	402610000	401190000	381560000	444140000	550720000
sp|Q1416	TTLL12	sp|Q1416	1	25	25	25	52	74.403	3366000000	3773000000	3489300000	3390000000	3341200000	3534500000
sp|Q1418	POLA2	sp|Q1418	1	8	8	8	26	65.947	316170000	447570000	404690000	349960000	304460000	394450000
sp|Q1419	FHL2	sp|Q1419	1	11	11	11	56	32.193	1495200000	1277100000	1555300000	1317900000	1693900000	1352100000
sp|Q1419	DPYSL3	sp|Q1419	1	21	21	17	55	61.963	5628300000	4779400000	5421200000	4956500000	4546900000	5246200000
sp|Q1419	MRPL58	sp|Q1419	1	3	3	3	19	23.63	151320000	128850000	0	0	120760000	115260000
sp|Q1420	ZMYM3	sp|Q1420	1	2	2	2	4.3	152.38	0	0	0	0	0	7288500
sp|Q1420	DCTN1	sp|Q1420	1	28	28	28	31	141.69	1836600000	1488500000	1752300000	1402000000	1685600000	1550300000
sp|Q1420	DYNC1H1	sp|Q1420	1	##	164	##	42	532.4	2.4479E+10	2.483E+10	2.463E+10	25350000000	26183000000	2.5929E+10
sp|Q1423	EIF2B1	sp|Q1423	1	12	12	12	47	33.712	1040600000	1125800000	1060800000	1222600000	964210000	680970000
sp|Q1424	EIF4A2	sp|Q1424	1	15	3	3	43	46.402	145100000	64665000	240470000	0	134540000	161920000
sp|Q1424	ELOA	sp|Q1424	1	3	3	3	5.6	89.908	165030000	0	125860000	126510000	0	0
sp|Q1424	MAP7	sp|Q1424	1	7	7	7	12	84.051	0	391730000	398540000	0	397440000	354700000
sp|Q1424	CTTN	sp|Q1424	1	23	23	23	48	61.585	7109500000	5862300000	6915000000	4547400000	5302500000	5107900000
sp|Q1424	ENDOG	sp|Q1424	1	2	2	2	13	32.62	56501000	0	0	0	0	0
sp|Q1425	FLOT2	sp|Q1425	1	9	9	9	27	47.064	380470000	450350000	413120000	428000000	326060000	314100000
sp|Q1425	RCN2	sp|Q1425	1	9	9	9	47	36.876	682340000	569290000	638150000	726880000	1057100000	836640000
sp|Q1425	TRIM25	sp|Q1425	1	19	19	19	45	70.973	1645500000	1255600000	1651600000	1701300000	1445200000	1322800000
sp|Q1431	FLNC	sp|Q1431	1	57	44	44	33	291.02	3237000000	3167000000	3341000000	3891200000	3779600000	3842100000
sp|Q1431	FKBP8	sp|Q1431	1	6	6	6	19	44.561	556710000	594100000	461280000	540020000	615530000	0
sp|Q1432	FAM50A	sp|Q1432	2	8	8	8	31	40.241	1056800000	1159600000	1075400000	1020000000	1094900000	1090500000
sp|Q1433	FRG1	sp|Q1433	1	5	5	5	19	29.172	290670000	345980000	0	305150000	367520000	0

sp|Q14344	GNA13	sp|Q14344	1	3	3	3	10	44.049	131110000	130170000	134080000	124510000	144400000	116840000
sp|Q14353	GAMT	sp|Q14353	1	3	3	3	26	26.318	50151000	0	0	0	0	0
sp|Q14376	GALE	sp|Q14376	1	10	10	10	50	38.281	615790000	637270000	642870000	666890000	536820000	711930000
sp|Q14432	PDE3A	sp|Q14432	1	14	14	14	22	124.98	1427400000	1448400000	1419400000	1330000000	1272500000	1334400000
sp|Q14444	CAPRIN1	sp|Q14444	1	15	15	15	30	78.365	5255600000	5558300000	5461500000	4149600000	3986600000	4149700000
sp|Q14498	RBM39	sp|Q14498	1	12	12	12	29	59.379	1806600000	1983600000	2070800000	1795200000	1922400000	1777800000
sp|Q14527	HLTF	sp|Q14527	1	5	5	5	8.8	113.93	111010000	0	0	0	0	76279000
sp|Q14554	PDIA5	sp|Q14554	1	1	1	1	6.6	59.594	0	0	0	0	0	42712000
sp|Q14558	PRPSAP1	sp|Q14558	1	8	8	7	34	39.393	519290000	639230000	540900000	624350000	387240000	510370000
sp|Q14562	DHX8	sp|Q14562	1	8	7	7	11	139.31	179770000	240020000	157030000	174640000	141490000	153540000
sp|Q14566	MCM6	sp|Q14566	1	29	29	29	40	92.888	5619700000	6406400000	5263300000	5315300000	5522700000	4974300000
sp|Q14573	ITPR3	sp|Q14573	2	29	29	24	17	304.1	1299700000	1475200000	1486400000	1472700000	1533700000	1725600000
sp|Q14643	ITPR1	sp|Q14643	1	14	9	9	7.9	313.93	242050000	259900000	189250000	258370000	276240000	273540000
sp|Q14651	PLS1	sp|Q14651	1	6	3	3	14	70.253	60733000	76098000	0	70846000	43841000	73624000
sp|Q14653	IRF3	sp|Q14653	1	2	2	2	7.7	47.219	0	0	0	71337000	81429000	71642000
sp|Q14669	TRIP12	sp|Q14669	1	8	8	8	5.6	220.43	256150000	348250000	270160000	227060000	234170000	271310000
sp|Q14671	PUM1	sp|Q14671	2	14	14	14	16	126.47	465210000	422900000	449060000	403740000	433290000	431140000
sp|Q14676	MDC1	sp|Q14676	1	16	16	16	15	226.66	582100000	546670000	568850000	374700000	567260000	579020000
sp|Q14677	CLINT1	sp|Q14677	1	12	12	12	24	68.259	1698300000	1465500000	1192600000	1316800000	1576400000	1521100000
sp|Q14680	MELK	sp|Q14680	1	3	3	3	9.1	74.641	76909000	83080000	0	0	70941000	80704000
sp|Q14683	SMC1A	sp|Q14683	1	29	29	29	29	143.23	2063000000	2711300000	1995500000	1830000000	1999100000	2535400000
sp|Q14684	RRP1B	sp|Q14684	1	12	12	12	23	84.427	704450000	633620000	652490000	563740000	687470000	690980000
sp|Q14690	PDCD11	sp|Q14690	1	18	18	18	14	208.7	767060000	847360000	760930000	844110000	797800000	818610000
sp|Q14691	GINS1	sp|Q14691	1	2	2	2	15	22.988	0	91644000	0	0	0	0
sp|Q14692	BMS1	sp|Q14692	1	13	13	13	14	145.81	932460000	784510000	885800000	637950000	829580000	599790000
sp|Q14694	USP10	sp|Q14694	1	10	10	10	23	87.133	473350000	402720000	357160000	284590000	417850000	350080000
sp|Q14696	MESD	sp|Q14696	1	8	8	8	43	26.076	945910000	720390000	791060000	1035000000	1142000000	1467000000
sp|Q14697	GANAB	sp|Q14697	1	40	40	40	47	106.87	1.7962E+10	1.8981E+10	1.7638E+10	20766000000	18690000000	2.1429E+10
sp|Q14728	MFSD10	sp|Q14728	1	1	1	1	2.9	48.339	0	0	0	0	0	54598000
sp|Q14738	PPP2R5D	sp|Q14738	2	10	10	7	23	69.991	616960000	752810000	496120000	531500000	632150000	470480000
sp|Q14739	LBR	sp|Q14739	1	7	7	7	12	70.702	710030000	711100000	660290000	474820000	767140000	767910000
sp|Q14764	MVP	sp|Q14764	1	28	28	28	40	99.326	2885900000	2764700000	2813700000	2579200000	2547900000	2619300000
sp|Q14789	GOLGB1	sp|Q14789	1	16	16	16	7.6	376.01	625460000	789570000	845530000	664660000	618870000	687830000
sp|Q14790	CASP8	sp|Q14790	1	5	5	5	14	55.391	693930000	1138300000	1249000000	1026900000	1045200000	1295900000
sp|Q14807	KIF22	sp|Q14807	1	6	6	6	13	73.261	92878000	63083000	108560000	117780000	75278000	93178000
sp|Q14839	CHD4	sp|Q14839	2	38	38	32	30	218	2957800000	2863300000	3102000000	2897400000	2988300000	2905400000
sp|Q14847	LASP1	sp|Q14847	1	14	14	14	59	29.717	5426900000	5894800000	6219400000	4646800000	6026500000	4007900000
sp|Q14914	PTGR1	sp|Q14914	1	13	13	13	45	35.869	2728400000	2242300000	1981400000	1872100000	2332100000	2382100000
sp|Q14919	DRAP1	sp|Q14919	1	3	3	3	22	22.35	210950000	186490000	284430000	179360000	193790000	228200000

sp|Q1496	ZNF638	sp|Q1496	1	6	6	6	3	220.62	1551300000	1398200000	1584000000	1770200000	1975800000	1419500000
sp|Q1497	KPNB1	sp|Q1497	1	30	30	30	41	97.169	1.2228E+10	1.3841E+10	1.2216E+10	13000000000	11867000000	1.2574E+10
sp|Q1497	NOLC1	sp|Q1497	1	9	9	9	15	73.602	2078700000	1908400000	2315100000	1781900000	1985500000	2071900000
sp|Q1498	NUMA1	sp|Q1498	1	71	71	71	47	238.26	8597700000	7875900000	8324500000	8284100000	7682100000	7274000000
sp|Q1499	PSME4	sp|Q1499	1	7	7	7	5.3	211.33	0	126120000	121090000	127900000	115690000	101870000
sp|Q14C8	GAPVD1	sp|Q14C8	1	22	22	22	23	164.98	1435300000	1553300000	1630000000	1727000000	1688700000	1637200000
sp|Q14CX	NAA25	sp|Q14CX	1	11	11	11	14	112.29	317260000	342650000	311270000	297980000	307380000	305360000
sp|Q1500	NCAPH	sp|Q1500	1	15	15	15	31	82.562	889110000	623750000	1167500000	885080000	912400000	787740000
sp|Q1500	PCLAF	sp|Q1500	1	1	1	1	16	11.986	0	0	24310000	0	0	0
sp|Q1500	SPCS2	sp|Q1500	1	7	7	7	39	25.003	1014900000	1108100000	947170000	1189500000	1342000000	1786800000
sp|Q1500	EMC2	sp|Q1500	1	5	5	5	23	34.833	221040000	659010000	367720000	579310000	633180000	548100000
sp|Q1500	WTAP	sp|Q1500	1	4	4	4	16	44.243	198400000	216760000	220380000	177020000	225690000	209410000
sp|Q1500	PSMD6	sp|Q1500	1	17	17	17	41	45.531	2587900000	2464600000	3307100000	2633900000	2476800000	2953400000
sp|Q1501	MORF4L2	sp|Q1501	1	6	5	5	26	32.307	198270000	224160000	233510000	146530000	96786000	107780000
sp|Q1501	ABRAXAS	sp|Q1501	1	6	6	6	20	46.9	0	141110000	185660000	0	0	0
sp|Q1501	SEPT2	sp|Q1501	1	13	13	13	52	41.487	4062700000	4557900000	3935400000	3789400000	4646400000	3630600000
sp|Q1502	SART3	sp|Q1502	1	7	7	7	10	109.93	437440000	390120000	489140000	381410000	439280000	576220000
sp|Q1502	NCAPD2	sp|Q1502	1	24	24	24	27	157.18	1969700000	1825600000	1860800000	1681600000	1740000000	1778700000
sp|Q1502	SUZ12	sp|Q1502	1	2	2	2	6.4	83.054	77827000	0	67991000	0	0	53537000
sp|Q1502	EXOSC7	sp|Q1502	1	9	9	9	43	31.821	1097100000	990960000	1167100000	1160300000	1286700000	1189600000
sp|Q1502	EFTUD2	sp|Q1502	1	40	39	39	59	109.43	7607500000	7054400000	7263200000	6844200000	6371200000	6566600000
sp|Q1503	LARS2	sp|Q1503	1	11	11	11	20	101.97	272940000	403440000	355140000	412250000	467060000	353710000
sp|Q1503	SNX17	sp|Q1503	1	4	4	4	15	52.901	0	0	0	0	0	69607000
sp|Q1504	ARL6IP1	sp|Q1504	1	2	2	2	8.9	23.362	0	0	0	0	0	720140000
sp|Q1504	RAB3GAF	sp|Q1504	1	10	10	10	14	110.52	431630000	178790000	484340000	289130000	586090000	358430000
sp|Q1504	SLC39A1	sp|Q1504	1	3	3	3	9.1	54.212	331390000	478600000	351780000	331620000	409330000	452610000
sp|Q1504	KARS	sp|Q1504	1	20	20	20	43	68.047	4857600000	5420200000	4593500000	4894800000	4151200000	5138000000
sp|Q1505	RRS1	sp|Q1505	1	9	9	9	33	41.193	474370000	919070000	627750000	720880000	665290000	842520000
sp|Q1505	POLD3	sp|Q1505	1	3	3	3	9.7	51.4	182960000	203520000	233230000	157620000	202350000	226610000
sp|Q1505	EIF4H	sp|Q1505	1	10	10	10	50	27.385	4759200000	4507000000	4827800000	3430800000	4959700000	3315500000
sp|Q1505	ACAP2	sp|Q1505	1	1	1	1	2.4	88.028	0	0	0	0	23525000	0
sp|Q1505	KIF14	sp|Q1505	1	2	2	2	2.7	186.49	27820000	0	30848000	20725000	54849000	24665000
sp|Q1506	WDR43	sp|Q1506	1	11	11	11	26	74.89	1104800000	975270000	836180000	950180000	1202700000	791000000
sp|Q1506	ACOX1	sp|Q1506	1	11	11	11	32	74.423	629470000	736180000	688320000	752930000	887770000	728750000
sp|Q1507	OXA1L	sp|Q1507	1	3	3	3	7.4	48.547	127690000	332770000	266830000	136510000	198250000	223620000
sp|Q1507	EEA1	sp|Q1507	1	9	9	9	9.1	162.46	208230000	225210000	188740000	183530000	227740000	0
sp|Q1508	PDIA6	sp|Q1508	1	14	14	14	43	48.121	5647900000	6249200000	7077800000	7426300000	7706000000	6647800000
sp|Q1510	PAFAH1B	sp|Q1510	1	3	3	3	20	25.734	347630000	161160000	317060000	326500000	220630000	338230000
sp|Q1512	PEA15	sp|Q1512	1	5	5	5	62	15.04	240820000	350760000	258680000	239480000	205480000	231210000

sp|Q1512	EBP	sp|Q1512	1	3	3	3	17	26.352	2858100000	2846900000	2521100000	2487200000	2697700000	2224700000
sp|Q1512	PMVK	sp|Q1512	1	9	9	9	46	21.995	726690000	854130000	725180000	619560000	680010000	848450000
sp|Q1514	PLEC	sp|Q1514	1	##	262	##	58	531.78	1.1136E+11	1.254E+11	1.1758E+11	1.2391E+11	1.271E+11	1.2998E+11
sp|Q1515	PCM1	sp|Q1515	1	2	2	2	1.4	228.54	0	0	62506000	0	0	73868000
sp|Q1515	NOMO1	sp|Q1515	1	19	2	2	24	134.32	0	0	0	0	0	34001000
sp|Q1516	PON2	sp|Q1516	1	4	4	4	20	39.38	441340000	587470000	498690000	448100000	601040000	562700000
sp|Q1518	PPA1	sp|Q1518	1	18	18	18	70	32.66	6199700000	6557800000	6652300000	5676100000	5850100000	5454700000
sp|Q1518	PTGES3	sp|Q1518	1	7	7	7	51	18.697	7149400000	6792400000	7871600000	7023100000	7297100000	7272300000
sp|Q1520	STK38	sp|Q1520	2	4	4	4	10	54.19	124900000	110580000	107220000	102900000	97170000	113700000
sp|Q1523	NONO	sp|Q1523	1	25	24	23	60	54.231	1.8766E+10	1.8096E+10	1.7436E+10	17651000000	17992000000	2.0657E+10
sp|Q1525	PTPA	sp|Q1525	1	10	10	10	44	40.667	1008300000	969220000	690940000	898880000	819790000	465540000
sp|Q1526	PTPRK	sp|Q1526	1	1	1	1	1	162.1	0	0	0	0	30331000	0
sp|Q1526	PWP2	sp|Q1526	1	13	13	13	21	102.45	611490000	728700000	538160000	512700000	612830000	623250000
sp|Q1527	RABEP1	sp|Q1527	1	4	4	4	6.7	99.289	97771000	154150000	0	146850000	214250000	0
sp|Q1528	RAB35	sp|Q1528	1	4	4	4	24	23.025	617400000	672520000	511130000	1101900000	484750000	440800000
sp|Q1528	RNPS1	sp|Q1528	1	6	6	6	26	34.208	887520000	826370000	768080000	860650000	839720000	872640000
sp|Q1529	RBBP5	sp|Q1529	1	3	3	3	10	59.152	184240000	162450000	127150000	117280000	0	116430000
sp|Q1529	RCN1	sp|Q1529	1	15	15	15	65	38.89	3709400000	4903200000	4117600000	4988900000	5148800000	4758100000
sp|Q1536	TMED2	sp|Q1536	1	4	4	4	18	22.761	1036700000	628540000	1099000000	1227200000	887700000	1348600000
sp|Q1536	PCBP1	sp|Q1536	2	13	13	9	60	37.497	1.4357E+10	1.6546E+10	1.4434E+10	14598000000	16036000000	1.7989E+10
sp|Q1536	PCBP2	sp|Q1536	2	12	8	8	47	38.58	8167800000	6987800000	7629400000	7808600000	8200600000	8306100000
sp|Q1536	ELOC	sp|Q1536	1	7	7	7	66	12.473	3005700000	2968600000	1940300000	2379500000	3360500000	2818000000
sp|Q1537	ELOB	sp|Q1537	1	7	7	7	83	13.133	2346300000	1745800000	2677100000	2105300000	3231200000	1852200000
sp|Q1538	RHEB	sp|Q1538	1	7	7	7	36	20.497	545620000	589770000	591120000	577140000	635850000	530780000
sp|Q1538	UBE3C	sp|Q1538	1	6	6	6	8.5	123.92	100850000	85109000	82554000	80026000	79848000	82333000
sp|Q1538	TOMM20	sp|Q1538	1	1	1	1	9	16.298	0	0	0	0	0	282350000
sp|Q1539	DHCR24	sp|Q1539	1	6	6	6	13	60.101	851110000	1006000000	908970000	961230000	791050000	700660000
sp|Q1539	SF3B3	sp|Q1539	1	31	31	31	36	135.58	3993900000	3468500000	3906100000	4096500000	3816300000	3638500000
sp|Q1539	PUM3	sp|Q1539	1	14	14	14	30	73.584	659890000	701660000	617070000	596990000	370380000	608900000
sp|Q1540	RSU1	sp|Q1540	1	8	8	8	37	31.54	581380000	722660000	601100000	637870000	659890000	636740000
sp|Q1541	CNN3	sp|Q1541	1	11	10	10	48	36.413	947440000	727660000	458220000	508740000	755100000	317610000
sp|Q1541	RPS6KA1	sp|Q1541	1	12	8	7	22	82.722	338730000	233670000	471200000	333790000	257590000	379860000
sp|Q1542	SAFB	sp|Q1542	1	19	19	11	22	102.64	2397600000	2310000000	2699200000	2489600000	2599600000	2326600000
sp|Q1542	SF3B4	sp|Q1542	1	1	1	1	3.3	44.385	0	0	0	264590000	0	0
sp|Q1542	SF3A2	sp|Q1542	1	7	7	7	23	49.255	1551100000	1803900000	1231800000	1306100000	1510900000	2052300000
sp|Q1543	PPP1R7	sp|Q1543	1	8	8	8	30	41.564	242490000	334180000	249640000	446900000	255900000	265840000
sp|Q1543	SEC23A	sp|Q1543	1	18	18	16	33	86.16	2079600000	2397900000	2095900000	2300700000	2153300000	2175700000
sp|Q1543	SEC23B	sp|Q1543	1	13	11	11	24	86.478	556740000	536900000	589780000	463060000	469550000	477240000
sp|Q1545	SF3A1	sp|Q1545	1	24	24	24	36	88.885	3932400000	3432500000	3775500000	3875300000	4023100000	3879400000

sp|Q1547	SKIV2L	sp|Q1547	1	7	7	7	11	137.75	152720000	257300000	309940000	384400000	315700000	195880000
sp|Q1552	SURF1	sp|Q1552	1	4	4	4	21	33.331	208800000	275550000	396540000	148460000	83683000	151750000
sp|Q1553	SS18	sp|Q1553	1	1	1	1	1.9	45.929	0	0	17549000	0	0	0
sp|Q1555	MAPRE2	sp|Q1555	1	4	4	4	18	37.031	302630000	272960000	275060000	255820000	358910000	278170000
sp|Q1558	TGFBI	sp|Q1558	1	7	7	7	16	74.68	322480000	366130000	455410000	278580000	289670000	243250000
sp|Q1559	SLC9A3R	sp|Q1559	1	6	6	6	22	37.413	236330000	185590000	197580000	183230000	248370000	276280000
sp|Q1563	TSN	sp|Q1563	1	8	8	8	42	26.183	1284900000	1343400000	990610000	1118800000	1135200000	1091000000
sp|Q1563	SF1	sp|Q1563	1	13	13	13	31	68.329	2074000000	2095300000	2262500000	2077100000	2299900000	2144600000
sp|Q1564	TRIP10	sp|Q1564	1	8	8	8	21	68.351	374550000	378210000	335350000	453100000	411710000	281540000
sp|Q1564	TRIP11	sp|Q1564	1	3	3	3	1.7	227.58	0	0	0	0	0	161980000
sp|Q1564	TRIP13	sp|Q1564	1	16	16	16	40	48.55	4718600000	3503400000	4522300000	4639800000	4777000000	4780300000
sp|Q1564	OASL	sp|Q1564	1	4	4	4	12	59.225	0	84126000	0	79637000	0	0
sp|Q1565	TRIP4	sp|Q1565	1	6	6	6	15	66.145	91270000	0	106870000	0	103860000	97237000
sp|Q1565	TRIP6	sp|Q1565	1	15	15	15	59	50.287	1835800000	2088500000	1797200000	1798400000	1836200000	1943300000
sp|Q1569	MAPRE1	sp|Q1569	2	14	14	14	56	29.999	3952700000	3283100000	3940100000	3590900000	3548900000	4272000000
sp|Q1571	TSC22D1	sp|Q1571	1	1	1	1	1.1	109.68	0	0	0	0	0	17695000
sp|Q1571	ELAVL1	sp|Q1571	1	9	9	9	33	36.091	2868500000	2560000000	2705800000	2961000000	2998500000	2723200000
sp|Q1573	NSDHL	sp|Q1573	1	8	8	8	31	41.9	890090000	934900000	838140000	851520000	800900000	870310000
sp|Q1574	MYLK	sp|Q1574	1	1	1	1	0.4	210.71	0	0	0	0	0	43787000
sp|Q1575	TAB1	sp|Q1575	1	1	1	1	2.2	54.643	0	0	0	0	0	32282000
sp|Q1575	SLC1A5	sp|Q1575	1	11	11	11	31	56.598	1942600000	2197900000	2425200000	2534100000	2925600000	2607300000
sp|Q1577	MLF2	sp|Q1577	1	3	3	3	16	28.147	0	0	0	0	0	115370000
sp|Q1578	TOMM34	sp|Q1578	1	15	15	15	58	34.559	1464800000	1499600000	1575200000	1633000000	1769900000	1589900000
sp|Q1579	SMAD2	sp|Q1579	1	4	4	2	11	52.306	147830000	145790000	160890000	152130000	183420000	169090000
sp|Q1580	MSMO1	sp|Q1580	1	1	1	1	4.8	35.215	0	0	0	19500000	0	0
sp|Q1581	TBCE	sp|Q1581	1	6	6	6	17	59.345	97647000	227480000	170450000	126430000	0	0
sp|Q1581	TBCC	sp|Q1581	1	4	4	4	27	39.248	0	92821000	0	91091000	83380000	77929000
sp|Q1583	STXBP2	sp|Q1583	1	3	3	3	8.1	66.452	0	0	0	130780000	0	0
sp|Q1583	VAMP3	sp|Q1583	3	4	4	4	52	11.309	1441800000	924540000	900520000	1065100000	1448700000	1496100000
sp|Q1584	NEDD8	sp|Q1584	1	2	2	2	17	9.0714	0	711680000	740240000	0	417150000	0
sp|Q1590	ATP6AP1	sp|Q1590	1	6	6	6	16	52.025	241820000	145500000	188020000	130240000	188990000	197590000
sp|Q1590	RAB11B	sp|Q1590	2	13	13	13	61	24.488	3023000000	2997300000	3214000000	4008600000	3794500000	2842100000
sp|Q1594	ZYX	sp|Q1594	1	19	19	19	56	61.277	5669900000	5296300000	5034900000	5038500000	4560100000	4203500000
sp|Q1613	ETFDH	sp|Q1613	1	2	2	2	3.9	68.495	0	0	0	0	0	72583000
sp|Q1618	SEPT7	sp|Q1618	2	13	13	13	33	50.679	4454400000	4436000000	4212300000	3481200000	3449500000	3432000000
sp|Q1618	ADRM1	sp|Q1618	1	6	6	6	20	42.153	770390000	662720000	650940000	614900000	695820000	564160000
sp|Q1620	CCDC6	sp|Q1620	1	12	12	12	34	53.29	692720000	670870000	720270000	579510000	734720000	663700000
sp|Q1622	UAP1	sp|Q1622	1	9	9	8	23	58.768	693090000	611710000	866760000	609740000	729710000	736280000
sp|Q1627	IGFBP7	sp|Q1627	1	3	3	3	18	29.13	201220000	261790000	215000000	162280000	212200000	215780000

sp|Q16401	PSMD5	sp|Q16401	1	20	20	20	56	56.195	3184600000	3663500000	4364700000	3606200000	3784300000	3486100000
sp|Q16513	PKN2	sp|Q16513	1	13	13	13	23	112.03	636110000	592300000	577400000	597820000	642650000	525650000
sp|Q16527	CSRP2	sp|Q16527	1	3	3	3	18	20.954	156360000	233590000	160390000	135340000	0	151500000
sp|Q16531	DDB1	sp|Q16531	1	36	36	36	38	126.97	4485000000	4132400000	4129000000	3441800000	3053300000	3857800000
sp|Q16537	PPP2R5E	sp|Q16537	1	4	3	3	11	54.699	307140000	191510000	249040000	161800000	187550000	168850000
sp|Q16539	MAPK14	sp|Q16539	1	6	6	6	23	41.293	197390000	201480000	207360000	182860000	216270000	230620000
sp|Q16543	CDC37	sp|Q16543	1	11	11	11	33	44.468	3733600000	3352000000	3185500000	3340900000	3629500000	3239700000
sp|Q16555	DPYSL2	sp|Q16555	1	19	16	15	45	62.293	3751000000	2987700000	3852200000	3125300000	3168600000	2811100000
sp|Q16563	SYPL1	sp|Q16563	1	3	3	3	20	28.565	441240000	556880000	511120000	424250000	548320000	482840000
sp|Q16576	RBBP7	sp|Q16576	1	14	14	10	45	47.82	5557800000	6506900000	5549600000	4711000000	5449600000	5219000000
sp|Q16595	FXN	sp|Q16595	1	2	2	2	13	23.135	0	0	0	0	0	91802000
sp|Q16611	BAK1	sp|Q16611	1	1	1	1	16	23.408	0	0	0	0	0	36470000
sp|Q16626	MEA1	sp|Q16626	1	2	2	2	16	19.904	0	0	0	0	0	138130000
sp|Q16629	SRSF7	sp|Q16629	1	6	5	5	25	27.366	2310300000	1363300000	2362300000	2805300000	3009200000	2552100000
sp|Q16630	CPSF6	sp|Q16630	1	9	9	9	23	59.209	1361200000	1510500000	1386500000	1400700000	1249800000	1654700000
sp|Q16637	SMN1	sp|Q16637	1	5	5	5	31	31.848	247550000	364430000	363430000	238870000	314960000	321710000
sp|Q16643	DBN1	sp|Q16643	1	16	16	16	38	71.428	3905900000	3444600000	3380000000	2786100000	3471700000	3168700000
sp|Q16644	MAPKAPK	sp|Q16644	2	2	2	2	8.1	42.987	0	108260000	0	0	107860000	125930000
sp|Q16658	FSCN1	sp|Q16658	1	27	27	27	62	54.529	2.3897E+10	2.6226E+10	2.3974E+10	24355000000	21623000000	2.3893E+10
sp|Q16666	IFI16	sp|Q16666	2	15	15	15	26	88.255	1251300000	1229400000	1114500000	1696100000	1109300000	1418600000
sp|Q16698	DECR1	sp|Q16698	1	6	6	6	23	36.067	671580000	470270000	576350000	672810000	634800000	589910000
sp|Q16706	MAN2A1	sp|Q16706	1	3	3	3	3.1	131.14	102670000	0	0	0	0	0
sp|Q16718	NDUFA5	sp|Q16718	1	5	5	5	66	13.459	431740000	538920000	416940000	425400000	536240000	475570000
sp|Q16719	KYNU	sp|Q16719	1	20	20	20	50	52.351	5689900000	6661200000	6348000000	5869000000	5506400000	6121500000
sp|Q16740	CLPP	sp|Q16740	1	5	5	5	27	30.18	379030000	298560000	351880000	317490000	498210000	491280000
sp|Q16762	TST	sp|Q16762	1	3	3	3	15	33.429	0	0	113690000	0	0	0
sp|Q16763	UBE2S	sp|Q16763	1	7	7	7	45	23.845	354490000	302530000	217080000	211240000	230240000	404150000
sp|Q16774	GUK1	sp|Q16774	1	5	5	5	33	21.725	0	294580000	172500000	0	427820000	161480000
sp|Q16775	HAGH	sp|Q16775	1	3	3	3	9.4	33.805	0	0	0	77083000	0	0
sp|Q16795	NDUFA9	sp|Q16795	1	7	7	7	25	42.509	498360000	655350000	619430000	359160000	300670000	299820000
sp|Q16798	ME3	sp|Q16798	1	2	2	2	6	67.068	0	25432000	0	33162000	23515000	21258000
sp|Q16822	PCK2	sp|Q16822	2	9	9	9	23	70.698	226240000	186300000	261830000	340140000	352210000	342520000
sp|Q16831	UPP1	sp|Q16831	1	1	1	1	3.2	33.934	0	0	0	0	0	27968000
sp|Q16836	HADH	sp|Q16836	1	10	10	10	40	34.293	1138100000	1962300000	1065800000	1243400000	1237500000	1545500000
sp|Q16850	CYP51A1	sp|Q16850	1	8	8	8	19	56.805	402590000	340070000	474110000	311690000	326160000	294150000
sp|Q16851	UGP2	sp|Q16851	1	12	12	12	30	56.94	1461500000	1508000000	1684400000	1601100000	1817700000	1605400000
sp|Q16880	UGT8	sp|Q16880	1	2	2	2	4.6	61.437	72588000	57474000	84832000	58270000	0	0
sp|Q16881	TXNRD1	sp|Q16881	1	27	27	26	50	70.905	1.3338E+10	1.3386E+10	1.3355E+10	15066000000	13286000000	1.2877E+10
sp|Q16890	TPD52L1	sp|Q16890	1	3	3	3	27	22.449	242960000	140850000	150460000	0	0	0

sp|Q1689	IMMT	sp|Q1689	1	26	26	26	49	83.677	5125600000	4736100000	4028000000	5330600000	6021100000	5848600000
sp|Q17RY	LY6K	sp|Q17RY	1	2	2	2	13	18.673	124730000	159610000	0	0	0	0
sp|Q1ED3	KNOP1	sp|Q1ED3	1	2	2	2	5.5	51.588	0	0	0	0	0	115230000
sp|Q1KMD	HNRNPUL	sp|Q1KMD	1	23	23	23	36	85.104	4177200000	4308200000	4072200000	4583500000	4275600000	3934500000
sp|Q27J8	INF2	sp|Q27J8	1	21	21	21	30	135.62	1095200000	986090000	974490000	877940000	1035700000	1013700000
sp|Q2996	HLA-C	sp|Q2996	4	12	9	0	46	40.968	1705500000	1473100000	1526400000	1573600000	1692100000	2128700000
sp|Q29RF	PDS5A	sp|Q29RF	1	22	22	20	23	150.83	951380000	1298300000	1165400000	1058200000	1130100000	883760000
sp|Q2KHF	QSER1	sp|Q2KHF	1	2	2	2	2.3	189.97	0	0	0	14429000	13751000	14539000
sp|Q2M2I	AAK1	sp|Q2M2I	1	3	3	3	7.5	103.88	0	108120000	180230000	0	0	242370000
sp|Q2M38	WASHC4	sp|Q2M38	1	6	6	6	7.1	136.4	83266000	63548000	85578000	102850000	87881000	94390000
sp|Q2NL8	TSR1	sp|Q2NL8	1	15	15	15	27	91.809	896600000	904580000	838290000	845800000	663510000	838030000
sp|Q2PZI	DPY19L1	sp|Q2PZI	2	3	3	3	6.7	77.318	0	0	0	102890000	102430000	0
sp|Q2TAA	IAH1	sp|Q2TAA	1	1	1	1	6.5	27.598	0	0	0	0	0	10880000
sp|Q2TAL	QRICH1	sp|Q2TAL	1	9	9	9	18	86.435	239650000	398450000	267570000	557370000	405340000	377840000
sp|Q2TAY	SMU1	sp|Q2TAY	1	15	15	15	44	57.543	1723200000	1997900000	1882400000	1350100000	1157900000	1446600000
sp|Q2VPK	CTU2	sp|Q2VPK	1	2	2	2	6.4	56.107	0	0	0	0	38485000	0
sp|Q32MZ	LRRFIP1	sp|Q32MZ	1	12	12	11	22	89.252	1823700000	1452900000	1393400000	1511000000	1568400000	1751400000
sp|Q32P2	P3H1	sp|Q32P2	1	17	17	16	33	83.393	1247800000	1226100000	1012100000	1090400000	1103700000	1159600000
sp|Q32P4	TRMT5	sp|Q32P4	1	1	1	1	2.9	58.246	0	0	0	0	0	55699000
sp|Q3B72	TWISTNB	sp|Q3B72	1	1	1	1	4.7	37.432	0	0	0	0	0	137960000
sp|Q3KQU	MAP7D1	sp|Q3KQU	1	8	8	8	16	92.819	478070000	404350000	442650000	431890000	499580000	380220000
sp|Q3LXA	TKFC	sp|Q3LXA	1	5	5	5	17	58.946	215100000	180520000	154170000	200930000	188490000	157560000
sp|Q3MHD	LSM12	sp|Q3MHD	1	2	2	2	12	21.701	269480000	354650000	303110000	339770000	401600000	345550000
sp|Q3SXM	HSDL1	sp|Q3SXM	1	1	1	1	3.3	37.001	0	0	0	0	0	33231000
sp|Q3V6T	CCDC88A	sp|Q3V6T	1	3	3	3	1.7	216.04	126830000	0	0	0	0	144490000
sp|Q3YEC	RABL6	sp|Q3YEC	1	4	4	4	7.3	79.548	0	0	0	0	209790000	0
sp|Q3ZAQ	VMA21	sp|Q3ZAQ	1	1	1	1	12	11.354	0	125360000	0	0	0	0
sp|Q3ZCM	TUBB8	sp|Q3ZCM	1	10	2	2	25	49.775	1.3874E+10	1.5187E+10	9164300000	13379000000	13805000000	1.0997E+10
sp|Q3ZCQ	TIMM50	sp|Q3ZCQ	1	7	7	7	27	39.646	628120000	778510000	640540000	623080000	771110000	595320000
sp|Q49A2	GLYR1	sp|Q49A2	1	6	6	6	16	60.556	181860000	199780000	248390000	170570000	259510000	221850000
sp|Q49AR	C5orf22	sp|Q49AR	1	2	2	2	12	49.967	0	0	25183000	0	0	0
sp|Q4G0F	VPS26B	sp|Q4G0F	1	1	1	1	3.3	39.154	0	0	0	0	20308000	0
sp|Q4G0J	LARP7	sp|Q4G0J	1	6	6	6	13	66.898	223690000	153340000	177320000	197280000	248800000	216570000
sp|Q4G0N	NADK2	sp|Q4G0N	1	7	7	7	24	49.432	702140000	568040000	708390000	631830000	618390000	889240000
sp|Q4J6C	PREPL	sp|Q4J6C	1	5	5	5	12	83.926	132340000	121470000	210910000	103340000	109160000	0
sp|Q4KMF	TBC1D10	sp|Q4KMF	1	2	2	2	5.7	87.198	110900000	209650000	0	0	0	0
sp|Q4KMQ	ANO6	sp|Q4KMQ	1	1	1	1	2.4	106.16	0	0	0	0	0	47713000
sp|Q4KWH	PLCH1	sp|Q4KWH	1	2	2	2	2.6	189.22	0	371370000	0	0	0	0
sp|Q4V32	GRIPAP1	sp|Q4V32	1	9	9	9	21	95.988	161630000	121120000	124800000	170530000	199120000	113480000

sp	Q4VC3	CCDC58	sp	Q4VC3	1	4	4	4	37	16.62	364620000	280410000	182730000	323630000	441810000	206030000
sp	Q52LJ(	FAM98B	sp	Q52LJ(	1	6	6	6	30	37.19	740640000	666530000	613080000	992250000	1118900000	710820000
sp	Q53EL	PDCD4	sp	Q53EL	1	11	11	11	33	51.735	1445700000	1604300000	1474800000	540070000	570580000	534590000
sp	Q53EP	FNDC3B	sp	Q53EP	1	5	5	5	6.6	132.89	294490000	252520000	326420000	237970000	211570000	270540000
sp	Q53F1!	NCBP3	sp	Q53F1!	1	1	1	1	2.1	70.592	0	0	49910000	0	0	0
sp	Q53FT	HIKESHI	sp	Q53FT	1	2	2	2	15	21.627	0	0	0	108770000	0	0
sp	Q9P0S	ORMDL1	sp	Q9P0S	3	2	2	2	17	17.371	270660000	302310000	291340000	398350000	330170000	376540000
sp	Q53G4	IFI44L	sp	Q53G4	1	2	2	2	6.2	51.322	0	0	0	0	492810000	0
sp	Q53GQ	HSD17B1	sp	Q53GQ	1	6	6	6	25	34.324	641000000	678100000	598870000	602720000	661830000	815970000
sp	Q53GS	GLE1	sp	Q53GS	1	3	3	3	7.4	79.835	0	0	0	0	0	47206000
sp	Q53GS	USP39	sp	Q53GS	1	9	9	9	23	65.38	838410000	655920000	830280000	718850000	797750000	693540000
sp	Q53H1	AGK	sp	Q53H1	1	7	7	7	26	47.137	294610000	404130000	279640000	358230000	359760000	182100000
sp	Q53H8	LACTB2	sp	Q53H8	1	2	2	2	6.9	32.805	0	0	0	0	0	38573000
sp	Q53H9	PYCR3	sp	Q53H9	1	6	6	6	33	28.663	0	0	0	0	0	194000000
sp	Q53HC	EIPR1	sp	Q53HC	1	3	3	3	11	43.603	0	0	0	0	136300000	0
sp	Q53HL	CDCA8	sp	Q53HL	1	2	2	2	9.6	31.323	62654000	79071000	74838000	92178000	88249000	90930000
sp	Q53QV	LBH	sp	Q53QV	1	1	1	1	16	12.217	0	0	0	0	0	17812000
sp	Q53S0	RAB6D	sp	Q53S0	2	2	1	1	12	28.242	0	0	0	0	31640000	0
sp	Q53T5!	HS1BP3	sp	Q53T5!	1	1	1	1	4.3	42.78	0	0	0	0	0	10673000
sp	Q562R	ACTBL2	sp	Q562R	1	6	1	1	18	42.003	0	0	0	0	0	187390000
sp	Q567V	MPV17L2	sp	Q567V	1	1	1	1	7.8	23.18	0	0	0	0	0	61045000
sp	Q56VL	OCIAD2	sp	Q56VL	1	4	4	4	23	16.953	320520000	428140000	322530000	293570000	342870000	399300000
sp	Q58FF	HSP90AB	sp	Q58FF	1	6	1	1	11	58.264	0	0	0	0	440770000	0
sp	Q58FF	HSP90AB	sp	Q58FF	1	14	1	1	27	44.348	1533900000	2268900000	1943400000	1709600000	1124800000	1667200000
sp	Q5BJD	TMEM41E	sp	Q5BJD	1	1	1	1	6.5	32.513	0	251590000	198250000	216790000	294040000	155000000
sp	Q5BJF	TMEM97	sp	Q5BJF	1	2	2	2	13	20.848	166640000	0	0	166620000	142150000	0
sp	Q5BJH	YIF1B	sp	Q5BJH	1	2	2	2	17	34.435	0	0	0	0	51790000	0
sp	Q5BKZ	ZNF326	sp	Q5BKZ	1	11	11	11	25	65.653	1064100000	989730000	989840000	916080000	1085300000	1157200000
sp	Q5EBL	PDZD11	sp	Q5EBL	1	1	1	1	19	16.131	0	0	0	30474000	0	0
sp	Q5F1R	DNAJC21	sp	Q5F1R	1	7	7	7	17	62.027	269560000	342830000	328550000	322370000	290510000	249210000
sp	Q5GLZ	HERC4	sp	Q5GLZ	1	4	4	4	6.1	118.56	116250000	114540000	150740000	144560000	116040000	113000000
sp	Q5H9R	PPP6R3	sp	Q5H9R	1	15	15	15	22	97.668	1908200000	1962200000	2241700000	1778700000	1884800000	2191800000
sp	Q5HYI8	RABL3	sp	Q5HYI8	1	1	1	1	8.9	26.422	0	54713000	0	0	66551000	0
sp	Q5JPE	NOMO2	sp	Q5JPE	2	19	19	2	24	139.44	1436500000	1413700000	1365200000	1556700000	1433700000	1508100000
sp	Q5JRA	MIA3	sp	Q5JRA	1	4	4	4	2.8	213.7	0	0	0	138050000	0	0
sp	Q5JRX	PITRM1	sp	Q5JRX	1	14	14	14	19	117.41	876060000	975600000	945420000	1074600000	1342200000	1099300000
sp	Q5JS54	PSMG4	sp	Q5JS54	1	1	1	1	20	13.775	124650000	0	0	0	144710000	106650000
sp	Q5JSH	WDR44	sp	Q5JSH	1	5	5	5	8.7	101.37	92411000	252420000	315890000	191810000	256640000	207120000
sp	Q5JSL3	DOCK11	sp	Q5JSL3	1	1	1	1	0.7	237.67	0	0	0	0	0	53570000

sp|Q5JSZ	PRRC2B	sp|Q5JSZ	1	2	1	1	1.2	242.96	0	0	0	0	0	15103000
sp|Q5JTH	RRP12	sp|Q5JTH	1	18	18	18	17	143.7	917650000	751810000	725660000	892120000	920370000	797100000
sp|Q5JTJ	COA6	sp|Q5JTJ	1	4	4	4	34	14.116	629840000	609050000	767240000	450030000	604050000	0
sp|Q5JTV	TOR1AIP	sp|Q5JTV	1	11	11	10	24	66.248	1323500000	1093500000	1327100000	1206200000	1377200000	1867200000
sp|Q5JTZ	AARS2	sp|Q5JTZ	1	2	2	2	3.8	107.34	102190000	37813000	0	0	0	129600000
sp|Q5JUR	TEX30	sp|Q5JUR	1	1	1	1	4.8	25.585	0	0	0	0	62148000	0
sp|Q5JVF	PCID2	sp|Q5JVF	1	5	5	5	16	46.029	342340000	361750000	376260000	373830000	256250000	428250000
sp|Q5K65	SAMD9	sp|Q5K65	1	10	10	10	9.8	184.28	439410000	374340000	394780000	339530000	517830000	325380000
sp|Q5M77	SPECC1	sp|Q5M77	1	8	8	8	11	118.58	221190000	371130000	177890000	265240000	241390000	216700000
sp|Q5MIZ	PPP4R3B	sp|Q5MIZ	1	5	2	2	6.7	97.457	293760000	290880000	417390000	286830000	233940000	267830000
sp|Q5QJE	DNTTIP2	sp|Q5QJE	1	9	9	9	16	84.468	428400000	477930000	303060000	428660000	480230000	408270000
sp|Q5R3I	TTC38	sp|Q5R3I	1	1	1	1	4.3	52.787	0	0	0	0	0	32002000
sp|Q5RI1	COX20	sp|Q5RI1	1	3	3	3	32	13.291	270580000	333190000	293390000	367540000	292110000	421330000
sp|Q5RKV	EXOSC6	sp|Q5RKV	1	6	6	6	27	28.235	535050000	426310000	384770000	400160000	451730000	465310000
sp|Q5SNT	TMEM201	sp|Q5SNT	1	3	3	3	5.1	72.235	0	0	0	114790000	0	0
sp|Q5SRE	NUP188	sp|Q5SRE	1	14	14	14	11	196.04	337150000	398250000	355730000	315700000	275040000	244310000
sp|Q5SSJ	HP1BP3	sp|Q5SSJ	1	12	12	12	23	61.206	1134300000	1259600000	1234500000	1237200000	1379000000	1343400000
sp|Q5SW	CEP170	sp|Q5SW	2	6	6	6	5.7	175.29	195700000	252570000	195190000	229620000	301590000	0
sp|Q5SW	ODR4	sp|Q5SW	1	3	3	3	11	51.103	116380000	0	0	0	127520000	0
sp|Q5SY1	NOL9	sp|Q5SY1	1	6	6	6	13	79.322	381480000	367270000	382840000	269320000	256660000	297570000
sp|Q5T0F	CC2D1B	sp|Q5T0F	1	3	3	3	7.1	94.223	0	0	0	77223000	0	0
sp|Q5T0N	FNBP1L	sp|Q5T0N	1	2	2	2	5.8	70.065	0	0	0	0	0	25814000
sp|Q5T1C	THEM4	sp|Q5T1C	1	1	1	1	5	27.129	0	0	0	0	15164000	0
sp|Q5T1M	FKBP15	sp|Q5T1M	1	6	6	6	7.8	133.63	143470000	106660000	93316000	170740000	125620000	137970000
sp|Q5T20	ZC3H13	sp|Q5T20	1	3	3	3	2.5	196.63	333420000	166380000	385680000	289050000	376470000	358840000
sp|Q5T28	SPOUT1	sp|Q5T28	1	4	4	4	17	42.008	0	303310000	0	245220000	0	0
sp|Q5T3I	GPATCH	sp|Q5T3I	1	4	4	4	16	50.381	82850000	67807000	0	0	84955000	81880000
sp|Q5T4S	UBR4	sp|Q5T4S	1	49	49	49	14	573.83	1980200000	1916000000	1821500000	1803100000	1851900000	1988700000
sp|Q5T5C	STXBP5	sp|Q5T5C	1	2	2	2	2.3	127.57	0	0	0	0	0	46456000
sp|Q5T5P	KIAA1217	sp|Q5T5P	1	3	3	3	1.7	214.11	0	54125000	47162000	0	0	0
sp|Q5T65	MRPL2	sp|Q5T65	1	3	3	3	18	33.3	334430000	319780000	0	331830000	342450000	0
sp|Q5T6F	UBAP2	sp|Q5T6F	1	12	12	12	22	117.11	1038400000	926080000	943030000	886840000	1054300000	986870000
sp|Q5T6V	C9orf64	sp|Q5T6V	1	5	5	5	12	39.028	175770000	224260000	150840000	197330000	153350000	107050000
sp|Q5T8D	ACBD5	sp|Q5T8D	2	2	2	2	3.6	60.091	386030000	380200000	255480000	408440000	0	0
sp|Q5T8P	RBM26	sp|Q5T8P	1	12	12	11	14	113.6	481730000	531220000	374760000	672690000	462290000	588300000
sp|Q5T9A	ATAD3B	sp|Q5T9A	1	20	7	6	36	72.572	742950000	769250000	472800000	551900000	812610000	723580000
sp|Q5T9L	WLS	sp|Q5T9L	1	2	2	2	5.9	62.253	128450000	0	0	0	104590000	0
sp|Q5TA5	CPTP	sp|Q5TA5	1	1	1	1	10	24.365	0	0	0	0	32844000	0
sp|Q5TAQ	DCAF8	sp|Q5TAQ	1	1	1	1	1.8	66.851	0	0	0	0	36496000	0

sp|Q5TC1	ATPAF1	sp|Q5TC1	1	2	2	2	9.5	36.436	0	0	0	95371000	0	0
sp|Q5TDH	DDI2	sp|Q5TDH	1	5	5	5	21	44.522	431340000	253060000	393570000	363850000	328440000	287930000
sp|Q5TFE	NT5DC1	sp|Q5TFE	1	9	9	9	30	51.844	409500000	640520000	501780000	607120000	539370000	493440000
sp|Q5TGZ	MINOS1	sp|Q5TGZ	1	2	2	2	19	8.8081	0	0	0	743220000	0	0
sp|Q5THJ	VPS13D	sp|Q5THJ	1	1	1	1	0.4	491.91	0	0	0	0	1876500000	0
sp|Q5THK	PRR14L	sp|Q5THK	1	1	1	1	0.8	237.3	0	0	0	0	0	281830000
sp|Q5TZA	CROCC	sp|Q5TZA	1	1	1	1	0.4	228.52	0	0	0	0	0	874450000
sp|Q5U3C	TMEM164	sp|Q5U3C	1	1	1	1	6.1	33.507	0	0	0	0	0	0
sp|Q5U5X	LYRM7	sp|Q5U5X	1	3	3	3	40	11.955	249650000	244070000	276530000	249050000	0	0
sp|Q5U64	C12orf60	sp|Q5U64	1	1	1	1	3.3	27.626	0	0	0	0	0	107730000
sp|Q5UCC	EMC10	sp|Q5UCC	1	1	1	1	6.5	27.347	0	0	0	0	86965000	0
sp|Q5UIP	RIF1	sp|Q5UIP	1	9	9	9	5.5	274.46	198350000	315410000	288230000	240590000	279300000	284690000
sp|Q5VSL	STRIP1	sp|Q5VSL	1	4	4	4	8.4	95.575	75392000	0	0	0	87095000	60190000
sp|Q5VT2	CDC42BP	sp|Q5VT2	1	3	3	2	1.6	197.3	0	271750000	0	0	354980000	0
sp|Q5VT5	RPRD2	sp|Q5VT5	1	13	13	13	16	156.02	269890000	318210000	297230000	295510000	274120000	261150000
sp|Q5VT6	MARC1	sp|Q5VT6	2	3	3	3	17	37.499	105290000	101940000	83823000	141080000	79215000	109450000
sp|Q5VT7	ANXA8L1	sp|Q5VT7	2	9	9	9	39	36.879	771970000	938520000	517990000	751250000	798250000	667240000
sp|Q5VTL	PRPF38B	sp|Q5VTL	1	2	2	2	3.8	64.467	0	0	0	0	0	87961000
sp|Q5VTR	RNF20	sp|Q5VTR	1	8	8	6	10	113.66	362550000	345720000	381280000	363450000	418290000	436230000
sp|Q5VV4	CDKAL1	sp|Q5VV4	1	1	1	1	2.1	65.111	0	0	0	0	0	24205000
sp|Q5VW3	BROX	sp|Q5VW3	1	6	6	6	25	46.476	570270000	497210000	426180000	447730000	554980000	549050000
sp|Q5VW3	FOCAD	sp|Q5VW3	1	4	4	4	3.6	200.07	398800000	343270000	0	273650000	249070000	310760000
sp|Q5VYK	ECPAS	sp|Q5VYK	1	27	27	27	22	204.29	1682700000	1473800000	1619100000	1408400000	1476900000	1541100000
sp|Q5VZF	MBNL2	sp|Q5VZF	1	3	1	1	7.5	40.517	0	0	0	0	0	16310000
sp|Q5VZK	CARMIL1	sp|Q5VZK	1	3	3	3	3	151.56	293740000	211820000	304970000	0	185270000	255110000
sp|Q5VZL	ZMYM4	sp|Q5VZL	1	2	2	2	1.9	172.79	0	0	0	0	0	81062000
sp|Q5XKF	MIC13	sp|Q5XKF	1	2	2	2	33	13.087	114490000	56559000	0	0	0	61970000
sp|Q5ZPR	CD276	sp|Q5ZPR	1	3	3	3	12	57.235	401960000	388880000	258720000	502040000	388930000	418550000
sp|Q63HN	RNF213	sp|Q63HN	1	11	11	11	2.9	591.4	187830000	217660000	178370000	173690000	150670000	153500000
sp|Q63ZY	KANK2	sp|Q63ZY	1	8	8	8	14	91.173	166740000	187190000	187070000	207360000	210570000	242630000
sp|Q641Q	WASHC2/	sp|Q641Q	2	10	10	1	14	147.18	249070000	295300000	328950000	224300000	256620000	238560000
sp|Q658Y	FAM91A1	sp|Q658Y	1	4	4	4	7.3	93.908	62449000	53287000	57212000	0	0	59403000
sp|Q66K1	TBC1D9B	sp|Q66K1	1	3	3	3	3.7	140.52	0	0	0	0	0	188280000
sp|Q66K7	MAP1S	sp|Q66K7	1	2	2	2	3	112.21	59764000	36354000	45651000	61336000	45279000	34645000
sp|Q66PJ	ARL6IP4	sp|Q66PJ	1	1	1	1	3.8	44.915	0	0	0	0	0	71006000
sp|Q68CP	ARID2	sp|Q68CP	1	3	3	3	2.1	197.39	0	0	0	0	0	114730000
sp|Q68CZ	TNS3	sp|Q68CZ	2	10	10	9	12	155.26	294500000	448070000	353740000	475190000	269690000	312480000
sp|Q68DH	LMBRD2	sp|Q68DH	1	1	1	1	3	81.171	0	0	0	0	0	19736000
sp|Q68E0	INTS3	sp|Q68E0	1	4	4	4	7.3	118.07	230870000	250940000	141560000	177990000	170980000	244060000

sp|Q68EM	ARHGAP1	sp|Q68EM	1	16	16	16	36	95.436	1386000000	986370000	1103100000	1222500000	1435000000	1093500000
sp|Q69YL	NCBP2-A	sp|Q69YL	1	1	1	1	14	10.89	0	0	0	0	30496000	0
sp|Q69YN	CWF19L1	sp|Q69YN	1	5	5	5	14	60.618	98930000	80037000	122710000	161560000	87364000	0
sp|Q69YN	VIRMA	sp|Q69YN	1	3	3	3	2.6	202.02	0	0	42965000	36344000	39501000	0
sp|Q6DD8	ZNF787	sp|Q6DD8	1	2	2	2	8.1	40.428	0	0	0	0	0	16918000
sp|Q6DD8	ATL3	sp|Q6DD8	1	14	14	14	44	60.541	2237200000	2539700000	1868100000	2565200000	2210600000	2425400000
sp|Q6DKI	RPL7L1	sp|Q6DKI	1	2	2	2	11	28.661	279130000	240000000	0	0	0	0
sp|Q6DKJ	NXN	sp|Q6DKJ	1	5	5	5	15	48.392	108060000	126400000	98958000	134780000	147440000	135980000
sp|Q6EEV	POLR2M	sp|Q6EEV	1	2	2	2	25	15.131	96313000	145010000	104240000	91462000	130710000	0
sp|Q6EMK	VASN	sp|Q6EMK	1	2	2	2	6.5	71.712	0	84031000	0	0	0	0
sp|Q6FI81	CIAPIN1	sp|Q6FI81	1	6	6	6	26	33.582	317090000	272480000	273710000	201580000	310980000	331960000
sp|Q6GMY	PTRHD1	sp|Q6GMY	1	4	4	4	42	15.805	285980000	267900000	299390000	258060000	275840000	226640000
sp|Q6I9Y2	THOC7	sp|Q6I9Y2	1	3	3	3	18	23.743	0	0	0	65495000	0	0
sp|Q6IA86	ELP2	sp|Q6IA86	1	8	8	8	10	92.499	537000000	577910000	480330000	628260000	538330000	544790000
sp|Q6IAA8	LAMTOR1	sp|Q6IAA8	1	6	6	6	57	17.745	388560000	320900000	421020000	385020000	486810000	401790000
sp|Q6IAN0	DHRS7B	sp|Q6IAN0	1	4	4	4	16	35.119	94114000	0	0	89326000	110590000	128030000
sp|Q6IBS0	TWF2	sp|Q6IBS0	1	14	14	11	50	39.548	2229400000	2201900000	2251200000	1505000000	2091100000	1585600000
sp|Q6IBW	NCAPH2	sp|Q6IBW	1	2	2	2	8.1	68.226	0	0	0	0	0	46677000
sp|Q6IN85	PPP4R3A	sp|Q6IN85	1	6	6	3	8.8	95.367	0	213960000	186230000	164220000	0	150540000
sp|Q6IQ49	SDE2	sp|Q6IQ49	1	1	1	1	3.1	49.741	0	0	0	0	0	71693000
sp|Q6KC7	NIPBL	sp|Q6KC7	1	10	10	10	5.2	316.05	284520000	325050000	0	473200000	214460000	275460000
sp|Q6L8Q	PDE12	sp|Q6L8Q	1	7	7	7	22	67.351	557820000	496690000	446620000	466190000	555990000	464260000
sp|Q6NUK	SLC25A24	sp|Q6NUK	1	15	15	15	38	53.354	1183100000	1165300000	1034800000	1151900000	1067100000	1294400000
sp|Q6NUM	RETSAT	sp|Q6NUM	1	3	3	3	6.7	66.819	0	0	63311000	0	0	0
sp|Q6NUQ	TMEM214	sp|Q6NUQ	1	3	3	3	5.5	77.15	0	0	0	0	0	124950000
sp|Q6NVY	HIBCH	sp|Q6NVY	1	6	6	6	17	43.482	433630000	291010000	348930000	525110000	482070000	234330000
sp|Q6NXE	ARMC6	sp|Q6NXE	1	5	5	5	11	54.141	260740000	240080000	229110000	273880000	193890000	212830000
sp|Q6NXR	TTI2	sp|Q6NXR	1	2	2	2	5.9	56.914	0	0	0	0	0	24268000
sp|Q6NYC	JMJD6	sp|Q6NYC	1	6	6	6	23	46.461	235420000	239940000	239870000	226470000	219020000	217370000
sp|Q6NYC	PPP1R18	sp|Q6NYC	1	8	8	8	25	67.942	328700000	311900000	492150000	548590000	416320000	531750000
sp|Q6P58	MZT2A	sp|Q6P58	2	1	1	1	14	16.22	0	0	0	0	0	46431000
sp|Q6NZI2	CAVIN1	sp|Q6NZI2	1	10	10	10	37	43.476	2615600000	2927300000	2979000000	3562000000	3161100000	2905100000
sp|Q6P0Q	MAST2	sp|Q6P0Q	1	2	1	1	1.7	196.43	0	0	0	0	0	90567000
sp|Q6P15	DHX57	sp|Q6P15	1	3	3	3	3.5	155.6	0	314050000	0	0	0	0
sp|Q6P16	MRPL54	sp|Q6P16	1	2	2	2	41	15.819	0	103780000	74647000	121020000	0	0
sp|Q6P1A	LPCAT3	sp|Q6P1A	1	4	4	4	12	56.034	0	0	149580000	0	0	188230000
sp|Q6P1J9	CDC73	sp|Q6P1J9	1	14	14	14	28	60.576	706380000	691250000	696710000	691660000	837050000	596410000
sp|Q6P1K	PMF1	sp|Q6P1K	1	1	1	1	5.9	23.339	0	0	0	0	0	22896000
sp|Q6P1L	MRPL14	sp|Q6P1L	1	3	3	3	23	15.947	128280000	154710000	128970000	162100000	187670000	168060000

sp|Q6P1M	SLC27A4	sp|Q6P1M	1	6	6	6	14	72.063	184870000	146020000	203800000	195900000	196210000	151930000
sp|Q6P1N	CC2D1A	sp|Q6P1N	1	6	6	6	9.4	104.06	0	79452000	140300000	93227000	142000000	154480000
sp|Q96IZ6	METTL2A	sp|Q96IZ6	2	1	1	1	5.8	43.537	0	0	0	0	0	48886000
sp|Q6P1X	TAF2	sp|Q6P1X	1	2	2	2	2	136.97	367850000	0	0	0	0	1044300000
sp|Q6P1X	C8orf82	sp|Q6P1X	1	1	1	1	8.3	23.889	0	0	0	0	0	71965000
sp|Q6P2E	EDC4	sp|Q6P2E	1	18	18	18	24	151.66	881700000	965630000	1000600000	900430000	1131100000	976230000
sp|Q96GK	FAHD2A	sp|Q96GK	2	3	3	3	17	34.596	0	0	0	0	85629000	0
sp|Q6P2Q	PRPF8	sp|Q6P2Q	1	63	63	63	35	273.6	8919700000	8805900000	9018600000	8500500000	8006700000	8391000000
sp|Q6P3W	SCYL2	sp|Q6P3W	1	2	2	2	3.9	103.71	0	0	79993000	0	122630000	0
sp|Q6P3X	TTC27	sp|Q6P3X	1	4	4	4	5.9	96.631	44877000	90812000	158620000	0	0	0
sp|Q6P58	FAHD1	sp|Q6P58	1	6	6	6	31	24.843	342880000	172000000	277980000	350200000	310500000	0
sp|Q6P6C	ALKBH5	sp|Q6P6C	1	1	1	1	7.4	44.255	0	0	0	0	0	51360000
sp|Q6P99	PDXDC1	sp|Q6P99	2	14	14	14	22	86.706	521900000	607860000	655020000	611230000	770760000	515960000
sp|Q6PCE	PGM2L1	sp|Q6PCE	1	3	3	3	8.4	70.441	65194000	65507000	0	85566000	63884000	58589000
sp|Q6PD6	CTR9	sp|Q6PD6	1	10	10	10	11	133.5	496760000	445790000	551120000	524200000	443540000	443830000
sp|Q6PGF	TTC37	sp|Q6PGF	1	15	15	15	15	175.48	491800000	631910000	494870000	458240000	477990000	496730000
sp|Q6PI48	DARS2	sp|Q6PI48	1	11	11	11	23	73.562	457450000	322630000	412580000	404580000	388610000	380680000
sp|Q6PI78	TMEM65	sp|Q6PI78	1	2	2	2	9.2	25.498	0	0	0	0	0	77694000
sp|Q6PID	TTC33	sp|Q6PID	1	2	2	2	18	29.411	0	88754000	125420000	0	0	0
sp|Q6PIU	NCEH1	sp|Q6PIU	1	9	9	9	30	45.807	368650000	463550000	386370000	475160000	652080000	545330000
sp|Q6PJG	ELMSAN1	sp|Q6PJG	1	2	2	2	3.5	114.99	0	0	0	0	0	13983000
sp|Q6PJG	BRAT1	sp|Q6PJG	1	9	9	9	21	88.118	244050000	307580000	156240000	163350000	115920000	219540000
sp|Q6PJT	ZC3H14	sp|Q6PJT	1	6	6	6	13	82.875	142780000	243480000	183890000	270870000	238150000	146180000
sp|Q6PKG	LARP1	sp|Q6PKG	2	24	24	24	36	123.51	3265000000	3266100000	3075800000	2820400000	2925900000	3128800000
sp|Q6PL1	ATAD2	sp|Q6PL1	1	2	2	2	2	158.55	0	0	44609000	0	0	0
sp|Q6QNY	BLOC1S3	sp|Q6QNY	1	1	1	1	9.4	21.255	0	0	0	0	0	0
sp|Q6QNY	BLOC1S2	sp|Q6QNY	1	1	1	1	7.7	15.961	0	0	0	0	0	33219000
sp|Q6RFH	WDR74	sp|Q6RFH	1	6	6	6	21	42.441	242240000	249170000	260500000	284530000	319380000	239170000
sp|Q6RW	AGTRAP	sp|Q6RW	1	2	2	2	25	17.419	234860000	188830000	294590000	0	259630000	236360000
sp|Q6S8J	POTEE	sp|Q6S8J	2	11	2	0	12	121.36	572230000	286080000	518030000	763040000	454180000	469850000
sp|Q6TFL	CCDC171	sp|Q6TFL	1	1	1	1	1.4	152.81	0	0	0	0	0	129060000
sp|Q6UB3	MTHFD1L	sp|Q6UB3	1	20	19	19	25	105.79	1351300000	1181400000	1398800000	1557900000	1581400000	1225800000
sp|Q6UN1	FIP1L1	sp|Q6UN1	1	3	3	3	8.4	66.526	176570000	134030000	0	177950000	176720000	170190000
sp|Q6UW	KDELC1	sp|Q6UW	1	2	2	2	6.6	58.042	0	0	0	0	109000000	0
sp|Q6UW	TMEM205	sp|Q6UW	1	2	2	2	16	21.198	141430000	252080000	126280000	0	294500000	345850000
sp|Q6UW	UQCC3	sp|Q6UW	1	1	1	1	24	10.081	0	0	0	0	0	22967000
sp|Q6UW	LRSAM1	sp|Q6UW	1	4	4	4	7.7	83.593	118210000	170240000	160430000	118920000	0	168130000
sp|Q6UW	SPINK6	sp|Q6UW	1	1	1	1	20	8.5849	600770000	544960000	627970000	351020000	484430000	499840000
sp|Q6UW	LCLAT1	sp|Q6UW	1	2	2	2	8.2	48.92	0	0	0	0	0	125020000

sp	Q6UX0	CWC27	sp	Q6UX0	1	7	7	7	31	53.846	309170000	220120000	217650000	236310000	219750000	225090000
sp	Q6UX6	DRAM2	sp	Q6UX6	1	1	1	1	8.3	29.766	0	0	0	0	0	41389000
sp	Q6UXH	CRELD2	sp	Q6UXH	1	2	2	2	7.1	38.191	0	0	0	0	0	84008000
sp	Q6UXN	WDR82	sp	Q6UXN	1	9	9	9	36	35.079	1007300000	840730000	1273500000	714140000	698530000	471250000
sp	Q6UXV	APOOL	sp	Q6UXV	1	4	4	4	20	29.159	127500000	0	0	0	0	0
sp	Q6VN2	RANBP10	sp	Q6VN2	1	1	1	1	2.3	67.256	0	0	0	0	0	119250000
sp	Q6WC	MPRIP	sp	Q6WC	1	17	17	17	26	116.53	669320000	670140000	680760000	702740000	600960000	602640000
sp	Q6WK	RAB11FIP	sp	Q6WK	1	2	2	1	1.6	137.17	357010000	370230000	341750000	0	0	324330000
sp	Q6XQN	NAPRT	sp	Q6XQN	1	6	6	6	18	57.578	210380000	267720000	288830000	299320000	262770000	231030000
sp	Q6XZF	DNMBP	sp	Q6XZF	1	2	2	2	1.4	177.35	0	0	0	54164000	0	0
sp	Q6Y1H	HACD2	sp	Q6Y1H	1	1	1	1	3.9	28.368	0	0	0	0	0	46876000
sp	Q6Y7W	GIGYF2	sp	Q6Y7W	1	9	9	9	12	150.07	398940000	342600000	389890000	336300000	383660000	348040000
sp	Q6YHK	CD109	sp	Q6YHK	1	5	5	5	5.6	161.69	129930000	134810000	102440000	166130000	347880000	153150000
sp	Q6YN1	HSDL2	sp	Q6YN1	1	15	15	15	42	45.394	1085000000	1051800000	1086000000	1052900000	1110000000	1119700000
sp	Q6YP2	KYAT3	sp	Q6YP2	1	6	6	6	19	51.4	153010000	177810000	164040000	189800000	162880000	155460000
sp	Q6ZNE	NFXL1	sp	Q6ZNE	1	1	1	1	2.6	101.34	0	0	0	67456000	0	0
sp	Q6ZRF	QSOX2	sp	Q6ZRF	1	5	5	5	11	77.528	110050000	223480000	196420000	111160000	88479000	127580000
sp	Q6ZRS	SRCAP	sp	Q6ZRS	1	6	6	6	2.1	343.55	0	1130400000	1279400000	0	1479800000	0
sp	Q6ZS1	RIPOR1	sp	Q6ZS1	1	1	1	1	1.3	132.31	0	0	0	23861000	0	0
sp	Q6ZSJ	C1orf122	sp	Q6ZSJ	1	1	1	1	11	11.471	0	0	0	0	0	200020000
sp	Q6ZUX	LHFPL2	sp	Q6ZUX	1	1	1	1	6.6	24.486	0	0	0	0	102160000	0
sp	Q6ZVM	TOM1L2	sp	Q6ZVM	1	4	4	4	14	55.556	59768000	89553000	68345000	112480000	82810000	56311000
sp	Q6ZW	SYDE1	sp	Q6ZW	1	1	1	1	2.2	79.792	0	45213000	0	0	0	0
sp	Q6ZXV	TMTC3	sp	Q6ZXV	1	4	4	4	5.8	104.01	0	0	497880000	0	0	617620000
sp	Q6ZYL	GTF2H5	sp	Q6ZYL	1	1	1	1	21	8.0533	0	0	0	0	0	20840000
sp	Q70CQ	USP34	sp	Q70CQ	1	3	3	3	1.6	404.23	0	104210000	0	0	0	0
sp	Q70E7	RAPH1	sp	Q70E7	1	3	3	3	3.5	135.25	0	0	0	0	193600000	0
sp	Q70J9	UNC13D	sp	Q70J9	1	9	9	9	13	123.28	504680000	348290000	419740000	334910000	393970000	342330000
sp	Q70UQ	IKBIP	sp	Q70UQ	1	8	8	8	26	39.309	813760000	743670000	747970000	886720000	1029100000	878920000
sp	Q712K	UBE2R2	sp	Q712K	1	3	3	2	12	27.166	0	0	0	0	0	16656000
sp	Q71DI	HIST2H3A	sp	Q71DI	1	5	1	1	46	15.388	17472000	26395000	62619000	105090000	0	12061000
sp	Q71RC	LARP4	sp	Q71RC	1	6	6	6	13	80.595	333950000	442530000	348700000	334550000	280290000	335880000
sp	Q71UM	RPS27L	sp	Q71UM	1	5	2	2	41	9.4771	577230000	548670000	719380000	564690000	813020000	976190000
sp	Q76FK	NOL8	sp	Q76FK	1	2	2	2	3.3	131.61	0	80716000	0	0	42399000	0
sp	Q7KZ8	SUPT6H	sp	Q7KZ8	1	16	16	16	14	199.07	694260000	631590000	739080000	648330000	633930000	625930000
sp	Q7KZF	SND1	sp	Q7KZF	1	40	40	40	55	102	1.1107E+10	1.1435E+10	1.0058E+10	10868000000	11939000000	1.1942E+10
sp	Q7KZI	MARK2	sp	Q7KZI	4	4	4	4	8.4	87.91	0	0	0	125290000	130690000	119490000
sp	Q7KZN	COX15	sp	Q7KZN	1	4	4	4	13	46.03	251080000	180890000	226250000	321340000	198080000	241670000
sp	Q7L01	DDX46	sp	Q7L01	1	26	26	26	31	117.36	2146900000	2280800000	2010600000	1966800000	2169300000	2007000000

sp|Q7L0Y	TRMT10C	sp|Q7L0Y	1	16	16	16	51	47.346	740090000	737900000	708520000	825830000	656250000	758790000
sp|Q7L1Q	BZW1	sp|Q7L1Q	1	16	15	15	38	48.043	5506000000	5651300000	5195600000	5554200000	5387500000	5946100000
sp|Q7L2E	DHX30	sp|Q7L2E	1	27	27	27	28	133.94	1697800000	1523700000	1563400000	1416800000	1255400000	1498600000
sp|Q7L2H	EIF3M	sp|Q7L2H	1	8	8	8	29	42.502	1875100000	1961400000	1552600000	1489000000	1900900000	1741600000
sp|Q7L2J(	MEPCE	sp|Q7L2J(	1	3	3	3	9.1	74.354	67338000	79603000	85368000	92970000	94270000	132510000
sp|Q7L3B	CDC37L1	sp|Q7L3B	1	1	1	1	4.5	38.834	0	0	0	0	24098000	0
sp|Q7L4I2	RSRC2	sp|Q7L4I2	1	2	2	2	8.8	50.559	0	74434000	49252000	0	73705000	74833000
sp|Q7L52	RRAGA	sp|Q7L52	2	4	4	4	17	36.566	0	0	0	0	0	315210000
sp|Q7L57	CYFIP1	sp|Q7L57	2	13	13	13	12	145.18	874240000	743350000	728560000	706110000	779300000	779640000
sp|Q7L5D	GET4	sp|Q7L5D	1	2	2	2	8.6	36.504	0	0	0	0	0	61014000
sp|Q7L5N	COPS6	sp|Q7L5N	1	8	8	8	35	36.163	1255600000	1514800000	1291500000	1038100000	1142000000	1055700000
sp|Q7L5N	LPCAT2	sp|Q7L5N	1	8	8	8	22	60.207	250290000	233850000	226490000	177630000	185450000	251840000
sp|Q7L5Y	ENOSF1	sp|Q7L5Y	1	1	1	1	3.8	49.786	0	0	0	0	18712000	0
sp|Q9H2K	TAOK3	sp|Q9H2K	2	1	1	1	1.4	105.4	0	0	0	0	0	62086000
sp|Q7LBC	KDM3B	sp|Q7LBC	1	6	6	6	7.2	191.58	198700000	251710000	211640000	148490000	231690000	161230000
sp|Q7LBR	CHMP1B	sp|Q7LBR	1	2	2	2	7.5	22.109	133290000	0	121130000	155590000	0	125960000
sp|Q7LGA	HS2ST1	sp|Q7LGA	1	4	4	4	15	41.881	123690000	0	0	325660000	119430000	0
sp|Q7RTV	PHF5A	sp|Q7RTV	1	5	5	5	48	12.405	718070000	977540000	843280000	857800000	1102100000	751890000
sp|Q7Z2E	APTX	sp|Q7Z2E	1	2	2	2	11	40.74	78807000	0	0	68663000	0	0
sp|Q7Z2K	ERMP1	sp|Q7Z2K	1	5	5	5	6	100.23	124200000	0	0	0	0	0
sp|Q7Z2T	TRMT1L	sp|Q7Z2T	1	4	4	4	10	81.746	0	0	0	201500000	0	0
sp|Q7Z2W	ZC3HAV1	sp|Q7Z2W	1	26	26	26	41	101.43	2989500000	2993900000	2676100000	3133900000	3633300000	4062000000
sp|Q7Z2W	MRPL21	sp|Q7Z2W	1	4	4	4	26	22.814	248270000	239650000	184610000	200320000	320490000	203900000
sp|Q7Z2Z	EFL1	sp|Q7Z2Z	1	8	8	8	12	125.43	137360000	139620000	158320000	127130000	111610000	100380000
sp|Q7Z3B	NUP54	sp|Q7Z3B	1	4	4	4	9.7	55.435	231950000	280450000	223890000	311890000	351130000	226180000
sp|Q7Z3C	ATG9A	sp|Q7Z3C	1	3	3	3	4.8	94.446	0	0	0	0	127960000	83567000
sp|Q7Z3E	ARMC9	sp|Q7Z3E	1	1	1	1	1.8	91.818	0	36970000	0	0	0	0
sp|Q7Z3J	C16orf62	sp|Q7Z3J	1	6	6	6	9	109.56	122720000	100480000	97311000	114290000	0	0
sp|Q7Z3K	POGZ	sp|Q7Z3K	1	5	5	5	5.5	155.34	135130000	144160000	177540000	113690000	147580000	185220000
sp|Q7Z3T	ZFYVE16	sp|Q7Z3T	1	2	2	2	2	168.9	0	0	0	51578000	0	0
sp|Q7Z3U	MON2	sp|Q7Z3U	1	2	2	2	1.6	190.36	0	41718000	34522000	39185000	0	35968000
sp|Q7Z40	MYH14	sp|Q7Z40	1	9	1	1	4.4	227.87	0	0	0	0	0	423730000
sp|Q7Z41	NUFIP2	sp|Q7Z41	1	8	8	8	22	76.12	501990000	464090000	431350000	459470000	627970000	618070000
sp|Q7Z42	SZRD1	sp|Q7Z42	1	1	1	1	14	16.997	0	0	0	0	0	403920000
sp|Q7Z43	MAVS	sp|Q7Z43	1	1	1	1	2.6	56.527	0	0	0	0	64839000	0
sp|Q7Z46	CLASP1	sp|Q7Z46	1	11	9	9	10	169.45	195700000	141730000	130930000	138980000	129730000	94464000
sp|Q7Z47	DHX29	sp|Q7Z47	1	5	5	5	5.5	155.23	221950000	134150000	169510000	174230000	152740000	0
sp|Q7Z4G	COMMD6	sp|Q7Z4G	1	1	1	1	15	9.638	0	0	0	0	0	69940000
sp|Q7Z4G	TRMT11	sp|Q7Z4G	1	1	1	1	2.6	53.42	0	0	0	0	0	22765000

sp|Q7Z4H	KDELC2	sp|Q7Z4H	1	2	2	2	6.5	58.572	75342000	54486000	0	75416000	0	86533000
sp|Q7Z4Q	HEATR3	sp|Q7Z4Q	1	5	5	5	10	74.582	167010000	103470000	143290000	150870000	150670000	157900000
sp|Q7Z4S	KIF21A	sp|Q7Z4S	4	12	12	12	11	187.18	326540000	219550000	393790000	496820000	411250000	202070000
sp|Q7Z4V	HDGFL2	sp|Q7Z4V	1	4	3	3	7.6	74.316	0	0	368010000	0	200010000	171620000
sp|Q7Z4W	DCXR	sp|Q7Z4W	1	7	7	7	42	25.913	248680000	181130000	253820000	230440000	379190000	218440000
sp|Q7Z5K	WAPL	sp|Q7Z5K	1	4	4	4	5.4	132.94	195910000	174980000	131790000	82945000	0	0
sp|Q7Z5L	IRF2BP2	sp|Q7Z5L	1	11	11	8	34	61.024	1089900000	937770000	919090000	918880000	1041600000	862120000
sp|Q7Z6B	SRGAP1	sp|Q7Z6B	1	2	2	1	2	124.26	109740000	0	0	0	0	0
sp|Q7Z6E	RBBP6	sp|Q7Z6E	1	3	3	3	2.1	201.56	87329000	0	0	0	0	0
sp|Q7Z6J	SH3RF1	sp|Q7Z6J	1	3	3	3	5	93.128	0	210880000	205110000	192050000	280820000	0
sp|Q7Z6M	RABEPK	sp|Q7Z6M	1	3	3	3	13	40.564	140590000	0	0	0	0	111660000
sp|Q7Z6Z	HUWE1	sp|Q7Z6Z	1	55	55	55	20	481.89	4213000000	4356500000	4116300000	4057000000	4147700000	3912600000
sp|Q7Z73	YTHDF3	sp|Q7Z73	1	11	7	5	21	63.86	408350000	351410000	307610000	535920000	401500000	349190000
sp|Q7Z7A	CNTRL	sp|Q7Z7A	1	2	2	2	0.9	268.88	0	0	1824000000	0	0	0
sp|Q7Z7E	UBE2Q1	sp|Q7Z7E	1	2	2	2	9.2	46.126	225560000	122460000	195490000	317570000	192870000	191370000
sp|Q7Z7F	MRPL55	sp|Q7Z7F	1	2	2	2	19	15.128	0	0	0	0	29472000	44987000
sp|Q7Z7H	TMED4	sp|Q7Z7H	1	4	3	3	23	25.943	123800000	0	142660000	169680000	125280000	0
sp|Q7Z7H	MRPL10	sp|Q7Z7H	1	1	1	1	7.3	29.282	33071000	39425000	109160000	39700000	28312000	42293000
sp|Q7Z7K	CMC1	sp|Q7Z7K	1	1	1	1	16	12.49	54393000	0	0	0	0	0
sp|Q7Z7K	CENPV	sp|Q7Z7K	1	2	2	2	9.8	29.946	0	0	0	93388000	0	0
sp|Q86SF	GALNT7	sp|Q86SF	1	12	12	12	23	75.388	1005100000	756130000	1029900000	970900000	991230000	1159700000
sp|Q86SX	GLRX5	sp|Q86SX	1	3	3	3	28	16.628	336680000	366000000	336530000	371940000	423260000	287880000
sp|Q86TB	PATL1	sp|Q86TB	1	3	3	3	7.1	86.849	73819000	0	0	0	0	0
sp|Q86TG	PEG10	sp|Q86TG	1	3	3	3	8.9	80.172	204900000	0	158100000	262920000	200890000	155680000
sp|Q86TI2	DPP9	sp|Q86TI2	1	5	5	5	7.6	98.262	83493000	91598000	0	0	0	103630000
sp|Q86TM	SYVN1	sp|Q86TM	1	1	1	1	4.1	67.684	0	0	0	0	0	30425000
sp|Q86TS	MRPL52	sp|Q86TS	1	2	2	2	27	13.664	118210000	100910000	90326000	77611000	126470000	99537000
sp|Q86TU	SETD3	sp|Q86TU	1	6	6	6	14	67.256	230790000	238700000	250830000	245480000	272200000	234760000
sp|Q86TX	ACOT1	sp|Q86TX	2	8	8	8	25	46.277	372050000	343500000	317930000	303370000	379770000	363470000
sp|Q86U0	RBM23	sp|Q86U0	1	1	1	1	2.5	48.73	0	0	0	0	2667200	0
sp|Q86U3	NOP9	sp|Q86U3	1	5	5	5	12	69.437	0	252580000	231330000	0	261310000	0
sp|Q86U4	PABPN1	sp|Q86U4	2	5	5	5	16	32.749	667530000	716670000	729470000	679870000	585160000	594600000
sp|Q86U4	METTL3	sp|Q86U4	1	1	1	1	2.1	64.473	0	0	0	0	0	70627000
sp|Q86U8	PBRM1	sp|Q86U8	1	6	6	6	5.6	192.95	126130000	92057000	119400000	127570000	157900000	121380000
sp|Q86U9	YRDC	sp|Q86U9	1	3	3	3	14	29.328	193780000	177400000	0	0	130100000	0
sp|Q86UE	MTDH	sp|Q86UE	1	17	17	17	41	63.836	1777000000	2000000000	1458200000	1599700000	1852600000	1483700000
sp|Q86UK	ZNF598	sp|Q86UK	1	3	3	3	5.1	98.636	112510000	151700000	0	97627000	0	0
sp|Q86UL	GPAT4	sp|Q86UL	1	1	1	1	2.6	52.07	0	0	0	0	0	34613000
sp|Q86UP	KTN1	sp|Q86UP	2	51	51	51	44	156.27	6666800000	7116300000	5862900000	6043100000	6276700000	6196100000

sp|Q86UP	ZFHX4	sp|Q86UP	1	1	1	1	0.6	393.73	0	0	0	0	651400000	0
sp|Q86UV	USP48	sp|Q86UV	1	6	6	6	9	119.03	332780000	467230000	296600000	404720000	295390000	154040000
sp|Q86UY	NAA40	sp|Q86UY	1	1	1	1	5.1	27.194	0	0	0	0	0	50605000
sp|Q86V2	AACS	sp|Q86V2	1	4	4	4	5.8	75.143	70636000	56172000	48048000	28958000	0	0
sp|Q86V4	LUZP1	sp|Q86V4	1	9	9	9	10	120.27	318930000	180820000	199320000	172260000	236470000	0
sp|Q86V8	ALYREF	sp|Q86V8	1	7	7	7	35	26.888	4419100000	4304500000	4485000000	3472800000	4122900000	4478300000
sp|Q86V8	GPR180	sp|Q86V8	1	1	1	1	3.4	49.395	0	0	0	0	0	36503000
sp|Q86VI3	IQGAP3	sp|Q86VI3	1	3	1	1	2.4	184.7	0	0	0	0	0	7928700
sp|Q86VM	ZC3H18	sp|Q86VM	1	4	4	4	7.1	106.38	97790000	117940000	159820000	142960000	210550000	150990000
sp|Q86VN	VPS36	sp|Q86VN	1	1	1	1	3.4	43.816	0	0	0	0	0	35048000
sp|Q86VP	CAND1	sp|Q86VP	2	37	37	37	38	136.37	5914600000	5645600000	5528500000	5166600000	5346800000	5238500000
sp|Q86VS	HOOK3	sp|Q86VS	1	2	2	2	5.2	83.125	0	0	0	0	0	413470000
sp|Q86VU	COMTD1	sp|Q86VU	1	1	1	1	5.7	28.808	0	0	0	0	0	38934000
sp|Q86VV	RTTN	sp|Q86VV	1	1	1	1	0.3	248.63	0	0	0	0	0	515810000
sp|Q86VW	SESTD1	sp|Q86VW	1	2	2	2	4	79.347	0	0	0	35296000	0	0
sp|Q86VX	COMMD7	sp|Q86VX	1	1	1	1	4.5	22.54	0	0	0	0	15940000	0
sp|Q86VZ	SGMS1	sp|Q86VZ	1	2	2	2	6.1	48.616	0	0	0	58319000	152740000	0
sp|Q86W4	THOC6	sp|Q86W4	1	7	7	7	33	37.535	372750000	380990000	337260000	374820000	337190000	322000000
sp|Q86W5	METTL16	sp|Q86W5	1	4	4	4	7.3	63.62	91726000	70946000	108570000	106880000	83774000	132270000
sp|Q86W9	PPFIBP1	sp|Q86W9	1	7	7	7	10	114.02	209910000	213560000	210210000	158020000	169410000	201390000
sp|Q86WB	ZC3HC1	sp|Q86WB	1	2	2	2	6.6	55.261	0	0	59581000	0	0	0
sp|Q86WC	OSTM1	sp|Q86WC	1	1	1	1	3.3	37.256	0	0	0	0	0	43693000
sp|Q86WC	NR2C2AP	sp|Q86WC	1	3	3	3	35	15.876	90617000	0	67015000	87499000	88248000	0
sp|Q86WR	CCDC25	sp|Q86WR	1	3	3	3	19	24.479	0	0	0	0	101210000	0
sp|Q86X5	CARM1	sp|Q86X5	1	3	3	3	9.7	65.853	181180000	200160000	0	170790000	181150000	0
sp|Q86X7	NIT1	sp|Q86X7	1	3	3	3	9.5	35.896	207380000	0	0	0	257370000	0
sp|Q86X8	COMMD2	sp|Q86X8	1	3	3	3	26	22.745	0	58022000	143990000	0	0	66863000
sp|Q86XA	HEATR5A	sp|Q86XA	1	2	2	2	1.6	222	0	0	55372000	0	0	0
sp|Q86XI2	NCAPG2	sp|Q86XI2	1	5	5	5	6.1	130.96	106530000	158330000	109360000	105670000	142470000	80794000
sp|Q86XL	ANKLE2	sp|Q86XL	1	8	8	8	14	104.11	1191900000	1093800000	1443900000	1237800000	1317000000	958960000
sp|Q86XP	DDX42	sp|Q86XP	1	19	19	19	31	102.97	1541600000	1361500000	1458800000	1432900000	1343700000	1500800000
sp|Q86XZ	SPATS2	sp|Q86XZ	1	2	2	2	6.4	59.544	0	84432000	0	0	0	0
sp|Q86Y3	NDUFA11	sp|Q86Y3	1	4	4	4	47	14.852	226100000	235640000	256210000	192150000	274270000	240110000
sp|Q86Y5	DNAAF5	sp|Q86Y5	1	13	13	13	23	93.52	783540000	791320000	725170000	591420000	686520000	601640000
sp|Q86Y8	STX12	sp|Q86Y8	1	3	3	3	15	31.642	0	0	161120000	159550000	0	0
sp|Q86YQ	CPNE8	sp|Q86YQ	3	4	3	3	9.2	63.107	0	179640000	0	97172000	143880000	121360000
sp|Q8IU81	IRF2BP1	sp|Q8IU81	1	4	3	3	8.9	61.687	0	0	0	0	76319000	0
sp|Q8IU85	CAMK1D	sp|Q8IU85	2	4	4	4	14	42.913	86232000	103130000	108830000	90999000	107830000	87460000
sp|Q8IUD	ERC1	sp|Q8IUD	1	10	10	10	13	128.08	411270000	316340000	341630000	309790000	438080000	505140000

sp|Q8IUE(	HIST2H2A	sp|Q8IUE(	1	2	1	1	12	13.995	0	0	0	0	0	1362900000
sp|Q8IUF8	RIOX2	sp|Q8IUF8	1	2	2	2	8.8	52.8	0	0	0	0	22206000	0
sp|Q8IUI8	CRLF3	sp|Q8IUI8	1	1	1	1	5.7	49.765	0	0	0	0	50009000	0
sp|Q8IUR(	TRAPPC5	sp|Q8IUR(	1	3	3	3	27	20.783	0	133770000	0	0	0	0
sp|Q8IV08	PLD3	sp|Q8IV08	1	4	4	4	11	54.705	203830000	172620000	180170000	164040000	141490000	153470000
sp|Q8IV48	ERI1	sp|Q8IV48	1	1	1	1	4.3	40.063	0	0	0	0	0	166420000
sp|Q8IVD9	NUDCD3	sp|Q8IVD9	1	3	3	3	12	40.822	0	111240000	93333000	0	74483000	0
sp|Q8IVF2	AHNAK2	sp|Q8IVF2	1	61	59	59	29	616.62	5559200000	5515100000	5419300000	4804500000	4969800000	5295000000
sp|Q8IVL5	P3H2	sp|Q8IVL5	1	2	1	1	3.5	80.984	0	0	0	0	21794000	0
sp|Q8IVL6	P3H3	sp|Q8IVL6	1	7	7	7	17	81.836	95502000	153920000	150990000	190560000	182680000	149220000
sp|Q8IVM	CCDC50	sp|Q8IVM	1	4	4	4	17	35.822	0	294420000	151970000	155590000	187400000	175430000
sp|Q8IVP5	FUNDC1	sp|Q8IVP5	1	1	1	1	11	17.177	14175000	0	0	18152000	15626000	0
sp|Q8IVS2	MCAT	sp|Q8IVS2	1	6	6	6	30	42.961	299970000	311120000	0	201500000	265610000	0
sp|Q8IVT2	MISP	sp|Q8IVT2	1	16	16	16	38	75.356	1238700000	1040900000	1194200000	1122200000	1311200000	1119300000
sp|Q8IVV2	LOXHD1	sp|Q8IVV2	1	1	1	1	0.4	221.94	0	0	0	0	0	29723000
sp|Q8IW1	APLF	sp|Q8IW1	1	1	1	1	3.9	56.955	0	0	0	0	0	134880000
sp|Q8IWA	WDR75	sp|Q8IWA	1	7	7	7	12	94.498	183910000	141240000	147070000	117360000	124760000	228590000
sp|Q8IWA	MFN1	sp|Q8IWA	1	2	2	2	4.9	84.159	103230000	0	0	0	0	0
sp|Q8IWB	ITPRIP	sp|Q8IWB	2	8	8	8	25	62.059	130550000	141510000	133760000	287810000	213340000	229830000
sp|Q8IWB	WDFY1	sp|Q8IWB	1	7	7	7	23	46.323	359900000	323140000	366850000	333330000	386620000	362720000
sp|Q8IWE	FAM114A	sp|Q8IWE	1	6	6	6	15	60.741	344030000	368350000	401670000	323440000	461480000	335260000
sp|Q8IWJ	GCC2	sp|Q8IWJ	1	3	3	3	2	195.91	0	0	0	0	0	80187000
sp|Q8IWR	ZC3H7A	sp|Q8IWR	1	3	3	3	5.9	110.54	43008000	0	0	0	0	0
sp|Q8IWS	PHF6	sp|Q8IWS	1	9	9	9	32	41.29	947010000	1068900000	1028400000	792740000	860550000	967730000
sp|Q8IWT	ZBTB8OS	sp|Q8IWT	1	1	1	1	10	19.491	0	0	0	0	29115000	0
sp|Q8IWT	LRRC8A	sp|Q8IWT	1	6	6	5	8.6	94.198	78750000	95916000	0	107150000	50481000	0
sp|Q8IWV	UBR1	sp|Q8IWV	1	2	2	2	2.1	200.21	0	0	0	0	23066000	0
sp|Q8IWX	CHERP	sp|Q8IWX	1	9	9	9	20	103.7	399480000	475040000	440430000	419190000	403380000	520980000
sp|Q8IWZ	ANKHD1	sp|Q8IWZ	1	11	11	7	7.1	269.45	316370000	340710000	268030000	294250000	340680000	267230000
sp|Q8IWZ	SUGP1	sp|Q8IWZ	1	1	1	1	2.8	72.47	0	0	0	0	45714000	0
sp|Q8IX01	SUGP2	sp|Q8IX01	1	5	5	5	6.5	120.21	76976000	105660000	0	99958000	72072000	97828000
sp|Q8IX03	WWC1	sp|Q8IX03	1	1	1	1	3	125.3	0	0	0	0	0	6694700
sp|Q8IX12	CCAR1	sp|Q8IX12	1	8	8	8	11	132.82	432510000	511140000	432560000	477190000	559490000	653460000
sp|Q8IX18	DHX40	sp|Q8IX18	1	1	1	1	1.7	88.559	0	0	0	0	13900000	0
sp|Q8IXB1	DNAJC10	sp|Q8IXB1	1	8	8	8	14	91.079	370230000	467780000	344910000	450170000	471190000	414650000
sp|Q8IXH7	NELFCD	sp|Q8IXH7	1	8	8	8	17	66.246	217220000	191240000	406620000	180230000	209780000	344250000
sp|Q8IXI1	RHOT2	sp|Q8IXI1	1	6	6	5	19	68.117	317110000	401890000	384950000	475760000	522820000	508750000
sp|Q8IXI2	RHOT1	sp|Q8IXI2	1	2	1	1	4.4	70.783	0	0	0	0	0	28434000
sp|Q8IXM	MRPL41	sp|Q8IXM	1	3	3	3	42	15.383	198480000	142700000	199470000	256720000	297930000	250500000

sp|Q8IXQ3	C9orf40	sp|Q8IXQ3	1	1	1	1	14	21.063	0	0	0	0	0	14356000
sp|Q8IXS6	PALM2	sp|Q8IXS6	1	3	3	3	12	42.185	0	0	0	105570000	137980000	109730000
sp|Q8IXT5	RBM12B	sp|Q8IXT5	1	2	2	2	2.3	118.1	0	0	0	0	23131000	0
sp|Q8IXU6	SLC35F2	sp|Q8IXU6	1	2	2	2	14	41.211	0	0	0	39790000	52904000	0
sp|Q8IY17	PNPLA6	sp|Q8IY17	1	2	2	2	2.4	149.99	0	0	0	0	0	23031000
sp|Q8IY21	DDX60	sp|Q8IY21	1	3	3	3	2.2	197.85	33122000	38378000	0	0	0	0
sp|Q8IY37	DHX37	sp|Q8IY37	1	4	4	4	5.5	129.54	0	0	0	0	93395000	0
sp|Q8IY50	SLC35F3	sp|Q8IY50	1	1	1	1	1.7	46.817	0	0	0	0	0	1269500000
sp|Q8IY67	RAVER1	sp|Q8IY67	1	10	10	10	31	63.876	465760000	266550000	403930000	629470000	400980000	376840000
sp|Q8IY81	FTSJ3	sp|Q8IY81	1	16	16	16	31	96.557	981760000	1214100000	1108100000	1177700000	1036900000	1040400000
sp|Q8IY95	TMEM192	sp|Q8IY95	1	2	2	2	11	30.922	0	0	106310000	115900000	0	0
sp|Q8IYB3	SRRM1	sp|Q8IYB3	1	7	7	7	11	102.33	973950000	1037900000	780430000	823780000	1031600000	1014100000
sp|Q8IYB5	SMAP1	sp|Q8IYB5	1	2	2	2	7.5	50.386	0	0	50395000	78465000	0	0
sp|Q8IYB8	SUPV3L1	sp|Q8IYB8	1	3	3	3	4.5	87.99	0	124180000	0	0	0	0
sp|Q8IYD1	GSPT2	sp|Q8IYD1	1	8	1	1	13	68.882	0	558740000	362540000	407850000	0	0
sp|Q8IYI6	EXOC8	sp|Q8IYI6	1	3	3	3	8.3	81.798	0	0	0	97466000	0	0
sp|Q8IYL3	C1orf174	sp|Q8IYL3	1	1	1	1	5.8	25.977	0	0	0	0	0	104560000
sp|Q8IYS2	KIAA2013	sp|Q8IYS2	1	2	2	2	4.1	69.156	0	89954000	0	0	0	96466000
sp|Q8IYT4	KATNAL2	sp|Q8IYT4	1	1	1	1	2.2	61.252	0	0	0	0	0	1164400000
sp|Q8IYU8	MICU2	sp|Q8IYU8	1	1	1	1	3.9	49.666	0	0	0	0	0	48519000
sp|Q8IZ69	TRMT2A	sp|Q8IZ69	1	4	4	4	10	68.725	91841000	84360000	81905000	86962000	98590000	102820000
sp|Q8IZ83	ALDH16A	sp|Q8IZ83	1	2	2	2	6.7	85.126	0	0	0	0	0	59692000
sp|Q8IZH2	XRN1	sp|Q8IZH2	1	2	2	2	1.5	194.1	0	52827000	0	0	0	42672000
sp|Q8IZL8	PELP1	sp|Q8IZL8	1	13	13	13	18	119.7	1102800000	927710000	1168800000	1114200000	1158300000	1237800000
sp|Q8IZP0	ABI1	sp|Q8IZP0	1	4	4	4	13	55.08	0	0	0	0	0	168680000
sp|Q8IZW	TNS4	sp|Q8IZW	1	2	2	2	3.9	76.763	0	0	0	7691000	0	0
sp|Q8N0U	VKORC1L	sp|Q8N0U	1	3	3	3	15	19.835	139410000	102420000	0	232250000	156610000	0
sp|Q8N0X	SPART	sp|Q8N0X	1	6	6	6	14	72.832	434340000	444360000	460420000	365420000	401460000	0
sp|Q8N10	MIER1	sp|Q8N10	1	3	3	3	9.4	57.983	74117000	72643000	57749000	0	52593000	0
sp|Q8N12	RPTOR	sp|Q8N12	1	3	3	3	4	149.04	0	0	0	0	147150000	0
sp|Q8N12	CNPY4	sp|Q8N12	1	2	2	2	10	28.309	0	0	0	0	43667000	0
sp|Q8N16	CCAR2	sp|Q8N16	1	19	19	19	34	102.9	1481900000	1161500000	1580500000	1332900000	1617300000	1427000000
sp|Q8N18	NDUFAF2	sp|Q8N18	1	10	10	10	59	19.856	756370000	931470000	979920000	981550000	1094700000	966440000
sp|Q8N1B	VPS52	sp|Q8N1B	1	4	4	4	7.6	82.22	0	1027000000	0	0	0	0
sp|Q8N1F	NUP93	sp|Q8N1F	1	23	23	23	32	93.487	2446900000	2163700000	2298300000	2296400000	2695600000	2547400000
sp|Q8N1G	CMTR1	sp|Q8N1G	1	7	7	7	11	95.32	119970000	148730000	117570000	115670000	183640000	132100000
sp|Q8N1G	LRRC47	sp|Q8N1G	1	19	19	19	52	63.472	1884900000	1902400000	1771000000	1849300000	1884200000	1861900000
sp|Q8N20	INTS1	sp|Q8N20	1	1	1	1	0.8	244.29	0	0	0	0	10453000	0
sp|Q8N25	HIST3H2E	sp|Q8N25	1	6	1	1	41	13.908	0	0	0	0	0	135960000

sp|Q8N2F	ARMC10	sp|Q8N2F	1	2	2	2	8.2	37.54	0	0	28597000	0	0	0
sp|Q8N2G	GHDC	sp|Q8N2G	1	1	1	1	2.3	57.522	0	0	0	21173000	0	0
sp|Q8N2H	SYS1	sp|Q8N2H	1	1	1	1	14	17.615	0	0	0	0	0	21508000
sp|Q8N2U	TMEM256	sp|Q8N2U	1	1	1	1	7.1	11.741	0	0	0	0	124710000	0
sp|Q8N33	GPD1L	sp|Q8N33	1	2	2	2	6	38.418	0	151820000	95201000	110200000	0	176360000
sp|Q8N35	SLC35F6	sp|Q8N35	1	2	2	2	7	40.214	0	0	0	0	0	184410000
sp|Q8N39	ARHGAP1	sp|Q8N39	1	6	6	6	14	74.976	119800000	159360000	171700000	146290000	150410000	121000000
sp|Q8N3C	ASCC3	sp|Q8N3C	1	18	18	18	14	251.46	400560000	401530000	527650000	371150000	316250000	387180000
sp|Q8N3D	EHBP1L1	sp|Q8N3D	1	7	7	7	7.5	161.85	264850000	284810000	314190000	221600000	186830000	198310000
sp|Q8N3E	PLCD3	sp|Q8N3E	1	2	2	2	5.2	89.257	0	0	0	143670000	0	0
sp|Q8N3R	MPP5	sp|Q8N3R	1	2	2	2	3.9	77.293	0	0	0	0	0	16224000
sp|Q8N3U	STAG2	sp|Q8N3U	3	12	12	12	15	141.32	490220000	524940000	759650000	604000000	701120000	573910000
sp|Q8N3V	SYNPO	sp|Q8N3V	1	5	5	5	9.4	99.462	0	248560000	0	0	0	0
sp|Q8N3X	FNBP4	sp|Q8N3X	1	4	4	4	5.7	110.26	123260000	135440000	142510000	121170000	154520000	107600000
sp|Q8N4C	MINK1	sp|Q8N4C	1	2	1	1	1.5	149.82	0	0	0	0	27255000	0
sp|Q8N4H	TOMM5	sp|Q8N4H	1	3	3	3	29	6.0352	364300000	414060000	364280000	389380000	396340000	452170000
sp|Q8N4Q	ZADH2	sp|Q8N4Q	1	1	1	1	2.4	40.14	0	0	0	0	0	23459000
sp|Q8N4Q	CHCHD4	sp|Q8N4Q	1	5	5	5	46	15.996	366480000	247630000	318710000	282820000	500180000	443150000
sp|Q8N4V	MMGT1	sp|Q8N4V	1	3	3	3	25	14.686	196350000	154830000	96449000	119550000	141950000	117730000
sp|Q8N54	OGFOD1	sp|Q8N54	1	8	8	8	19	63.245	393820000	327640000	392330000	416650000	347620000	384470000
sp|Q8N55	AFAP1	sp|Q8N55	1	3	3	3	4.7	80.724	0	49471000	28640000	78229000	69629000	0
sp|Q8N58	TTC39C	sp|Q8N58	1	1	1	1	2.9	65.869	0	0	34350000	0	0	0
sp|Q8N5A	ZGPAT	sp|Q8N5A	1	2	2	2	5.6	57.358	460140000	497890000	0	349230000	280990000	472630000
sp|Q8N5G	SMIM20	sp|Q8N5G	1	1	1	1	21	7.7019	0	0	0	0	0	39058000
sp|Q8N5K	CISD2	sp|Q8N5K	1	4	4	4	39	15.278	397390000	387620000	364320000	506540000	391700000	468120000
sp|Q8N5L	RPP25L	sp|Q8N5L	1	1	1	1	14	17.631	0	0	0	0	0	13640000
sp|Q8N5M	TTC9C	sp|Q8N5M	1	4	4	4	27	20.012	580800000	371290000	466230000	0	397560000	455740000
sp|Q8N5M	JAGN1	sp|Q8N5M	1	2	2	2	13	21.125	308610000	346780000	0	342160000	331750000	413840000
sp|Q8N5N	MRPL50	sp|Q8N5N	1	3	3	3	28	18.325	0	114040000	0	207760000	508330000	0
sp|Q8N68	CPSF7	sp|Q8N68	1	8	8	8	22	52.049	378280000	469650000	379690000	545590000	454230000	418620000
sp|Q8N6H	ARFGAP2	sp|Q8N6H	1	9	9	9	23	56.72	306120000	277820000	270510000	272580000	240920000	311350000
sp|Q8N6L	KRTCAP2	sp|Q8N6L	1	1	1	1	13	14.678	0	0	0	0	0	189590000
sp|Q8N6M	OTUD6B	sp|Q8N6M	1	3	3	3	17	33.812	117180000	163570000	87284000	177490000	172380000	116070000
sp|Q8N6R	METTL13	sp|Q8N6R	1	2	2	2	5.4	78.767	0	0	0	0	0	51654000
sp|Q8N6T	ARFGAP1	sp|Q8N6T	1	8	8	8	31	44.667	686950000	649260000	631850000	736200000	803720000	662810000
sp|Q8N72	CDKN2A	sp|Q8N72	1	4	4	4	39	13.903	2798800000	3856700000	2610200000	1900100000	2298200000	1740500000
sp|Q8N76	EMC1	sp|Q8N76	1	9	9	9	13	111.76	600560000	520710000	632360000	704590000	621570000	671260000
sp|Q8N7H	PAF1	sp|Q8N7H	1	11	11	11	24	59.975	497680000	411160000	357310000	521540000	597700000	450510000
sp|Q8N80	UBR7	sp|Q8N80	1	5	5	5	16	47.998	162990000	209360000	130630000	139560000	142450000	153840000

sp|Q8N8S	ENAH	sp|Q8N8S	2	13	13	13	21	66.509	812010000	1081300000	923120000	855360000	877400000	978830000
sp|Q8N98	MRPL43	sp|Q8N98	1	3	3	3	14	23.431	205980000	225790000	215030000	244850000	0	138650000
sp|Q8N99	C12orf29	sp|Q8N99	1	3	3	3	15	37.49	818270000	539740000	522020000	956820000	540290000	448460000
sp|Q8N9A	CNEP1R1	sp|Q8N9A	1	1	1	1	8.8	14.267	0	0	0	0	182710000	0
sp|Q8N9N	LRRC57	sp|Q8N9N	1	2	2	2	8.4	26.754	0	0	0	85961000	0	117860000
sp|Q8N9Q	SREK1IP1	sp|Q8N9Q	1	1	1	1	7.7	18.177	0	0	0	0	0	52945000
sp|Q8N9T	KRI1	sp|Q8N9T	1	4	4	4	7	82.597	0	0	0	0	85400000	0
sp|Q8NAV	PRPF38A	sp|Q8NAV	1	5	5	5	21	37.476	398100000	0	0	0	383770000	0
sp|Q8NB1	MLKL	sp|Q8NB1	1	5	5	5	18	54.478	225780000	200380000	163070000	179310000	195930000	189810000
sp|Q8NB3	GATD1	sp|Q8NB3	1	1	1	1	4.5	23.297	0	0	0	0	18920000	0
sp|Q8NB4	ATP11C	sp|Q8NB4	1	1	1	1	1.6	129.48	0	0	0	0	44041000	0
sp|Q8NB9	SPATA5	sp|Q8NB9	1	8	7	7	13	97.903	324780000	252950000	309160000	400240000	294120000	295410000
sp|Q8NBF	NHLRC2	sp|Q8NBF	1	4	4	4	14	79.443	89553000	110910000	96724000	91877000	86179000	65651000
sp|Q8NBI	XXYLT1	sp|Q8NBI	1	1	1	1	3.3	43.806	0	0	0	0	38172000	0
sp|Q8NBJ	GOLM1	sp|Q8NBJ	1	5	5	5	17	45.333	199470000	244840000	280180000	194770000	0	287450000
sp|Q8NBJ	COLGALT	sp|Q8NBJ	1	19	19	19	29	71.635	2704300000	2519800000	2523600000	2959800000	2929300000	2854400000
sp|Q8NBJ	SUMF2	sp|Q8NBJ	1	4	4	4	17	33.843	325550000	462370000	396790000	317800000	442130000	279110000
sp|Q8NBK	SUMF1	sp|Q8NBK	1	1	1	1	4	40.556	0	0	0	0	0	55050000
sp|Q8NBL	POGLUT1	sp|Q8NBL	1	2	2	2	5.6	46.189	0	0	0	0	0	77358000
sp|Q8NBM	UBAC2	sp|Q8NBM	1	1	1	1	5.2	38.963	0	0	0	0	0	0
sp|Q8NBN	TMEM87A	sp|Q8NBN	1	3	3	3	7.9	63.429	0	0	0	307320000	0	0
sp|Q8NBN	RDH13	sp|Q8NBN	1	3	3	3	15	35.932	110830000	92140000	0	128930000	120310000	77301000
sp|Q8NBQ	HSD17B1	sp|Q8NBQ	1	3	3	3	14	32.935	174890000	186390000	232930000	202190000	212930000	241570000
sp|Q8NBS	TXNDC5	sp|Q8NBS	1	18	18	18	47	47.628	6475000000	8025500000	8291000000	6820800000	8091000000	8851300000
sp|Q8NBT	SPC24	sp|Q8NBT	1	6	6	6	59	22.478	420130000	476450000	431120000	473130000	314310000	220810000
sp|Q8NBU	ATAD1	sp|Q8NBU	1	5	5	5	17	40.744	285640000	45994000	223130000	0	229250000	282110000
sp|Q8NBX	SCCPDH	sp|Q8NBX	1	2	2	2	5.1	47.151	132120000	113210000	115860000	183240000	150540000	62786000
sp|Q8NC5	SERBP1	sp|Q8NC5	1	20	20	20	51	44.965	2.5927E+10	2.5124E+10	2.493E+10	21056000000	24755000000	2.1848E+10
sp|Q8NC5	LEMD2	sp|Q8NC5	1	4	4	4	15	56.974	154200000	0	139020000	154200000	175300000	166090000
sp|Q8NC6	NOA1	sp|Q8NC6	1	1	1	1	2.3	78.457	0	0	0	0	19034000	0
sp|Q8NCA	FAM98A	sp|Q8NCA	1	8	8	8	26	55.4	636230000	815660000	688810000	759460000	664090000	797360000
sp|Q8NCC	DAGLB	sp|Q8NCC	1	6	6	6	13	73.731	0	94455000	0	90519000	53786000	100910000
sp|Q8NCN	PDPR	sp|Q8NCN	1	4	4	4	6.7	99.363	78353000	51963000	94920000	71668000	0	57391000
sp|Q8NCU	C2CD4A	sp|Q8NCU	2	2	2	2	5.1	39.743	646640000	472190000	510020000	0	0	0
sp|Q8NCW	NAXE	sp|Q8NCW	1	7	7	7	37	31.674	514410000	621330000	678550000	576030000	724020000	765230000
sp|Q8ND2	RNF214	sp|Q8ND2	1	1	1	1	2	77.667	0	0	0	0	0	11992000
sp|Q8ND5	LSM14A	sp|Q8ND5	1	4	4	4	9.9	50.529	220460000	0	210700000	191880000	203480000	0
sp|Q8NDC	MAPK1IP1	sp|Q8NDC	1	1	1	1	6.9	24.269	0	0	0	0	0	181760000
sp|Q8NDX	PHC3	sp|Q8NDX	2	3	3	3	6.4	106.16	0	0	0	48103000	0	0

sp|Q8NDX	ZNF740	sp|Q8NDX	1	1	1	1	12	21.857	0	0	0	0	20011000	0
sp|Q8NE0	CNNM3	sp|Q8NE0	1	2	2	2	3.8	76.118	0	418920000	0	0	0	0
sp|Q8NE7	ABCF1	sp|Q8NE7	1	20	20	20	31	95.925	2503500000	1966900000	2416800000	2239800000	2856700000	2518800000
sp|Q8NE8	MCU	sp|Q8NE8	1	2	2	2	8	39.866	0	0	0	0	0	166750000
sp|Q8NEE	PIK3C3	sp|Q8NEE	1	3	3	3	5.2	101.55	0	50741000	0	41139000	0	34626000
sp|Q8NEJ	NGDN	sp|Q8NEJ	1	4	4	4	18	35.894	221170000	155580000	0	247280000	263620000	0
sp|Q8NEM	SHCBP1	sp|Q8NEM	1	6	6	6	9.2	75.69	0	113660000	0	0	0	0
sp|Q8NEW	SLC30A7	sp|Q8NEW	1	1	1	1	4	41.625	0	0	0	0	0	266720000
sp|Q8NEX	WFDC11	sp|Q8NEX	1	1	1	1	12	10.34	0	0	0	0	0	257620000
sp|Q8NEY	NAV1	sp|Q8NEY	1	2	2	2	1.7	202.47	0	0	0	0	50189000	53571000
sp|Q8NEY	PPHLN1	sp|Q8NEY	1	3	3	3	7.4	52.736	0	0	0	0	0	79578000
sp|Q8NEZ	VPS37A	sp|Q8NEZ	1	2	2	2	8.3	44.314	0	0	0	0	83733000	0
sp|Q8NEZ	FBXO22	sp|Q8NEZ	1	4	4	4	12	44.508	239440000	205270000	152740000	326720000	311620000	318710000
sp|Q8NF3	LPCAT1	sp|Q8NF3	1	12	12	12	27	59.151	1789900000	1900800000	1502600000	1814700000	1782300000	1916200000
sp|Q8NF6	ZMIZ2	sp|Q8NF6	1	2	2	2	4.9	96.536	0	0	39550000	45655000	0	0
sp|Q8NF9	SYNE1	sp|Q8NF9	1	4	4	4	0.6	1011.1	1606100000	2247800000	1500000000	2164000000	2091200000	0
sp|Q8NFC	BOD1L1	sp|Q8NFC	2	3	3	3	1.5	330.46	162500000	65247000	0	0	0	47436000
sp|Q8NFD	ARID1B	sp|Q8NFD	1	5	4	4	4.6	236.12	0	51259000	0	0	0	33802000
sp|Q8NFF	FLAD1	sp|Q8NFF	1	3	3	3	9.7	65.265	138210000	147500000	91882000	169550000	178630000	216970000
sp|Q8NFH	NUP43	sp|Q8NFH	1	5	5	5	19	42.15	424920000	332520000	442920000	390800000	457880000	453480000
sp|Q8NFH	NUP37	sp|Q8NFH	1	8	8	8	31	36.707	693440000	554000000	786940000	510020000	671330000	576700000
sp|Q8NFH	NUP35	sp|Q8NFH	1	5	5	5	25	34.773	453160000	549240000	759420000	832570000	804370000	676170000
sp|Q8NFJ	GPRC5A	sp|Q8NFJ	1	5	5	5	24	40.251	1287300000	1381500000	1213500000	971460000	1235100000	1102500000
sp|Q8NFQ	TOR1AIP2	sp|Q8NFQ	1	7	6	6	21	51.263	242610000	346960000	0	277960000	331310000	408660000
sp|Q8NFU	IPMK	sp|Q8NFU	1	1	1	1	2.2	47.221	0	0	0	0	0	123220000
sp|Q8NFV	ABHD11	sp|Q8NFV	1	3	3	3	9.2	34.69	0	56161000	0	192240000	51144000	0
sp|Q8NFW	CMAS	sp|Q8NFW	1	2	2	2	5.5	48.379	0	0	0	0	151420000	0
sp|Q8NHH	ATL2	sp|Q8NHH	1	5	5	5	12	66.228	0	99708000	117440000	123790000	145880000	164130000
sp|Q8NHQ	DDX55	sp|Q8NHQ	1	2	2	2	7	68.546	0	112890000	108940000	64343000	0	94511000
sp|Q8NI22	MCFD2	sp|Q8NI22	1	1	1	1	12	16.39	0	0	0	0	0	79103000
sp|Q8NI27	THOC2	sp|Q8NI27	1	9	9	9	7.2	182.77	329850000	345520000	335510000	340560000	411850000	259410000
sp|Q8NI36	WDR36	sp|Q8NI36	1	13	13	13	18	105.32	483590000	405720000	709930000	630340000	556050000	521830000
sp|Q8NI60	COQ8A	sp|Q8NI60	1	1	1	1	1.9	71.949	0	0	0	0	0	7095000
sp|Q8TAA	VANGL1	sp|Q8TAA	1	2	2	2	9	59.974	126460000	0	0	0	97739000	0
sp|Q8TAD	SLC30A5	sp|Q8TAD	1	1	1	1	3.1	84.046	0	63483000	0	0	0	0
sp|Q8TAE	GADD45G	sp|Q8TAE	1	5	5	5	28	25.384	252980000	238180000	0	0	208650000	248670000
sp|Q8TAF	WDR48	sp|Q8TAF	1	3	3	3	8.3	76.21	0	82604000	0	0	73646000	0
sp|Q8TAG	EXOC6	sp|Q8TAG	1	2	2	2	5.1	93.721	0	0	48281000	0	33330000	0
sp|Q8TAP	MPLKIP	sp|Q8TAP	1	1	1	1	14	19.147	0	0	0	0	34206000	0

sp|Q8TAQ	SMARCC2	sp|Q8TAQ	1	12	6	6	14	132.88	272960000	287730000	254270000	306390000	260150000	243020000
sp|Q8TAT	NPLOC4	sp|Q8TAT	1	15	15	15	42	68.119	1143300000	1110400000	1169800000	1228400000	1087400000	1227400000
sp|Q8TB3	NUBPL	sp|Q8TB3	1	1	1	1	6.3	34.082	0	0	0	0	0	20691000
sp|Q8TB6	SLC35B2	sp|Q8TB6	1	2	2	2	4.9	47.514	0	200430000	164490000	190760000	0	202890000
sp|Q8TBB	KLHDC4	sp|Q8TBB	1	1	1	1	3.1	57.891	0	0	52294000	0	0	0
sp|Q8TBC	UBA3	sp|Q8TBC	1	12	12	12	43	51.852	533750000	493360000	507020000	456540000	532150000	546810000
sp|Q8TBQ	TMEM167	sp|Q8TBQ	1	1	1	1	13	8.0598	0	0	0	0	0	302620000
sp|Q8TBX	PIP4K2C	sp|Q8TBX	1	1	1	1	5.5	47.299	0	0	0	0	0	72501000
sp|Q8TC0	TBC1D15	sp|Q8TC0	1	7	7	7	11	79.49	0	317060000	0	0	0	0
sp|Q8TC1	RDH11	sp|Q8TC1	1	6	6	6	26	35.386	793020000	372070000	556080000	723160000	820060000	956420000
sp|Q8TCB	IFI44	sp|Q8TCB	1	1	1	1	2.7	50.49	0	0	0	0	0	0
sp|Q8TCD	NT5C	sp|Q8TCD	1	3	3	3	25	23.382	0	0	0	0	89475000	0
sp|Q8TCF	ZFAND1	sp|Q8TCF	1	1	1	1	5.6	30.787	0	0	0	0	0	27171000
sp|Q8TCJ	STT3B	sp|Q8TCJ	1	11	11	11	15	93.673	1422200000	1492100000	1444800000	1514200000	1262300000	1273100000
sp|Q8TCS	PNPT1	sp|Q8TCS	1	20	20	20	33	85.95	1477700000	1731500000	1272400000	1527400000	1495900000	1484200000
sp|Q8TCT	HM13	sp|Q8TCT	1	7	7	7	21	41.488	1095900000	991930000	711030000	1217800000	1144000000	1100300000
sp|Q8TD0	MAPK15	sp|Q8TD0	1	1	1	1	1.5	59.831	0	0	0	0	0	224750000
sp|Q8TD1	BICD2	sp|Q8TD1	1	4	4	4	6.7	93.532	0	0	0	0	0	90351000
sp|Q8TD3	GPT2	sp|Q8TD3	1	2	2	2	5.9	57.903	0	0	0	0	533980000	0
sp|Q8TDB	DTX3L	sp|Q8TDB	1	1	1	1	2	83.553	0	0	0	0	0	16160000
sp|Q8TDD	DDX54	sp|Q8TDD	1	5	5	5	7.6	98.594	0	89306000	0	74391000	49091000	0
sp|Q8TDN	BRIX1	sp|Q8TDN	1	12	12	12	43	41.401	2478500000	3354400000	2122400000	3187100000	2385500000	2967500000
sp|Q8TDP	RNASEH2	sp|Q8TDP	1	2	2	2	24	17.84	104570000	103210000	0	76020000	93270000	94272000
sp|Q8TDQ	GNPDA2	sp|Q8TDQ	1	6	3	3	36	31.084	125620000	191790000	0	0	0	181610000
sp|Q8TDX	NEK7	sp|Q8TDX	1	10	10	9	38	34.551	828200000	741850000	769590000	907890000	863410000	650720000
sp|Q8TED	UTP15	sp|Q8TED	1	9	9	9	28	58.414	396240000	350550000	448730000	393730000	401030000	373520000
sp|Q8TED	GPX8	sp|Q8TED	1	6	6	6	33	23.881	718760000	528450000	601460000	626470000	553630000	719710000
sp|Q8TEM	NUP210	sp|Q8TEM	1	15	15	15	14	205.11	877230000	724310000	1002800000	1055500000	913440000	1046300000
sp|Q8TEQ	GEMIN5	sp|Q8TEQ	1	25	25	25	23	168.59	1387400000	1548100000	1338400000	1232100000	1421100000	1331000000
sp|Q9Y4G	RAPGEF2	sp|Q9Y4G	2	1	1	1	0.9	167.41	0	0	0	0	0	30393000
sp|Q8TEX	IPO4	sp|Q8TEX	1	21	21	21	26	118.71	1205700000	1316000000	1269300000	1234800000	1493000000	1213200000
sp|Q8TF6	ZNF384	sp|Q8TF6	1	3	3	3	12	63.218	0	74171000	0	0	0	0
sp|Q8WTS	ABHD5	sp|Q8WTS	1	1	1	1	5.7	39.095	0	0	0	0	10188000	0
sp|Q8WTS	SETD7	sp|Q8WTS	1	4	4	4	14	40.72	206790000	0	217050000	0	258460000	148930000
sp|Q8WTT	NOC3L	sp|Q8WTT	1	8	8	8	13	92.547	326470000	420660000	504090000	372560000	300920000	252740000
sp|Q8WTV	SCARB1	sp|Q8WTV	1	4	4	4	7.6	60.877	310430000	278200000	396040000	321500000	0	502900000
sp|Q8WTV	COG1	sp|Q8WTV	1	3	3	3	6	108.98	0	0	0	22939000	21218000	0
sp|Q8WU1	PYROXD1	sp|Q8WU1	1	2	2	2	10	55.792	0	0	0	0	0	21796000
sp|Q8WU7	SMAP2	sp|Q8WU7	1	2	2	2	6.5	46.785	0	0	0	0	30062000	0

sp|Q8WU	ZC3H15	sp|Q8WU	1	14	14	14	37	48.602	1429400000	1513500000	1400700000	1335400000	1451100000	1400400000
sp|Q8WU	PPIL4	sp|Q8WU	1	2	2	2	6.3	57.224	0	0	0	131440000	0	0
sp|Q8WU	PHF10	sp|Q8WU	1	1	1	1	3.6	56.05	0	0	0	0	34048000	0
sp|Q8WU	PPP1R13	sp|Q8WU	1	8	8	8	16	89.09	351390000	475670000	420530000	276240000	374130000	407690000
sp|Q8WU	TMEM263	sp|Q8WU	1	1	1	1	13	11.748	0	0	0	0	0	60642000
sp|Q8WU	PTPMT1	sp|Q8WU	1	3	3	3	21	22.843	60024000	0	52603000	68726000	47338000	0
sp|Q8WU	NUP133	sp|Q8WU	1	20	20	20	25	128.98	998440000	1199700000	986980000	1040900000	1032400000	1039500000
sp|Q8WU	PDCD6IP	sp|Q8WU	1	39	39	39	57	96.022	8288000000	8366800000	8068500000	7357800000	7657000000	8386700000
sp|Q8WU	SLC38A5	sp|Q8WU	1	5	5	5	12	51.457	299760000	278880000	186740000	0	0	0
sp|Q8WU	CHAC2	sp|Q8WU	1	2	2	2	19	20.874	0	0	165560000	0	0	0
sp|Q8WU	CHMP7	sp|Q8WU	1	2	2	2	7.5	50.91	112040000	165580000	88023000	122400000	123550000	134160000
sp|Q8WU	THEM6	sp|Q8WU	1	4	4	4	28	23.865	0	66467000	0	0	0	0
sp|Q8WU	NAT14	sp|Q8WU	1	1	1	1	11	21.65	0	0	0	0	0	22813000
sp|Q8WV	NSMCE1	sp|Q8WV	1	1	1	1	5.3	30.855	0	0	0	0	0	15702000
sp|Q8WV	LEO1	sp|Q8WV	1	2	2	2	2.6	75.403	81446000	42351000	0	126710000	81449000	85631000
sp|Q8WV	SMIM4	sp|Q8WV	1	1	1	1	16	8.6961	0	0	0	0	51969000	0
sp|Q8WV	NUDCD2	sp|Q8WV	1	4	4	4	46	17.676	185330000	222610000	212480000	224590000	251870000	254280000
sp|Q8WV	TFB1M	sp|Q8WV	1	2	2	2	10	39.542	92926000	120470000	73153000	0	0	81398000
sp|Q8WV	SCFD1	sp|Q8WV	1	11	11	11	23	72.379	603080000	679350000	729450000	581150000	679110000	628990000
sp|Q8WV	CANT1	sp|Q8WV	1	1	1	1	4.5	44.839	0	0	0	0	0	40731000
sp|Q8WV	HNRNPLL	sp|Q8WV	1	12	11	11	32	60.082	581460000	490400000	454700000	587500000	647790000	551450000
sp|Q8WV	FAR1	sp|Q8WV	1	3	3	3	7.4	59.356	0	0	0	0	0	167640000
sp|Q8WV	UBLCP1	sp|Q8WV	1	3	3	3	8.5	36.804	239960000	288500000	264490000	203160000	170120000	308680000
sp|Q8WW	TSEN15	sp|Q8WW	1	2	2	2	20	18.641	76423000	84037000	107890000	0	102360000	95562000
sp|Q8WW	PCNP	sp|Q8WW	1	5	5	5	36	18.925	1039900000	863080000	1290600000	1149200000	1389500000	1675600000
sp|Q8WW	DNAJA4	sp|Q8WW	1	2	2	2	4.8	44.797	0	0	0	403430000	0	0
sp|Q8WW	SPRYD4	sp|Q8WW	1	4	4	4	32	23.128	122160000	159770000	0	0	241540000	0
sp|Q8WW	GLMP	sp|Q8WW	1	1	1	1	6.4	43.864	0	0	0	0	0	50387000
sp|Q8WW	MAIP1	sp|Q8WW	1	3	3	3	11	32.544	245090000	240610000	251620000	214030000	210280000	232360000
sp|Q8WW	LMO7	sp|Q8WW	1	28	28	28	22	192.69	2662800000	2595900000	2392100000	1883900000	2319500000	2175700000
sp|Q8WW	SLC44A1	sp|Q8WW	1	2	2	2	4.4	73.301	0	48661000	66073000	0	0	0
sp|Q8WW	CKAP2	sp|Q8WW	1	3	3	3	5.6	76.986	121880000	112200000	61795000	126390000	128170000	133330000
sp|Q8WW	ATXN2L	sp|Q8WW	1	22	22	22	31	113.37	1798100000	1831200000	1993700000	2073600000	2007900000	2105600000
sp|Q8WW	PHIP	sp|Q8WW	1	4	4	4	4.3	206.69	74860000	0	0	0	0	0
sp|Q8WW	PRPF31	sp|Q8WW	1	8	8	8	28	55.455	503250000	487930000	554230000	411400000	376200000	532690000
sp|Q8WX	NELFB	sp|Q8WX	1	8	8	8	20	65.697	275750000	310680000	354620000	378500000	375100000	328790000
sp|Q8WX	PALLD	sp|Q8WX	2	28	28	28	30	150.56	3972700000	3770000000	3513700000	3202200000	3558100000	3562800000
sp|Q8WX	METTL21	sp|Q8WX	1	1	1	1	6.9	24.6	0	0	0	5872200	0	0
sp|Q8WX	ATRIP	sp|Q8WX	1	1	1	1	1.4	85.837	0	0	0	0	0	167530000

sp	Q8WXE	STON2	sp	Q8WXE	1	1	1	1	1	101.16	0	0	0	0	0	82981000
sp	Q8WXF	PSPC1	sp	Q8WXF	1	10	9	9	23	58.743	648990000	709740000	665200000	720640000	805100000	763490000
sp	Q8WXI	GATAD2B	sp	Q8WXI	1	12	12	10	28	65.26	755580000	652890000	641290000	549890000	591720000	749680000
sp	Q8WXX	DNAJC9	sp	Q8WXX	1	11	11	11	49	29.909	862190000	737730000	953640000	895370000	917720000	986820000
sp	Q8WY2	BRI3BP	sp	Q8WY2	1	2	2	2	14	27.835	250170000	245640000	216130000	224050000	180560000	332540000
sp	Q8WYA	CTNNBL1	sp	Q8WYA	1	5	5	5	9.1	65.173	166220000	214370000	192200000	148410000	169400000	169930000
sp	Q8WYP	AHCTF1	sp	Q8WYP	1	16	16	16	11	252.5	640190000	654620000	631480000	529500000	578170000	920210000
sp	Q8WYQ	ZCCHC14	sp	Q8WYQ	1	1	1	1	0.9	100.04	0	58622000	0	0	0	0
sp	Q8WZ8	OVCA2	sp	Q8WZ8	1	4	4	4	23	24.418	168100000	136310000	160000000	126510000	116430000	109910000
sp	Q8WZA	LZIC	sp	Q8WZA	1	5	5	5	37	21.494	292950000	310470000	286960000	310210000	335550000	155740000
sp	Q9246	DDB2	sp	Q9246	1	8	8	8	24	47.863	396190000	396760000	593490000	343750000	332320000	362510000
sp	Q9249	DDX1	sp	Q9249	1	31	31	31	49	82.431	5851100000	5893000000	5393500000	5559700000	5427500000	5114700000
sp	Q9250	SLC39A7	sp	Q9250	1	2	2	2	3	50.117	0	0	85205000	0	0	0
sp	Q9250	HSD17B8	sp	Q9250	1	1	1	1	5	26.973	0	0	0	0	0	30998000
sp	Q9252	FAM3C	sp	Q9252	1	2	2	2	12	24.68	249690000	184910000	0	0	0	0
sp	Q9252	H1FX	sp	Q9252	1	5	5	5	31	22.487	983670000	930920000	869160000	1069800000	1178400000	1334400000
sp	Q9253	PSMF1	sp	Q9253	1	2	2	2	12	29.816	176300000	236340000	173580000	183090000	228230000	202520000
sp	Q9253	SLC7A6	sp	Q9253	1	3	2	2	11	56.827	0	0	0	0	0	471530000
sp	Q9253	GBF1	sp	Q9253	1	16	16	16	12	206.44	694540000	669040000	650650000	616850000	715910000	636740000
sp	Q9254	RTF1	sp	Q9254	1	7	7	7	11	80.313	0	0	0	224910000	0	0
sp	Q9254	NCSTN	sp	Q9254	1	4	4	4	6.1	78.41	267580000	264520000	275540000	256240000	307100000	237590000
sp	Q9254	TM9SF4	sp	Q9254	1	7	7	7	18	74.518	472030000	456960000	369310000	438130000	334690000	427370000
sp	Q9255	MRPS27	sp	Q9255	1	8	8	8	22	47.611	555750000	610190000	462080000	398640000	476920000	458700000
sp	Q9257	AP3S1	sp	Q9257	1	4	4	4	28	21.732	226770000	122920000	259070000	202010000	281690000	222720000
sp	Q9257	TSC1	sp	Q9257	1	1	1	1	1.4	129.77	0	0	0	0	0	17428000
sp	Q9257	UBXN4	sp	Q9257	1	3	3	3	14	56.777	280610000	250650000	217070000	297320000	288960000	312080000
sp	Q9259	NDRG1	sp	Q9259	1	4	4	4	20	42.835	332460000	198060000	262980000	318920000	329600000	253670000
sp	Q9259	HSPH1	sp	Q9259	1	40	39	37	57	96.864	9558800000	1.0267E+10	9690700000	10504000000	10207000000	1.1486E+10
sp	Q9260	CNOT9	sp	Q9260	1	6	6	6	25	33.631	409260000	327120000	455840000	246190000	0	0
sp	Q9260	LPGAT1	sp	Q9260	1	3	3	3	12	43.089	0	0	0	0	0	109010000
sp	Q9261	MYO18A	sp	Q9261	1	2	2	2	1.2	233.11	0	0	0	0	0	36100000
sp	Q9261	LARP4B	sp	Q9261	1	9	9	9	18	80.551	320630000	464830000	476660000	582190000	442560000	282460000
sp	Q9261	GCN1	sp	Q9261	1	72	72	72	34	292.75	5023100000	4793700000	4675600000	4747600000	4494400000	4731600000
sp	Q9262	DHX38	sp	Q9262	1	8	8	8	9.1	140.5	230250000	248960000	250190000	176340000	305130000	252740000
sp	Q9262	NUP205	sp	Q9262	1	26	26	26	16	227.92	1502100000	1585200000	1503100000	1712800000	1668600000	1592300000
sp	Q9262	PXDN	sp	Q9262	1	5	5	5	5.9	165.27	192430000	185110000	208160000	228710000	215640000	212910000
sp	Q9264	PIGK	sp	Q9264	1	3	3	3	12	45.251	103400000	0	118970000	184520000	89609000	47764000
sp	Q9266	MRPS31	sp	Q9266	1	7	7	7	26	45.318	318130000	280120000	319290000	303420000	419420000	412920000
sp	Q9266	AKAP1	sp	Q9266	1	4	4	4	7.1	97.34	145760000	122570000	100220000	122440000	135700000	129410000

sp	Q92685	ALG3	sp	Q92685	1	1	1	1	2.3	50.126	0	0	0	0	0	48592000
sp	Q92688	ANP32B	sp	Q92688	1	9	7	7	32	28.787	2287200000	3357700000	2691300000	2683500000	3018000000	2667000000
sp	Q92692	NECTIN2	sp	Q92692	1	1	1	1	3.5	57.741	0	0	0	0	0	31372000
sp	Q92696	RABGGTA	sp	Q92696	1	7	7	7	15	65.071	201400000	163140000	162070000	219440000	272480000	221480000
sp	Q92733	PRCC	sp	Q92733	1	5	5	5	15	52.417	0	137070000	204390000	127510000	176530000	144090000
sp	Q92734	TFG	sp	Q92734	1	10	10	10	35	43.447	1913300000	1631100000	1705800000	1367300000	1349100000	1404400000
sp	Q92747	ARPC1A	sp	Q92747	1	10	9	9	39	41.569	1014000000	1041800000	866990000	917080000	942850000	1085700000
sp	Q92759	GTF2H4	sp	Q92759	1	1	1	1	2.8	52.186	0	0	0	0	0	66459000
sp	Q92769	HDAC2	sp	Q92769	1	10	10	7	30	55.364	2661400000	2928800000	3173200000	2296600000	2584200000	2654300000
sp	Q92783	STAM	sp	Q92783	1	3	3	3	8.7	59.179	0	90835000	146640000	0	0	0
sp	Q92785	DPF2	sp	Q92785	1	5	5	5	16	44.155	258970000	243940000	325010000	193920000	211120000	227940000
sp	Q92791	P3H4	sp	Q92791	1	3	3	3	9.2	50.381	0	104120000	136650000	0	71859000	0
sp	Q92793	CREBBP	sp	Q92793	2	4	4	4	2.8	265.35	126640000	0	0	165530000	239900000	139330000
sp	Q92797	SYMPK	sp	Q92797	1	8	8	8	12	141.15	279250000	357250000	339930000	331540000	388670000	318030000
sp	Q92804	TAF15	sp	Q92804	1	7	5	5	21	61.829	834050000	1079100000	1023200000	783060000	879440000	755430000
sp	Q92820	GGH	sp	Q92820	1	2	2	2	8.2	35.964	65319000	84785000	70487000	83718000	79004000	94415000
sp	Q92841	DDX17	sp	Q92841	1	26	17	17	41	80.272	5089700000	6012900000	5593700000	5124800000	4899300000	4752000000
sp	Q92878	RAD50	sp	Q92878	1	24	24	24	23	153.89	1113200000	1214700000	1028000000	1242600000	1305900000	1352800000
sp	Q92879	CELF1	sp	Q92879	2	7	7	7	15	52.063	728710000	792790000	555290000	588130000	833790000	789090000
sp	Q92882	OSTF1	sp	Q92882	1	4	4	4	24	23.787	0	0	0	0	0	206570000
sp	Q92888	ARHGEF1	sp	Q92888	1	3	3	3	4.4	102.43	0	148190000	142740000	0	0	104670000
sp	Q92889	ERCC4	sp	Q92889	1	2	2	2	4.8	104.48	101860000	0	0	0	0	211260000
sp	Q92890	UFD1	sp	Q92890	1	7	7	7	33	34.5	730370000	694030000	620100000	598170000	698430000	414280000
sp	Q92896	GLG1	sp	Q92896	1	12	12	12	16	134.55	489010000	755540000	631020000	594840000	562510000	543020000
sp	Q92900	UPF1	sp	Q92900	1	27	27	27	31	124.34	1979000000	1873700000	2293700000	2135200000	2218800000	1981400000
sp	Q92905	COPS5	sp	Q92905	1	12	12	12	42	37.578	804760000	909510000	922980000	688630000	686740000	784010000
sp	Q92917	GPKOW	sp	Q92917	1	8	8	8	27	52.228	483670000	366510000	442880000	502820000	504830000	345680000
sp	Q92922	SMARCC1	sp	Q92922	1	16	16	10	21	122.87	2442200000	2014000000	2057700000	1997000000	1660700000	2486900000
sp	Q92934	BAD	sp	Q92934	1	2	2	2	16	18.392	0	0	0	0	0	26945000
sp	Q92945	KHSRP	sp	Q92945	1	22	20	20	40	73.114	5167900000	4796100000	5590000000	4979600000	5517400000	4585900000
sp	Q92947	GCDH	sp	Q92947	1	5	5	5	27	48.127	149940000	0	0	0	148010000	134510000
sp	Q92973	TNPO1	sp	Q92973	1	20	20	17	33	102.35	6971700000	6621600000	7118400000	7047000000	6868100000	6249100000
sp	Q92974	ARHGEF2	sp	Q92974	1	10	10	10	15	111.54	636060000	557580000	443910000	550580000	527240000	506450000
sp	Q92979	EMG1	sp	Q92979	1	9	9	9	52	26.72	996490000	887480000	830350000	977720000	863480000	1128300000
sp	Q92990	GLMN	sp	Q92990	1	7	7	7	15	68.207	250760000	358450000	314060000	288150000	308770000	254130000
sp	Q92995	USP13	sp	Q92995	1	3	3	3	8.2	97.326	115910000	0	0	88804000	103860000	95652000
sp	Q93008	USP9X	sp	Q93008	1	25	25	15	13	292.28	1058000000	1064000000	1098100000	964160000	847080000	957790000
sp	Q93009	USP7	sp	Q93009	1	18	18	18	25	128.3	924280000	983170000	1090900000	862370000	816590000	1184400000
sp	Q93045	STMN2	sp	Q93045	1	2	1	1	11	20.828	0	0	0	0	0	1381900000

sp|Q9305(	ATP6V0A	sp|Q9305(	2	7	7	7	10	96.412	0	287240000	307460000	288960000	260320000	238490000
sp|Q9305	LPP	sp|Q9305	1	15	15	15	37	65.746	933600000	860230000	959870000	789630000	857440000	931150000
sp|Q9307	MED12	sp|Q9307	1	1	1	1	1.4	243.08	0	0	0	0	18164000	0
sp|Q9309	PTP4A1	sp|Q9309	1	6	6	3	39	19.815	363450000	481460000	354250000	616950000	601830000	673670000
sp|Q969E	SCAMP4	sp|Q969E	1	1	1	1	4.8	25.728	0	0	0	0	108270000	0
sp|Q969E	TSR2	sp|Q969E	1	1	1	1	4.7	20.894	0	0	0	0	0	108320000
sp|Q969G	SMARCE1	sp|Q969G	1	8	8	8	29	46.649	449820000	589870000	672150000	531690000	510750000	578990000
sp|Q969G	CAVIN3	sp|Q969G	1	2	2	2	10	27.701	0	0	0	0	351660000	0
sp|Q969H	MYDGF	sp|Q969H	1	5	5	5	37	18.795	2110300000	1709400000	3236800000	2307600000	2432800000	2708400000
sp|Q969J	BORCS5	sp|Q969J	1	2	2	2	13	22.222	0	0	0	0	43485000	0
sp|Q969M	YIPF5	sp|Q969M	1	1	1	1	4.7	27.989	0	0	0	0	0	140250000
sp|Q969N	PIGT	sp|Q969N	1	2	2	2	5	65.699	0	0	0	0	269620000	0
sp|Q969Q	RPL36AL	sp|Q969Q	1	4	4	1	32	12.469	4420000000	5402200000	4812600000	4663700000	4402000000	4472600000
sp|Q969S	ZNF622	sp|Q969S	1	6	6	6	17	54.271	358080000	240550000	306690000	321390000	273250000	227680000
sp|Q969S	GFM2	sp|Q969S	1	3	3	3	5.4	86.6	141710000	99803000	81861000	88744000	106480000	113240000
sp|Q969T	WBP2	sp|Q969T	1	1	1	1	3.8	28.087	0	0	0	0	88114000	0
sp|Q969U	PSMG2	sp|Q969U	1	7	7	7	26	29.396	367920000	141920000	284820000	207010000	252490000	265270000
sp|Q969V	NCLN	sp|Q969V	1	11	11	11	23	62.974	738130000	805930000	681810000	829080000	735580000	694900000
sp|Q969V	MKL1	sp|Q969V	1	1	1	1	3.1	98.918	0	0	0	0	0	11371000
sp|Q969X	TMBIM1	sp|Q969X	1	1	1	1	4.5	34.607	0	0	0	0	0	37363000
sp|Q969X	ERGIC1	sp|Q969X	1	6	6	6	27	32.592	1338000000	1368800000	1016300000	1067600000	1052700000	1613600000
sp|Q969X	UTP4	sp|Q969X	1	6	6	6	14	76.889	329060000	425540000	380700000	407440000	283720000	294000000
sp|Q969Y	GTPBP3	sp|Q969Y	1	1	1	1	4.7	52.058	16481000	23143000	8561500	17623000	27722000	27122000
sp|Q969Z	TBRG4	sp|Q969Z	1	9	9	9	19	70.737	663220000	789430000	765200000	611910000	876410000	1093900000
sp|Q96A2	FAM162A	sp|Q96A2	1	4	4	4	30	17.342	786330000	231050000	898950000	549240000	822660000	1019500000
sp|Q96A3	CCDC47	sp|Q96A3	1	17	17	17	37	55.873	1294000000	1447800000	1347700000	1636500000	1544300000	1971700000
sp|Q96A3	MRPL24	sp|Q96A3	1	3	3	3	16	24.915	0	0	0	0	0	81780000
sp|Q96A4	SYAP1	sp|Q96A4	1	9	9	9	30	39.933	687610000	456540000	560780000	433350000	505170000	501600000
sp|Q96A6	EXOC4	sp|Q96A6	1	9	9	9	14	110.5	296380000	292470000	290980000	244300000	201860000	230540000
sp|Q96A7	MAGOHB	sp|Q96A7	1	4	4	1	31	17.276	1249800000	1486000000	1587000000	1336400000	1598900000	1171900000
sp|Q96AB	ISOC2	sp|Q96AB	1	2	2	2	24	22.337	107280000	75088000	72565000	0	0	119220000
sp|Q96AC	FERMT2	sp|Q96AC	1	31	31	29	53	77.86	5613900000	6394900000	5526300000	5506500000	6017400000	5062100000
sp|Q96AE	FUBP1	sp|Q96AE	1	23	23	21	47	67.56	9296600000	8325400000	7712600000	7773800000	9684700000	9379700000
sp|Q96AG	LRRC59	sp|Q96AG	1	12	12	12	45	34.93	8670000000	8449300000	7354000000	9119800000	8869300000	8628900000
sp|Q96AQ	PBXIP1	sp|Q96AQ	1	3	3	3	6.8	80.642	0	0	90974000	0	0	0
sp|Q96AT	KIAA1143	sp|Q96AT	1	2	2	2	16	17.465	0	103400000	86311000	0	0	0
sp|Q96AT	RPE	sp|Q96AT	2	4	4	4	24	24.927	796440000	776610000	994680000	651710000	850540000	815450000
sp|Q96AY	FKBP10	sp|Q96AY	1	13	13	13	27	64.244	1812700000	1302700000	1251200000	1433700000	1394200000	1436800000
sp|Q96B0	RAD51AP	sp|Q96B0	1	1	1	1	4.3	38.457	0	0	0	0	0	0

sp|Q96B2	EXOSC8	sp|Q96B2	1	5	5	5	30	30.039	211290000	265930000	220370000	265230000	247650000	238370000
sp|Q96B3	AKT1S1	sp|Q96B3	1	4	4	4	13	27.383	0	0	255560000	0	0	0
sp|Q96B4	TOMM6	sp|Q96B4	1	2	2	2	57	8.0019	710200000	609630000	533900000	789620000	1271200000	851390000
sp|Q96B5	ZNF428	sp|Q96B5	1	4	4	4	30	20.48	185330000	225520000	131200000	179520000	209040000	127210000
sp|Q96B9	SH3KBP1	sp|Q96B9	1	3	3	3	6.8	73.125	74507000	117460000	152490000	125170000	114490000	0
sp|Q96BH	RNF25	sp|Q96BH	1	2	2	2	9.2	51.218	0	0	0	55397000	0	0
sp|Q96BN	OTULIN	sp|Q96BN	1	10	10	10	36	40.262	612610000	476570000	411580000	649900000	536330000	605360000
sp|Q96BP	PPWD1	sp|Q96BP	1	7	7	7	18	73.574	234690000	177160000	150250000	156600000	225790000	134060000
sp|Q96BR	COA7	sp|Q96BR	1	4	4	4	30	25.709	243470000	147230000	313610000	0	183160000	154430000
sp|Q96BW	TAMM41	sp|Q96BW	1	1	1	1	4.4	51.066	0	0	0	0	0	55289000
sp|Q96BY	DOCK10	sp|Q96BY	1	1	1	1	0.7	249.53	0	0	0	0	0	5328100
sp|Q96C0	FAM136A	sp|Q96C0	1	1	1	1	10	15.641	0	0	26457000	0	0	0
sp|Q96C1	EFHD2	sp|Q96C1	2	5	5	5	23	26.697	341510000	231940000	223890000	285680000	275740000	235930000
sp|Q96C2	GALM	sp|Q96C2	1	2	2	2	6.7	37.765	0	0	0	0	91778000	0
sp|Q96C3	PYCR2	sp|Q96C3	1	10	9	9	40	33.637	464810000	381360000	504620000	540460000	442060000	461530000
sp|Q96C5	CUSTOS	sp|Q96C5	1	1	1	1	8	28.17	0	0	0	0	0	0
sp|Q96C8	DCPS	sp|Q96C8	1	6	6	6	24	38.608	214770000	226060000	173590000	226930000	208620000	208530000
sp|Q96C9	PPP1R14	sp|Q96C9	1	4	4	4	48	15.911	1286800000	1101800000	1082300000	888870000	737110000	802670000
sp|Q96CB	INTS12	sp|Q96CB	1	1	1	1	3	48.807	0	0	0	0	0	0
sp|Q96CN	ISOC1	sp|Q96CN	1	7	7	7	32	32.236	426350000	595390000	489400000	422150000	361270000	388970000
sp|Q96CN	GCC1	sp|Q96CN	1	1	1	1	1.3	87.81	0	0	0	0	0	491920000
sp|Q96CP	FLYWCH2	sp|Q96CP	2	4	4	4	46	14.563	142030000	0	154100000	110120000	165080000	0
sp|Q96CS	FAF2	sp|Q96CS	1	9	9	9	34	52.623	1105700000	1303800000	1305300000	1293300000	1242200000	1233300000
sp|Q96CT	CCDC124	sp|Q96CT	1	7	7	7	34	25.835	469600000	459740000	559180000	458560000	519830000	609720000
sp|Q96CU	FOXRED1	sp|Q96CU	1	2	2	2	6.6	53.811	0	0	0	0	47262000	0
sp|Q96CV	OPTN	sp|Q96CV	1	1	1	1	2.3	65.921	0	0	0	0	0	12537000
sp|Q96CW	AP2M1	sp|Q96CW	1	5	5	5	12	49.654	321510000	326420000	332180000	341390000	310170000	0
sp|Q96CW	TUBGCP3	sp|Q96CW	1	4	4	4	6.6	103.57	69851000	0	124710000	0	0	57874000
sp|Q96CX	KCTD12	sp|Q96CX	1	7	7	7	37	35.7	503090000	458580000	490930000	315680000	451510000	474400000
sp|Q96D4	NMD3	sp|Q96D4	1	8	8	8	22	57.603	419940000	610990000	413360000	460630000	555420000	473730000
sp|Q96DB	RMDN1	sp|Q96DB	1	2	2	2	7.3	35.808	139330000	126300000	0	92409000	0	0
sp|Q96DE	NUDT16	sp|Q96DE	1	3	3	2	27	21.273	21315000	72806000	49906000	51976000	52685000	29534000
sp|Q96DG	CMBL	sp|Q96DG	1	8	8	8	37	28.048	1701600000	1635500000	1573300000	1297700000	1445600000	1616200000
sp|Q96DH	MSI2	sp|Q96DH	2	4	4	4	17	35.196	0	252980000	0	0	206480000	274780000
sp|Q96DI7	SNRNP40	sp|Q96DI7	1	12	12	12	46	39.31	1668100000	1815300000	1599300000	1528300000	1637000000	1268000000
sp|Q96DV	MRPL38	sp|Q96DV	1	7	7	7	24	44.596	546690000	403980000	351350000	447710000	391620000	364960000
sp|Q96DZ	ERLEC1	sp|Q96DZ	1	2	2	2	6.4	54.858	0	0	0	0	0	22853000
sp|Q96E1	MRRF	sp|Q96E1	1	7	7	7	35	29.277	849810000	1088600000	767860000	896240000	988420000	966230000
sp|Q96E2	MTERF3	sp|Q96E2	1	1	1	1	3.1	47.971	0	0	0	0	43760000	0

sp|Q96EA	SPDL1	sp|Q96EA	1	4	4	4	9.6	70.171	140110000	127690000	116960000	117310000	125940000	0
sp|Q96EB	ELP4	sp|Q96EB	1	3	3	3	12	46.587	0	0	0	0	31230000	0
sp|Q96EC	YIPF6	sp|Q96EC	1	1	1	1	5.1	26.256	0	0	0	0	0	37400000
sp|Q96EE	SEH1L	sp|Q96EE	1	11	11	11	49	39.648	1261800000	1313800000	1252500000	1465100000	1246700000	1370900000
sp|Q96EI5	TCEAL4	sp|Q96EI5	1	3	3	3	21	24.647	163640000	141500000	0	133540000	140480000	118140000
sp|Q96EK	KIF1BP	sp|Q96EK	1	8	8	8	16	71.813	404390000	317710000	256980000	327900000	340800000	442440000
sp|Q96EK	GNPNAT1	sp|Q96EK	1	7	7	7	59	20.749	1427800000	1185600000	1308600000	1239300000	1679000000	1221000000
sp|Q96EL	MRPL53	sp|Q96EL	1	4	4	4	49	12.107	0	291540000	478730000	425780000	407630000	0
sp|Q96EM	L3HYPDH	sp|Q96EM	1	2	2	2	7.3	38.137	0	0	0	0	0	26914000
sp|Q96EP	DAZAP1	sp|Q96EP	1	5	5	5	19	43.383	1766100000	1292400000	1774600000	1611500000	1424000000	1915000000
sp|Q96EQ	SGTB	sp|Q96EQ	1	1	1	1	7.9	33.429	0	0	0	0	0	16306000
sp|Q96ER	SAAL1	sp|Q96ER	1	1	1	1	2.1	53.557	0	0	0	0	0	39830000
sp|Q96ER	CCDC51	sp|Q96ER	1	8	8	8	24	45.811	409090000	397540000	315480000	301010000	354140000	392770000
sp|Q96EU	RRP36	sp|Q96EU	1	1	1	1	4.2	29.823	0	0	27704000	0	0	0
sp|Q96EX	SMIM12	sp|Q96EX	1	1	1	1	12	10.799	0	0	95581000	0	0	0
sp|Q96EY	DNAJA3	sp|Q96EY	1	8	8	8	24	52.488	581860000	843870000	684120000	899530000	965600000	927590000
sp|Q96EY	TMA16	sp|Q96EY	1	2	2	2	17	23.864	0	206020000	0	0	0	0
sp|Q96EY	MVB12A	sp|Q96EY	1	1	1	1	9.5	28.783	0	0	0	0	0	7626200
sp|Q96EY	PTCD3	sp|Q96EY	1	3	3	3	6	78.549	105160000	94282000	160160000	141960000	167520000	146860000
sp|Q96EY	MMAB	sp|Q96EY	1	2	2	2	12	27.388	74710000	71748000	82952000	0	109870000	0
sp|Q96EZ	MCRS1	sp|Q96EZ	1	1	1	1	3.9	51.803	0	0	0	0	0	119860000
sp|Q96F8	EDC3	sp|Q96F8	1	6	6	6	17	56.077	148320000	239750000	142210000	294770000	186190000	182420000
sp|Q96FJ	STAMBPL	sp|Q96FJ	1	2	2	2	8.5	49.783	0	107190000	0	125430000	0	0
sp|Q96FJ	DYNLL2	sp|Q96FJ	1	4	2	2	57	10.35	119080000	130280000	121070000	139200000	141430000	162490000
sp|Q96FQ	S100A16	sp|Q96FQ	1	5	5	5	53	11.801	2612600000	2556000000	2421900000	2123400000	2614300000	2358200000
sp|Q96FV	SCRN2	sp|Q96FV	1	2	2	2	11	46.596	293100000	0	0	0	246340000	0
sp|Q96FV	THOC1	sp|Q96FV	1	9	9	9	15	75.665	286060000	256520000	286030000	249780000	264460000	310230000
sp|Q96FW	OTUB1	sp|Q96FW	1	10	10	10	49	31.284	2338100000	2524100000	2641700000	2516900000	2497400000	2296400000
sp|Q96FX	TRMT61A	sp|Q96FX	1	2	2	2	9.7	31.381	81893000	77832000	69329000	72437000	81791000	76341000
sp|Q96FX	PERP	sp|Q96FX	1	1	1	1	8.8	21.386	0	0	0	16244000	0	0
sp|Q96FZ	CHMP6	sp|Q96FZ	1	1	1	1	6.5	23.485	0	0	0	0	0	42222000
sp|Q96G0	PGM2	sp|Q96G0	1	8	8	8	18	68.283	390830000	342980000	335090000	352470000	310590000	331070000
sp|Q96G2	IMP4	sp|Q96G2	1	3	3	3	11	33.756	115790000	98055000	91924000	100210000	117510000	0
sp|Q96G2	CERS2	sp|Q96G2	1	4	4	4	14	44.876	568610000	1124200000	897230000	871700000	907830000	796540000
sp|Q96G4	DUS3L	sp|Q96G4	1	6	6	6	15	72.593	141180000	213590000	161550000	174520000	191270000	182610000
sp|Q96GA	LTV1	sp|Q96GA	1	1	1	1	4	54.854	0	0	0	51389000	0	0
sp|Q96GC	MRPL48	sp|Q96GC	1	4	4	4	22	23.934	0	300840000	245180000	228090000	296200000	282230000
sp|Q96GC	VMP1	sp|Q96GC	1	2	2	2	9.1	46.237	0	0	91920000	199730000	188320000	0
sp|Q96GD	PDXP	sp|Q96GD	1	2	2	2	13	31.698	113490000	0	0	0	0	0

sp|Q96GD	AURKB	sp|Q96GD	2	5	5	5	22	39.31	107790000	99238000	127940000	109770000	103000000	122860000
sp|Q96GE	DMAC1	sp|Q96GE	1	2	2	2	33	12.257	0	0	147250000	0	0	0
sp|Q96GG	DCUN1D1	sp|Q96GG	1	4	4	4	21	30.124	317960000	313590000	0	0	382510000	252660000
sp|Q96GM	SMARCD1	sp|Q96GM	3	5	5	5	13	58.232	203190000	177610000	215600000	196260000	199520000	293420000
sp|Q96GM	TOE1	sp|Q96GM	1	5	5	5	18	56.547	278460000	0	214220000	222710000	265210000	0
sp|Q96GQ	DDX27	sp|Q96GQ	1	15	15	15	21	89.834	1084800000	1030100000	1088300000	1169200000	949670000	1082100000
sp|Q96GW	MARS2	sp|Q96GW	1	2	2	2	7.4	66.59	0	0	0	76803000	0	0
sp|Q96GX	APIP	sp|Q96GX	1	1	1	1	7	27.125	0	0	0	0	0	94002000
sp|Q96H2	SNF8	sp|Q96H2	1	1	1	1	4.7	28.864	0	0	0	0	0	84416000
sp|Q96H7	ZC3HAV1	sp|Q96H7	1	3	3	3	15	32.962	197090000	0	0	0	0	0
sp|Q96HC	PDLIM5	sp|Q96HC	1	22	22	22	51	63.944	4031000000	4452500000	3712700000	4915400000	5132600000	4985500000
sp|Q96HE	ERO1A	sp|Q96HE	2	12	12	12	36	54.392	1298000000	1125900000	1114100000	1167400000	1256400000	1148400000
sp|Q96HP	OXNAD1	sp|Q96HP	1	1	1	1	3.8	34.854	0	0	0	0	0	16944000
sp|Q96HQ	CDKN2AI	sp|Q96HQ	1	1	1	1	16	13.196	0	0	0	0	0	13484000
sp|Q96HR	NAF1	sp|Q96HR	1	1	1	1	3	53.716	0	0	0	0	0	104330000
sp|Q96HS	PGAM5	sp|Q96HS	1	12	12	12	50	32.004	966600000	972850000	999430000	796340000	863630000	1138600000
sp|Q96HY	DDRGK1	sp|Q96HY	1	3	3	3	15	35.61	129580000	178740000	168580000	201360000	212850000	174290000
sp|Q96I24	FUBP3	sp|Q96I24	1	21	20	20	52	61.64	2887200000	2968800000	2954500000	2509500000	2933700000	2711400000
sp|Q96I25	RBM17	sp|Q96I25	1	7	7	7	21	44.961	285630000	337640000	337810000	325830000	474620000	305650000
sp|Q96I51	RCC1L	sp|Q96I51	1	2	2	2	6.7	49.996	0	0	0	0	0	110280000
sp|Q96I99	SUCLG2	sp|Q96I99	1	14	14	14	39	46.51	1466400000	1618800000	1573700000	1695200000	1655500000	1623600000
sp|Q96IJ6	GMPPA	sp|Q96IJ6	1	6	6	6	23	46.291	304810000	342680000	254280000	295320000	260770000	228870000
sp|Q96IR7	HPDL	sp|Q96IR7	1	2	2	2	14	39.385	147400000	48558000	60893000	261520000	148640000	155600000
sp|Q96IU4	ABHD14B	sp|Q96IU4	1	5	5	5	33	22.345	421980000	385440000	376590000	481830000	436890000	356780000
sp|Q96IX5	USMG5	sp|Q96IX5	1	3	3	3	45	6.4575	0	2160900000	1806300000	0	0	2263000000
sp|Q96IY1	NSL1	sp|Q96IY1	1	1	1	1	5.3	32.162	0	0	0	0	39689000	0
sp|Q96IZ0	PAWR	sp|Q96IZ0	1	8	8	8	46	36.567	786180000	841680000	830540000	748350000	934640000	718760000
sp|Q96J01	THOC3	sp|Q96J01	1	5	5	5	18	38.771	424110000	459320000	517220000	459040000	435830000	418580000
sp|Q96J02	ITCH	sp|Q96J02	1	3	3	3	4.3	102.8	59894000	0	0	88629000	0	0
sp|Q96J42	TXNDC15	sp|Q96J42	1	1	1	1	5.3	39.885	0	0	0	22057000	0	0
sp|Q96JB	DNAH8	sp|Q96JB	1	4	4	4	1.2	514.66	1698800000	1932300000	922310000	1546600000	1959900000	1834500000
sp|Q96JB	COG3	sp|Q96JB	1	3	3	3	4.7	94.095	22657000	0	48201000	43751000	42297000	61220000
sp|Q96JB	CDK5RAP	sp|Q96JB	1	5	5	5	12	56.92	0	113590000	93419000	104800000	110060000	118270000
sp|Q96JC	VPS39	sp|Q96JC	1	2	2	2	4.2	101.81	0	0	0	0	54259000	0
sp|Q96JG	VPS50	sp|Q96JG	1	5	5	5	7.9	111.17	197770000	273420000	248400000	244000000	203190000	222030000
sp|Q96JH	VCPIP1	sp|Q96JH	1	3	3	3	4.1	134.32	0	0	0	0	0	88298000
sp|Q96JI7	SPG11	sp|Q96JI7	1	2	2	2	2.1	278.86	172500000	0	0	0	0	0
sp|Q96JJ3	ELMO2	sp|Q96JJ3	2	5	5	5	12	82.614	116170000	120830000	0	143480000	171240000	0
sp|Q96JJ7	TMX3	sp|Q96JJ7	1	9	9	9	21	51.871	558610000	476790000	531650000	568630000	643160000	584000000

sp	Q96JM	CHAMP1	sp	Q96JM	1	3	3	3	4.1	89.098	59837000	45807000	63620000	50413000	48063000	37626000
sp	Q96JX	SERAC1	sp	Q96JX	1	1	1	1	1.4	74.147	0	0	0	0	246850000	0
sp	Q96JY	PDLIM2	sp	Q96JY	1	1	1	1	3.1	37.458	0	0	0	0	0	20454000
sp	Q96K1	BTF3L4	sp	Q96K1	1	5	5	5	41	17.27	363500000	467630000	375410000	453560000	478880000	356230000
sp	Q96K1	RNF170	sp	Q96K1	1	1	1	1	5.4	29.814	0	0	0	22407000	0	0
sp	Q96K3	SLC35E1	sp	Q96K3	1	1	1	1	5.9	44.772	0	0	0	0	0	53081000
sp	Q96K7	USP47	sp	Q96K7	1	10	10	10	12	157.31	296390000	268770000	211260000	225890000	211720000	257880000
sp	Q96KA	CLPTM1L	sp	Q96KA	1	9	9	9	24	62.228	979580000	794570000	916160000	1188400000	711970000	946770000
sp	Q96KB	PBK	sp	Q96KB	1	8	8	8	35	36.085	735750000	864580000	703830000	760860000	723430000	693530000
sp	Q96KG	SCYL1	sp	Q96KG	1	5	5	5	7.8	89.63	205480000	194150000	192250000	223530000	189560000	229660000
sp	Q96KP	EXOC2	sp	Q96KP	1	3	3	3	5	104.07	0	0	120440000	0	0	0
sp	Q96KP	CNDP2	sp	Q96KP	1	15	15	15	43	52.878	1785500000	1645900000	1605000000	1723300000	1689800000	1936000000
sp	Q96KQ	EHMT2	sp	Q96KQ	1	2	2	2	3.1	132.37	27990000	0	27841000	43453000	79487000	0
sp	Q96KR	ZFR	sp	Q96KR	1	20	20	20	29	117.01	1.2539E+10	1.0824E+10	1.3084E+10	10140000000	11699000000	1.1214E+10
sp	Q96L9	SNX27	sp	Q96L9	1	3	3	3	6.7	61.264	0	53128000	72294000	77988000	85240000	62620000
sp	Q96LD	TRIM47	sp	Q96LD	1	7	7	7	15	69.531	0	0	0	240010000	235840000	215500000
sp	Q96LJ	DHRS1	sp	Q96LJ	1	3	3	3	11	33.909	63817000	76688000	0	0	44838000	92404000
sp	Q96LR	UBE2E2	sp	Q96LR	1	3	3	2	22	22.255	0	0	0	0	82623000	0
sp	Q96M2	PRRC1	sp	Q96M2	1	6	6	6	17	46.701	455380000	452760000	464020000	553430000	320590000	469910000
sp	Q96ME	FBXL18	sp	Q96ME	1	2	2	2	3	88.34	0	0	0	41800000	0	0
sp	Q96ME	ZNF512	sp	Q96ME	1	2	2	2	9.3	64.681	112660000	97252000	107210000	110480000	117500000	118250000
sp	Q96MG	NSMCE3	sp	Q96MG	1	1	1	1	5.3	34.308	0	0	0	0	0	0
sp	Q96MU	YTHDC1	sp	Q96MU	1	2	2	2	2.5	84.699	0	0	0	0	34476000	0
sp	Q96MW	CCDC43	sp	Q96MW	1	1	1	1	13	25.248	49188000	20128000	66237000	0	0	0
sp	Q96MX	WDR92	sp	Q96MX	1	3	3	3	14	39.74	64088000	88196000	0	68069000	77900000	102770000
sp	Q96N6	MBOAT7	sp	Q96N6	1	5	5	5	15	52.764	745390000	852890000	952290000	828510000	629590000	770540000
sp	Q96N6	DOCK7	sp	Q96N6	1	3	3	3	2.8	242.56	87937000	101180000	0	106350000	110750000	89159000
sp	Q96NC	ZMAT2	sp	Q96NC	1	1	1	1	10	23.612	0	0	0	0	0	109760000
sp	Q96NH	TBC1D32	sp	Q96NH	1	1	1	1	1.4	144.75	0	0	0	0	134720000	0
sp	Q96NU	SAMD11	sp	Q96NU	1	2	2	2	6	72.707	0	76549000	91376000	0	119060000	0
sp	Q96P1	NSUN5	sp	Q96P1	2	2	2	2	5.8	46.691	103830000	141820000	0	94401000	74227000	0
sp	Q96P1	RPRD1A	sp	Q96P1	1	6	6	5	28	35.719	328270000	375430000	334360000	420060000	428690000	446430000
sp	Q96P4	ARAP1	sp	Q96P4	1	2	2	2	1.7	162.19	0	0	0	0	32863000	0
sp	Q96P7	IPO9	sp	Q96P7	1	14	14	14	20	115.96	2057700000	2040300000	2211500000	2361700000	1841700000	1672700000
sp	Q96PC	MIA2	sp	Q96PC	3	4	4	4	4.2	159.83	0	267650000	0	235730000	245340000	224480000
sp	Q96PK	RBM14	sp	Q96PK	1	17	17	17	32	69.491	3866900000	3855200000	3843100000	4371200000	4131600000	5096000000
sp	Q96PU	UHRF2	sp	Q96PU	1	2	2	2	3	89.984	0	0	0	46957000	0	0
sp	Q96PU	QKI	sp	Q96PU	1	2	2	2	7	37.67	116340000	91436000	110510000	0	104970000	79267000
sp	Q96PZ	PUS7	sp	Q96PZ	1	12	12	12	29	75.035	560520000	521310000	642330000	593380000	613510000	538090000

sp	Q96Q1	TRNT1	sp	Q96Q1	2	7	7	7	20	50.127	336380000	413390000	371820000	372870000	406480000	388810000
sp	Q96Q1	SMG1	sp	Q96Q1	1	1	1	1	0.6	410.5	0	0	0	12533000	0	0
sp	Q96QC	PPP1R10	sp	Q96QC	1	6	6	6	9.7	99.057	159310000	0	0	166620000	201280000	0
sp	Q96QD	SLC38A2	sp	Q96QD	1	3	3	3	11	56.025	352780000	415850000	442100000	291820000	301440000	348270000
sp	Q96QK	VPS35	sp	Q96QK	1	19	19	19	28	91.706	3063800000	3283500000	3441900000	3138700000	2944700000	2965800000
sp	Q96QR	PURB	sp	Q96QR	1	4	4	4	28	33.24	275980000	267700000	293990000	312990000	286410000	328980000
sp	Q96QU	XPO6	sp	Q96QU	1	2	2	2	2.1	128.88	0	0	25497000	32359000	0	0
sp	Q96R0	SPAG5	sp	Q96R0	1	4	4	4	5.4	134.42	165090000	306320000	146650000	185800000	148210000	204780000
sp	Q96RD	PANX1	sp	Q96RD	1	1	1	1	3.3	48.05	0	0	0	0	0	9832500
sp	Q96RE	NACC1	sp	Q96RE	2	5	5	5	15	57.258	136860000	139770000	144730000	127310000	0	0
sp	Q96RN	MED15	sp	Q96RN	1	1	1	1	1.4	86.753	0	0	0	0	0	15828000
sp	Q96RP	GFM1	sp	Q96RP	1	18	18	18	28	83.471	990020000	1154500000	1061500000	1063200000	1211700000	1099200000
sp	Q96RQ	ERGIC2	sp	Q96RQ	1	5	5	5	18	42.548	235260000	280570000	316400000	291230000	405750000	307040000
sp	Q96RQ	MCCC1	sp	Q96RQ	1	3	3	3	8.4	80.472	0	0	0	37742000	42899000	0
sp	Q96RS	NUDCD1	sp	Q96RS	1	9	9	9	18	66.755	429050000	491520000	430710000	397620000	435780000	442420000
sp	Q96RT	ERBIN	sp	Q96RT	1	2	2	2	3.5	158.3	0	45742000	0	34048000	0	0
sp	Q96RU	FNBP1	sp	Q96RU	1	2	2	2	6.2	71.306	0	0	0	0	26393000	0
sp	Q96S4	TP53RK	sp	Q96S4	1	4	4	4	23	28.16	206840000	187390000	162480000	201420000	172230000	151150000
sp	Q96S5	PIGS	sp	Q96S5	1	8	8	8	22	61.655	289950000	361430000	355560000	363340000	332920000	429590000
sp	Q96S5	WRNIP1	sp	Q96S5	1	4	4	4	9.2	72.132	0	0	0	0	136570000	0
sp	Q96S5	RANBP9	sp	Q96S5	1	2	2	2	4.7	77.846	0	32375000	0	0	38211000	0
sp	Q96S6	CLCC1	sp	Q96S6	1	1	1	1	3.1	62.022	0	0	0	0	0	30112000
sp	Q96S8	UBL7	sp	Q96S8	1	5	5	5	19	40.51	228910000	315260000	233580000	237950000	0	211500000
sp	Q96S9	MYADM	sp	Q96S9	1	3	3	3	14	35.273	289350000	307220000	272770000	355000000	357180000	332310000
sp	Q96SB	SRPK1	sp	Q96SB	2	7	7	6	14	74.324	636670000	527180000	643010000	420880000	417840000	491570000
sp	Q96SI9	STRBP	sp	Q96SI9	1	6	3	3	11	73.652	0	0	0	0	0	37928000
sp	Q96SK	TMEM209	sp	Q96SK	1	5	5	5	17	62.921	148430000	149730000	0	147020000	134780000	0
sp	Q96ST	IWS1	sp	Q96ST	1	4	4	4	6.8	91.954	216830000	110320000	127930000	155600000	189970000	110740000
sp	Q96ST	SIN3A	sp	Q96ST	1	9	9	9	12	145.17	234410000	252470000	340100000	304820000	295860000	298690000
sp	Q96SU	OSBPL9	sp	Q96SU	1	5	5	5	9.8	83.184	188800000	190650000	0	0	237600000	196700000
sp	Q96SW	CRBN	sp	Q96SW	1	1	1	1	4.8	50.545	0	0	0	19575000	0	0
sp	Q96SY	INTS14	sp	Q96SY	1	2	2	2	7.7	57.47	0	0	0	0	0	76651000
sp	Q96SZ	ADO	sp	Q96SZ	1	2	2	2	10	29.751	0	0	0	0	0	35891000
sp	Q96SZ	CDK5RAF	sp	Q96SZ	1	3	3	3	9.7	67.688	0	0	42236000	0	0	0
sp	Q96T2	RSF1	sp	Q96T2	1	5	5	5	4.9	163.82	131470000	189550000	170130000	140740000	129960000	112160000
sp	Q96T3	RBM15	sp	Q96T3	1	8	8	8	14	107.19	310060000	268970000	287400000	263440000	331560000	269640000
sp	Q96T5	RUFY1	sp	Q96T5	1	7	7	7	15	79.817	326450000	351440000	339190000	318520000	298760000	346850000
sp	Q96T7	MMS19	sp	Q96T7	1	6	6	6	11	113.29	0	268050000	193550000	171670000	125430000	143160000
sp	Q96T8	UHRF1	sp	Q96T8	1	11	11	11	18	89.813	385040000	356040000	299190000	228440000	220090000	256260000

sp|Q96TA	FAM129B	sp|Q96TA	1	22	22	22	40	84.137	3119300000	3721900000	3536400000	2802700000	2212100000	2791300000
sp|Q96TA	YME1L1	sp|Q96TA	1	7	7	7	12	86.454	246470000	225340000	309580000	230450000	188360000	249820000
sp|Q96TC	RMDN3	sp|Q96TC	1	8	8	8	23	52.118	293380000	167890000	306320000	539100000	283170000	322220000
sp|Q9941	MYCBP	sp|Q9941	1	5	5	5	52	11.967	668260000	580260000	598260000	458890000	586600000	556850000
sp|Q9942	TBCB	sp|Q9942	1	6	6	6	30	27.325	977340000	1014200000	772390000	915340000	905590000	954430000
sp|Q9943	PSMB7	sp|Q9943	1	6	6	6	26	29.965	1918300000	2494700000	2159600000	2008800000	2311500000	1937600000
sp|Q9943	CNN2	sp|Q9943	1	11	11	10	54	33.697	3244000000	3198300000	3273600000	3147800000	2884200000	2580500000
sp|Q9944	SEC62	sp|Q9944	1	1	1	1	2.3	45.861	0	0	0	0	0	94620000
sp|Q9944	PCYT2	sp|Q9944	1	3	3	3	13	43.835	245070000	276330000	264540000	228870000	301320000	351980000
sp|Q9945	CDC5L	sp|Q9945	1	20	20	20	36	92.25	1658300000	1872400000	1515100000	1664600000	1900300000	1800600000
sp|Q9946	PSMD1	sp|Q9946	1	37	37	37	49	105.84	5338500000	5224200000	4998500000	4829100000	6157100000	5810600000
sp|Q9947	SDF2	sp|Q9947	1	5	5	5	33	23.026	83222000	116230000	135810000	129400000	138530000	166410000
sp|Q9947	PFDN5	sp|Q9947	1	9	9	9	71	17.328	2083700000	1455200000	1897700000	1948800000	2797900000	1927400000
sp|Q9949	PARK7	sp|Q9949	1	12	12	12	74	19.891	1.1139E+10	1.4358E+10	1.046E+10	11741000000	14105000000	1.1584E+10
sp|Q9950	EYA3	sp|Q9950	1	2	2	2	5.8	62.662	70416000	0	0	0	0	0
sp|Q9953	VAT1	sp|Q9953	1	11	11	11	47	41.92	1732200000	2590400000	2154800000	2114200000	2137500000	1898800000
sp|Q9953	LGMN	sp|Q9953	1	3	3	3	18	49.411	110700000	250390000	0	56852000	0	0
sp|Q9954	DNAJC2	sp|Q9954	1	8	8	8	16	71.996	287270000	232950000	245430000	213180000	224570000	226700000
sp|Q9954	MPHOSPI	sp|Q9954	1	2	2	2	18	19.024	0	0	99471000	0	0	122950000
sp|Q9955	MAP3K14	sp|Q9955	1	2	2	2	1.7	104.04	273300000	179980000	0	274210000	243920000	282380000
sp|Q9956	NUP88	sp|Q9956	1	13	13	13	26	83.541	934090000	992410000	685350000	1010300000	873180000	926910000
sp|Q9957	PIK3R4	sp|Q9957	1	3	3	3	3.1	153.1	0	0	0	0	0	59228000
sp|Q9957	POP1	sp|Q9957	1	13	13	13	24	114.71	514130000	438840000	516490000	548810000	404590000	458190000
sp|Q9958	S100A13	sp|Q9958	1	6	6	6	49	11.471	1958300000	1951300000	1869100000	1670500000	2265100000	1819000000
sp|Q9959	SCAF11	sp|Q9959	1	10	10	10	11	164.65	546100000	547550000	438380000	0	522770000	522910000
sp|Q9959	TIMM17A	sp|Q9959	1	1	1	1	13	18.023	0	0	0	0	38539000	0
sp|Q9959	TSNAX	sp|Q9959	1	6	6	6	30	33.112	429620000	362190000	416460000	554880000	452420000	367490000
sp|Q9961	EIF3C	sp|Q9961	2	29	29	29	38	105.34	4210500000	4884800000	4797600000	4312300000	4483000000	4578500000
sp|Q9961	TTC1	sp|Q9961	1	9	9	9	39	33.526	1110300000	874430000	1031600000	1015800000	899840000	998950000
sp|Q9961	DNAJC7	sp|Q9961	1	20	20	20	45	56.44	2222500000	2277800000	2221200000	2115100000	2024800000	2433300000
sp|Q9961	CDCA3	sp|Q9961	1	2	2	2	13	28.998	0	0	68928000	47822000	0	102530000
sp|Q9962	C12orf57	sp|Q9962	1	3	3	3	37	13.178	198300000	191790000	321240000	188670000	174530000	157180000
sp|Q9962	PHB2	sp|Q9962	1	14	14	14	53	33.296	6808500000	7739000000	8491600000	8566700000	7989000000	7614400000
sp|Q9962	COPS8	sp|Q9962	1	5	5	5	41	23.225	790860000	1001400000	837520000	787810000	890800000	930800000
sp|Q9963	PRPF18	sp|Q9963	1	1	1	1	5.8	39.859	0	0	0	0	0	0
sp|Q9964	SDHC	sp|Q9964	1	2	2	2	12	18.61	153300000	167860000	159160000	170400000	170010000	173960000
sp|Q9965	CHP1	sp|Q9965	1	3	3	3	24	22.456	78204000	0	83750000	89902000	88641000	0
sp|Q9966	KIF2C	sp|Q9966	1	10	10	9	25	81.312	373750000	299430000	390130000	421600000	473230000	464180000
sp|Q9970	ATXN2	sp|Q9970	1	5	5	5	5	140.28	0	188170000	0	0	0	134710000

sp|Q9970	MTR	sp|Q9970	1	3	3	3	4	140.53	0	81586000	0	0	0	0
sp|Q9971	HSD17B1	sp|Q9971	1	15	15	15	79	26.923	6172800000	6468500000	6224700000	7035500000	7033900000	5760600000
sp|Q9971	COL12A1	sp|Q9971	1	15	15	15	7.5	333.14	498770000	615060000	554910000	396670000	328510000	440460000
sp|Q9972	SIGMAR1	sp|Q9972	1	3	3	3	24	25.127	658530000	520610000	597840000	564000000	650060000	557210000
sp|Q9972	HNRNPAE	sp|Q9972	1	6	6	6	19	36.224	2636700000	2429900000	2651100000	2091200000	1931200000	1772300000
sp|Q9973	NAP1L4	sp|Q9973	1	13	13	12	46	42.823	3810400000	4811500000	3994900000	4108000000	4044000000	4180600000
sp|Q9973	MGST2	sp|Q9973	1	1	1	1	9.5	16.62	0	0	0	0	0	692700000
sp|Q9974	NAPG	sp|Q9974	1	5	5	5	21	34.746	191350000	216960000	219600000	223090000	209260000	0
sp|Q9975	TXN2	sp|Q9975	1	3	3	3	37	18.383	311640000	293510000	384380000	489720000	432460000	306100000
sp|Q9979	ACO2	sp|Q9979	1	24	24	24	43	85.424	2903400000	2827800000	3062500000	3343600000	3177800000	3422200000
sp|Q9980	TM9SF2	sp|Q9980	1	9	9	9	19	75.775	2330100000	2064200000	2296100000	2616300000	2685300000	2489800000
sp|Q9980	COQ7	sp|Q9980	1	1	1	1	8.3	24.277	0	0	0	0	0	30634000
sp|Q9980	SLC29A1	sp|Q9980	1	4	4	4	9.4	50.219	225840000	273410000	197040000	158970000	130380000	123090000
sp|Q9981	TSG101	sp|Q9981	1	6	6	6	23	43.944	316580000	280620000	199120000	252420000	477200000	304410000
sp|Q9982	CPNE1	sp|Q9982	1	9	9	9	21	59.058	903740000	913370000	924980000	966310000	878340000	800300000
sp|Q9983	CCT7	sp|Q9983	1	29	29	29	61	59.366	1.3389E+10	1.3546E+10	1.2675E+10	11845000000	12414000000	1.3665E+10
sp|Q9983	MYD88	sp|Q9983	1	2	2	2	13	33.233	0	0	0	0	0	88734000
sp|Q9984	EBNA1BP	sp|Q9984	1	11	11	11	36	34.852	1684200000	1832100000	1771900000	1655900000	1778300000	1864600000
sp|Q9987	HAUS7	sp|Q9987	1	1	1	1	4.3	40.778	0	0	0	0	0	19146000
sp|Q9987	PRMT1	sp|Q9987	2	14	14	14	38	42.461	4500600000	4646100000	4885500000	4567400000	4752000000	5073000000
sp|Q9993	BAG1	sp|Q9993	1	5	5	5	16	38.778	532490000	373520000	450100000	422280000	536490000	423030000
sp|Q9994	ATF6B	sp|Q9994	1	1	1	1	2	76.708	0	0	0	0	0	2958000
sp|Q9994	RNF5	sp|Q9994	1	1	1	1	8.3	19.881	0	0	0	0	0	48053000
sp|Q9994	AGPAT1	sp|Q9994	1	1	1	1	7.4	31.716	14345000	0	24060000	42163000	29022000	44616000
sp|Q9996	SH3GL1	sp|Q9996	2	8	8	8	27	41.489	919280000	967310000	906480000	976760000	1152300000	1155400000
sp|Q9998	VRK1	sp|Q9998	1	6	6	6	23	45.476	158310000	0	0	0	160230000	175990000
sp|Q9BPV	NIPSNAP	sp|Q9BPV	1	4	4	4	16	33.31	179720000	256290000	211850000	164610000	214640000	233580000
sp|Q9BPX	NCAPG	sp|Q9BPX	1	19	19	19	27	114.33	974840000	1099200000	1191400000	926810000	932450000	810830000
sp|Q9BPX	ARPC5L	sp|Q9BPX	1	4	3	3	46	16.941	519310000	536200000	0	366380000	646330000	475480000
sp|Q9BPX	MICU1	sp|Q9BPX	1	1	1	1	4.4	54.351	0	0	0	0	37346000	0
sp|Q9BPZ	PAIP2	sp|Q9BPZ	1	2	2	2	35	14.984	0	0	0	85620000	58433000	124810000
sp|Q9BPZ	MAPKAP1	sp|Q9BPZ	1	1	1	1	3.3	59.122	0	0	41220000	0	0	0
sp|Q9BQ0	RBM4B	sp|Q9BQ0	1	11	1	1	30	40.149	0	0	0	0	0	31010000
sp|Q9BQ1	NABP2	sp|Q9BQ1	1	1	1	1	3.3	22.338	0	0	0	0	46198000	0
sp|Q9BQ3	DDX50	sp|Q9BQ3	1	8	6	6	16	82.564	129990000	143880000	168890000	143260000	160900000	137740000
sp|Q9BQ4	MRPL34	sp|Q9BQ4	1	1	1	1	11	10.165	0	0	0	0	61002000	0
sp|Q9BQ5	ELAC2	sp|Q9BQ5	1	23	23	23	43	92.218	1715200000	1793100000	1360300000	1520400000	1505200000	1812000000
sp|Q9BQ6	TRIR	sp|Q9BQ6	1	5	5	5	35	18.419	310300000	0	99237000	117880000	288260000	189670000
sp|Q9BQ6	GRWD1	sp|Q9BQ6	1	7	7	7	30	49.419	1151100000	1127900000	1123400000	1260500000	1245800000	1306400000

sp|Q9BQ6	MACROD	sp|Q9BQ6	1	3	3	3	15	35.505	221360000	297950000	269350000	333170000	291920000	315750000
sp|Q9BQ7	TCF25	sp|Q9BQ7	1	3	3	3	5.5	76.666	345110000	0	0	0	311730000	0
sp|Q9BQ7	CMSS1	sp|Q9BQ7	1	3	3	3	12	31.884	0	0	0	0	118990000	0
sp|Q9BQ9	ECSIT	sp|Q9BQ9	1	3	3	3	14	49.148	49228000	60598000	0	83986000	68107000	0
sp|Q9BQA	WDR77	sp|Q9BQA	1	9	9	9	45	36.724	1374200000	1758700000	1343700000	1458400000	1395600000	1543600000
sp|Q9BQE	VKORC1	sp|Q9BQE	1	1	1	1	8	18.234	0	0	0	0	0	534910000
sp|Q9BQC	DPH2	sp|Q9BQC	1	1	1	1	3.9	52.082	0	0	0	0	15078000	0
sp|Q9BQC	MRPL57	sp|Q9BQC	1	2	2	2	30	12.266	291860000	413070000	142070000	431430000	937920000	778250000
sp|Q9BQD	KXD1	sp|Q9BQD	1	1	1	1	8.5	19.668	0	0	0	0	0	17530000
sp|Q9BQE	TUBA1C	sp|Q9BQE	1	21	21	4	61	49.895	1.0735E+11	1.0725E+11	1.0515E+11	98960000000	90018000000	1.0601E+11
sp|Q9BQE	SELENOS	sp|Q9BQE	1	1	1	1	9	21.163	0	0	0	0	0	0
sp|Q9BQE	APOL2	sp|Q9BQE	1	4	4	4	14	37.092	188250000	220390000	201420000	190790000	232460000	85456000
sp|Q9BQC	MYBBP1A	sp|Q9BQC	1	31	31	31	27	148.85	4222200000	5015700000	4510600000	4068600000	4621900000	4784500000
sp|Q9BQL	FERMT1	sp|Q9BQL	1	3	1	1	4.7	77.436	0	0	0	0	0	32959000
sp|Q9BQF	MGME1	sp|Q9BQF	1	5	5	5	20	39.42	0	105470000	0	0	0	0
sp|Q9BQC	GORASP1	sp|Q9BQC	1	1	1	1	4.8	46.482	0	0	0	0	0	30551000
sp|Q9BR6	ACBD6	sp|Q9BR6	1	3	3	3	15	31.15	0	0	0	0	0	84154000
sp|Q9BR7	CORO1B	sp|Q9BR7	1	11	11	10	30	54.234	1347600000	1194900000	1202700000	1154800000	1017900000	1281100000
sp|Q9BRA	TXNDC17	sp|Q9BRA	1	5	5	5	44	13.941	1451500000	1550700000	1666900000	1832900000	1539900000	1626100000
sp|Q9BRF	CPPED1	sp|Q9BRF	1	2	2	2	12	35.548	0	0	122360000	0	0	0
sp|Q9BRG	VPS25	sp|Q9BRG	1	2	2	2	17	20.747	0	152200000	0	0	0	0
sp|Q9BRJ	MRPL45	sp|Q9BRJ	1	6	6	6	31	35.351	296580000	528050000	441850000	233440000	372800000	130970000
sp|Q9BRJ	C7orf50	sp|Q9BRJ	1	7	7	7	46	22.083	1210400000	1169700000	1076000000	1151500000	1255200000	1413300000
sp|Q9BRJ	NUDT16L	sp|Q9BRJ	1	2	1	1	13	23.338	0	0	0	0	0	15380000
sp|Q9BRK	SDF4	sp|Q9BRK	1	4	4	4	11	41.806	161450000	164920000	139680000	228150000	210060000	0
sp|Q9BRF	PYM1	sp|Q9BRF	1	3	3	3	25	22.655	0	215380000	270980000	0	0	0
sp|Q9BRG	AIFM2	sp|Q9BRG	1	4	4	4	13	40.526	363480000	297210000	251650000	328000000	391210000	398060000
sp|Q9BRF	ADPGK	sp|Q9BRF	1	3	3	3	12	54.088	95153000	109190000	95537000	78090000	51454000	73762000
sp|Q9BRS	RIOK1	sp|Q9BRS	1	1	1	1	3	65.582	0	0	0	0	0	32226000
sp|Q9BRT	UQCC2	sp|Q9BRT	1	2	2	2	23	14.875	0	0	0	90032000	0	0
sp|Q9BRT	LLPH	sp|Q9BRT	1	2	2	2	22	15.225	162110000	152970000	161700000	0	0	143670000
sp|Q9BRT	GINS4	sp|Q9BRT	1	3	3	3	21	26.047	169440000	168330000	146170000	124050000	203840000	120770000
sp|Q9BRX	PELO	sp|Q9BRX	1	9	9	9	28	43.359	530820000	755590000	588580000	575700000	636110000	529780000
sp|Q9BRX	GINS3	sp|Q9BRX	1	2	2	2	16	24.534	0	0	0	0	58421000	0
sp|Q9BRX	FAM213A	sp|Q9BRX	1	1	1	1	4.8	25.764	0	0	0	0	0	37816000
sp|Q9BRZ	TRIM56	sp|Q9BRZ	1	3	3	3	6.2	81.487	59129000	0	55945000	59083000	65990000	64389000
sp|Q9BS2	ERP44	sp|Q9BS2	1	7	7	7	21	46.971	1836000000	2064900000	2051800000	2310500000	1858700000	1709700000
sp|Q9BS4	LXN	sp|Q9BS4	1	2	2	2	12	25.75	261170000	90201000	220110000	325450000	308870000	323850000
sp|Q9BSC	NOL10	sp|Q9BSC	1	8	8	8	22	80.301	246760000	273420000	397770000	292180000	396800000	294260000

sp	Q9BSD	NTPCR	sp	Q9BSD	1	7	7	7	48	20.713	498340000	507580000	401210000	602900000	526510000	515910000
sp	Q9BSE	AGMAT	sp	Q9BSE	1	1	1	1	8.5	37.66	0	0	0	0	0	104350000
sp	Q9BSF	TIMM29	sp	Q9BSF	1	1	1	1	6.5	29.233	0	0	0	18983000	0	0
sp	Q9BSH	TACO1	sp	Q9BSH	1	4	4	4	24	32.477	389340000	310070000	282030000	392860000	442140000	307330000
sp	Q9BSJ	TUBGCP2	sp	Q9BSJ	1	4	4	4	6.1	102.53	0	0	0	77448000	0	0
sp	Q9BSJ	ESYT1	sp	Q9BSJ	1	23	23	23	31	122.85	1929400000	2398700000	1981900000	2304200000	2387800000	2243700000
sp	Q9BSL	UBAC1	sp	Q9BSL	1	1	1	1	4.2	45.338	0	0	0	0	0	22459000
sp	Q9BSR	YIPF4	sp	Q9BSR	1	1	1	1	3.7	27.082	0	0	0	0	0	82659000
sp	Q9BSV	TSEN34	sp	Q9BSV	1	2	2	2	8.4	33.652	0	0	0	0	0	45056000
sp	Q9BT0	CNPY3	sp	Q9BT0	1	3	3	3	17	30.748	120090000	0	121930000	144280000	194280000	307180000
sp	Q9BT2	ALG1	sp	Q9BT2	2	6	6	6	18	52.518	445540000	393160000	326160000	401290000	376360000	363740000
sp	Q9BT7	PSMG3	sp	Q9BT7	1	4	4	4	51	13.104	203630000	164320000	200840000	333780000	239000000	255570000
sp	Q9BT7	COPS4	sp	Q9BT7	1	16	16	16	52	46.268	1452100000	1510600000	1529600000	1449600000	1434200000	1481800000
sp	Q9BTC	DIDO1	sp	Q9BTC	1	11	11	11	10	243.87	482480000	514540000	419290000	591660000	515770000	568960000
sp	Q9BTC	MTA3	sp	Q9BTC	1	5	2	2	9.8	67.503	89017000	44268000	0	0	58560000	106600000
sp	Q9BTD	RBM42	sp	Q9BTD	1	4	4	4	19	50.413	0	0	163270000	0	220650000	313190000
sp	Q9BTE	DCTN5	sp	Q9BTE	1	2	2	2	12	20.126	126420000	0	17950000	0	173850000	74214000
sp	Q9BTE	MCMBP	sp	Q9BTE	1	10	10	10	22	72.979	447960000	454820000	490290000	367780000	420720000	305080000
sp	Q9BTE	AARSD1	sp	Q9BTE	1	5	5	5	21	45.479	160260000	209630000	192860000	219070000	199690000	180110000
sp	Q9BTE	DCUN1D5	sp	Q9BTE	2	3	3	3	12	27.508	136490000	121000000	139540000	136280000	165320000	122820000
sp	Q9BTT	ANP32E	sp	Q9BTT	1	5	5	5	23	30.692	1729100000	1730400000	1803600000	1677600000	1469700000	1722700000
sp	Q9BTU	PI4K2A	sp	Q9BTU	1	2	2	2	4	54.022	0	0	0	26491000	0	0
sp	Q9BTV	TMEM43	sp	Q9BTV	1	14	14	14	51	44.875	1866600000	1808500000	1335100000	1804100000	1889400000	1577700000
sp	Q9BTW	TBCD	sp	Q9BTW	1	12	12	12	17	132.6	554050000	582260000	589360000	538220000	508000000	493570000
sp	Q9BTX	NDC1	sp	Q9BTX	1	8	8	8	20	76.304	185640000	197710000	359880000	378130000	432020000	378080000
sp	Q9BTY	HGH1	sp	Q9BTY	1	7	7	7	30	42.129	156610000	155470000	240670000	174770000	218970000	138440000
sp	Q9BTZ	DHRS4	sp	Q9BTZ	2	4	4	4	14	29.537	0	0	98832000	0	133710000	163090000
sp	Q9BU2	LMF2	sp	Q9BU2	1	1	1	1	3	79.697	0	0	0	0	0	0
sp	Q9BU6	NDUFAF3	sp	Q9BU6	1	2	2	2	13	20.35	0	0	0	0	0	120480000
sp	Q9BU7	MMTAG2	sp	Q9BU7	1	2	2	2	9.1	29.411	161740000	158090000	91806000	0	166590000	0
sp	Q9BU8	DOHH	sp	Q9BU8	1	5	5	5	29	32.904	175440000	161320000	127840000	176710000	214650000	236930000
sp	Q9BUE	TMEM70	sp	Q9BUE	1	2	2	2	8.8	28.969	0	0	0	0	0	26183000
sp	Q9BUE	ISCA1	sp	Q9BUE	1	1	1	1	10	14.179	0	0	0	0	0	42604000
sp	Q9BUF	TUBB6	sp	Q9BUF	1	22	14	9	73	49.857	3907400000	3719800000	4087700000	4761400000	3396300000	4009800000
sp	Q9BUH	PAXX	sp	Q9BUH	1	2	2	2	15	21.639	0	0	0	0	0	76421000
sp	Q9BUI	POLR3C	sp	Q9BUI	1	3	3	3	9.4	60.611	0	0	0	0	0	110390000
sp	Q9BUJ	HNRNPUL	sp	Q9BUJ	1	22	22	22	35	95.737	2448500000	1885100000	2522500000	2783700000	2931700000	2909200000
sp	Q9BUK	MSTO1	sp	Q9BUK	1	5	5	4	7.5	61.835	405240000	220370000	348370000	311230000	284300000	415750000
sp	Q9BUL	PDCD10	sp	Q9BUL	1	4	4	4	26	24.701	432870000	366630000	422810000	336800000	345670000	399640000

sp	Q9BUL	RPP25	sp	Q9BUL	1	2	2	2	22	20.632	0	0	0	0	0	51703000
sp	Q9BUM	G6PC3	sp	Q9BUM	1	1	1	1	6.1	38.734	0	18420000	0	0	0	0
sp	Q9BUN	DERL1	sp	Q9BUN	1	4	4	4	14	28.8	207410000	234440000	239330000	261640000	187170000	221760000
sp	Q9BUP	HTATIP2	sp	Q9BUP	1	7	7	7	34	27.049	901900000	655130000	832840000	973720000	758060000	706070000
sp	Q9BUQ	DDX23	sp	Q9BUQ	1	17	17	17	24	95.581	719480000	712570000	633560000	698360000	719990000	850630000
sp	Q9BUR	WRAP53	sp	Q9BUR	1	7	7	7	24	59.309	195690000	232510000	195990000	122200000	222680000	186310000
sp	Q9BUR	APOO	sp	Q9BUR	1	5	5	5	33	22.284	0	191060000	303350000	0	274930000	322900000
sp	Q9BV2	MRI1	sp	Q9BV2	1	4	4	4	15	39.149	0	237230000	171730000	0	0	0
sp	Q9BV3	WDR18	sp	Q9BV3	1	6	6	6	17	47.405	324030000	296170000	372960000	401850000	436820000	0
sp	Q9BV4	VAMP8	sp	Q9BV4	1	2	2	2	18	11.438	0	0	0	0	0	322130000
sp	Q9BV4	THUMPD3	sp	Q9BV4	1	8	8	8	25	57.002	278410000	381790000	201520000	262980000	181360000	247320000
sp	Q9BV5	ADI1	sp	Q9BV5	1	4	4	4	28	21.498	219470000	148790000	0	199340000	168750000	159170000
sp	Q9BV6	RNF126	sp	Q9BV6	1	1	1	1	7.4	35.584	0	0	0	0	0	165020000
sp	Q9BV8	EMC6	sp	Q9BV8	1	1	1	1	9.1	12.017	0	0	0	0	0	0
sp	Q9BV8	NTMT1	sp	Q9BV8	1	8	8	8	50	25.387	500180000	846210000	618880000	585150000	785770000	629610000
sp	Q9BVC	MLST8	sp	Q9BVC	1	3	3	3	19	35.876	200820000	174520000	142830000	178880000	162440000	0
sp	Q9BVC	TMEM109	sp	Q9BVC	1	2	2	2	9.1	26.21	676170000	746430000	675280000	805200000	972780000	1072300000
sp	Q9BVG	PBDC1	sp	Q9BVG	1	4	4	4	23	26.056	370520000	305300000	349640000	345260000	436910000	303780000
sp	Q9BVG	PTDSS2	sp	Q9BVG	1	2	2	2	6.2	56.252	0	51668000	34812000	0	0	55348000
sp	Q9BVI4	NOC4L	sp	Q9BVI4	1	7	7	7	17	58.467	268360000	350170000	283590000	296670000	364280000	316630000
sp	Q9BVJ	UTP14A	sp	Q9BVJ	2	8	8	8	16	87.977	315640000	365230000	355540000	351640000	410140000	263510000
sp	Q9BVJ	DUSP23	sp	Q9BVJ	1	3	3	3	26	16.588	71325000	0	0	0	0	0
sp	Q9BVK	TMED9	sp	Q9BVK	1	8	8	7	35	27.277	1270200000	1036600000	907730000	1340600000	1088200000	629220000
sp	Q9BVL	NUP58	sp	Q9BVL	1	3	3	3	8.3	60.896	0	0	243250000	0	0	0
sp	Q9BVL	SELENOO	sp	Q9BVL	1	1	1	1	1.8	73.488	0	0	0	0	0	0
sp	Q9BVP	GNL3	sp	Q9BVP	1	17	17	17	38	61.992	1186700000	1338300000	1169100000	1508200000	1148700000	1249800000
sp	Q9BVQ	SPATA5L	sp	Q9BVQ	1	5	5	5	11	80.709	50775000	0	43374000	0	0	0
sp	Q9BVS	RIOK2	sp	Q9BVS	1	2	2	2	5.8	63.282	0	0	0	38247000	29046000	0
sp	Q9BVT	TMUB1	sp	Q9BVT	1	2	2	2	16	26.261	0	148880000	160070000	0	0	0
sp	Q9BW1	KIFC1	sp	Q9BW1	1	7	7	7	13	73.747	294740000	334370000	293390000	427910000	366090000	352880000
sp	Q9BW2	NUP85	sp	Q9BW2	1	10	10	10	23	75.019	941800000	1058700000	976070000	985640000	1085100000	1046500000
sp	Q9BW6	ELOVL1	sp	Q9BW6	1	2	2	2	9.7	32.662	252910000	241420000	236010000	315210000	171110000	120370000
sp	Q9BW7	HIRIP3	sp	Q9BW7	1	2	2	2	5.4	61.956	0	0	0	0	93096000	0
sp	Q9BW7	HIGD2A	sp	Q9BW7	1	1	1	1	27	11.528	637470000	166170000	197280000	866270000	605850000	0
sp	Q9BW9	NUDT9	sp	Q9BW9	1	3	3	3	11	39.125	173890000	113320000	134550000	113190000	144610000	109460000
sp	Q9BW9	TARS2	sp	Q9BW9	1	4	4	4	9.5	81.035	0	68290000	0	0	0	0
sp	Q9BWD	ACAT2	sp	Q9BWD	1	13	13	13	57	41.35	3430000000	2798500000	2570200000	2876100000	2937800000	2580600000
sp	Q9BWF	RBM4	sp	Q9BWF	1	13	13	3	41	40.313	1640400000	1344400000	1451800000	1446200000	1478900000	1594000000
sp	Q9BWH	FUNDC2	sp	Q9BWH	1	2	2	2	13	20.675	0	0	0	0	872150000	0

sp|Q9BWJ	SF3B5	sp|Q9BWJ	1	4	4	4	56	10.135	1711000000	1530200000	1263900000	1256200000	1494400000	1696900000
sp|Q9BWI	SFXN3	sp|Q9BWI	1	10	9	9	42	35.503	924200000	922550000	813410000	1100600000	1305200000	1151900000
sp|Q9BWC	YIPF2	sp|Q9BWC	1	1	1	1	9.2	35.151	0	0	0	0	0	33804000
sp|Q9BWU	SLC4A1A	sp|Q9BWU	1	3	3	3	5.9	88.813	0	0	0	146510000	0	0
sp|Q9BX4	LSM14B	sp|Q9BX4	1	5	5	5	27	42.07	122130000	134400000	100500000	0	161520000	167360000
sp|Q9BX6	SORBS1	sp|Q9BX6	1	1	1	1	0.8	142.51	0	190950000	527140000	223660000	0	0
sp|Q9BX6	HINT2	sp|Q9BX6	1	6	6	6	52	17.162	818640000	894980000	1060000000	1207200000	1232500000	985130000
sp|Q9BXB	OSBPL11	sp|Q9BXB	1	3	2	2	5.1	83.642	60256000	67684000	61077000	52921000	26748000	64086000
sp|Q9BXB	OSBPL10	sp|Q9BXB	1	5	5	4	8.5	83.969	213050000	350840000	312830000	228040000	206600000	254090000
sp|Q9BXJ	NAA15	sp|Q9BXJ	2	21	21	21	32	101.27	2519600000	2810800000	2441100000	2167300000	2283500000	2587900000
sp|Q9BXK	BCL2L13	sp|Q9BXK	1	4	4	4	16	52.723	0	160580000	146380000	0	123260000	0
sp|Q9BXP	SRRT	sp|Q9BXP	1	19	19	19	26	100.67	2827000000	3080900000	3110000000	3335800000	3261800000	2870200000
sp|Q9BXR	QTRT1	sp|Q9BXR	1	4	4	4	17	44.047	0	75456000	0	0	0	0
sp|Q9BXS	AP1M1	sp|Q9BXS	2	13	13	13	40	48.586	615250000	806100000	752500000	718630000	724050000	699370000
sp|Q9BXS	NUSAP1	sp|Q9BXS	1	3	3	3	8.2	49.451	0	145740000	0	0	0	90790000
sp|Q9BXT	CACNG6	sp|Q9BXT	1	1	1	1	2.7	28.129	0	0	0	0	0	383660000
sp|Q9BXU	STK31	sp|Q9BXU	1	1	1	1	0.8	115.69	0	0	0	0	0	30467000
sp|Q9BXV	GON7	sp|Q9BXV	1	2	2	2	41	10.859	0	0	0	0	136730000	0
sp|Q9BXW	HDHD5	sp|Q9BXW	1	5	5	5	19	46.321	160410000	282940000	158860000	174270000	178100000	139810000
sp|Q9BXW	FANCD2	sp|Q9BXW	1	6	6	6	5.7	164.13	0	392140000	346940000	0	188000000	0
sp|Q9BXY	MAK16	sp|Q9BXY	1	4	4	4	19	35.368	248310000	206100000	226040000	180830000	193990000	241710000
sp|Q9BY3	ITPA	sp|Q9BY3	1	5	5	5	44	21.445	585050000	537560000	472520000	412780000	563960000	528090000
sp|Q9BY4	RTFDC1	sp|Q9BY4	1	2	2	2	9.5	33.886	92449000	60653000	135080000	102160000	0	75735000
sp|Q9BY4	CHMP4A	sp|Q9BY4	1	6	6	6	42	25.098	245810000	445100000	216030000	343260000	330960000	381540000
sp|Q9BY4	EIF2A	sp|Q9BY4	1	19	19	19	45	64.989	2462500000	2416200000	2489000000	2042900000	1910800000	2091300000
sp|Q9BY7	POLDIP3	sp|Q9BY7	1	7	7	7	23	46.089	935440000	953700000	819040000	1052600000	1141400000	683770000
sp|Q9BYC	MRPL32	sp|Q9BYC	1	2	2	2	12	21.405	0	36842000	0	0	0	0
sp|Q9BYC	MRPL20	sp|Q9BYC	1	3	3	3	26	17.442	0	140130000	0	0	0	228060000
sp|Q9BYD	MRPL13	sp|Q9BYD	1	8	8	8	48	20.692	399500000	382780000	349710000	335220000	359650000	343600000
sp|Q9BYD	MRPL9	sp|Q9BYD	1	5	5	5	24	30.243	327420000	305720000	302450000	273670000	349810000	368010000
sp|Q9BYD	MRPL4	sp|Q9BYD	1	7	7	7	42	34.919	545710000	741530000	732890000	575760000	492390000	443940000
sp|Q9BYD	MRPL1	sp|Q9BYD	1	4	4	4	23	36.908	273580000	264160000	328470000	519150000	509230000	492230000
sp|Q9BYG	NIFK	sp|Q9BYG	1	9	9	9	44	34.222	977630000	909920000	911800000	1041500000	1084600000	1189100000
sp|Q9BYJ	YTHDF1	sp|Q9BYJ	1	7	2	2	14	60.873	58860000	0	0	70944000	84144000	75007000
sp|Q9BYN	SRXN1	sp|Q9BYN	1	3	3	3	34	14.259	134540000	121540000	128810000	137560000	196810000	126890000
sp|Q9BYN	MRPS26	sp|Q9BYN	1	3	3	3	15	24.211	0	253260000	250440000	0	223750000	324020000
sp|Q9BYT	NLN	sp|Q9BYT	1	11	11	11	19	80.651	640800000	582600000	489660000	608670000	598750000	474650000
sp|Q9BYX	TBC1D2	sp|Q9BYX	1	5	5	5	9.6	105.41	490130000	657640000	373830000	540630000	583350000	683150000
sp|Q9BZD	NUF2	sp|Q9BZD	1	2	2	2	3.9	54.303	0	0	42896000	0	0	0

sp	Q9BZE	MRPL37	sp	Q9BZE	1	8	8	8	28	48.117	540060000	711140000	571630000	572320000	636400000	739490000
sp	Q9BZE	GTPBP4	sp	Q9BZE	1	19	19	19	38	73.964	2163900000	2145600000	2113800000	2683300000	2195400000	2406900000
sp	Q9BZF	OSBPL8	sp	Q9BZF	1	7	7	7	12	101.19	212710000	169100000	201070000	182600000	154340000	202620000
sp	Q9BZG	RAB34	sp	Q9BZG	1	3	3	3	13	29.044	172850000	192810000	198330000	179410000	214230000	158590000
sp	Q9BZH	WDR11	sp	Q9BZH	1	3	3	3	3.5	136.68	0	58859000	0	82249000	58515000	0
sp	Q9BZI7	UPF3B	sp	Q9BZI7	1	5	5	5	15	57.761	204010000	164430000	209040000	192070000	164270000	204240000
sp	Q9BZJ	CRNKL1	sp	Q9BZJ	1	4	4	4	5.7	100.45	0	133530000	109590000	168230000	0	128680000
sp	Q9BZK	TBL1XR1	sp	Q9BZK	1	10	10	4	33	55.594	601920000	693860000	483630000	430530000	545400000	489640000
sp	Q9BZL	UBL5	sp	Q9BZL	1	1	1	1	15	8.5468	0	0	0	0	20720000	0
sp	Q9BZV	UBXN6	sp	Q9BZV	1	4	4	4	18	49.753	66927000	113140000	77947000	100580000	152110000	73598000
sp	Q9BZX	UCK2	sp	Q9BZX	1	3	3	3	17	29.299	0	187300000	239290000	307310000	302560000	286160000
sp	Q9BZZ	API5	sp	Q9BZZ	1	8	8	8	17	59.004	1367900000	1349600000	896800000	1134000000	1295800000	1127000000
sp	Q9C00	DPY30	sp	Q9C00	1	2	2	2	38	11.25	215020000	249820000	0	162620000	233670000	186110000
sp	Q9C0B	FTO	sp	Q9C0B	1	7	7	7	20	58.281	159480000	166650000	217430000	114800000	98828000	139630000
sp	Q9C0B	ZDHHC5	sp	Q9C0B	1	1	1	1	2.2	77.544	0	0	0	0	0	43864000
sp	Q9C0B	TANGO6	sp	Q9C0B	1	1	1	1	1.6	120.75	0	0	0	11575000	0	0
sp	Q9C0C	TNKS1BP	sp	Q9C0C	1	28	28	28	28	181.79	1052900000	1093400000	970610000	1123800000	1115200000	957140000
sp	Q9C0C	UBE2O	sp	Q9C0C	1	7	7	7	9.1	141.29	202950000	333450000	245520000	184480000	232490000	218680000
sp	Q9C0D	SELENOI	sp	Q9C0D	1	1	1	1	4	45.228	0	0	0	0	0	137030000
sp	Q9C0E	XPO4	sp	Q9C0E	1	3	3	3	4.1	130.14	129080000	101800000	106390000	100230000	0	76398000
sp	Q9C0E	LNPK	sp	Q9C0E	1	3	3	3	7.9	47.739	117590000	99308000	75839000	0	0	0
sp	Q9C0I1	MTMR12	sp	Q9C0I1	1	4	4	4	7.9	86.147	0	0	0	70059000	0	0
sp	Q9C0J	WDR33	sp	Q9C0J	1	4	4	4	6.6	145.89	83521000	77858000	74551000	79535000	90640000	69532000
sp	Q9GZL	WDR12	sp	Q9GZL	1	13	13	13	42	47.707	1758400000	1829200000	1755900000	1809400000	1782800000	1663900000
sp	Q9GZM	YIPF3	sp	Q9GZM	1	1	1	1	2.6	38.247	0	0	0	0	0	54964000
sp	Q9NXR	NDE1	sp	Q9NXR	2	1	1	1	3.3	37.72	0	0	0	0	0	0
sp	Q9GZN	C20orf27	sp	Q9GZN	1	2	2	2	19	19.291	0	0	138240000	137700000	0	125800000
sp	Q9GZP	PITHD1	sp	Q9GZP	1	6	6	6	41	24.178	273430000	108690000	109960000	287130000	293210000	183670000
sp	Q9GZP	IMUP	sp	Q9GZP	1	1	1	1	26	10.897	0	397760000	396420000	0	0	0
sp	Q9GZP	DERL2	sp	Q9GZP	2	4	4	4	21	27.567	114810000	128120000	0	87845000	146770000	89829000
sp	Q9GZR	REXO4	sp	Q9GZR	1	4	4	4	13	46.671	186060000	176860000	155600000	310110000	213930000	239090000
sp	Q9GZR	DDX24	sp	Q9GZR	1	11	11	11	19	96.331	459610000	294430000	388100000	473340000	413200000	345890000
sp	Q9GZS	POLR1E	sp	Q9GZS	1	2	2	2	7.3	53.961	223380000	146290000	207040000	204300000	217230000	204580000
sp	Q9GZS	WDR61	sp	Q9GZS	1	10	10	10	44	33.58	1323700000	1079800000	1035200000	1137700000	1327000000	1197900000
sp	Q9GZT	SLIRP	sp	Q9GZT	1	3	3	3	30	12.349	763390000	901200000	676230000	751380000	729040000	765510000
sp	Q9GZT	NIF3L1	sp	Q9GZT	1	8	8	8	29	41.968	544800000	426160000	551840000	535210000	271570000	349670000
sp	Q9GZU	FAM192A	sp	Q9GZU	1	3	3	3	16	28.912	0	172960000	0	0	0	0
sp	Q9GZY	MFF	sp	Q9GZY	1	8	8	8	30	38.464	687530000	718320000	440800000	700720000	462240000	642050000
sp	Q9GZZ	NAA50	sp	Q9GZZ	1	10	10	10	56	19.398	1644100000	1592000000	1152800000	1392700000	1482700000	1248600000

sp|Q9GZZ	UBA5	sp|Q9GZZ	1	4	4	4	17	44.863	0	247850000	132420000	215980000	178950000	0
sp|Q9H01	MTFR1L	sp|Q9H01	1	2	2	2	13	31.957	0	0	0	0	0	31861000
sp|Q9H06	TMEM126	sp|Q9H06	1	2	2	2	21	21.527	0	67143000	0	88372000	120770000	93958000
sp|Q9H06	MAF1	sp|Q9H06	1	1	1	1	5.1	28.77	0	0	0	20371000	0	0
sp|Q9H07	PAIP1	sp|Q9H07	1	12	12	12	32	53.524	1846200000	1736400000	1383900000	1508200000	1866900000	1419100000
sp|Q9H07	CLPB	sp|Q9H07	1	9	9	9	18	78.728	288680000	386720000	289350000	222670000	247080000	344840000
sp|Q9H08	LSG1	sp|Q9H08	1	7	7	7	16	75.225	97505000	161260000	96732000	0	114840000	150160000
sp|Q9H09	FAM107B	sp|Q9H09	1	3	3	3	33	15.558	390150000	421000000	341920000	326330000	396760000	305340000
sp|Q9H0A	NAT10	sp|Q9H0A	1	24	24	24	31	115.73	2288300000	2441600000	2151300000	2779900000	2180100000	2050600000
sp|Q9H0A	COMMD4	sp|Q9H0A	1	3	3	3	26	21.764	114190000	137290000	142160000	117070000	0	113420000
sp|Q9H0B	KLC2	sp|Q9H0B	1	9	7	7	18	68.934	290010000	396730000	324260000	362040000	383690000	370270000
sp|Q9H0C	ILKAP	sp|Q9H0C	1	8	8	8	23	42.906	225920000	178170000	271060000	232550000	279850000	374590000
sp|Q9H0D	XRN2	sp|Q9H0D	1	12	12	12	18	108.58	583660000	731660000	667920000	611340000	519810000	606380000
sp|Q9H0E	TOLLIP	sp|Q9H0E	1	1	1	1	5.1	30.281	0	0	0	0	19913000	0
sp|Q9H0E	SAP130	sp|Q9H0E	1	1	1	1	1.1	110.32	0	0	0	0	11547000	0
sp|Q9H0F	ARL6	sp|Q9H0F	1	1	1	1	10	21.097	0	0	0	0	0	0
sp|Q9H0G	NSRP1	sp|Q9H0G	1	1	1	1	2.5	66.389	0	0	0	0	0	35848000
sp|Q9H0H	RACGAP1	sp|Q9H0H	1	8	8	8	22	71.026	273240000	382120000	275890000	228700000	187380000	213410000
sp|Q9H0J	PARP12	sp|Q9H0J	1	2	2	2	6.3	79.063	0	0	31001000	39229000	0	0
sp|Q9H0L	CSTF2T	sp|Q9H0L	1	8	3	3	21	64.436	110790000	0	0	100990000	84368000	98017000
sp|Q9H0P	NT5C3A	sp|Q9H0P	1	6	6	6	21	37.948	254430000	264780000	230840000	278170000	314580000	248140000
sp|Q9H0R	TMEM222	sp|Q9H0R	1	2	2	2	21	23.23	70541000	79265000	0	66923000	79543000	60871000
sp|Q9H0S	DDX47	sp|Q9H0S	1	11	11	11	35	50.646	989920000	1009800000	934980000	643460000	1063500000	1018500000
sp|Q9H0U	MAGT1	sp|Q9H0U	1	9	9	9	24	38.036	739150000	1087900000	910670000	675040000	988230000	763380000
sp|Q9H0U	RAB1B	sp|Q9H0U	2	10	9	5	69	22.171	2780500000	3131300000	2655800000	3140200000	3867100000	2662800000
sp|Q9H0U	MRPL18	sp|Q9H0U	1	3	3	3	18	20.576	0	0	0	202150000	0	0
sp|Q9H0U	TSPYL1	sp|Q9H0U	1	1	1	1	4.6	49.192	0	0	0	0	0	37207000
sp|Q9H0W	C11orf54	sp|Q9H0W	1	3	3	3	17	35.117	0	0	0	0	121200000	0
sp|Q9H17	SIL1	sp|Q9H17	1	1	1	1	2.6	52.084	0	0	0	0	0	11185000
sp|Q9H1A	ANAPC1	sp|Q9H1A	1	3	3	3	1.9	216.5	0	0	0	0	0	43244000
sp|Q9H1B	IRF2BPL	sp|Q9H1B	1	7	4	4	11	82.658	152600000	231010000	0	156270000	141780000	0
sp|Q9H1C	CYSTM1	sp|Q9H1C	1	1	1	1	10	10.631	0	0	0	0	0	65510000
sp|Q9H1E	NUCKS1	sp|Q9H1E	1	5	5	5	28	27.296	1010500000	1072000000	1281100000	972700000	1028500000	1014900000
sp|Q9H1I8	ASCC2	sp|Q9H1I8	1	5	5	5	12	86.359	142340000	117290000	320210000	156010000	112390000	141370000
sp|Q9H1K	ISCU	sp|Q9H1K	1	2	2	2	22	17.999	0	0	0	0	0	96112000
sp|Q9H1Y	ATG5	sp|Q9H1Y	1	1	1	1	5.5	32.447	0	0	0	0	0	49660000
sp|Q9H22	EHD4	sp|Q9H22	1	12	11	10	31	61.174	674840000	531690000	460300000	582960000	599250000	502000000
sp|Q9H26	VPS33B	sp|Q9H26	1	3	3	3	8.1	70.584	60523000	0	91810000	0	0	0
sp|Q9H29	SH3BGRL	sp|Q9H29	1	4	4	4	67	10.438	2057000000	2978100000	2126400000	1414600000	2849300000	1732100000

sp|Q9H2G	SLK	sp|Q9H2G	1	13	13	13	15	142.69	450500000	518840000	220880000	297360000	354030000	436000000
sp|Q9H2H	PPIL3	sp|Q9H2H	1	5	5	5	40	18.154	377200000	367720000	295870000	360600000	547010000	478840000
sp|Q9H2J	PDCL3	sp|Q9H2J	1	4	4	4	26	27.614	142300000	113620000	132880000	121550000	114200000	132050000
sp|Q9H2M	RAB3GAF	sp|Q9H2M	1	12	12	12	12	155.98	349300000	576150000	279720000	514000000	382240000	482890000
sp|Q9H2P	ADNP	sp|Q9H2P	1	9	9	9	13	123.56	598150000	602790000	600200000	632680000	485220000	580480000
sp|Q9H2U	DHX36	sp|Q9H2U	1	13	13	13	20	114.76	661010000	636880000	639800000	501820000	441360000	519220000
sp|Q9H2U	PPA2	sp|Q9H2U	1	11	11	11	41	37.92	1346400000	1555100000	1392600000	1327900000	1622100000	1635400000
sp|Q9H2V	SPNS1	sp|Q9H2V	1	2	2	2	5.9	56.629	0	0	0	0	0	26313000
sp|Q9H2W	MRPL46	sp|Q9H2W	1	5	5	5	24	31.705	344470000	338780000	354540000	266470000	396270000	387460000
sp|Q9H30	PNN	sp|Q9H30	1	11	11	11	19	81.627	1550600000	1252000000	1419600000	1157700000	1621000000	1448100000
sp|Q9H33	TMEM245	sp|Q9H33	1	3	3	3	4.7	97.356	76556000	367140000	110220000	115030000	0	0
sp|Q9H3K	GHITM	sp|Q9H3K	1	3	3	3	6.7	37.205	383060000	290450000	245840000	0	0	309890000
sp|Q9H3K	BOLA2	sp|Q9H3K	1	6	6	6	80	10.116	2241000000	2237600000	2136600000	2125200000	2139400000	2245800000
sp|Q9H3N	TMX1	sp|Q9H3N	1	7	7	7	24	31.791	696460000	737910000	715000000	881280000	738840000	722780000
sp|Q9H3P	NELFA	sp|Q9H3P	1	8	8	8	28	57.276	181410000	467080000	0	201560000	278370000	266900000
sp|Q9H3P	ACBD3	sp|Q9H3P	1	10	10	10	28	60.593	725040000	848830000	527780000	779310000	930320000	705640000
sp|Q9H3Q	CDC42EP	sp|Q9H3Q	1	5	5	5	27	37.979	165970000	142310000	0	0	123320000	115410000
sp|Q9H3R	CENPH	sp|Q9H3R	1	2	2	2	12	28.481	0	64141000	0	0	0	0
sp|Q9H3S	PTPN23	sp|Q9H3S	1	10	10	10	10	178.97	305070000	328170000	224590000	299350000	292930000	305300000
sp|Q9H3U	UNC45A	sp|Q9H3U	1	20	20	20	28	103.08	1403200000	1396100000	1218800000	1355600000	1316200000	1241400000
sp|Q9H3Y	PPDPF	sp|Q9H3Y	1	1	1	1	15	11.777	0	0	0	0	18689000	0
sp|Q9H3Z	DNAJC5	sp|Q9H3Z	1	2	2	2	20	22.149	0	0	44469000	0	0	0
sp|Q9H41	DSN1	sp|Q9H41	1	2	2	2	8.7	40.067	31205000	104890000	0	54080000	0	0
sp|Q9H44	CHMP4B	sp|Q9H44	2	9	9	8	46	24.95	1644800000	1578100000	1428900000	1496700000	1846700000	1564200000
sp|Q9H44	RWDD1	sp|Q9H44	1	3	3	3	18	27.939	72149000	70472000	64145000	67389000	0	65166000
sp|Q9H48	POFUT1	sp|Q9H48	1	8	8	8	34	43.955	836550000	841930000	687800000	886250000	730810000	934050000
sp|Q9H49	PIGU	sp|Q9H49	1	2	2	2	6.4	50.051	91789000	114290000	100400000	124900000	0	0
sp|Q9H4A	WNK1	sp|Q9H4A	4	14	14	14	9.2	250.79	478310000	508050000	458970000	532510000	631380000	469930000
sp|Q9H4A	RNPEP	sp|Q9H4A	1	16	16	16	32	72.595	1916900000	2236700000	1896400000	1910300000	1972900000	1815100000
sp|Q9H4A	GOLPH3	sp|Q9H4A	1	7	7	7	35	33.81	811710000	756120000	688540000	696880000	941470000	819280000
sp|Q9H4G	EPB41L1	sp|Q9H4G	1	8	8	7	15	98.502	472890000	551960000	488610000	428790000	392850000	449580000
sp|Q9H4G	GLIPR2	sp|Q9H4G	1	2	2	2	20	17.218	119940000	170290000	0	111500000	150000000	133450000
sp|Q9H4H	FAM83D	sp|Q9H4H	1	4	3	3	10	64.424	51031000	0	46702000	57847000	81154000	0
sp|Q9H4I3	TRABD	sp|Q9H4I3	1	1	1	1	5.9	42.321	0	0	0	0	0	29282000
sp|Q9H4L	SENP3	sp|Q9H4L	1	5	5	5	14	65.009	374330000	532780000	0	548140000	174260000	0
sp|Q9H4L	OSBPL3	sp|Q9H4L	1	1	1	1	1.5	101.22	0	0	0	0	0	0
sp|Q9H4M	EHD1	sp|Q9H4M	1	20	20	17	50	60.626	2128200000	2475200000	2145700000	2152300000	2260100000	2365500000
sp|Q9H4Z	PCIF1	sp|Q9H4Z	1	1	1	1	2.3	80.669	0	0	0	0	0	39896000
sp|Q9H55	ALG2	sp|Q9H55	1	2	2	2	7.7	47.091	0	0	0	0	67543000	0

sp|Q9H58	HEATR1	sp|Q9H58	1	22	22	22	13	242.37	1402000000	1175500000	1517100000	1331100000	1024900000	1002600000
sp|Q9H5Q	TFB2M	sp|Q9H5Q	1	2	2	2	6.8	45.348	0	111940000	0	0	94739000	145880000
sp|Q9H5V	CDCP1	sp|Q9H5V	1	3	3	3	3.2	92.931	53939000	56061000	64634000	62548000	65879000	79217000
sp|Q9H5V	CXorf56	sp|Q9H5V	1	1	1	1	5.9	25.624	0	0	0	0	0	23207000
sp|Q9H6E	TUT1	sp|Q9H6E	1	2	2	2	4.9	93.846	0	61333000	0	0	0	60618000
sp|Q9H6F	CCDC86	sp|Q9H6F	1	6	6	6	26	40.235	448730000	347570000	373690000	0	0	408410000
sp|Q9H6R	NOL6	sp|Q9H6R	1	17	17	17	21	127.59	576040000	707360000	723950000	642180000	888050000	699210000
sp|Q9H6S	YTHDC2	sp|Q9H6S	1	11	11	11	13	160.25	167120000	255820000	220320000	249940000	239780000	168070000
sp|Q9H6S	EPS8L2	sp|Q9H6S	1	2	2	2	4.3	80.62	161020000	123570000	168090000	121750000	148120000	114450000
sp|Q9H6T	RPAP3	sp|Q9H6T	1	7	7	7	16	75.718	343100000	462120000	401140000	376250000	319830000	301440000
sp|Q9H6V	LDAH	sp|Q9H6V	1	2	2	2	7.4	37.318	0	0	69872000	0	0	0
sp|Q9H6Y	WDR55	sp|Q9H6Y	1	4	4	4	17	42.07	178580000	215250000	0	0	221610000	213770000
sp|Q9H6Z	RANBP3	sp|Q9H6Z	1	5	5	5	12	60.209	548360000	408850000	408690000	398860000	268380000	470870000
sp|Q9H77	DCTPP1	sp|Q9H77	1	9	9	9	58	18.681	1397600000	1529200000	1394700000	1296200000	1755000000	1495600000
sp|Q9H78	SH2D4A	sp|Q9H78	1	8	8	8	23	52.726	413410000	129770000	0	158660000	0	239430000
sp|Q9H7B	RPF2	sp|Q9H7B	1	5	5	5	21	35.582	375850000	461200000	392120000	458500000	427160000	334770000
sp|Q9H7B	SMYD3	sp|Q9H7B	1	3	3	3	9.8	49.097	0	59094000	46547000	65898000	35046000	0
sp|Q9H7D	DOCK5	sp|Q9H7D	1	3	3	3	1.9	215.31	0	93001000	92680000	0	0	0
sp|Q9H7D	WDR26	sp|Q9H7D	1	1	1	1	3	72.123	0	0	0	0	30784000	0
sp|Q9H7E	C8orf33	sp|Q9H7E	1	4	4	4	31	24.992	698430000	478890000	540850000	779620000	557260000	554430000
sp|Q9H7N	SCAF1	sp|Q9H7N	1	2	2	2	2	139.27	0	0	0	0	29117000	0
sp|Q9H7Z	PTGES2	sp|Q9H7Z	1	5	5	5	18	41.943	570540000	583210000	476900000	439740000	471080000	379340000
sp|Q9H81	PHAX	sp|Q9H81	1	4	4	4	13	44.402	78115000	81547000	93989000	0	0	0
sp|Q9H83	UBE2Z	sp|Q9H83	1	8	8	8	23	38.21	592980000	501310000	617160000	497620000	363550000	442070000
sp|Q9H84	GEMIN7	sp|Q9H84	1	1	1	1	9.9	14.536	0	0	0	0	0	28538000
sp|Q9H84	ACAD9	sp|Q9H84	1	11	11	11	24	68.76	823440000	1112000000	1114300000	1143100000	1134600000	1208600000
sp|Q9H85	NT5DC2	sp|Q9H85	1	2	2	2	3.7	60.718	37433000	0	0	42919000	0	0
sp|Q9H87	RMND5A	sp|Q9H87	1	1	1	1	3.3	43.992	0	0	24300000	0	0	0
sp|Q9H8H	NOL11	sp|Q9H8H	1	8	8	8	18	81.123	307230000	328120000	324010000	284730000	322530000	292460000
sp|Q9H8H	DDX31	sp|Q9H8H	1	4	4	4	6.2	94.086	136280000	110400000	92788000	0	0	0
sp|Q9H8H	METTL7A	sp|Q9H8H	1	2	2	2	11	28.319	0	0	0	0	0	65148000
sp|Q9H8M	MINDY3	sp|Q9H8M	1	1	1	1	3.4	49.724	0	0	0	0	19322000	0
sp|Q9H8S	MOB1A	sp|Q9H8S	2	6	6	6	29	25.079	422390000	614230000	437320000	368920000	503500000	385890000
sp|Q9H8Y	ANKZF1	sp|Q9H8Y	1	4	4	4	8.4	80.926	66558000	0	0	93470000	104090000	0
sp|Q9H8Y	GORASP2	sp|Q9H8Y	1	3	3	3	8	47.145	453800000	419970000	382010000	361030000	503930000	566900000
sp|Q9H91	JPT2	sp|Q9H91	1	8	8	8	62	20.063	1233300000	1294600000	1398000000	1395600000	1489000000	1086900000
sp|Q9H93	SP140L	sp|Q9H93	1	2	1	1	3.3	67.005	0	0	0	113150000	0	0
sp|Q9H93	SLC25A22	sp|Q9H93	2	4	4	4	14	34.47	239140000	0	0	250450000	272750000	368020000
sp|Q9H94	MED20	sp|Q9H94	1	2	2	2	12	23.222	0	0	0	0	0	96260000

sp|Q9H97	QTRT2	sp|Q9H97	1	2	2	2	8.2	46.712	0	0	0	0	0	15721000
sp|Q9H99	ARMT1	sp|Q9H99	1	3	3	3	13	51.172	340280000	420350000	320050000	432770000	306050000	568790000
sp|Q9H9A	CNOT10	sp|Q9H9A	1	3	3	3	6	82.309	172790000	0	0	0	0	73574000
sp|Q9H9A	LRRC40	sp|Q9H9A	1	16	16	16	38	68.249	754780000	840890000	751370000	713540000	660050000	672970000
sp|Q9H9B	SFXN1	sp|Q9H9B	1	12	12	11	56	35.619	2477800000	2768700000	2229100000	3024700000	3240200000	3153000000
sp|Q9H9C	VIPAS39	sp|Q9H9C	1	1	1	1	4.3	57.005	0	0	0	0	0	11150000
sp|Q9H9F	ACTR5	sp|Q9H9F	1	2	2	2	4.8	68.297	0	0	0	0	0	54586000
sp|Q9H9H	VPS37B	sp|Q9H9H	1	4	4	4	25	31.307	129390000	129950000	0	140750000	117470000	108970000
sp|Q9H9J	MRPL44	sp|Q9H9J	1	8	8	8	39	37.535	450500000	389330000	376000000	421620000	368260000	370640000
sp|Q9H9P	L2HGDH	sp|Q9H9P	1	3	3	3	8.4	50.315	79207000	0	72136000	55597000	83471000	0
sp|Q9H9Q	COPS7B	sp|Q9H9Q	1	3	3	3	16	29.622	147410000	145220000	138280000	164220000	166130000	0
sp|Q9H9T	ELP3	sp|Q9H9T	1	4	4	4	12	62.258	117170000	135380000	127010000	125930000	98329000	107720000
sp|Q9H9Y	POLR1B	sp|Q9H9Y	1	3	3	3	3.1	128.23	71792000	75405000	65215000	65387000	64620000	0
sp|Q9HA7	CARS2	sp|Q9HA7	1	2	2	2	5.1	62.223	0	0	0	0	0	311350000
sp|Q9HAB	PPCS	sp|Q9HAB	1	2	2	2	6.8	34.005	145130000	0	104240000	110190000	0	0
sp|Q9HAD	WDR41	sp|Q9HAD	1	1	1	1	4.8	51.727	47726000	0	44431000	37572000	39303000	0
sp|Q9HAK	EBF2	sp|Q9HAK	1	1	1	1	1.9	62.649	0	0	0	0	0	748090000
sp|Q9HAU	PLEKHA5	sp|Q9HAU	1	3	3	3	3.6	127.46	0	0	0	0	0	46051000
sp|Q9HAU	UPF2	sp|Q9HAU	1	3	3	3	3.2	147.81	0	75386000	0	73080000	73462000	0
sp|Q9HAV	GNB4	sp|Q9HAV	2	8	3	3	34	37.567	0	0	216560000	245290000	0	0
sp|Q9HAV	XPO5	sp|Q9HAV	1	15	15	15	15	136.31	709460000	679500000	615380000	706580000	762840000	778050000
sp|Q9HAV	GRPEL1	sp|Q9HAV	1	7	7	7	38	24.279	1064800000	1455200000	1059700000	1200100000	1676000000	1750300000
sp|Q9HB0	C12orf10	sp|Q9HB0	1	11	11	11	40	42.449	1506600000	1622800000	1564300000	1578800000	1523600000	1327600000
sp|Q9HB1	PLEKHA2	sp|Q9HB1	1	6	6	6	21	47.254	168280000	209720000	146210000	169150000	185230000	231580000
sp|Q9HB4	SCPEP1	sp|Q9HB4	1	2	2	2	7.1	50.83	0	0	0	74864000	0	0
sp|Q9HB7	CACYBP	sp|Q9HB7	1	14	14	14	68	26.21	8399300000	8202800000	8555400000	8243900000	9057200000	8661400000
sp|Q9HBH	RDH14	sp|Q9HBH	1	2	2	2	8.6	36.864	0	0	76289000	0	0	0
sp|Q9HBI	PARVB	sp|Q9HBI	1	3	2	2	11	41.714	0	0	0	176810000	0	0
sp|Q9HBL	PLGRKT	sp|Q9HBL	1	2	2	2	16	17.201	55779000	0	0	71067000	101570000	0
sp|Q9HBM	SPC25	sp|Q9HBM	1	2	2	2	10	26.152	109530000	116850000	104270000	86739000	88680000	89274000
sp|Q9HC0	TMEM165	sp|Q9HC0	1	4	4	4	23	34.905	634030000	768470000	542270000	599870000	782810000	834590000
sp|Q9HC2	SLC25A1	sp|Q9HC2	1	2	2	2	6.2	35.511	52879000	48104000	36993000	62730000	58949000	52238000
sp|Q9HC3	EML4	sp|Q9HC3	1	9	9	9	12	108.91	326240000	309930000	327820000	326870000	285930000	380160000
sp|Q9HC3	MRM3	sp|Q9HC3	1	4	4	4	13	47.019	0	142020000	88012000	91004000	96683000	113580000
sp|Q9HC3	GLOD4	sp|Q9HC3	1	12	12	12	42	34.793	971880000	1371100000	1137100000	931120000	1157200000	949890000
sp|Q9HC5	CBX8	sp|Q9HC5	2	3	3	3	8.2	43.395	0	61422000	0	0	0	0
sp|Q9HC0	MCCC2	sp|Q9HC0	1	9	9	9	22	61.332	437350000	434120000	474340000	516440000	517610000	453560000
sp|Q9HCD	NCOA5	sp|Q9HCD	1	7	7	7	17	65.536	232860000	324660000	210260000	202410000	270500000	237180000
sp|Q9HCE	MOV10	sp|Q9HCE	1	9	9	9	12	113.67	263840000	252800000	235380000	232760000	237540000	333180000

sp|Q9HCN	GPN1	sp|Q9HCN	1	4	4	4	18	41.74	0	0	0	0	0	1010200000
sp|Q9HCN	SDF2L1	sp|Q9HCN	1	4	4	4	34	23.598	359850000	300340000	248380000	343040000	455260000	507980000
sp|Q9HCS	XAB2	sp|Q9HCS	1	8	8	8	11	100.01	253700000	299110000	215680000	283310000	0	268220000
sp|Q9HCU	PREB	sp|Q9HCU	1	3	3	3	15	45.468	123100000	0	0	0	87951000	141590000
sp|Q9HD1	SRA1	sp|Q9HD1	1	4	4	4	24	25.673	191460000	245480000	281440000	159180000	198810000	178630000
sp|Q9HD2	ATP13A1	sp|Q9HD2	1	8	8	8	9.1	132.95	265800000	249460000	162800000	269960000	214360000	286960000
sp|Q9HD2	GOPC	sp|Q9HD2	1	6	6	6	17	50.519	243940000	205720000	0	247700000	261900000	190430000
sp|Q9HD4	CHMP1A	sp|Q9HD4	1	2	2	2	8.7	21.703	0	68269000	77530000	61619000	0	0
sp|Q9HD4	TM9SF3	sp|Q9HD4	1	5	5	5	10	67.887	636890000	624500000	733320000	878810000	833930000	806700000
sp|Q9HD6	MYO10	sp|Q9HD6	1	4	4	4	3.2	237.34	206510000	161780000	178350000	147070000	154140000	159640000
sp|Q9HDC	APMAP	sp|Q9HDC	1	11	11	11	34	46.48	1509200000	1600800000	1564900000	1675800000	1343600000	1503500000
sp|Q9NNW	WDR6	sp|Q9NNW	1	4	4	4	7.4	121.72	107280000	101980000	120350000	121400000	94216000	108140000
sp|Q9NNW	TXNRD2	sp|Q9NNW	1	3	2	2	9.7	56.506	0	0	0	0	77248000	0
sp|Q9NP5	ABCB6	sp|Q9NP5	1	10	10	10	21	93.884	357550000	398980000	362010000	438620000	407780000	384100000
sp|Q9NP6	ARFGAP3	sp|Q9NP6	1	2	2	2	4.8	56.928	0	0	0	0	63104000	0
sp|Q9NP6	HMG20A	sp|Q9NP6	1	2	2	2	12	40.143	0	21307000	0	0	0	0
sp|Q9NP7	RAB18	sp|Q9NP7	1	8	8	8	52	22.977	1737300000	1214900000	1306400000	1520100000	1747200000	1440300000
sp|Q9NP7	ALG13	sp|Q9NP7	1	1	1	1	1.7	126.05	0	0	0	0	0	0
sp|Q9NP7	PALMD	sp|Q9NP7	1	4	4	4	14	62.757	152550000	193710000	157970000	140590000	167360000	149160000
sp|Q9NP7	SSU72	sp|Q9NP7	1	4	4	4	24	22.574	232550000	250520000	263220000	225820000	280720000	233560000
sp|Q9NP7	VTA1	sp|Q9NP7	1	7	7	7	31	33.879	1037300000	1127100000	1212500000	772530000	1178400000	987920000
sp|Q9NP8	SARS2	sp|Q9NP8	1	6	6	6	19	58.282	257690000	186280000	237480000	295420000	364160000	265970000
sp|Q9NP9	MRPS30	sp|Q9NP9	1	6	6	6	25	50.364	582950000	266500000	624860000	438010000	358410000	337880000
sp|Q9NP9	DYNLRB1	sp|Q9NP9	2	3	3	3	26	10.921	0	0	0	688600000	575150000	0
sp|Q9NPA	EMC7	sp|Q9NPA	1	6	6	6	48	26.47	209110000	265060000	328300000	349880000	202760000	176220000
sp|Q9NPA	ENY2	sp|Q9NPA	1	2	2	2	24	11.528	204820000	283460000	306280000	151660000	225350000	205850000
sp|Q9NPD	EXOSC4	sp|Q9NPD	1	6	6	6	29	26.383	324770000	196990000	0	419700000	559650000	334270000
sp|Q9NPD	UBE2T	sp|Q9NPD	1	5	5	5	37	22.521	366190000	409600000	412380000	209610000	285220000	289290000
sp|Q9NPE	NOP10	sp|Q9NPE	1	2	2	2	38	7.7059	288180000	243020000	199150000	190690000	0	224200000
sp|Q9NPF	OSGEP	sp|Q9NPF	1	4	4	4	19	36.426	291000000	0	365810000	316760000	289280000	340180000
sp|Q9NPF	DMAP1	sp|Q9NPF	1	2	2	2	6.4	52.992	0	25743000	0	0	0	0
sp|Q9NPI	BRD7	sp|Q9NPI	1	1	1	1	3.4	74.138	0	0	0	54957000	0	0
sp|Q9NPI	DCP1A	sp|Q9NPI	1	1	1	1	4.1	63.309	0	0	0	0	51935000	0
sp|Q9NPJ	ACOT13	sp|Q9NPJ	1	2	2	2	16	14.96	454600000	499730000	498170000	493590000	582670000	539360000
sp|Q9NPQ	RIC8A	sp|Q9NPQ	1	4	4	4	12	59.709	0	0	124540000	85620000	128060000	0
sp|Q9NQ2	LUC7L	sp|Q9NQ2	1	10	5	5	28	43.727	153330000	208920000	219920000	215000000	231840000	193680000
sp|Q9NQ4	LZTFL1	sp|Q9NQ4	1	1	1	1	4.3	34.592	0	0	0	0	0	35588000
sp|Q9NQ5	MRPL40	sp|Q9NQ5	1	2	2	2	17	24.49	264080000	277300000	336690000	289830000	316560000	255460000
sp|Q9NQ5	PPAN	sp|Q9NQ5	1	6	6	6	20	53.193	243040000	207270000	165600000	194740000	206720000	155080000

sp|Q9NQ8	TIGAR	sp|Q9NQ8	1	5	5	5	26	30.062	312840000	360980000	196180000	321260000	361750000	259530000
sp|Q9NQ9	COPRS	sp|Q9NQ9	1	2	2	2	19	20.066	0	0	0	0	24275000	0
sp|Q9NQC	RTN4	sp|Q9NQC	1	11	11	11	18	129.93	6506000000	7046400000	5409300000	7613700000	7142100000	6147500000
sp|Q9NQC	RPRD1B	sp|Q9NQC	1	6	5	5	32	36.899	185970000	195630000	171250000	240970000	189940000	392220000
sp|Q9NQF	PFDN4	sp|Q9NQF	1	2	2	2	20	15.314	318080000	0	0	0	0	0
sp|Q9NQF	NIT2	sp|Q9NQF	1	9	9	9	47	30.608	761720000	988050000	952320000	819410000	749290000	859800000
sp|Q9NQS	INCENP	sp|Q9NQS	1	2	2	2	3.3	105.43	82440000	65811000	0	0	0	0
sp|Q9NQT	EXOSC5	sp|Q9NQT	1	4	4	4	41	25.249	300670000	263900000	321220000	371010000	454490000	0
sp|Q9NQT	EXOSC3	sp|Q9NQT	1	7	7	7	46	29.572	575880000	455520000	462790000	341060000	384890000	510520000
sp|Q9NQW	ANLN	sp|Q9NQW	1	15	15	15	20	124.2	344270000	504710000	483950000	456320000	454320000	458080000
sp|Q9NQW	XPNPEP1	sp|Q9NQW	1	8	8	8	25	69.917	376760000	469780000	404080000	400230000	461090000	491860000
sp|Q9NQX	GPHN	sp|Q9NQX	1	3	3	3	6.9	79.748	93592000	134220000	0	0	100530000	0
sp|Q9NQZ	UTP3	sp|Q9NQZ	1	6	6	6	19	54.557	0	136150000	0	227330000	167540000	161920000
sp|Q9NR0	BIRC6	sp|Q9NR0	1	2	2	2	0.7	530.25	0	0	0	0	42623000	0
sp|Q9NR1	PDLIM7	sp|Q9NR1	1	17	17	17	48	49.844	3742100000	3247800000	2954400000	3207900000	3639300000	3331900000
sp|Q9NR2	DIABLO	sp|Q9NR2	1	3	3	3	12	27.131	308550000	340560000	449600000	333480000	269190000	311910000
sp|Q9NR3	DDX21	sp|Q9NR3	1	34	34	32	48	87.343	1.0338E+10	8784900000	8939300000	10901000000	10632000000	1.2393E+10
sp|Q9NR3	SAR1A	sp|Q9NR3	1	9	9	6	55	22.367	1418900000	1644000000	1477400000	1876900000	2157700000	1728800000
sp|Q9NR4	NANS	sp|Q9NR4	1	9	9	9	35	40.307	1189800000	1228500000	1410500000	1268900000	1403600000	1301100000
sp|Q9NR4	SH3GLB2	sp|Q9NR4	1	9	9	9	32	43.973	341310000	320900000	267830000	313590000	136820000	243010000
sp|Q9NR5	EIF2B3	sp|Q9NR5	1	7	7	7	25	50.24	391490000	276460000	484510000	384340000	396940000	300510000
sp|Q9NR5	MBNL1	sp|Q9NR5	1	4	4	2	14	41.817	797430000	668390000	896610000	515550000	843580000	789250000
sp|Q9NRF	CTPS2	sp|Q9NRF	1	5	4	4	11	65.677	158340000	153720000	0	167180000	0	0
sp|Q9NRF	POLE3	sp|Q9NRF	1	3	3	3	24	16.859	340510000	454410000	553930000	555190000	418180000	301500000
sp|Q9NRG	CHRAC1	sp|Q9NRG	1	3	3	3	25	14.71	154800000	113420000	140450000	172100000	219320000	0
sp|Q9NRG	SDR39U1	sp|Q9NRG	1	1	1	1	9.4	34.747	0	0	0	0	0	16186000
sp|Q9NRG	AAAS	sp|Q9NRG	1	7	7	7	19	59.573	292510000	354410000	328180000	382420000	410290000	448340000
sp|Q9NRL	BAZ1A	sp|Q9NRL	1	5	5	5	5.7	178.7	128010000	80930000	97850000	107460000	84133000	128590000
sp|Q9NRL	STRN4	sp|Q9NRL	1	3	3	3	9	80.595	0	0	132620000	0	0	0
sp|Q9NRN	AASDHPP	sp|Q9NRN	1	5	5	5	21	35.776	113400000	116540000	106440000	166490000	161990000	125920000
sp|Q9NRP	OSTC	sp|Q9NRP	1	1	1	1	8.1	16.829	0	0	0	0	0	310270000
sp|Q9NRR	UBQLN4	sp|Q9NRR	1	10	7	7	26	63.852	547590000	362530000	430460000	573470000	416410000	195880000
sp|Q9NRV	HEBP1	sp|Q9NRV	1	4	4	4	24	21.097	400010000	499910000	396260000	488100000	447170000	303680000
sp|Q9NRW	VPS45	sp|Q9NRW	1	4	4	4	11	65.076	0	116610000	180770000	0	0	102660000
sp|Q9NRX	PNO1	sp|Q9NRX	1	4	4	4	20	27.924	0	0	0	0	71735000	0
sp|Q9NRX	MRPL17	sp|Q9NRX	1	5	5	5	31	20.05	204640000	264660000	200050000	198530000	282840000	161250000
sp|Q9NRX	PHPT1	sp|Q9NRX	1	4	4	4	51	13.832	1077900000	1234800000	836070000	1172300000	1432000000	1066500000
sp|Q9NRY	FAM114A	sp|Q9NRY	1	3	3	3	11	55.468	62239000	56656000	55195000	92390000	74291000	74377000
sp|Q9NRY	PLSCR3	sp|Q9NRY	1	1	1	1	6.8	31.648	0	0	0	0	0	11430000

sp|Q9NRZ	HELLS	sp|Q9NRZ	1	10	10	10	14	97.073	333830000	335590000	520930000	167210000	228080000	433260000
sp|Q9NS6	TOMM22	sp|Q9NS6	1	6	6	6	49	15.521	1500700000	1157900000	1133500000	1220300000	1487500000	1371100000
sp|Q9NS8	LANCL2	sp|Q9NS8	1	5	5	5	16	50.854	192970000	197970000	198490000	290170000	222870000	132940000
sp|Q9NS8	KIF15	sp|Q9NS8	1	6	6	6	4.9	160.16	0	116480000	0	0	78519000	0
sp|Q9NS9	RAD18	sp|Q9NS9	1	1	1	1	3.2	56.222	0	0	0	0	0	33924000
sp|Q9NSD	FARSB	sp|Q9NSD	1	19	19	19	37	66.115	2501400000	2890200000	2712900000	2501600000	2482200000	2784500000
sp|Q9NSE	IARS2	sp|Q9NSE	1	19	19	19	27	113.79	1096800000	860510000	1082800000	1247300000	1223600000	1014300000
sp|Q9NSI	FAM207A	sp|Q9NSI	1	3	3	3	22	25.456	69271000	67964000	67083000	57046000	72244000	75080000
sp|Q9NSK	KLC4	sp|Q9NSK	1	7	2	2	13	68.639	68327000	91856000	97442000	0	0	0
sp|Q9NSF	CENPM	sp|Q9NSF	1	1	1	1	6.1	19.737	0	0	0	0	0	0
sp|Q9NSV	DIAPH3	sp|Q9NSV	1	5	5	5	6.1	136.92	151680000	197680000	98504000	0	120750000	96585000
sp|Q9NT6	ATG3	sp|Q9NT6	1	6	6	6	30	35.864	384260000	395130000	355030000	395330000	416650000	365920000
sp|Q9NTI	PDS5B	sp|Q9NTI	1	14	12	12	12	164.67	556470000	466240000	600640000	579490000	582230000	622740000
sp|Q9NTJ	SMC4	sp|Q9NTJ	1	33	33	33	29	147.18	2988400000	3143400000	3169400000	2774500000	3147400000	3058400000
sp|Q9NTJ	SACM1L	sp|Q9NTJ	1	5	5	5	9.2	66.966	209260000	279490000	252340000	312670000	317670000	349080000
sp|Q9NTK	OLA1	sp|Q9NTK	1	14	14	14	43	44.743	2827100000	2271600000	2782500000	2736700000	2929100000	2489500000
sp|Q9NTM	CUTC	sp|Q9NTM	1	2	2	2	12	29.341	0	142760000	104560000	95985000	100620000	149940000
sp|Q9NTX	ECHDC1	sp|Q9NTX	1	6	6	6	27	33.698	369210000	334060000	424740000	334380000	346910000	497670000
sp|Q9NTZ	RBM12	sp|Q9NTZ	1	11	11	11	14	97.394	659590000	753900000	602190000	511000000	524550000	674920000
sp|Q9NU2	MDN1	sp|Q9NU2	1	11	11	11	3.1	632.81	200010000	176130000	181100000	207510000	201130000	180690000
sp|Q9NUI	DECR2	sp|Q9NUI	1	2	2	2	9.6	30.777	0	0	0	0	34865000	0
sp|Q9NUJ	ABHD10	sp|Q9NUJ	1	4	4	4	18	33.932	265680000	309850000	223360000	298760000	329340000	454890000
sp|Q9NUJ	TCP11L1	sp|Q9NUJ	1	1	1	1	2.4	57.034	0	0	0	0	0	17596000
sp|Q9NUL	STAU2	sp|Q9NUL	1	3	3	3	7.4	62.608	0	160760000	301420000	157230000	0	0
sp|Q9NUM	TMEM106	sp|Q9NUM	1	3	3	3	11	31.127	0	211530000	0	0	0	0
sp|Q9NUC	TXLNG	sp|Q9NUC	2	4	4	4	11	60.585	0	154730000	0	0	0	0
sp|Q9NUC	SPATS2L	sp|Q9NUC	1	8	8	8	17	61.728	553180000	483980000	353420000	336320000	534800000	485790000
sp|Q9NUC	UFSP2	sp|Q9NUC	1	1	1	1	6	53.261	0	0	0	0	0	75195000
sp|Q9NUC	ABCF3	sp|Q9NUC	1	9	9	9	19	79.744	369660000	297390000	385740000	323210000	307840000	310700000
sp|Q9NUC	FAM49B	sp|Q9NUC	2	9	9	9	44	36.748	1053500000	1084100000	1223000000	1034000000	1196300000	1078100000
sp|Q9NUU	DDX19A	sp|Q9NUU	1	11	11	3	29	53.974	1116600000	1232100000	1056300000	1204400000	1252300000	1185200000
sp|Q9NV0	DCAF13	sp|Q9NV0	1	3	3	3	8.3	51.402	98143000	110260000	121770000	130640000	0	0
sp|Q9NV3	IMP3	sp|Q9NV3	1	2	2	2	15	21.85	156280000	187900000	170720000	161860000	185010000	185370000
sp|Q9NV5	MRGBP	sp|Q9NV5	1	1	1	1	6.4	22.417	0	0	0	0	0	26142000
sp|Q9NV9	TMEM30A	sp|Q9NV9	1	1	1	1	4.7	40.683	0	0	0	0	0	26796000
sp|Q9NVA	UQCC1	sp|Q9NVA	1	1	1	1	3	34.6	0	0	0	0	0	19649000
sp|Q9NVA	SEPT11	sp|Q9NVA	1	14	14	8	40	49.398	2692300000	2472700000	2426300000	1992500000	2222600000	1986500000
sp|Q9NVD	PARVA	sp|Q9NVD	1	8	8	7	32	42.243	644080000	534780000	676730000	517730000	440080000	451590000
sp|Q9NVE	PANK4	sp|Q9NVE	1	4	4	4	7.8	85.99	0	0	0	0	123840000	0

sp|Q9NVC	TBC1D13	sp|Q9NVC	1	5	5	5	16	46.553	290720000	319800000	313420000	276680000	322420000	278520000
sp|Q9NVH	DNAJC11	sp|Q9NVH	1	8	8	8	19	63.277	355450000	527720000	422140000	469540000	538520000	465730000
sp|Q9NVH	INTS7	sp|Q9NVH	1	7	7	7	12	106.83	1197800000	1649800000	1381400000	1268000000	1517100000	1441000000
sp|Q9NVI	FANCI	sp|Q9NVI	1	10	10	10	9.2	149.32	880220000	1177800000	1117800000	779350000	674610000	704830000
sp|Q9NVI	ATAD3A	sp|Q9NVI	1	22	22	7	36	71.368	4070700000	4023200000	4076900000	4691000000	5135500000	4800900000
sp|Q9NVJ	ARL8B	sp|Q9NVJ	1	5	5	2	36	21.539	528990000	632550000	720520000	508310000	512850000	521880000
sp|Q9NVM	INTS13	sp|Q9NVM	1	1	1	1	1.8	80.224	14982000	0	0	0	0	0
sp|Q9NVN	GNL3L	sp|Q9NVN	1	2	2	2	4.3	65.572	142520000	182070000	0	0	0	144630000
sp|Q9NVP	DDX18	sp|Q9NVP	1	12	12	12	25	75.406	979990000	935930000	1040300000	1422000000	1141800000	1220600000
sp|Q9NVP	ASF1B	sp|Q9NVP	1	4	4	3	33	22.433	199280000	177610000	166930000	112970000	124270000	168550000
sp|Q9NVC	FAIM	sp|Q9NVC	1	4	4	4	39	20.215	0	238070000	0	0	0	0
sp|Q9NVS	MRPS18A	sp|Q9NVS	1	3	3	3	18	22.184	0	0	0	0	0	191440000
sp|Q9NVS	PNPO	sp|Q9NVS	1	4	4	4	24	29.988	593380000	260450000	0	516400000	486980000	450480000
sp|Q9NVU	SDAD1	sp|Q9NVU	1	2	2	2	3.6	79.87	0	0	0	67746000	0	0
sp|Q9NVV	MTPAP	sp|Q9NVV	1	4	4	4	11	66.171	0	108330000	93286000	0	96198000	120760000
sp|Q9NVX	NLE1	sp|Q9NVX	1	8	8	8	26	53.32	352480000	548190000	321120000	279550000	405420000	385440000
sp|Q9NVZ	NECAP2	sp|Q9NVZ	1	3	3	3	23	28.338	276660000	224470000	230420000	167580000	234470000	230010000
sp|Q9NW1	RBM28	sp|Q9NW1	1	10	10	10	15	85.737	353350000	305970000	281100000	318190000	288900000	313520000
sp|Q9NW1	ANO10	sp|Q9NW1	1	5	5	5	8.9	76.328	253060000	301690000	232300000	242890000	248770000	326950000
sp|Q9NW6	RBM22	sp|Q9NW6	1	5	5	5	15	46.895	164350000	144920000	207970000	216360000	174580000	165190000
sp|Q9NW6	BSDC1	sp|Q9NW6	1	2	2	2	10	47.163	0	67032000	45449000	0	58748000	0
sp|Q9NW8	DMAC2	sp|Q9NW8	1	2	2	2	12	29.267	0	83951000	54254000	52393000	0	0
sp|Q9NW8	WDR70	sp|Q9NW8	1	6	6	6	11	73.2	246460000	276610000	0	0	0	0
sp|Q9NWI	ARGLU1	sp|Q9NWI	1	2	2	2	5.5	33.216	0	0	0	0	2305500000	3904400000
sp|Q9NWI	SLTM	sp|Q9NWI	1	8	8	8	9.8	117.15	348880000	297150000	382720000	583200000	386240000	0
sp|Q9NWI	FKBP14	sp|Q9NWI	1	1	1	1	7.1	24.171	0	0	0	0	30952000	0
sp|Q9NWS	PIH1D1	sp|Q9NWS	1	4	4	4	21	32.363	134890000	151980000	0	0	302560000	224990000
sp|Q9NWS	RMND1	sp|Q9NWS	1	1	1	1	1.8	51.603	0	0	0	0	0	11581000
sp|Q9NWT	PAK1IP1	sp|Q9NWT	1	2	2	2	7.9	43.963	0	0	135720000	0	0	0
sp|Q9NWU	OXSM	sp|Q9NWU	1	3	3	3	14	48.842	108450000	107490000	111640000	106410000	112280000	0
sp|Q9NWU	GID8	sp|Q9NWU	1	1	1	1	9.2	26.748	0	0	0	0	0	27339000
sp|Q9NWU	MRPL22	sp|Q9NWU	1	4	4	4	25	23.64	204120000	244250000	209120000	206190000	233810000	233200000
sp|Q9NWV	C1orf123	sp|Q9NWV	1	4	4	4	31	18.048	243540000	243870000	209650000	278730000	298170000	215010000
sp|Q9NWV	BABAM1	sp|Q9NWV	1	3	3	3	18	36.56	113040000	104040000	153760000	157300000	152000000	132340000
sp|Q9NWV	CLN6	sp|Q9NWV	1	1	1	1	6.8	35.919	0	0	0	0	0	47261000
sp|Q9NWX	THG1L	sp|Q9NWX	1	3	3	3	15	34.83	141860000	0	78175000	0	144740000	116670000
sp|Q9NX0	COMMD8	sp|Q9NX0	1	2	2	2	21	21.09	172270000	194730000	0	164650000	258400000	120220000
sp|Q9NX1	NDUFB11	sp|Q9NX1	1	3	3	3	33	17.316	611940000	428460000	483650000	370280000	428200000	421210000
sp|Q9NX1	SDHAF2	sp|Q9NX1	1	4	4	4	36	19.599	218440000	220550000	258850000	270300000	267080000	249940000

sp|Q9NX2	MRPL16	sp|Q9NX2	1	3	3	3	16	28.449	192820000	220700000	128420000	113860000	181270000	96101000
sp|Q9NX2	NHP2	sp|Q9NX2	1	6	6	6	54	17.201	586710000	684050000	601910000	546790000	795080000	899060000
sp|Q9NX4	OCIAD1	sp|Q9NX4	1	7	7	7	28	27.626	528950000	830380000	694440000	583490000	655240000	511600000
sp|Q9NX4	ADPRHL2	sp|Q9NX4	1	3	3	3	13	38.946	0	0	0	0	107840000	0
sp|Q9NX4	MARCH5	sp|Q9NX4	1	2	2	2	10	31.231	0	0	0	0	0	180970000
sp|Q9NX5	HYPK	sp|Q9NX5	1	3	3	3	36	14.665	629560000	547520000	424620000	505930000	475990000	487970000
sp|Q9NX5	LYAR	sp|Q9NX5	1	8	8	8	33	43.614	517080000	535960000	845070000	464010000	688480000	0
sp|Q9NX6	IMPAD1	sp|Q9NX6	1	4	4	4	13	38.681	0	86910000	148750000	0	136760000	0
sp|Q9NX6	CHCHD3	sp|Q9NX6	1	7	7	7	29	26.152	1890800000	1828600000	1585200000	1856900000	1729900000	1897600000
sp|Q9NX7	DUS2	sp|Q9NX7	1	2	2	2	6.1	55.05	0	0	0	0	0	21545000
sp|Q9NX7	CMTM6	sp|Q9NX7	1	1	1	1	5.5	20.419	0	0	0	0	0	92160000
sp|Q9NXE	SMPD4	sp|Q9NXE	1	3	3	3	5.3	97.809	200090000	0	168080000	134960000	188890000	0
sp|Q9NXF	TEX10	sp|Q9NXF	1	7	7	7	10	105.67	217060000	242690000	221880000	302680000	231110000	269520000
sp|Q9NXG	THUMPD1	sp|Q9NXG	1	9	9	9	38	39.315	685320000	626870000	881550000	593430000	677780000	597900000
sp|Q9NXH	TOR4A	sp|Q9NXH	1	2	2	2	7.1	46.914	0	0	0	0	0	52735000
sp|Q9NXH	TRMT1	sp|Q9NXH	1	13	13	13	29	72.233	629640000	769460000	738580000	777020000	799600000	622040000
sp|Q9NXV	CDKN2AII	sp|Q9NXV	1	3	3	3	6.9	61.124	268960000	159620000	108990000	162070000	0	0
sp|Q9NXW	DNAJB12	sp|Q9NXW	1	5	5	5	15	41.818	174080000	139510000	142690000	161530000	194740000	166240000
sp|Q9NY1	GAR1	sp|Q9NY1	1	5	5	5	28	22.348	501710000	454440000	465710000	482910000	616920000	537940000
sp|Q9NY2	PPP4R2	sp|Q9NY2	1	7	7	7	24	46.898	249770000	390960000	331170000	345490000	353740000	299570000
sp|Q9NY3	DPP3	sp|Q9NY3	1	14	14	14	28	82.588	848280000	981510000	804150000	1012600000	1026100000	949740000
sp|Q9NY6	AATF	sp|Q9NY6	1	4	4	4	12	63.132	165640000	119780000	95451000	127520000	92205000	146960000
sp|Q9NY9	DDX56	sp|Q9NY9	1	5	5	5	11	61.589	322590000	446080000	0	312240000	313160000	406310000
sp|Q9NYB	TERF2IP	sp|Q9NYB	1	1	1	1	4.3	44.259	0	0	0	0	0	26039000
sp|Q9NYB	ABI2	sp|Q9NYB	1	2	2	2	5.8	55.663	0	0	0	0	63111000	0
sp|Q9NYF	BCLAF1	sp|Q9NYF	1	14	13	13	18	106.12	2424700000	1936700000	1966400000	2007600000	2194100000	1854000000
sp|Q9NYH	UTP6	sp|Q9NYH	1	7	7	7	15	70.193	279090000	294690000	334360000	364310000	290280000	253410000
sp|Q9NYJ	COA4	sp|Q9NYJ	1	2	2	2	25	10.134	159790000	195980000	134100000	187300000	249080000	258290000
sp|Q9NYK	MRPL39	sp|Q9NYK	1	5	5	5	15	38.711	300120000	0	248880000	238470000	236570000	268240000
sp|Q9NYL	MAP3K20	sp|Q9NYL	1	2	2	2	4.1	91.154	0	0	0	0	38167000	0
sp|Q9NYL	FKBP11	sp|Q9NYL	1	3	3	3	18	22.18	131450000	216640000	0	234150000	0	0
sp|Q9NYL	TMOD3	sp|Q9NYL	2	17	17	17	56	39.594	2190600000	2741500000	1875600000	1999300000	2058400000	1785800000
sp|Q9NYP	ELOVL5	sp|Q9NYP	1	2	2	2	7.4	35.293	96251000	94599000	156250000	73641000	88226000	106120000
sp|Q9NYU	UGGT1	sp|Q9NYU	1	25	25	24	22	177.19	994790000	1140800000	1195000000	1227900000	1196800000	1391800000
sp|Q9NYY	FASTKD2	sp|Q9NYY	1	6	6	6	12	81.462	226070000	191950000	141210000	193390000	144020000	252810000
sp|Q9NZ0	TECR	sp|Q9NZ0	1	9	9	9	23	36.034	2676000000	2489000000	2618200000	3283300000	2594300000	3225000000
sp|Q9NZ0	ERAP1	sp|Q9NZ0	1	6	6	6	13	107.23	96263000	0	105620000	134740000	107540000	103150000
sp|Q9NZ0	UBAP1	sp|Q9NZ0	1	3	3	3	12	55.083	62488000	0	50722000	148010000	0	0
sp|Q9NZ3	ACTR10	sp|Q9NZ3	1	3	3	3	9.4	46.306	0	191520000	117540000	129780000	149920000	0

sp|Q9NZ4	PSENEN	sp|Q9NZ4	1	1	1	1	15	12.029	0	0	0	0	0	65146000
sp|Q9NZ4	USE1	sp|Q9NZ4	1	1	1	1	6.2	29.371	0	0	23699000	0	0	0
sp|Q9NZ4	CISD1	sp|Q9NZ4	1	4	4	4	44	12.199	805640000	757060000	889390000	854000000	945230000	976410000
sp|Q9NZ6	C9orf78	sp|Q9NZ6	1	6	6	6	27	33.688	504350000	643410000	532140000	459240000	545060000	692790000
sp|Q9NZB	FAM120A	sp|Q9NZB	3	28	28	28	35	121.89	3727500000	3239900000	3374800000	2719700000	3047700000	3369500000
sp|Q9NZC	GDE1	sp|Q9NZC	1	1	1	1	4.5	37.718	0	0	0	0	0	61165000
sp|Q9NZD	GLTP	sp|Q9NZD	1	1	1	1	7.2	23.85	0	0	0	0	0	2657000
sp|Q9NZD	SPG21	sp|Q9NZD	1	6	6	6	25	34.96	214570000	257120000	293140000	286270000	171630000	196700000
sp|Q9NZI8	IGF2BP1	sp|Q9NZI8	3	16	14	14	34	63.48	1549700000	1613500000	1296600000	1685600000	1874100000	2120600000
sp|Q9NZJ	COQ3	sp|Q9NZJ	1	1	1	1	4.6	41.054	0	0	0	0	0	49826000
sp|Q9NZJ	MTCH1	sp|Q9NZJ	1	5	5	5	17	41.544	315700000	295040000	532000000	364770000	301650000	316680000
sp|Q9NZL	HSPBP1	sp|Q9NZL	1	9	9	9	34	39.474	515030000	599370000	680890000	538630000	521820000	667260000
sp|Q9NZL	MAT2B	sp|Q9NZL	1	13	13	13	48	37.551	1007600000	1305100000	1038900000	882170000	955910000	1135800000
sp|Q9NZM	MYOF	sp|Q9NZM	3	73	73	73	44	234.71	9199500000	8647100000	8692900000	8586700000	8774400000	9024900000
sp|Q9NZM	NOP53	sp|Q9NZM	1	4	4	4	18	54.389	57486000	0	75627000	82150000	0	0
sp|Q9NZN	EHD2	sp|Q9NZN	1	20	19	18	58	61.161	3565100000	3724000000	3810300000	4109900000	2931300000	3298200000
sp|Q9NZQ	NCKIPSD	sp|Q9NZQ	1	4	4	4	7.5	78.959	91425000	86781000	0	0	0	0
sp|Q9NZT	OGFR	sp|Q9NZT	1	11	11	11	37	73.324	485020000	359330000	416910000	419670000	456230000	419150000
sp|Q9NZV	MPP6	sp|Q9NZV	1	2	2	2	4.3	61.116	0	0	0	0	84851000	0
sp|Q9NZZ	CHMP5	sp|Q9NZZ	1	3	3	3	24	24.57	538610000	596690000	477830000	406930000	437740000	459360000
sp|Q9P00	COMMD9	sp|Q9P00	1	6	6	6	48	21.819	261550000	354670000	422060000	256220000	247100000	161650000
sp|Q9P00	CNIH4	sp|Q9P00	1	1	1	1	14	16.093	337390000	0	0	454030000	0	0
sp|Q9P01	CWC15	sp|Q9P01	1	2	2	2	15	26.624	138530000	130880000	122220000	89906000	140700000	105510000
sp|Q9P01	MRPL15	sp|Q9P01	1	2	2	2	8.1	33.419	0	0	0	0	0	48451000
sp|Q9P01	THYN1	sp|Q9P01	1	3	3	3	12	25.697	72787000	132220000	128980000	112170000	147100000	145210000
sp|Q9P02	CRIPT	sp|Q9P02	1	1	1	1	11	11.216	0	0	0	0	0	36897000
sp|Q9P03	HACD3	sp|Q9P03	1	6	6	6	20	43.159	1407800000	1335700000	1450200000	1567700000	1275700000	1779100000
sp|Q9P0I2	EMC3	sp|Q9P0I2	1	6	6	6	29	29.952	215310000	213430000	215550000	286500000	257550000	205930000
sp|Q9P0J	NDUFA13	sp|Q9P0J	1	3	3	3	16	16.698	130400000	249620000	197850000	124850000	98252000	82829000
sp|Q9P0J	KCMF1	sp|Q9P0J	1	2	2	2	7.3	41.945	0	0	0	110680000	45840000	0
sp|Q9P0K	RAI14	sp|Q9P0K	1	12	12	12	20	110.04	476590000	336030000	0	406440000	438200000	314080000
sp|Q9P0L	VAPA	sp|Q9P0L	1	12	12	11	53	27.893	5157400000	5325400000	4536600000	5710200000	7311900000	5744000000
sp|Q9P0M	MRPL27	sp|Q9P0M	1	3	3	3	28	16.073	0	0	0	0	0	130120000
sp|Q9P0P	RNF181	sp|Q9P0P	1	1	1	1	5.9	17.909	0	0	0	0	29001000	0
sp|Q9P0S	TMEM14C	sp|Q9P0S	1	1	1	1	8.9	11.564	0	0	0	0	0	382440000
sp|Q9P0T	TMEM9	sp|Q9P0T	1	2	2	2	18	20.574	0	0	0	0	0	209520000
sp|Q9P0U	TOMM7	sp|Q9P0U	1	2	2	2	44	6.2484	0	0	0	302910000	535410000	0
sp|Q9P0V	SH3BP4	sp|Q9P0V	1	4	4	4	6	107.49	0	0	0	0	61814000	0
sp|Q9P0V	SEPT10	sp|Q9P0V	1	10	9	9	36	52.592	601180000	518690000	495970000	538070000	498170000	522750000

sp|Q9P0W	HMG20B	sp|Q9P0W	1	1	1	1	7.6	35.812	0	0	0	0	0	6253700
sp|Q9P1F	ABRACL	sp|Q9P1F	1	2	2	2	36	9.0564	218200000	347970000	357280000	246190000	260840000	0
sp|Q9P25	VPS18	sp|Q9P25	1	1	1	1	1.5	110.18	0	0	0	0	46520000	0
sp|Q9P25	RCC2	sp|Q9P25	1	17	17	17	45	56.084	6380300000	6210500000	5899100000	6308000000	5562700000	5749300000
sp|Q9P26	KIAA1468	sp|Q9P26	1	1	1	1	1.9	134.63	0	0	0	0	0	0
sp|Q9P26	DIP2B	sp|Q9P26	1	6	6	6	6.5	171.49	160700000	0	196310000	118140000	118890000	148140000
sp|Q9P28	BCCIP	sp|Q9P28	1	7	7	7	31	35.979	1559200000	1284000000	1423400000	1199900000	1110200000	1430500000
sp|Q9P28	STK26	sp|Q9P28	2	10	10	5	30	46.528	749110000	777860000	669360000	707650000	754140000	885630000
sp|Q9P2B	PTGFRN	sp|Q9P2B	1	3	3	3	3.8	98.555	0	0	0	0	111160000	0
sp|Q9P2B	CTTNBP2	sp|Q9P2B	1	2	2	2	6.9	70.157	0	0	0	159670000	0	0
sp|Q9P2E	RRBP1	sp|Q9P2E	2	35	35	35	33	152.45	3348800000	3201100000	3491600000	2973200000	3375900000	3404500000
sp|Q9P2I0	CPSF2	sp|Q9P2I0	1	4	4	4	7.2	88.486	193870000	0	0	0	115290000	0
sp|Q9P2J5	LARS	sp|Q9P2J5	1	36	36	36	38	134.46	5053000000	4990500000	4840500000	5125100000	4982200000	5268300000
sp|Q9P2K	EIF2AK4	sp|Q9P2K	1	2	2	2	1.5	186.91	0	0	0	0	0	30029000
sp|Q9P2N	RBM27	sp|Q9P2N	1	5	4	4	7.6	118.72	219700000	173380000	161560000	203930000	165300000	228860000
sp|Q9P2R	ANKFY1	sp|Q9P2R	1	8	8	8	10	128.4	516780000	497000000	468000000	658700000	553800000	432170000
sp|Q9P2R	SUCLA2	sp|Q9P2R	1	12	12	12	33	50.317	1034000000	1165000000	868530000	1070700000	1646200000	1313000000
sp|Q9P2T	GMPR2	sp|Q9P2T	1	3	3	3	13	37.874	0	0	82091000	0	44337000	40203000
sp|Q9P2W	STX18	sp|Q9P2W	1	1	1	1	3.6	38.673	0	0	0	0	0	3188100
sp|Q9P2X	DPM3	sp|Q9P2X	1	1	1	1	13	10.094	166350000	292170000	176720000	0	311440000	312480000
sp|Q9UBB	ATXN10	sp|Q9UBB	1	16	16	16	41	53.488	2711400000	2692100000	2783000000	1795400000	1748000000	1812700000
sp|Q9UBB	MBD2	sp|Q9UBB	1	4	4	4	20	43.254	0	384820000	354930000	0	518540000	0
sp|Q9UBB	NCDN	sp|Q9UBB	1	8	8	8	18	78.863	132590000	0	0	171170000	178620000	172190000
sp|Q9UBC	EPS15L1	sp|Q9UBC	1	7	7	7	11	94.254	236840000	226640000	246640000	267870000	273970000	243130000
sp|Q9UBD	ORC3	sp|Q9UBD	1	4	4	4	8.4	82.253	190620000	154270000	133750000	129860000	138370000	142230000
sp|Q9UBE	SAE1	sp|Q9UBE	1	11	11	11	41	38.449	1123100000	1083700000	1157700000	1084600000	1235100000	1168400000
sp|Q9UBF	COPG2	sp|Q9UBF	1	8	4	4	14	97.621	79823000	100060000	124940000	84348000	100580000	83873000
sp|Q9UBF	RNF7	sp|Q9UBF	1	1	1	1	20	12.683	0	0	0	0	0	35522000
sp|Q9UBG	MRC2	sp|Q9UBG	1	2	2	2	2.8	166.67	0	57009000	0	0	0	0
sp|Q9UBI1	COMMD3	sp|Q9UBI1	1	3	3	3	22	22.151	0	0	0	0	0	102900000
sp|Q9UBI6	GNG12	sp|Q9UBI6	1	3	3	3	57	8.0061	2014400000	1713100000	1523600000	1478500000	2448900000	1639700000
sp|Q9UBK	MTRR	sp|Q9UBK	1	5	5	5	11	80.409	534940000	600640000	377130000	353670000	334080000	346670000
sp|Q9UBL	ASH2L	sp|Q9UBL	1	3	3	3	6.7	68.722	0	0	0	43837000	46940000	0
sp|Q9UBM	PEMT	sp|Q9UBM	1	1	1	1	6.5	22.134	0	0	0	0	30154000	0
sp|Q9UBM	DHCR7	sp|Q9UBM	1	5	5	5	13	54.489	1234900000	669650000	1148300000	957640000	811900000	1013300000
sp|Q9UBP	SPAST	sp|Q9UBP	1	1	1	1	3.9	67.196	0	0	0	0	0	29532000
sp|Q9UBP	METTL1	sp|Q9UBP	1	3	3	3	26	31.471	0	0	255840000	0	380520000	0
sp|Q9UBP	GULP1	sp|Q9UBP	1	3	3	3	9.2	34.49	0	0	311750000	0	0	259200000
sp|Q9UBQ	VPS29	sp|Q9UBQ	1	6	6	6	33	20.505	1227200000	1476000000	1308500000	1125100000	1326100000	1343900000

sp|Q9UBQ	EIF3K	sp|Q9UBQ	1	6	6	6	34	25.059	2090600000	1793700000	1735200000	1838800000	2051000000	1911600000
sp|Q9UBQ	GRHPR	sp|Q9UBQ	1	10	10	10	46	35.668	1911900000	2596700000	2404100000	2032200000	2136200000	2500200000
sp|Q9UBR	CTSZ	sp|Q9UBR	1	6	6	6	24	33.868	427760000	596720000	379750000	414770000	463270000	471100000
sp|Q9UBS	DNAJB11	sp|Q9UBS	1	9	9	9	29	40.513	1059600000	908330000	1157100000	1390000000	1296800000	1183700000
sp|Q9UBT	UBA2	sp|Q9UBT	1	21	21	21	47	71.223	2886100000	3213200000	3106800000	2767300000	3159500000	2400800000
sp|Q9UBU	MORF4L1	sp|Q9UBU	1	7	7	6	31	41.473	537700000	476300000	350140000	282250000	395970000	0
sp|Q9UBU	NXF1	sp|Q9UBU	1	11	11	11	25	70.182	668640000	697970000	724060000	527130000	606150000	838300000
sp|Q9UBV	SEL1L	sp|Q9UBV	1	3	3	3	6	88.754	135560000	0	100590000	156960000	126230000	88961000
sp|Q9UBV	PEF1	sp|Q9UBV	1	3	3	3	12	30.381	0	0	0	0	193970000	0
sp|Q9UBW	COPS7A	sp|Q9UBW	1	7	7	7	34	30.276	342040000	357300000	419460000	434630000	442890000	327980000
sp|Q9UBX	SLC25A1(	sp|Q9UBX	1	8	8	8	37	31.282	864530000	511480000	723250000	804240000	1007300000	943180000
sp|Q9UDW	UQCR10	sp|Q9UDW	1	2	2	2	38	7.3084	408070000	0	415300000	0	364980000	0
sp|Q9UDY	TJP2	sp|Q9UDY	1	7	7	7	6.8	133.96	477120000	416030000	334290000	364640000	323100000	442970000
sp|Q9UDY	DNAJB4	sp|Q9UDY	1	11	10	10	40	37.806	1026300000	1002200000	855010000	901300000	988110000	1067800000
sp|Q9UDY	MALT1	sp|Q9UDY	1	2	2	2	3.8	92.271	0	0	0	0	0	45513000
sp|Q9UER	DAXX	sp|Q9UER	1	3	3	3	8.9	81.372	0	0	0	0	53689000	0
sp|Q9UEU	VTI1B	sp|Q9UEU	1	2	2	2	13	26.688	35510000	0	46236000	0	53632000	0
sp|Q9UEW	STK39	sp|Q9UEW	1	4	2	2	9.4	59.473	0	0	0	58318000	0	0
sp|Q9UEY	ADD3	sp|Q9UEY	1	5	5	5	11	79.154	191340000	244750000	190040000	161390000	180850000	193470000
sp|Q9UFC	LRWD1	sp|Q9UFC	1	5	5	5	12	70.86	159500000	238480000	128490000	284140000	206190000	163270000
sp|Q9UFN	NIPSNAP:	sp|Q9UFN	1	1	1	1	7.3	28.466	0	0	0	0	0	10955000
sp|Q9UFW	CGGBP1	sp|Q9UFW	1	1	1	1	6	18.82	0	0	0	0	0	119870000
sp|Q9UG6	ABCF2	sp|Q9UG6	1	15	15	15	29	71.289	1673700000	1693300000	2035700000	1904600000	1594900000	1594700000
sp|Q9UGI	TES	sp|Q9UGI	1	19	19	19	54	47.996	2794600000	2966500000	2571100000	2890100000	2934200000	2959300000
sp|Q9UGJ	TUBGCP4	sp|Q9UGJ	1	3	3	3	6.9	76.088	102810000	121280000	126880000	0	0	113640000
sp|Q9UGM	WARS2	sp|Q9UGM	1	2	2	2	7.2	40.146	0	0	0	0	35675000	0
sp|Q9UGP	LIMD1	sp|Q9UGP	1	2	2	2	4.4	72.189	93812000	67384000	0	68844000	0	85538000
sp|Q9UGP	SEC63	sp|Q9UGP	1	12	12	12	23	87.996	759880000	842200000	714070000	970160000	1154300000	1184800000
sp|Q9UGR	ZC3H7B	sp|Q9UGR	1	1	1	1	1.1	111.58	0	0	0	0	0	17197000
sp|Q9UGU	TCF20	sp|Q9UGU	1	2	2	2	1.5	211.77	0	0	0	0	29278000	0
sp|Q9UGV	NDRG3	sp|Q9UGV	1	4	4	4	18	41.408	215480000	289760000	187130000	0	204050000	171420000
sp|Q9UGY	NOL12	sp|Q9UGY	1	1	1	1	5.6	24.663	0	24171000	0	0	0	0
sp|Q9UH6	ARMCX3	sp|Q9UH6	1	4	4	4	17	42.5	202320000	174710000	141190000	172220000	206940000	157940000
sp|Q9UH6	SWAP70	sp|Q9UH6	1	6	6	6	13	68.997	252190000	258880000	189830000	258000000	238730000	191450000
sp|Q9UH9	SUN2	sp|Q9UH9	1	6	6	6	13	80.31	102230000	162300000	133530000	89716000	74856000	92750000
sp|Q9UHA	LAMTOR3	sp|Q9UHA	1	3	3	3	44	13.623	187270000	433010000	248430000	236320000	253620000	311840000
sp|Q9UHB	LIMA1	sp|Q9UHB	1	10	10	10	17	85.225	836090000	851830000	742340000	708360000	753980000	782370000
sp|Q9UHB	AFF4	sp|Q9UHB	1	1	1	1	1.5	127.46	0	0	0	0	0	29154000
sp|Q9UHB	SRP68	sp|Q9UHB	1	13	13	13	28	70.729	1279500000	1249900000	1376400000	1232800000	1270500000	1333100000

sp|Q9UHD	CHORDC	sp|Q9UHD	1	14	14	14	55	37.489	4695900000	5042200000	5620500000	4920900000	6452300000	5063600000
sp|Q9UHD	TBK1	sp|Q9UHD	1	2	2	2	4.8	83.641	0	0	0	0	0	38884000
sp|Q9UHD	SEPT9	sp|Q9UHD	1	23	23	23	47	65.401	3898500000	4441200000	3956000000	3655600000	3639100000	3805800000
sp|Q9UHD	UBQLN2	sp|Q9UHD	1	9	6	6	27	65.695	1149700000	993290000	1282800000	797480000	805930000	732580000
sp|Q9UHG	PCYOX1	sp|Q9UHG	1	10	10	10	25	56.639	696160000	933490000	779490000	839830000	781590000	689630000
sp|Q9UHI	DDX20	sp|Q9UHI	1	10	10	10	19	92.239	208400000	358180000	392840000	546170000	666790000	403790000
sp|Q9UHK	AMACR	sp|Q9UHK	1	2	2	2	7.6	42.386	0	0	0	0	46381000	0
sp|Q9UHN	TMEM2	sp|Q9UHN	1	1	1	1	1	154.37	0	0	0	31086000	0	0
sp|Q9UHQ	CYB5R1	sp|Q9UHQ	1	6	6	6	22	34.094	1295600000	1259600000	1349500000	1652000000	1915500000	1822100000
sp|Q9UHR	BAIAP2L1	sp|Q9UHR	1	3	3	3	8.4	56.882	120240000	0	0	122700000	126470000	0
sp|Q9UHR	SAP30BP	sp|Q9UHR	1	2	2	2	8.4	33.87	0	91083000	0	96887000	0	126460000
sp|Q9UHR	ZNHIT2	sp|Q9UHR	1	4	4	4	12	42.883	0	115510000	0	0	0	0
sp|Q9UHV	PFDN2	sp|Q9UHV	1	6	6	6	40	16.648	1393200000	1763900000	1055000000	1690900000	1736300000	1665500000
sp|Q9UHW	GPN3	sp|Q9UHW	1	1	1	1	6.3	32.761	0	0	0	0	0	18015000
sp|Q9UHX	PUF60	sp|Q9UHX	1	18	18	18	39	59.875	4068100000	3874200000	4501900000	3854300000	3923800000	3476800000
sp|Q9UHY	NRBP1	sp|Q9UHY	1	5	5	5	13	59.844	132270000	144960000	166710000	96143000	74374000	135260000
sp|Q9UHY	ENOPH1	sp|Q9UHY	1	4	4	4	28	28.932	300070000	320100000	218590000	216600000	226840000	166590000
sp|Q9UI09	NDUFA12	sp|Q9UI09	1	2	2	2	17	17.114	101800000	95080000	85712000	43526000	78208000	74291000
sp|Q9UI10	EIF2B4	sp|Q9UI10	1	6	6	6	20	57.557	150010000	104100000	118910000	165790000	0	138960000
sp|Q9UI12	ATP6V1H	sp|Q9UI12	1	5	5	5	16	55.882	346600000	286880000	287820000	213560000	178930000	337770000
sp|Q9UI15	TAGLN3	sp|Q9UI15	1	2	1	1	14	22.472	0	0	0	0	0	32203000
sp|Q9UI26	IPO11	sp|Q9UI26	1	8	8	8	12	112.53	322230000	355890000	433620000	459300000	340360000	366180000
sp|Q9UI30	TRMT112	sp|Q9UI30	1	6	6	6	59	14.199	1822600000	2225800000	2017800000	1616900000	1565800000	1831400000
sp|Q9UIA	XPO7	sp|Q9UIA	1	13	13	13	18	123.91	621220000	716840000	655810000	614540000	609120000	766250000
sp|Q9UID	VPS51	sp|Q9UID	1	5	5	5	10	86.041	165570000	136670000	144270000	178520000	192730000	178550000
sp|Q9UIG	BAZ1B	sp|Q9UIG	1	17	17	17	17	170.9	1111500000	1241800000	1093400000	1058800000	1293100000	1140800000
sp|Q9UII4	HERC5	sp|Q9UII4	1	2	2	2	2.8	116.85	0	87784000	0	0	76878000	0
sp|Q9UIJ7	AK3	sp|Q9UIJ7	1	4	4	4	22	25.565	0	293050000	402670000	436220000	558170000	435240000
sp|Q9UIL1	SCOC	sp|Q9UIL1	1	2	2	2	28	18.045	0	0	0	20496000	0	0
sp|Q9UIQ	LNPEP	sp|Q9UIQ	1	6	6	6	8.1	117.35	246310000	236700000	0	173340000	249780000	175700000
sp|Q9UIV1	CNOT7	sp|Q9UIV1	1	4	4	4	23	32.745	144560000	122110000	91258000	132610000	118790000	223000000
sp|Q9UJ4	RABGEF1	sp|Q9UJ4	1	3	3	3	6.1	79.37	0	0	0	0	0	64939000
sp|Q9UJ7	NAGK	sp|Q9UJ7	1	6	6	6	25	37.375	273250000	380310000	254480000	367650000	314210000	299890000
sp|Q9UJ7	ANXA10	sp|Q9UJ7	1	4	4	4	18	37.277	123390000	64712000	99032000	161250000	103090000	123990000
sp|Q9UJ8	HACL1	sp|Q9UJ8	1	6	6	6	17	63.728	163780000	0	0	222110000	167760000	0
sp|Q9UJA	TRMT6	sp|Q9UJA	1	3	3	3	8.2	55.799	0	0	0	0	0	117880000
sp|Q9UJK	TSR3	sp|Q9UJK	1	2	2	2	12	33.596	0	23656000	0	0	0	0
sp|Q9UJS	SLC25A1	sp|Q9UJS	1	20	20	17	41	74.175	2735300000	2415100000	2482700000	2773500000	2854400000	2689000000
sp|Q9UJU	DBNL	sp|Q9UJU	1	9	9	9	30	48.207	872080000	781310000	870090000	1081000000	1004000000	839310000

sp|Q9UJV	DDX41	sp|Q9UJV	1	2	2	2	5	69.837	0	0	0	0	0	33561000
sp|Q9UJW	DCTN4	sp|Q9UJW	1	7	7	7	29	52.337	266760000	278310000	235540000	251580000	238570000	241250000
sp|Q9UJX	CDC23	sp|Q9UJX	1	9	9	9	24	68.833	209350000	180480000	169790000	220630000	236820000	266810000
sp|Q9UJX	ANAPC7	sp|Q9UJX	1	7	7	7	17	66.855	305620000	298790000	345090000	251760000	299030000	329410000
sp|Q9UJX	ANAPC5	sp|Q9UJX	1	3	3	3	6.2	85.076	0	0	63139000	0	0	0
sp|Q9UJX	ANAPC2	sp|Q9UJX	1	1	1	1	1.7	93.827	0	0	0	17264000	0	0
sp|Q9UJY	HSPB8	sp|Q9UJY	1	3	3	3	19	21.604	0	154400000	0	0	314670000	380660000
sp|Q9UJZ	STOML2	sp|Q9UJZ	1	12	12	12	58	38.534	2692800000	2641800000	2869100000	3090600000	3429900000	2943500000
sp|Q9UK4	VPS28	sp|Q9UK4	1	4	4	4	26	25.425	0	344670000	282650000	436490000	440980000	336140000
sp|Q9UK4	LSM7	sp|Q9UK4	1	2	2	2	33	11.602	39842000	0	134540000	43072000	0	214740000
sp|Q9UK5	DBR1	sp|Q9UK5	1	2	2	2	4.4	61.554	54206000	0	0	0	0	0
sp|Q9UK6	FAM208A	sp|Q9UK6	1	4	4	4	5.2	189.03	113130000	117420000	0	0	98722000	0
sp|Q9UK7	JPT1	sp|Q9UK7	1	5	5	5	64	16.014	4320000000	3805500000	3814600000	2847700000	3258400000	3372100000
sp|Q9UKD	MRTO4	sp|Q9UKD	1	11	11	11	54	27.56	2937200000	2248400000	2418700000	2258600000	2152600000	2722400000
sp|Q9UKF	CPSF3	sp|Q9UKF	1	9	9	9	20	77.485	330180000	413270000	315140000	342580000	319720000	253090000
sp|Q9UKG	APPL1	sp|Q9UKG	1	6	6	6	11	79.663	71324000	110710000	0	0	0	0
sp|Q9UKI	CDC42EP	sp|Q9UKI	1	2	2	2	13	27.678	0	43455000	49370000	0	33054000	0
sp|Q9UKK	PARP4	sp|Q9UKK	1	3	3	3	1.5	192.59	0	0	0	137050000	0	0
sp|Q9UKK	NUDT5	sp|Q9UKK	1	12	12	12	60	24.327	3064400000	2181000000	2629400000	2523100000	2704500000	2307200000
sp|Q9UKL	RCOR1	sp|Q9UKL	2	2	2	2	7.8	53.327	64853000	235760000	166000000	0	237710000	183600000
sp|Q9UKM	MAN1B1	sp|Q9UKM	1	1	1	1	2.4	79.579	0	0	0	0	54051000	0
sp|Q9UKM	RALY	sp|Q9UKM	1	11	11	11	36	32.463	2712700000	2495400000	2633500000	2882600000	2777300000	3609500000
sp|Q9UKN	GTF3C4	sp|Q9UKN	1	15	15	15	25	91.981	718650000	696730000	616450000	653440000	802990000	715570000
sp|Q9UKR	ERG28	sp|Q9UKR	1	2	2	2	12	15.864	0	0	0	0	0	53879000
sp|Q9UKS	PACSIN3	sp|Q9UKS	1	12	12	12	41	48.486	921350000	855170000	834400000	831570000	703480000	845050000
sp|Q9UKV	ACIN1	sp|Q9UKV	1	18	18	18	20	151.86	1955300000	1768900000	1906300000	1887000000	1824900000	1737800000
sp|Q9UKV	AGO2	sp|Q9UKV	4	6	6	6	12	97.207	177610000	0	198690000	89574000	133010000	104780000
sp|Q9UKX	NUP50	sp|Q9UKX	1	9	9	9	29	50.144	578550000	711630000	480470000	565880000	716240000	628230000
sp|Q9UKY	CDV3	sp|Q9UKY	1	11	11	11	69	27.335	1738300000	1906000000	1345200000	1904800000	2459100000	2071900000
sp|Q9UL1	BAG5	sp|Q9UL1	1	3	3	3	8.9	51.199	67567000	29936000	91394000	72679000	62043000	105440000
sp|Q9UL2	RAB21	sp|Q9UL2	1	6	6	6	34	24.347	667610000	539420000	729150000	791500000	725680000	1251200000
sp|Q9UL2	RAB22A	sp|Q9UL2	1	2	1	1	17	21.855	0	0	0	0	0	38631000
sp|Q9UL4	PNMA2	sp|Q9UL4	1	1	1	1	2.7	41.509	0	0	0	0	0	11205000
sp|Q9UL4	PSME2	sp|Q9UL4	1	11	11	11	52	27.401	1066100000	1306900000	981010000	1025700000	1087600000	863790000
sp|Q9ULA	DNPEP	sp|Q9ULA	1	4	4	4	12	52.428	148490000	128240000	169260000	147640000	0	187120000
sp|Q9ULC	RAB23	sp|Q9ULC	1	3	3	3	22	26.659	127380000	103910000	117390000	126770000	159550000	0
sp|Q9ULC	MCTS1	sp|Q9ULC	1	7	7	7	53	20.555	786700000	978950000	865410000	748050000	840280000	657130000
sp|Q9ULH	KIDINS22	sp|Q9ULH	1	2	2	2	1.6	196.54	0	0	683240000	0	0	952590000
sp|Q9ULI	HEG1	sp|Q9ULI	1	1	1	1	0.9	147.46	0	0	0	0	0	29266000

sp|Q9ULK	MED23	sp|Q9ULK	1	1	1	1	1.2	156.47	0	0	0	0	35879000	0
sp|Q9ULP	TBC1D24	sp|Q9ULP	1	2	2	2	4.7	62.919	0	0	0	137490000	0	0
sp|Q9ULR	ISY1	sp|Q9ULR	1	4	4	4	21	32.992	372390000	229280000	229410000	242510000	236050000	172980000
sp|Q9ULT	HECTD1	sp|Q9ULT	1	13	13	13	8.5	289.38	402970000	366010000	308010000	396690000	302830000	241900000
sp|Q9ULV	CIZ1	sp|Q9ULV	1	1	1	1	1.9	100.04	0	0	0	0	16401000	0
sp|Q9ULV	CORO1C	sp|Q9ULV	1	18	18	18	45	53.248	3418100000	2763300000	3413700000	2951700000	3545400000	3511500000
sp|Q9ULW	TPX2	sp|Q9ULW	1	18	18	18	33	85.652	595660000	653870000	584350000	1113500000	1052800000	930430000
sp|Q9ULX	NOB1	sp|Q9ULX	1	6	6	6	22	46.674	480630000	389850000	505190000	349190000	451830000	418530000
sp|Q9UM0	TMCO1	sp|Q9UM0	1	4	4	4	17	21.175	410500000	439860000	318600000	296740000	356240000	449360000
sp|Q9UMF	DDX19B	sp|Q9UMF	1	9	1	1	24	53.926	0	0	0	0	0	29426000
sp|Q9UMS	NFU1	sp|Q9UMS	1	4	4	4	19	28.462	197040000	150540000	193960000	201440000	252330000	178720000
sp|Q9UMS	PRPF19	sp|Q9UMS	1	16	16	16	55	55.18	7450700000	5695500000	7707000000	6876000000	6588200000	5626700000
sp|Q9UMX	UBQLN1	sp|Q9UMX	1	12	12	8	41	62.518	2326900000	2420700000	2295800000	2072000000	2358500000	2028500000
sp|Q9UMX	NENF	sp|Q9UMX	1	4	4	4	41	18.856	371860000	386400000	399600000	304360000	461250000	323990000
sp|Q9UMY	NOL7	sp|Q9UMY	1	1	1	1	3.9	29.426	0	0	0	0	0	319380000
sp|Q9UMY	SNX12	sp|Q9UMY	1	8	7	7	58	19.73	1245900000	1373400000	1162100000	1022600000	1076800000	1346100000
sp|Q9UN3	VPS4A	sp|Q9UN3	1	5	4	4	14	48.897	121400000	215450000	141130000	140730000	195680000	184640000
sp|Q9UN7	SOX13	sp|Q9UN7	1	1	1	1	2.3	69.228	0	0	0	0	2606100000	0
sp|Q9UN8	G3BP2	sp|Q9UN8	1	11	10	10	32	54.12	1557800000	1606900000	1860800000	1679800000	1842000000	1486200000
sp|Q9UNE	STUB1	sp|Q9UNE	1	8	8	8	30	34.856	0	495810000	369830000	378840000	376200000	508400000
sp|Q9UNF	PACSIN2	sp|Q9UNF	1	13	13	13	36	55.738	838170000	690030000	915340000	732520000	792540000	837390000
sp|Q9UNF	MAGED2	sp|Q9UNF	3	17	17	17	45	64.953	969980000	1066000000	978850000	953250000	805120000	857640000
sp|Q9UNH	SNX6	sp|Q9UNH	1	12	12	12	33	46.648	583150000	720050000	797540000	726910000	671220000	667130000
sp|Q9UNI	DUSP12	sp|Q9UNI	1	3	3	3	17	37.687	85700000	122220000	117620000	225050000	111850000	108130000
sp|Q9UNK	STX8	sp|Q9UNK	1	1	1	1	7.6	26.906	0	0	0	0	0	15181000
sp|Q9UNL	SSR3	sp|Q9UNL	1	1	1	1	7.6	21.08	446920000	391310000	366190000	575670000	117520000	376630000
sp|Q9UNM	PSMD13	sp|Q9UNM	1	17	17	17	50	42.945	3666400000	3786700000	3776400000	3904200000	3696300000	3447800000
sp|Q9UNN	FAF1	sp|Q9UNN	1	5	5	5	12	73.953	0	255960000	261770000	0	284820000	226110000
sp|Q9UNN	PROCR	sp|Q9UNN	1	2	2	2	9.2	26.671	156480000	179700000	173850000	133760000	0	0
sp|Q9UNP	PPIE	sp|Q9UNP	1	5	5	4	24	33.43	383650000	425020000	230940000	523310000	0	273000000
sp|Q9UNQ	DIMT1	sp|Q9UNQ	1	9	9	9	40	35.236	441760000	499670000	443240000	438360000	337560000	241090000
sp|Q9UNS	COPS3	sp|Q9UNS	1	12	12	12	42	47.873	1803800000	2017100000	1605500000	1531000000	1770700000	1746200000
sp|Q9UNX	RPL26L1	sp|Q9UNX	1	9	2	2	45	17.256	517310000	478560000	488710000	535860000	430340000	629910000
sp|Q9UNX	WDR3	sp|Q9UNX	1	17	17	17	25	106.1	506800000	397560000	549320000	544340000	469120000	498290000
sp|Q9UNZ	NSFL1C	sp|Q9UNZ	1	9	9	9	34	40.572	679300000	749050000	809530000	886220000	786350000	556290000
sp|Q9UPN	MACF1	sp|Q9UPN	1	19	18	18	4	838.3	403870000	490140000	375430000	438780000	464820000	511820000
sp|Q9UPN	CEP131	sp|Q9UPN	1	1	1	1	1.3	122.15	0	0	0	24318000	0	0
sp|Q9UPN	PPP6R1	sp|Q9UPN	1	5	5	5	11	96.723	0	0	0	0	113270000	151300000
sp|Q9UPN	TRIM33	sp|Q9UPN	1	4	4	4	6.7	122.53	0	0	0	270820000	235940000	171390000

sp	Q9UPQ	LIMCH1	sp	Q9UPQ	1	16	16	16	22	121.87	715700000	668360000	812700000	664740000	645010000	322100000
sp	Q9UPQ	TNRC6B	sp	Q9UPQ	1	2	2	2	2.7	194	0	0	63070000	0	0	0
sp	Q9UPT	EXOC7	sp	Q9UPT	1	4	4	4	6.9	83.381	166490000	0	0	199640000	0	0
sp	Q9UPT	ZC3H4	sp	Q9UPT	1	5	5	5	7.8	140.26	0	84655000	109800000	92074000	111730000	70287000
sp	Q9UPU	USP24	sp	Q9UPU	1	7	7	7	3.9	294.36	111610000	80984000	0	0	115100000	165400000
sp	Q9UPY	SLC7A11	sp	Q9UPY	1	3	3	3	6	55.422	396580000	438410000	370290000	578740000	483800000	499600000
sp	Q9UQ1	SHOC2	sp	Q9UQ1	1	4	4	4	16	64.887	99732000	0	0	0	0	0
sp	Q9UQ3	SRRM2	sp	Q9UQ3	1	33	33	33	18	299.61	3361800000	3776100000	2551700000	2958300000	2741600000	3040200000
sp	Q9UQ8	PA2G4	sp	Q9UQ8	1	22	22	22	65	43.786	1.4579E+10	1.3819E+10	1.3018E+10	13187000000	12515000000	1.3804E+10
sp	Q9UQ9	SPG7	sp	Q9UQ9	1	2	2	2	4.2	88.234	1101800000	0	1054800000	0	988510000	1194800000
sp	Q9UQE	BAIAP2	sp	Q9UQE	1	3	3	3	7.1	60.867	66003000	105950000	91912000	98373000	99530000	0
sp	Q9UQE	SMC3	sp	Q9UQE	1	34	34	34	36	141.54	3108900000	3204700000	2747600000	2654500000	2854600000	2890800000
sp	Q9Y22	NIP7	sp	Q9Y22	1	3	3	3	24	20.462	386650000	265870000	233400000	333040000	241750000	429150000
sp	Q9Y22	GNE	sp	Q9Y22	1	5	5	5	10	79.274	0	0	111310000	151500000	0	0
sp	Q9Y22	RTRAF	sp	Q9Y22	1	13	13	13	55	28.068	1702200000	1869900000	1586600000	1685200000	1272800000	1434300000
sp	Q9Y23	RUVBL2	sp	Q9Y23	1	25	25	25	55	51.156	5204100000	5788500000	5351800000	6353800000	6199700000	6726100000
sp	Q9Y23	PIN4	sp	Q9Y23	1	2	2	2	32	13.81	413800000	349290000	345400000	288740000	349650000	279810000
sp	Q9Y24	HIGD1A	sp	Q9Y24	1	1	1	1	19	10.143	0	0	0	0	0	132330000
sp	Q9Y24	GINS2	sp	Q9Y24	1	2	2	2	14	21.427	0	0	0	33867000	0	0
sp	Q9Y26	EIF3L	sp	Q9Y26	1	18	18	18	40	66.726	3672300000	3311400000	3841000000	2938300000	3180200000	3104400000
sp	Q9Y26	PLAA	sp	Q9Y26	1	25	25	25	39	87.156	2232100000	2441200000	2820800000	2858200000	2410700000	2734600000
sp	Q9Y26	RUVBL1	sp	Q9Y26	1	17	17	17	51	50.227	4770300000	6018300000	4899500000	5268100000	6048300000	5655400000
sp	Q9Y26	NUDC	sp	Q9Y26	1	16	16	16	54	38.242	6512800000	6860800000	6139700000	6017000000	7945000000	6957700000
sp	Q9Y27	BCS1L	sp	Q9Y27	1	5	5	5	16	47.534	0	297620000	222480000	312140000	230820000	268870000
sp	Q9Y27	VDAC3	sp	Q9Y27	1	8	8	8	39	30.658	2614000000	2750100000	2120600000	2814900000	2837800000	2386000000
sp	Q9Y28	CFL2	sp	Q9Y28	1	8	5	5	60	18.736	1202600000	1110400000	1476700000	1390500000	1222900000	819850000
sp	Q9Y28	ERGIC3	sp	Q9Y28	1	5	5	5	13	43.222	454080000	407900000	435460000	510030000	430420000	330790000
sp	Q9Y28	FARSA	sp	Q9Y28	1	12	12	12	36	57.563	2089000000	1894700000	1693000000	1853300000	1564200000	1803900000
sp	Q9Y29	ASF1A	sp	Q9Y29	1	3	2	2	27	22.968	115980000	103100000	147370000	150270000	0	0
sp	Q9Y29	DRG1	sp	Q9Y29	1	11	11	11	40	40.542	1480700000	1748700000	1496000000	1247300000	1255700000	1451100000
sp	Q9Y2A	NCKAP1	sp	Q9Y2A	1	14	14	14	19	128.79	959950000	963620000	800460000	992360000	958970000	758050000
sp	Q9Y2B	CNPY2	sp	Q9Y2B	1	9	9	9	58	20.652	1672300000	1752100000	1625700000	1790000000	1807000000	2124200000
sp	Q9Y2D	AKAP2	sp	Q9Y2D	1	7	7	7	14	94.659	0	144550000	227160000	156920000	193730000	210020000
sp	Q9Y2G	DNAJC16	sp	Q9Y2G	1	1	1	1	3.3	90.59	0	0	0	0	6224600	0
sp	Q9Y2H	FNDC3A	sp	Q9Y2H	1	5	5	5	7.9	131.85	0	156470000	166450000	131280000	131620000	137480000
sp	Q9Y2K	KDM2A	sp	Q9Y2K	1	6	6	6	7.2	132.79	0	0	386320000	308980000	270410000	228550000
sp	Q9Y2L	DIS3	sp	Q9Y2L	1	18	18	18	27	109	4169300000	4131700000	4971100000	4617100000	4994900000	4098900000
sp	Q9Y2L	TRAPPC8	sp	Q9Y2L	1	1	1	1	1.2	161	0	0	0	0	0	22228000
sp	Q9Y2P	RCL1	sp	Q9Y2P	1	5	5	5	19	40.842	177540000	206230000	155420000	163420000	145960000	144490000

sp|Q9Y2Q	GSTK1	sp|Q9Y2Q	1	4	4	4	24	25.497	432130000	327460000	408140000	524260000	391230000	325010000
sp|Q9Y2Q	LAMTOR2	sp|Q9Y2Q	1	3	3	3	30	13.507	281110000	258190000	179110000	278340000	296040000	213300000
sp|Q9Y2Q	MRPS28	sp|Q9Y2Q	1	2	2	2	11	20.843	0	195520000	231150000	190890000	0	167860000
sp|Q9Y2R	COA3	sp|Q9Y2R	1	2	2	2	18	11.731	0	431960000	0	0	587910000	579270000
sp|Q9Y2R	DDX52	sp|Q9Y2R	1	7	7	7	19	67.497	300560000	312910000	268980000	195590000	275400000	302300000
sp|Q9Y2R	MRPS17	sp|Q9Y2R	1	5	5	5	59	14.502	615550000	583970000	486910000	660370000	490870000	496570000
sp|Q9Y2R	MRPS7	sp|Q9Y2R	1	10	10	10	49	28.134	517650000	700110000	418660000	498260000	531060000	544690000
sp|Q9Y2S	TMA7	sp|Q9Y2S	1	2	2	2	30	7.0662	778600000	1002100000	595880000	642560000	820180000	667400000
sp|Q9Y2S	POLDIP2	sp|Q9Y2S	1	5	5	5	22	42.033	342040000	296650000	192290000	266880000	566340000	0
sp|Q9Y2T	AP3M1	sp|Q9Y2T	2	4	4	4	13	46.939	205060000	245490000	261610000	210120000	299870000	309750000
sp|Q9Y2T	GDA	sp|Q9Y2T	1	4	4	4	9.7	51.002	0	85146000	0	0	0	0
sp|Q9Y2U	LEMD3	sp|Q9Y2U	1	5	5	5	8.7	99.996	187500000	251790000	185940000	266260000	277370000	190120000
sp|Q9Y2V	CARHSP1	sp|Q9Y2V	1	6	6	6	71	15.892	1377800000	674420000	622220000	708050000	664500000	779990000
sp|Q9Y2W	THRAP3	sp|Q9Y2W	1	21	21	20	25	108.66	3085300000	3299700000	3186400000	2990400000	3537000000	3185600000
sp|Q9Y2W	WBP11	sp|Q9Y2W	1	15	15	15	31	69.997	1863200000	1732400000	1637100000	1485700000	1535600000	1669300000
sp|Q9Y2X	NOP58	sp|Q9Y2X	1	18	18	18	46	59.578	3264100000	3246100000	3329000000	4110400000	3928200000	3222000000
sp|Q9Y2X	GIT1	sp|Q9Y2X	1	3	2	2	6	84.34	731840000	728760000	530960000	0	550920000	659950000
sp|Q9Y2X	ZNF281	sp|Q9Y2X	1	4	4	4	8.4	96.914	0	0	738410000	471020000	488890000	0
sp|Q9Y2Z	SUGT1	sp|Q9Y2Z	1	15	15	15	49	41.024	2259700000	2378700000	2494900000	2415200000	2444200000	2468800000
sp|Q9Y2Z	YARS2	sp|Q9Y2Z	1	7	7	7	28	53.198	709040000	719510000	612170000	780700000	768710000	769630000
sp|Q9Y30	AMDHD2	sp|Q9Y30	1	1	1	1	2.9	43.747	0	0	0	0	21359000	0
sp|Q9Y30	ACOT9	sp|Q9Y30	1	12	12	12	33	49.901	1282300000	1240000000	1443300000	1474800000	1485100000	1374100000
sp|Q9Y31	AAR2	sp|Q9Y31	1	6	6	6	27	43.472	207720000	173220000	188960000	233540000	233030000	185040000
sp|Q9Y31	NOSIP	sp|Q9Y31	1	6	6	6	28	33.172	527990000	622420000	510110000	585540000	567010000	613170000
sp|Q9Y31	DERA	sp|Q9Y31	1	2	2	2	7.5	35.23	69978000	62691000	58464000	0	51746000	34100000
sp|Q9Y31	MEMO1	sp|Q9Y31	1	7	7	7	37	33.733	305260000	291030000	244300000	313240000	210290000	199180000
sp|Q9Y32	TMX2	sp|Q9Y32	1	3	3	3	14	34.037	0	0	0	173830000	0	0
sp|Q9Y33	LSM2	sp|Q9Y33	1	2	2	2	40	10.834	547610000	740800000	408680000	499350000	219640000	758560000
sp|Q9Y37	SH3GLB1	sp|Q9Y37	1	5	5	5	23	40.796	130810000	141370000	138500000	139730000	162750000	119180000
sp|Q9Y37	NDUFAF1	sp|Q9Y37	1	1	1	1	7	37.763	0	0	0	0	32720000	0
sp|Q9Y37	CAB39	sp|Q9Y37	2	6	6	6	15	39.869	321750000	290840000	264250000	315360000	188260000	170530000
sp|Q9Y38	LUC7L2	sp|Q9Y38	1	16	16	11	38	46.513	4030400000	4613000000	4104200000	3489400000	4294000000	3451000000
sp|Q9Y39	DHRS7	sp|Q9Y39	1	2	2	2	16	38.298	122960000	90386000	0	0	136080000	130540000
sp|Q9Y39	MRPS2	sp|Q9Y39	1	6	6	6	31	33.249	264610000	271170000	247610000	214760000	209210000	205540000
sp|Q9Y3A	UTP11	sp|Q9Y3A	1	3	3	3	12	30.446	48122000	32343000	0	0	108230000	62099000
sp|Q9Y3A	MOB4	sp|Q9Y3A	1	6	6	6	54	26.032	286280000	298920000	255340000	268150000	374840000	389620000
sp|Q9Y3A	RRP7A	sp|Q9Y3A	2	7	7	7	39	32.334	299650000	0	456670000	346050000	429430000	351870000
sp|Q9Y3A	SBDS	sp|Q9Y3A	1	17	17	17	65	28.763	3899500000	3892600000	3764800000	3260500000	3636400000	3646900000
sp|Q9Y3A	TMED5	sp|Q9Y3A	1	3	3	3	13	26.005	273010000	253740000	0	284150000	316110000	263950000

sp|Q9Y3B	EXOSC1	sp|Q9Y3B	1	2	2	2	14	21.452	0	0	0	0	0	166070000
sp|Q9Y3B	TMED7	sp|Q9Y3B	1	4	4	4	24	25.171	842440000	1118400000	689310000	1229700000	1262900000	1191000000
sp|Q9Y3B	SF3B6	sp|Q9Y3B	1	3	3	3	26	14.585	808130000	616380000	633380000	510130000	542410000	860440000
sp|Q9Y3B	MRPL11	sp|Q9Y3B	1	7	7	7	38	20.683	506360000	390100000	457000000	399120000	446790000	460140000
sp|Q9Y3B	REXO2	sp|Q9Y3B	1	7	7	7	35	26.832	524380000	397570000	611940000	491660000	427360000	467100000
sp|Q9Y3B	RRP15	sp|Q9Y3B	1	6	6	6	21	31.484	283730000	198150000	257450000	315430000	340410000	273640000
sp|Q9Y3C	NOP16	sp|Q9Y3C	1	5	5	5	28	21.188	385240000	408170000	281470000	405470000	385540000	454970000
sp|Q9Y3C	TPRKB	sp|Q9Y3C	1	1	1	1	10	19.661	0	0	0	0	0	7965000
sp|Q9Y3C	PPIL1	sp|Q9Y3C	1	4	4	4	30	18.237	1069100000	646120000	747290000	798540000	656800000	613010000
sp|Q9Y3C	MED31	sp|Q9Y3C	1	1	1	1	13	15.805	0	0	0	0	0	30909000
sp|Q9Y3C	UFC1	sp|Q9Y3C	1	4	4	4	22	19.458	279790000	469800000	320260000	299620000	337690000	336060000
sp|Q9Y3D	FAM96B	sp|Q9Y3D	1	3	3	3	30	17.663	132410000	212220000	170660000	0	0	190960000
sp|Q9Y3D	MRPS16	sp|Q9Y3D	1	1	1	1	13	15.345	0	0	0	0	0	23646000
sp|Q9Y3D	FIS1	sp|Q9Y3D	1	4	4	4	34	16.937	519880000	585470000	765170000	550680000	509880000	296080000
sp|Q9Y3D	PAM16	sp|Q9Y3D	1	2	2	2	25	13.825	198220000	285230000	254760000	381550000	318310000	287360000
sp|Q9Y3D	AK6	sp|Q9Y3D	1	5	5	5	41	20.061	233830000	237900000	267150000	244890000	201130000	220130000
sp|Q9Y3D	MRPS23	sp|Q9Y3D	1	6	6	6	36	21.77	428290000	245030000	215530000	291370000	370470000	520540000
sp|Q9Y3E	GOLT1B	sp|Q9Y3E	1	1	1	1	6.5	15.425	0	0	0	0	0	58315000
sp|Q9Y3E	HDGFL3	sp|Q9Y3E	1	2	1	1	12	22.619	0	0	0	0	45184000	0
sp|Q9Y3E	PTRH2	sp|Q9Y3E	1	5	5	5	44	19.193	1012400000	929530000	929600000	953980000	1137300000	857680000
sp|Q9Y3F	STRAP	sp|Q9Y3F	1	19	19	19	71	38.438	6084700000	6325500000	5449100000	5851000000	7289700000	5657100000
sp|Q9Y3I(	RTCB	sp|Q9Y3I(	1	20	20	20	51	55.21	5316900000	4781600000	5557100000	5439200000	5863100000	5561200000
sp|Q9Y3I1	FBXO7	sp|Q9Y3I1	1	4	4	4	11	58.502	118790000	104660000	106820000	101000000	133070000	97043000
sp|Q9Y3L	RAP2C	sp|Q9Y3L	2	5	5	3	40	20.745	149160000	432610000	200950000	313890000	219340000	238540000
sp|Q9Y3P	RABGAP1	sp|Q9Y3P	1	7	7	6	9	121.74	0	90632000	145820000	110770000	130090000	198450000
sp|Q9Y3T	NOC2L	sp|Q9Y3T	1	8	8	8	11	84.918	434390000	347360000	376850000	552240000	406790000	367180000
sp|Q9Y3U	RPL36	sp|Q9Y3U	1	5	5	5	31	12.254	6819900000	4839800000	6216500000	6570500000	7224700000	6674000000
sp|Q9Y3Y	CHTOP	sp|Q9Y3Y	1	5	5	5	24	26.396	813280000	883770000	713350000	1106700000	854910000	863250000
sp|Q9Y3Z	SAMHD1	sp|Q9Y3Z	1	8	8	8	16	72.2	300570000	392970000	325150000	351610000	267210000	235100000
sp|Q9Y44	KNSTRN	sp|Q9Y44	1	2	2	2	11	35.438	127280000	122310000	138570000	206610000	136880000	119200000
sp|Q9Y45	HBS1L	sp|Q9Y45	1	8	8	8	17	75.472	414430000	603900000	295470000	369940000	572540000	430450000
sp|Q9Y49	TLN1	sp|Q9Y49	1	##	112	99	66	269.76	3.0492E+10	2.8656E+10	2.7683E+10	26700000000	29961000000	2.8885E+10
sp|Q9Y4A	TRRAP	sp|Q9Y4A	1	1	1	1	0.3	437.6	0	0	0	0	58458000	0
sp|Q9Y4C	TCAF1	sp|Q9Y4C	1	6	6	6	8.7	102.12	180650000	0	190220000	190070000	0	0
sp|Q9Y4C	RBM19	sp|Q9Y4C	1	2	2	2	3	107.33	0	0	0	0	101670000	0
sp|Q9Y4E	USP15	sp|Q9Y4E	1	7	7	7	12	112.42	374750000	296270000	401430000	376100000	447730000	310970000
sp|Q9Y4G	TLN2	sp|Q9Y4G	1	15	2	2	5.8	271.61	107340000	112430000	96645000	0	0	0
sp|Q9Y4H	IRS2	sp|Q9Y4H	1	2	2	2	3.3	137.33	0	0	0	0	39178000	0
sp|Q9Y4I1	MYO5A	sp|Q9Y4I1	2	2	2	2	1.4	215.4	0	0	0	29563000	0	0

sp|Q9Y4K	CRYBG1	sp|Q9Y4K	1	1	1	1	0.7	188.67	0	0	0	0	0	730730000
sp|Q9Y4L	HYOU1	sp|Q9Y4L	2	29	29	29	36	111.33	3348200000	3321300000	3516300000	3726000000	3991100000	3779400000
sp|Q9Y4P	ATG4B	sp|Q9Y4P	1	5	5	5	21	44.294	124700000	147300000	159410000	133200000	172690000	168140000
sp|Q9Y4P	TBL2	sp|Q9Y4P	1	10	10	10	30	49.797	620100000	523880000	508700000	758580000	705300000	532720000
sp|Q9Y4R	TELO2	sp|Q9Y4R	1	5	5	5	7	91.746	60693000	71056000	97861000	58158000	59794000	59274000
sp|Q9Y4W	LAS1L	sp|Q9Y4W	1	11	11	11	26	83.064	487330000	476570000	453610000	544630000	497280000	506020000
sp|Q9Y4W	AFG3L2	sp|Q9Y4W	1	15	15	15	22	88.583	709900000	920930000	698060000	730420000	936710000	685940000
sp|Q9Y4X	KLF12	sp|Q9Y4X	1	1	1	1	4.7	44.239	0	0	0	57254000	0	0
sp|Q9Y4X	ARIH1	sp|Q9Y4X	1	9	9	9	28	64.117	408750000	560020000	445440000	442220000	337450000	260170000
sp|Q9Y4Y	LSM5	sp|Q9Y4Y	1	2	2	2	46	9.9374	533450000	557960000	562810000	481380000	925740000	468110000
sp|Q9Y4Z	LSM4	sp|Q9Y4Z	1	3	3	3	17	15.35	243140000	0	0	322790000	254050000	255040000
sp|Q9Y50	RNF114	sp|Q9Y50	1	7	7	7	40	25.694	455720000	290340000	331800000	327940000	435140000	420170000
sp|Q9Y51	SAMM50	sp|Q9Y51	1	14	14	14	41	51.976	932440000	900310000	824670000	861520000	946920000	965860000
sp|Q9Y52	PRRC2C	sp|Q9Y52	1	22	22	21	12	316.91	1173200000	1168700000	1185200000	1345800000	1079700000	1316000000
sp|Q9Y54	HSPB11	sp|Q9Y54	1	2	2	2	25	16.297	616590000	481060000	650820000	687800000	479600000	362800000
sp|Q9Y57	PPME1	sp|Q9Y57	1	15	15	15	48	42.315	1062600000	1005400000	842140000	847140000	992220000	758590000
sp|Q9Y5A	YTHDF2	sp|Q9Y5A	1	12	12	7	24	62.333	1578800000	1526300000	1406000000	1324800000	1512100000	1213900000
sp|Q9Y5B	CTDP1	sp|Q9Y5B	1	3	3	3	4.4	104.4	22969000	0	488560000	0	0	0
sp|Q9Y5B	PAXBP1	sp|Q9Y5B	1	1	1	1	1.6	104.8	0	0	0	0	0	26889000
sp|Q9Y5B	NME7	sp|Q9Y5B	1	1	1	1	2.9	42.491	0	0	0	0	0	46757000
sp|Q9Y5B	SUPT16H	sp|Q9Y5B	1	30	30	30	36	119.91	5662400000	5219500000	4890700000	4944700000	4969900000	5598100000
sp|Q9Y5J	UTP18	sp|Q9Y5J	1	8	8	8	21	62.003	483210000	395580000	414740000	332900000	359460000	447380000
sp|Q9Y5J	TIMM9	sp|Q9Y5J	1	2	2	2	35	10.378	373410000	494330000	380710000	507400000	336390000	448890000
sp|Q9Y5J	TIMM8B	sp|Q9Y5J	1	1	1	1	13	9.3435	0	0	0	0	0	66334000
sp|Q9Y5K	UCHL5	sp|Q9Y5K	1	11	11	11	47	37.606	1150700000	1067100000	1130700000	1013500000	966740000	1201400000
sp|Q9Y5K	CD2AP	sp|Q9Y5K	1	10	10	10	23	71.45	778980000	773070000	603840000	548850000	612140000	660530000
sp|Q9Y5K	ATP6V1D	sp|Q9Y5K	1	5	5	5	32	28.262	254240000	253250000	218010000	211260000	241650000	225360000
sp|Q9Y5L	TNPO3	sp|Q9Y5L	1	10	10	10	15	104.2	647510000	725620000	814030000	541140000	722530000	592280000
sp|Q9Y5L	TIMM13	sp|Q9Y5L	1	4	4	4	58	10.5	703950000	672390000	810930000	683460000	785910000	475500000
sp|Q9Y5M	SRPRB	sp|Q9Y5M	1	10	10	10	43	29.702	1421900000	1587900000	1362000000	2053700000	2525200000	2320300000
sp|Q9Y5P	COL4A3B	sp|Q9Y5P	1	3	3	3	6.6	70.834	0	105500000	92210000	80455000	67913000	0
sp|Q9Y5P	GMPPB	sp|Q9Y5P	1	4	4	4	17	39.834	376670000	358410000	429090000	399880000	306530000	294900000
sp|Q9Y5Q	GTF3C5	sp|Q9Y5Q	1	6	6	6	16	59.57	307070000	188370000	228030000	227410000	274830000	274050000
sp|Q9Y5Q	GTF3C3	sp|Q9Y5Q	1	8	8	8	16	101.27	157560000	144260000	176390000	113750000	165870000	165090000
sp|Q9Y5R	TRAPPC1	sp|Q9Y5R	1	1	1	1	11	16.831	0	0	0	0	0	68380000
sp|Q9Y5S	CDC42BP	sp|Q9Y5S	1	3	2	2	1.6	194.31	0	0	0	0	52868000	0
sp|Q9Y5S	RBM8A	sp|Q9Y5S	1	6	6	6	47	19.889	1960700000	2107900000	1981400000	1614300000	2253900000	2148500000
sp|Q9Y5T	USP16	sp|Q9Y5T	1	3	3	3	5	93.569	0	285110000	234980000	0	0	0
sp|Q9Y5U	TSSC4	sp|Q9Y5U	1	2	2	2	7.3	34.325	83203000	0	0	0	82333000	72261000

sp|Q9Y5U	IER3IP1	sp|Q9Y5U	1	2	2	2	34	8.9687	422000000	469650000	657620000	514230000	730250000	523530000
sp|Q9Y5X	SNX9	sp|Q9Y5X	1	4	4	4	12	66.591	670310000	722740000	568240000	424740000	156880000	335030000
sp|Q9Y5X	SNX8	sp|Q9Y5X	1	1	1	1	2.8	52.569	0	0	0	0	0	26317000
sp|Q9Y5X	SNX5	sp|Q9Y5X	1	3	3	3	9.9	46.816	0	0	0	0	0	35059000
sp|Q9Y5Y	NUBP2	sp|Q9Y5Y	1	8	8	8	47	28.825	651120000	881370000	642780000	724840000	665270000	624240000
sp|Q9Y5Z	HEBP2	sp|Q9Y5Z	1	1	1	1	6.3	22.875	0	0	0	0	0	14391000
sp|Q9Y60	MRFAP1	sp|Q9Y60	2	2	2	2	28	14.649	240950000	0	191640000	166320000	212950000	194470000
sp|Q9Y60	PUS1	sp|Q9Y60	1	5	5	5	14	47.47	180990000	145090000	116060000	132520000	192860000	200550000
sp|Q9Y60	LRRFIP2	sp|Q9Y60	1	5	4	4	8.5	82.17	0	146270000	292020000	147150000	0	0
sp|Q9Y61	FHOD1	sp|Q9Y61	1	5	5	5	8.5	126.55	107830000	141310000	128480000	0	130870000	150730000
sp|Q9Y61	PSAT1	sp|Q9Y61	1	17	17	17	49	40.422	4285700000	4177300000	4619900000	4954200000	5170800000	4482900000
sp|Q9Y61	NCOR2	sp|Q9Y61	1	6	6	6	4	274.8	0	116410000	157230000	0	0	0
sp|Q9Y63	NPTN	sp|Q9Y63	1	6	6	6	18	44.387	902630000	525090000	1033900000	719970000	657510000	621390000
sp|Q9Y65	ADGRG1	sp|Q9Y65	1	3	3	3	5.6	77.737	0	0	0	172580000	201950000	0
sp|Q9Y65	SPIN1	sp|Q9Y65	2	3	3	3	17	29.6	0	0	0	183740000	0	134520000
sp|Q9Y67	ALG5	sp|Q9Y67	1	4	4	4	17	36.946	0	0	146460000	158600000	183800000	0
sp|Q9Y67	MRPS18B	sp|Q9Y67	1	5	5	5	32	29.395	350440000	602070000	228280000	500800000	453110000	501750000
sp|Q9Y67	COPG1	sp|Q9Y67	1	29	29	25	46	97.717	4254100000	4708500000	3936800000	4550700000	4368300000	4163400000
sp|Q9Y67	AUP1	sp|Q9Y67	1	4	4	4	12	53.028	269710000	188590000	276930000	245450000	239980000	321000000
sp|Q9Y69	CLIC4	sp|Q9Y69	1	12	12	12	61	28.772	2558100000	2665700000	2650700000	2893600000	2551400000	3016700000
sp|Q9Y69	NFS1	sp|Q9Y69	1	6	6	6	21	50.195	462210000	289100000	356620000	367650000	452680000	371300000
sp|Q9Y6A	CFAP20	sp|Q9Y6A	1	4	4	4	25	22.774	146700000	337150000	0	252060000	0	118830000
sp|Q9Y6A	TACC3	sp|Q9Y6A	1	5	5	5	10	90.359	174790000	222270000	203680000	156350000	158000000	256950000
sp|Q9Y6A	SPCS1	sp|Q9Y6A	1	3	3	3	31	11.805	488270000	536480000	292660000	818410000	905070000	493300000
sp|Q9Y6B	SAR1B	sp|Q9Y6B	1	7	4	4	39	22.41	573160000	433370000	542810000	490040000	636320000	552610000
sp|Q9Y6C	MTCH2	sp|Q9Y6C	1	11	11	11	51	33.331	3173800000	3861500000	3221200000	3808000000	3218400000	3469800000
sp|Q9Y6D	ARFGEF2	sp|Q9Y6D	1	8	8	6	5.9	202.04	192390000	167170000	161190000	146960000	164700000	214380000
sp|Q9Y6D	MAD1L1	sp|Q9Y6D	1	8	8	8	19	83.066	257740000	184100000	215080000	301280000	231230000	216560000
sp|Q9Y6E	STK24	sp|Q9Y6E	1	7	2	2	15	49.307	165020000	131260000	0	111780000	117530000	126730000
sp|Q9Y6E	BZW2	sp|Q9Y6E	1	16	16	15	35	48.162	2773700000	2325300000	2595400000	2591700000	2833300000	2101600000
sp|Q9Y6G	MRPL42	sp|Q9Y6G	1	3	3	3	37	16.661	0	0	0	0	196700000	0
sp|Q9Y6G	COMMD1	sp|Q9Y6G	1	2	2	2	12	22.966	0	0	0	0	0	6005800
sp|Q9Y6G	DYNC1LI1	sp|Q9Y6G	1	13	13	12	32	56.578	985580000	945420000	1072100000	833430000	989870000	861230000
sp|Q9Y6H	CHCHD2	sp|Q9Y6H	2	3	3	3	31	15.512	1459700000	1032900000	1077800000	591560000	609850000	560300000
sp|Q9Y6I3	EPN1	sp|Q9Y6I3	2	6	6	6	16	60.293	270640000	283970000	298700000	261730000	280580000	303210000
sp|Q9Y6I9	TEX264	sp|Q9Y6I9	1	1	1	1	7.7	34.188	0	0	0	0	0	10396000
sp|Q9Y6K	CEPT1	sp|Q9Y6K	1	1	1	1	2.6	46.553	0	0	0	0	0	53406000
sp|Q9Y6K	OAS3	sp|Q9Y6K	1	6	6	6	9	121.17	221130000	270750000	315940000	411550000	297140000	203130000
sp|Q9Y6K	IKBKG	sp|Q9Y6K	1	5	5	5	16	48.197	230030000	209070000	214050000	0	272470000	187770000

sp|Q9Y6M	SLC30A1	sp|Q9Y6M	1	5	5	5	18	55.299	526320000	586700000	308250000	540240000	597190000	645340000
sp|Q9Y6M	SLC4A7	sp|Q9Y6M	1	1	1	1	1.4	136.04	0	0	0	0	0	16348000
sp|Q9Y6M	NDUFB9	sp|Q9Y6M	1	3	3	3	18	21.831	0	0	300400000	0	0	0
sp|Q9Y6N	SQOR	sp|Q9Y6N	1	10	10	10	28	49.96	525770000	475000000	485640000	553330000	618280000	565350000
sp|Q9Y6N	ROBO1	sp|Q9Y6N	1	3	3	3	2.8	180.93	0	88841000	160870000	0	131720000	0
sp|Q9Y6Q	CAPN6	sp|Q9Y6Q	1	1	1	1	1.2	74.576	0	0	0	0	0	164440000
sp|Q9Y6V	DDX49	sp|Q9Y6V	1	2	2	2	8.1	54.226	0	0	0	0	0	100050000
sp|Q9Y6W	WASF2	sp|Q9Y6W	1	3	3	3	8	54.283	115390000	103600000	0	111590000	0	0
sp|Q9Y6X	MAU2	sp|Q9Y6X	1	1	1	1	3.1	69.081	0	0	0	0	0	14853000
sp|Q9Y6Y	IVNS1ABP	sp|Q9Y6Y	1	3	3	3	6.5	71.729	0	0	0	0	0	65422000
sp|Q9Y6Y	SEC23IP	sp|Q9Y6Y	1	14	14	14	17	111.08	649850000	945080000	624330000	759050000	740010000	801280000
