# Supplementary figures and images for: Osthole Induces Apoptosis and Caspase-3/GSDME-Dependent Pyroptosis via NQO1-Mediated ROS Generation in HeLa Cells
Source: Oxid Med Cell Longev. 2022 Jun 8;2022:8585598. doi: 10.1155/2022/8585598 (PMC9200556; doi:10.1155/2022/8585598)

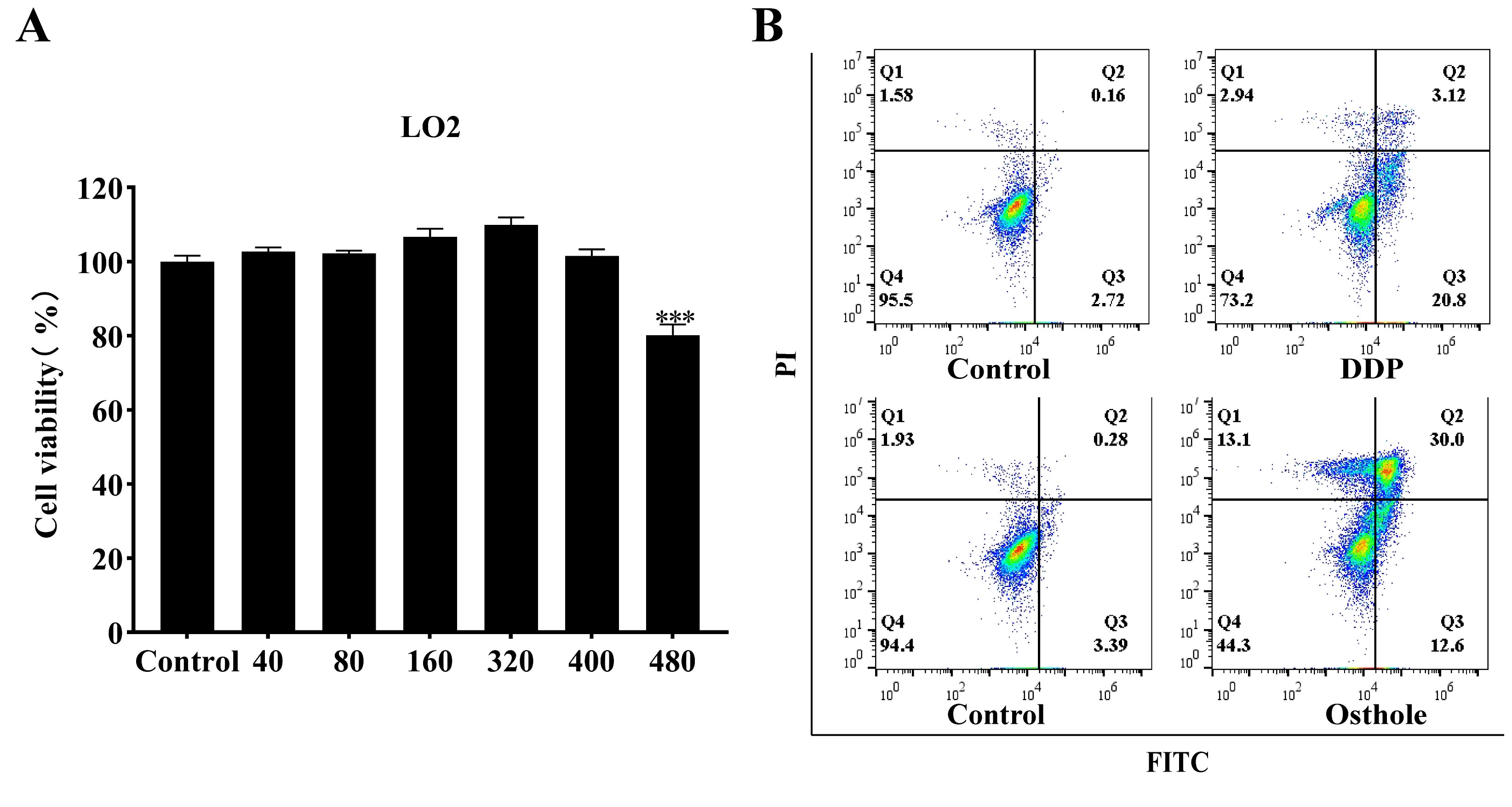

Supplement: Supplementary Materials — Figure S1: the effect of osthole on LO2 cells and the comparison of cell death types. (A) Cell viability of LO2 cells assessed by MTT assay after being treated with osthole for 18h. (B) Cell death was detected by Annexin V-FITC/PI staining by flow cytometry after being treated with DDP and osthole. Figure S2: the expression and activity of NQO1 were inhibited by osthole in HeLa cells. (A) The protein expression of NQO1 was decreased by osthole in HeLa cells. (B) The NQO1 activity was detected used human NQO1 ELISA kit. Figure S3: the protein expression of RIP3 was detected in U87 and HeLa cell. Figure S4: the activation of caspase-5, caspase-1, and GSDMD was detected in osthole-treated cells. [file 8585598.f1.zip › S1.jpg]

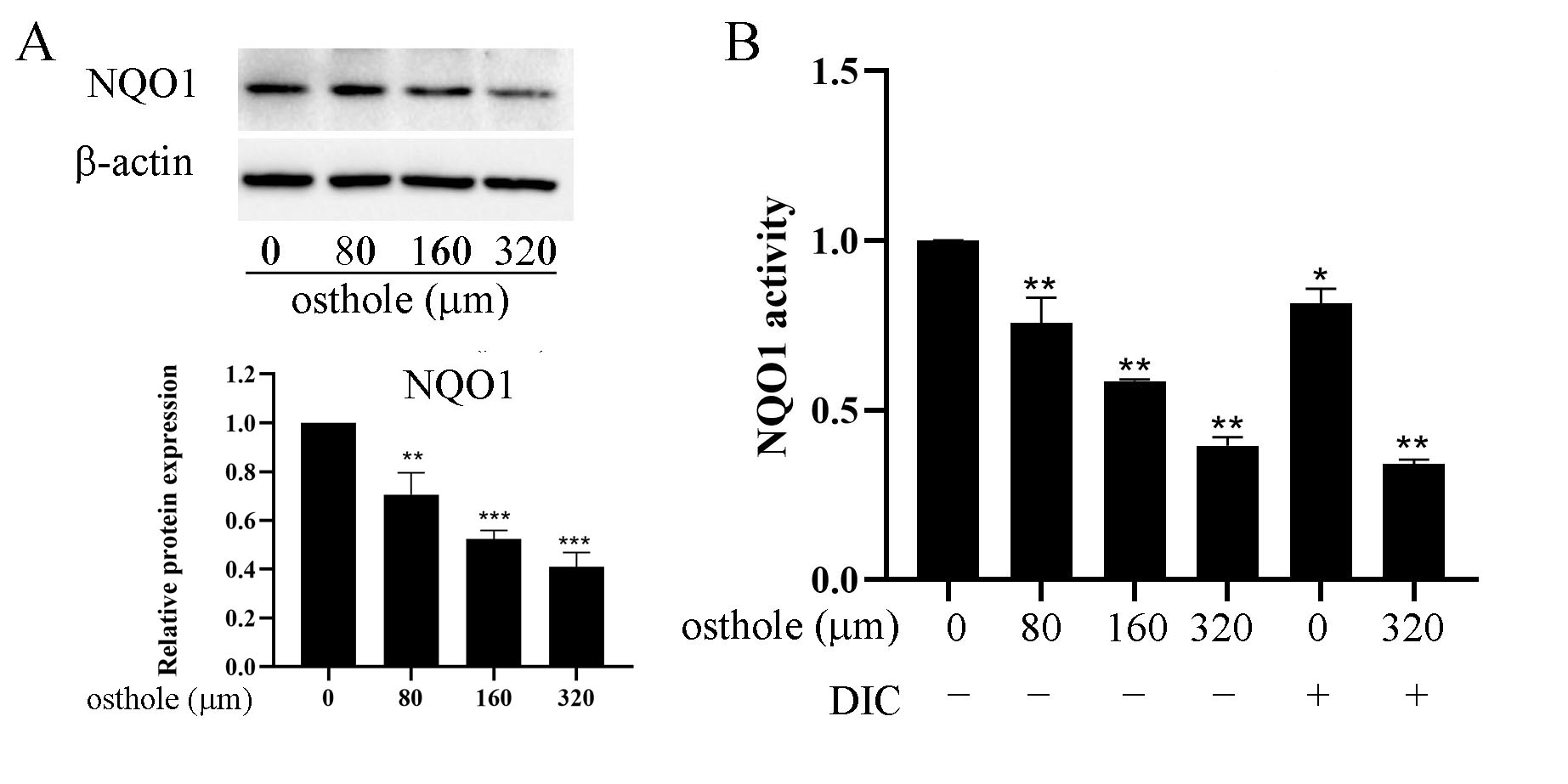

Supplement: Supplementary Materials — Figure S1: the effect of osthole on LO2 cells and the comparison of cell death types. (A) Cell viability of LO2 cells assessed by MTT assay after being treated with osthole for 18h. (B) Cell death was detected by Annexin V-FITC/PI staining by flow cytometry after being treated with DDP and osthole. Figure S2: the expression and activity of NQO1 were inhibited by osthole in HeLa cells. (A) The protein expression of NQO1 was decreased by osthole in HeLa cells. (B) The NQO1 activity was detected used human NQO1 ELISA kit. Figure S3: the protein expression of RIP3 was detected in U87 and HeLa cell. Figure S4: the activation of caspase-5, caspase-1, and GSDMD was detected in osthole-treated cells. [file 8585598.f1.zip › S2 (1).jpg]

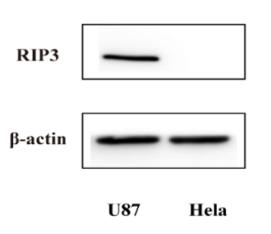

Supplement: Supplementary Materials — Figure S1: the effect of osthole on LO2 cells and the comparison of cell death types. (A) Cell viability of LO2 cells assessed by MTT assay after being treated with osthole for 18h. (B) Cell death was detected by Annexin V-FITC/PI staining by flow cytometry after being treated with DDP and osthole. Figure S2: the expression and activity of NQO1 were inhibited by osthole in HeLa cells. (A) The protein expression of NQO1 was decreased by osthole in HeLa cells. (B) The NQO1 activity was detected used human NQO1 ELISA kit. Figure S3: the protein expression of RIP3 was detected in U87 and HeLa cell. Figure S4: the activation of caspase-5, caspase-1, and GSDMD was detected in osthole-treated cells. [file 8585598.f1.zip › S3.jpg]

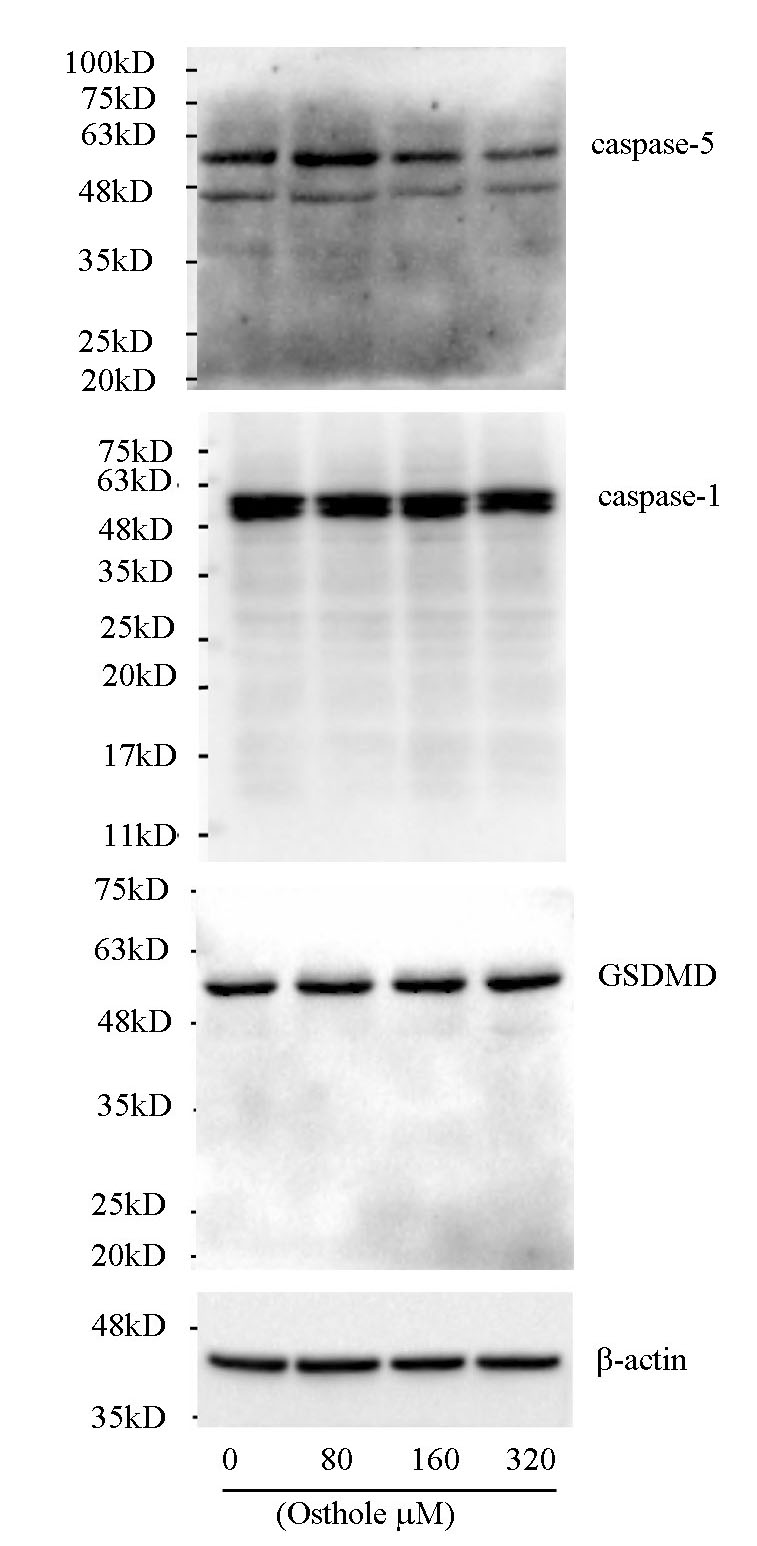

Supplement: Supplementary Materials — Figure S1: the effect of osthole on LO2 cells and the comparison of cell death types. (A) Cell viability of LO2 cells assessed by MTT assay after being treated with osthole for 18h. (B) Cell death was detected by Annexin V-FITC/PI staining by flow cytometry after being treated with DDP and osthole. Figure S2: the expression and activity of NQO1 were inhibited by osthole in HeLa cells. (A) The protein expression of NQO1 was decreased by osthole in HeLa cells. (B) The NQO1 activity was detected used human NQO1 ELISA kit. Figure S3: the protein expression of RIP3 was detected in U87 and HeLa cell. Figure S4: the activation of caspase-5, caspase-1, and GSDMD was detected in osthole-treated cells. [file 8585598.f1.zip › S4.jpg]
